# Functionalization, Fragmentation, and Expansion of *cyclo*‐P_4_R_2_ Ligands

**DOI:** 10.1002/chem.202501305

**Published:** 2025-05-24

**Authors:** Christoph Riesinger, Lisa Zimmermann, Robert Szlosek, Gábor Balázs, Jan Wieneke, Lisa‐Marie Orel, Luis Dütsch, Manfred Scheer

**Affiliations:** ^1^ Institute of Inorganic Chemistry University of Regensburg Universitätsstr. 31 93053 Regensburg Germany

**Keywords:** isolobal, P_4_ activation, phosphorus, reaction mechanism, transition metal

## Abstract

In this study, three isolobal complexes of the form [{L_n_M}(η^3^‐P_4_R_2_)]^+^ ({L_n_M}  = {CpMo(CO)_2_} (**A**), {Cp'''Ni} (**B**), {Cp'''Co}^–^ (**C**), R = Ph, iPr; Cp''' = 1,2,4‐^t^Bu_3_C_5_H_2_) are reacted with nucleophilic carbenes (L). While **C** does not show any reactivity, the cationic complexes **A** and **B** undergo addition reactions. The respective products [CpMo(CO)_2_(η^3^‐P_4_R_2_L)]^+^ (**1a** – **d**) and [Cp'''Ni(η^1:1^‐P_4_R_2_L)]^+^ (**3a** – **d**) show different geometries for the L‐P_3_PR_2_ ligands. Their reactivity towards EtO^–^ results in either a simple addition (for **1**) or a complex addition, ring‐opening, rearrangement sequence (for **3**). Moreover, [Cp'''Ni(η^2^‐IDippPP(OEt)PP^i^Pr_2_)] (**4**) could be methylated by the reaction with MeOTf, which affords an iso‐tetraphosphine ligand, marking the first example of complete functionalization of a polyphosphorus ligand to a complexed phosphine. Mechanistic studies shed light upon the fundamental principles, which differentiate the influence of the isolobal {CpMo(CO)_2_}, {Cp'''Ni}, and {Cp'''Co}^–^ transition metal units. Lastly, **3a**–**d** were chosen as model substrates for further nucleophilic functionalization. In this regard, **3** reacts with [CN]^−^ in a [3+1] fragmentation reaction affording the dimerized species [{Cp'''Ni}_2_(μ,η^1:1:1:1^‐*cyclo*‐P_4_(PR_2_)_2_)] (**6a**: R = Ph, **6b**: R  =  ^i^Pr) together with IDippP‐CN. In contrast, the reaction with [ECO]^−^ (E = P, As) led to an extension of the pnictogen framework yielding [Cp'''Ni(η^1:1^‐EP_4_Ph_2_IDipp)] (**8a**: E = P, **8b**: E = As).

## Graphical abstract

Three isolobal transition metal (TM) units are demonstrated to impact the transformation of coordinated P_4_ ligands in different ways. The observed reactivity primarily depends on the nature of the TM, an effect so far underexplored in phosphorus chemistry. This in‐depth analysis shines light on the underlying mechanistic principles and exploits them to access a plethora of unprecedented fully functionalized polyphosphorus compounds.

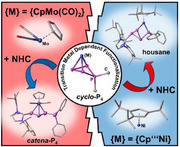

## 1 Introduction

**Scheme 1:**
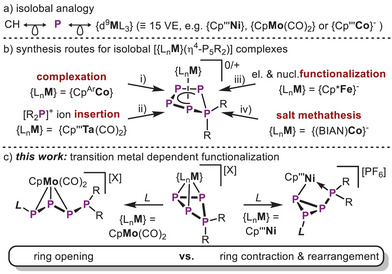
a) isolobal relationship of the CH fragment with the P atom and with {d^9^ **M**L_3_} transition metal fragments; b) stabilization of the same isostructural *cyclo*‐P_5_R_2_ scaffold, independent on the synthetic route and only dependent on the utilization of 14 VE TM units; c) contrasting reactivity based on isolobal TM units; Cp''' = 1,2,4‐*^t^* Bu_3_C_5_H_2_, Cp^Ar^  = C_5_(4‐Et‐C_6_H_4_)_5_, Cp*  = C_5_Me_5_, BIAN = 1,2‐bis(arylimino)acenaphthene diamine.

Transition metal (TM) mediated functionalization of white phosphorus (P_4_) is one of the most promising approaches towards the production of much‐needed organophosphorus compounds.^[^ ^1^, ^2^, ^3^, ^4^, ^5^, ^6^, ^7^ ^]^ This process consists of three steps, which includes i) the transformation of P_4_ with TM precursors, ii) the functionalization of the polyphosphorus (P_n_) ligands and iii) the release of the functionalized P_n_ unit from the TM. The first step has been a research target within the last decades and resulted in the synthesis of a plethora of complexes bearing P_n_ ligands.^[^ ^1^, ^2^, ^3^ ^]^ Many of these P_n_ ligands can be related to their carbocyclic and aromatic counterparts via the isolobal principle,^[^ ^8^ ^]^ which is one of the defining concepts in main group chemistry in general and even more so in phosphorus chemistry.^[^ ^9^, ^10^, ^11^ ^]^ It connects molecular fragments based on the number, symmetry, energy and occupancy of their frontier molecular orbitals. Initially established for the relationship of transition metal (TM) species with organic moieties it was soon transferred to p‐ and f‐block chemistry, where it since allows for predicting the stability, structure, and reactivity of isolobal compounds and molecular fragments, alike.^[^ ^12^ ^]^ Concerning the chemistry of P_n_ ligand complexes, the isolobal relationship between the common hydrocarbon fragment CH, TM units of the general formula {d^9^ML_3_} (M = metal, L = L‐type 2e^–^ donor ligand) bearing 15 valence electrons (VE) and the P atom becomes important (see Scheme 1a) allowing for the synthesis of a plethora of c*yclo*‐P_n_ ligands (n = 3,^[^ ^13^, ^14^, ^15^ ^]^ 4,^[^ ^16^, ^17^, ^18^, ^19^, ^20^, ^21^ ^]^ 5,^[^ ^22^, ^23^, ^24^ ^]^ 6,^[^ ^25^, ^26^, ^27^ ^]^ and 8^[^ ^28^ ^]^). Their preparation is largely based on electronic effects and on fulfilling the 18 VE rule. This results in efficient stabilization of e. g. *cyclo*‐P_3_ ligands by 15 VE and *cyclo*‐P_4_ ligands by 14 VE TM units, respectively.^[^ ^29^ ^]^

One general rule hereby is, that *iso*‐VE TM units will afford similar, if not the same, polyphosphorus scaffolds. Besides those, the stabilization of smaller P_1_ and P_2_ units coordinated to TMs has flourished over the past decades,^[^ ^29^, ^30^, ^31^, ^32^, ^33^, ^34^, ^35^ ^]^ resulting in e. g. the first side‐on complex of molecular P_2_.^[^ ^36^ ^]^ In contrast, the second step in the targeted production of organophosphorus compounds is the subsequent (multi)functionalization of these P_n_ ligands, which remains far less explored.^[^ ^3^ ^]^ One example of successful functionalization includes the synthesis of *cyclo*‐P_5_R_2_ ligands (R = alkyl/aryl substituent) which could be achieved in four different ways: i) complexation of pentaphosphorus cations [P_5_R_2_]^+^ (R = ^i^Pr, Cy) with [Cp^Ar^Co(μ‐X)]_2_ (Cp^Ar^ = C_5_(C_6_H_4_‐4‐Et)_5_; X = Cl, Br),^[^ ^37^ ^]^ ii) insertion of phosphenium ions [R_2_P]^+^ (R = Ph, *^t^* Bu) in a *cyclo*‐P_4_ ligand,^[^ ^38^ ^]^ iii) selective functionalization of one P atom of [Cp*Fe(η^5^‐P_5_)] with a set of main group nucleophiles and subsequent quenching with main group electrophiles^[^ ^39^, ^40^ ^]^ and iv) by salt metathesis reactions of an anionic *cyclo*‐P_4_ complex^[^ ^41^, ^42^ ^]^ or an anionic *cyclo*‐P_4_Ga(nacnac) complex^[^ ^43^ ^]^ with chlorophosphines, respectively. Notably, all these *cyclo*‐P_5_R_2_ ligands are stabilized by 14 VE TM units from across the d‐block (Scheme 1c).^[^ ^37^, ^38^, ^39^, ^40^, ^41^, ^42^, ^43^ ^]^ So far, the potential impact of the respective TM unit on the functionalization of polyphosphorus ligands, beyond its VE count, has been neglected. This motivated us to perform a comparative study focusing on iso‐VE TM units in the transformation of coordinated polyphosphorus ligands. The complexes [{L_n_M}(η^3^‐P_3_)] ({L_n_M} = {CpMo(CO)_2_}^[^ ^13^ ^]^ (**I**), {Cp'''Ni}^[^ ^14^, ^15^ ^]^ (**II**), {Cp'''Co}^–[^ ^44^ ^]^ (**III**), Cp''' = 1,2,4‐*^t^* Bu_3_C_5_H_2_) are especially interesting, as their 15 VE TM units are not only isolobal to each other but also to P_4_ itself. In analogy to P_4_,^[^ ^45^ ^]^ phosphenium ions readily insert into one of the P–P bonds of **I**, **II**,^[^ ^15^ ^]^ and **III**,^[^ ^44^ ^]^ respectively. The resulting ring‐expanded *cyclo*‐P_4_R_2_ ligands in [{L_n_M}(η^3^‐P_4_R_2_)]^+^ ({L_n_M}  = {CpMo(CO)_2_} (**A**), {Cp'''Ni} (**B**), {Cp'''Co}^–^ (**C**)) display important intermediates on the way to value‐added organophosphorus compounds, offering synthetic utility through both, the strained P_4_ cage, as well as the coordination to a TM.^[^ ^15^, ^44^ ^]^ This study compares the reactivity of **A**, **B** and **C** towards N‐heterocyclic carbenes (NHCs) and gives theoretical background to analyze the observed differences in reactivity. Subsequently, the carbene adducts are exposed to other small organic nucleophiles demonstrating the selective (multi)functionalization of polyphosphorus species.

## 2 Results and Discussion

### 2.1 Functionalization of *cyclo*‐P_4_R_2_ Complexes **A** – **C**

**Scheme 2:**
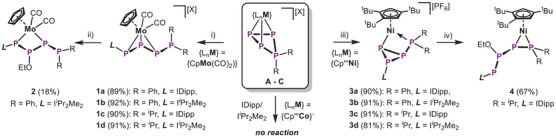
Synthesis of **1** – **4**: i) IDipp, *o*‐DFB, r.t., 16 h or I*^i^* Pr_2_Me_2_, THF, ‐80 °C – r.t., 16 h (R = Ph, *^i^* Pr; [X]^−^ = [OTf]^−^ for **1a/b** or [TEF]^−^ for **1c/d**); ii) KOEt, THF, –80 °C – r.t., 1 h (*L* = I*^i^* Pr_2_Me_2_, R = Ph, [X]^−^ = [OTf]^−^); iii) IDipp, *o*‐DFB, r.t., 16 h or I*^i^* Pr_2_Me_2_, THF, –80 °C – r.t., 16 h (R = Ph, *^i^* Pr); iv) KOEt, THF, –80 °C – r.t., 1 h, – K[PF_6_] (*L* = IDipp, R = *^i^* Pr).

**Figure 1:**
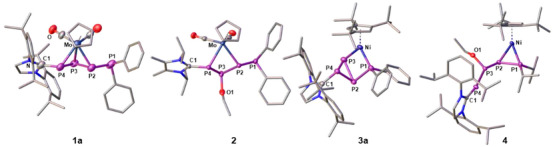
Molecular structures of **1a**, **2**, **3a**, and **4** in the solid state; H atoms and anions are omitted for clarity and ellipsoids are drawn at the 50% probability level.

**Figure 2:**
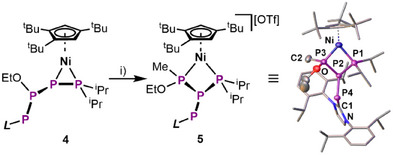
Synthesis and crystal structure of **5**; i) MeOTf, *o*‐/*m*‐DFB, –80 °C – r.t., 2 h (ellipsoids are drawn at the 50% probability level, anions and H atoms are omitted for clarity).

While complexes **B** and **C** have previously been reported,^[^ ^21^, ^46^ ^]^ the analogous compounds [CpMo(CO)_2_(η^3^‐P_4_R_2_)][X] (R = Ph, [X]^–^ = [OTf]^–^ (**A1**); R = *^i^* Pr, [X]^–^ = [TEF]^–^ (**A2**); [OTf]^–^ = [SO_3_CF_3_]^–^, [TEF]^–^ = [Al{OC(CF_3_)_3_}_4_]^–^) were prepared by exposing **I** to equimolar amounts of R_2_PCl in the presence of the Tl^+^ salt of the respective anion in *o*‐DFB (1,2‐difluorobenzene, see ESI). With the isostructural complexes **A** – **C** in hand, their reactivity towards neutral nucleophiles IDipp (1,3‐bis(2,6‐diisopropyl‐phenyl)imidazol‐2‐ylidene) and I*^i^* Pr_2_Me_2_ (1,4‐diisopropyl‐2,3‐dimethylimidazol‐5‐ylidene) was investigated. The same substitution pattern on both, the *cyclo*‐P_4_R_2_ ligand, as well as the NHC was chosen and varied to avoid any bias in reactivity and rule out potential steric/electronic side‐effects. As the anions do not influence the reactivity of the respective cations they were chosen considering optimal crystallinity of the respective products. Reacting **A** with one equivalent of NHC in THF or *o*‐DFB in all cases results in a color change to orange/red, indicating the formation of [CpMo(CO)_2_(η^3^‐P_4_R_2_ *L*)][X] (**1a**: R = Ph, *L* = IDipp, [X]^–^ = [OTf]^–^; **1b**: R = Ph, *L* = I*^i^* Pr_2_Me_2_, [X]^–^ = [OTf]^–^; **1c**: R = *^i^* Pr, *L* = IDipp, [X]^–^  =  [TEF]^–^; **1d**: R = *^i^* Pr, *L* = I*^i^* Pr_2_Me_2_, [X]^–^ = [TEF]^–^). After workup, the products **1a** – **d**, could be isolated as orange solids in yields of up to 92% (Scheme 2). The catena‐type *L*–P_3_–PR_2_ ligand in **1** coordinates to the Mo center in an η^3^‐mode via the allylic P2–P3–P4 unit (Figure 1). Performing the same NHC addition reactions with **B** as well leads to a rapid color change to yellowish green, indicating the formation of [Cp'''Ni(η^1:1^‐P_4_R_2_ *L*)][PF_6_] (**3a**: R = Ph, *L* = IDipp; **3b**: R = Ph, *L* = I*^i^* Pr_2_Me_2_; **3c**: R = *^i^* Pr, *L* = IDipp; **3d**: R = *^i^* Pr, *L* = I*^i^* Pr_2_Me_2_), which could be isolated in up to 91% yield (Scheme 2). This NHC addition results in a completely different geometry of the *L*–P_3_–PR_2_ ligand, which is rearranged and contracted to an NHC substituted P_3_ cycle featuring an exocyclic phosphino group. Notably, the latter is coordinated to the Ni center in **3a** – **d**, while P1 in **1a** – **d** remains uncoordinated. Surprisingly, **C** does not show any reactivity towards NHCs. Even after heating for several hours, there are no signs of conversion (see Figures S65 and S66). This lack of reactivity is attributed to the charge neutral nature of complex **C**, which diminishes its electrophilicity compared to the cationic **A** and **B**. Clearly, this demonstrates the effect of three different transition metal units on the functionalization of the *cyclo*‐P_4_R_2_ ligand. Furthermore, the NHC adducts **1** and **3** were reacted with a small, charged nucleophile, namely EtO^–^. The products [CpMo(CO)_2_(η^1:1^‐I*^i^* Pr_2_Me_2_PP(OEt)P‐PPh_2_)] (**2**) and [Cp'''Ni(η^2^‐IDippPP(OEt)PP*^i^* Pr_2_)] (**4**) both contain the same regio‐isomer of the ligand *L*–PP(OEt)P–PR_2_ (Scheme 2). However, it coordinates the Mo center in an η^1:1^‐fashion via the P2 and P4 atoms in **2**, while it binds to Ni in an η^2^‐mode via P1 and P2 in **4**.The solid state structures of **1** – **4** reveal the difference that the two TM units {CpMo(CO)_2_} and {Cp'''Ni} have on the geometry of the respective P_4_ ligands (Figure 1). While the P1─P2 bonds of the catena P_4_ ligand in **1a** – **d** (2.202(11) – 2.238(1) Å) are in the expected range of single bonds, the respective P2─P3 (2.139(12) – 2.142(1) Å) and P3─P4 (2.110(9) – 2.159(1) Å) bonds are slightly shorter indicating partial double bond character and the allylic nature of the P2–P3–P4 unit.^[^ ^47^ ^]^ The P4─C bond length (1.854(3) – 1.866(2) Å) indicates a P─C single bond, which is in accordance with the twist of the P2–P3–P4 plane against the NHC plane (*δ*(P3‐P4‐C1‐N1) = 138.8(1)° – 155.8(1)°) and dismisses potential electronic conjugation. In contrast, **3a** – **d** reveal an NHC functionalized *cyclo*‐P_3_ ligand with an exocyclic phosphino substituent. The respective P1─P2 bond length (2.190(1) – 2.197(5) Å) is reminiscent of its single bond character. Similarly, the P2─P4 (2.207(1) – 2.224(1) Å) as well as the P3─P4 (2.161(1) – 2.216(2) Å) distances indicate single bonds, while the P2─P3 (2.225(2) – 2.248(1) Å) bonds are slightly elongated. Overall, **3a** – **d** adopt a housane‐type^[^ ^48^ ^]^ scaffold with one of the corners being formed by the {Cp'''Ni} fragment, while the P4 atom occupies the roof position. In contrast, the addition of EtO^–^ in **2** occurs at the P3 atom, maintaining the catena‐type P_4_ ligand. However, all three P─P bonds (2.176(1) – 2.234(1) Å) are now in the range of single bonds.^[^ ^47^ ^]^ Thus, the final *L*–PP(OEt)P–PR_2_ moiety does not show allylic character anymore and coordinates to the Mo center in an η^1:1^‐mode via the P2 and the P4 atom. Addition of EtO^–^ to **3** leads to a much more complicated ring opening/rearrangement reaction (*vide infra*), resulting in the formation of **4**. The *L*–PP(OEt)P–PR_2_ ligand in **4** has the same connectivity as the one in **2**. However, it coordinates to the Ni center in a η^2^‐fashion via the P1 and the P2 atoms. This results in a significant shortening of this bond (2.123(1) Å), indicating double bond character. The P2─P3 (2.254(1) Å) and the P3─P4 (2.183(1) Å) bond lengths are in the range of single bonds.^[^ ^47^ ^]^ Notably, the *L*–PP(OEt)P–PR_2_ ligand in **4** reveals phosphidic character at P2 (formally X‐type ligand towards Ni) posing the potential for addition of an electrophile. Indeed, exposure of **4** to MeOTf leads to functionalization of the P_4_ ligand to finally yield [Cp'''Ni(η^1:1^‐MeP(OEt)P(IDippP)‐P*^i^* Pr_2_)][OTf] (**5**, Figure 2). Unfortunately, **5** co‐crystallizes with an imidazolium salt, which is formed as a side‐product and could thus not be further characterized. However, its molecular structure in the solid state reveals a novel *iso*‐tetraphosphine ligand bound to the Ni center in an η^1:1^‐mode. The central P2 atom connects the –P*^i^* Pr_2_, –P(OEt)Me and the –PIDipp moieties with bond lengths of 2.215(1) Å, 2.175(1) Å, and 2.144(1) Å, respectively. While the presence of two chiral centers (P2 and P3) enables the formation of four stereoisomers, only the diastereomer with the –PIDipp and the –OEt substituents in *cis*‐configuration (regarding the P2─P3 bond) crystallizes as a racemic mixture. Computational data suggests that, despite the phosphidic character of P2 (in **4**), the methylation occurs at P3 and is followed by rearrangement of the –PIDipp substituent (Figure S78). **5** is the first compound featuring an intact and fully saturated polyphosphorus ligand, arising from sequential functionalization of a P_n_ unit in the coordination sphere of a TM.

### 2.2 Mechanistic Investigations

**Figure 3:**
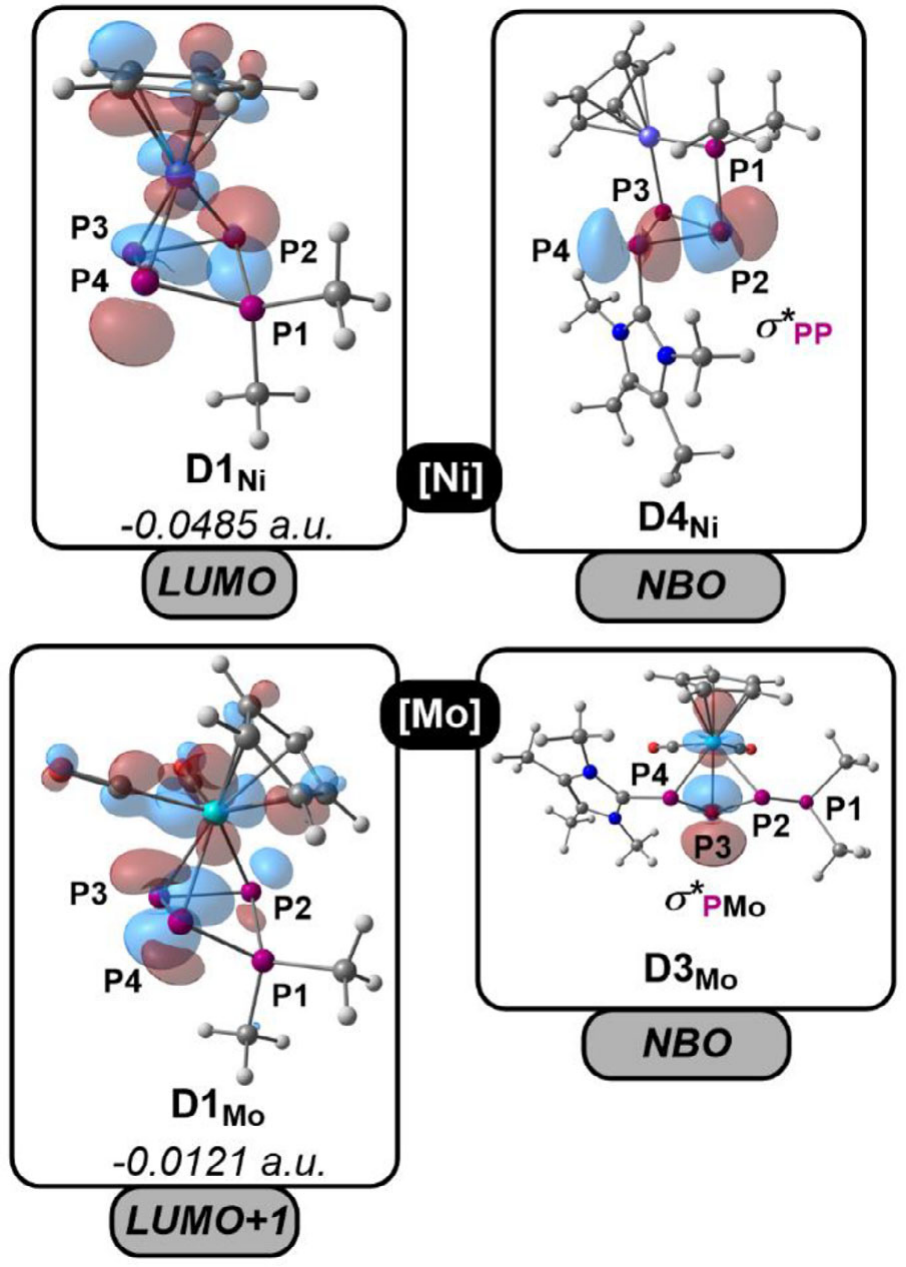
LUMO (**D1^Ni^**) and LUMO+1 (**D1^Mo^**) Kohn‐Sham orbitals and selected NBOs of **D4^Ni^** and **D3^Mo^** (*ω*B97X‐D3BJ/def2‐TZVP level of theory).

To obtain deeper mechanistic insight into the effect of the TM unit on the structure and transformation of the P_4_ ligand in **1** – **4**, a combination of experimental and computational investigations (model system based on [{L_n_M}(η^3^‐P_4_Me_2_)]^+^ (**D1_M_**, {L_n_M} = {CpMo(CO)_2_}, {CpNi}) and IMe_4_ (= 1,2,3,4‐tetramethylimidazol‐5‐ylidene), on the *ω*B97X‐D3BJ/def2‐TZVP level of theory was performed. Initially, the frontier molecular orbitals (MOs) of the starting materials **D1_M_** were inspected. Both **D1_Ni_** as well as **D1_Mo_** reveal low laying unoccupied MOs with significant contributions from the P2 and P4 atoms (LUMO of **D1_Ni_** and LUMO+1 of **D1_Mo_**, Figure 3). These display optimal points for attack by the carbene nucleophiles, thus affording [{CpMo(CO)_2_}(η^3^‐IMe_4_P‐P_2_‐PMe_2_)]^+^ (**D3_Mo_**) and [{CpNi}(η^2:1^‐IMe_4_P_3_‐PMe_2_)]^+^ (**D4_Ni_**), respectively. The frontier MOs of these two species (see ESI) reveal potential for further nucleophilic functionalization but are comparably delocalized across the whole molecules. However, NBO analysis showed a much clearer picture and revealed that the σ*(P2‐P4) orbital in **D4_Ni_** and the σ*(Mo‐P3) orbital in **D3_Mo_** correspond well to low laying unoccupied MOs (Figure S77). Both NBOs display optimal points for nucleophilic functionalization accompanied by cleavage of the respective P2─P4 or the Mo─P3 bonds.

**Figure 4:**
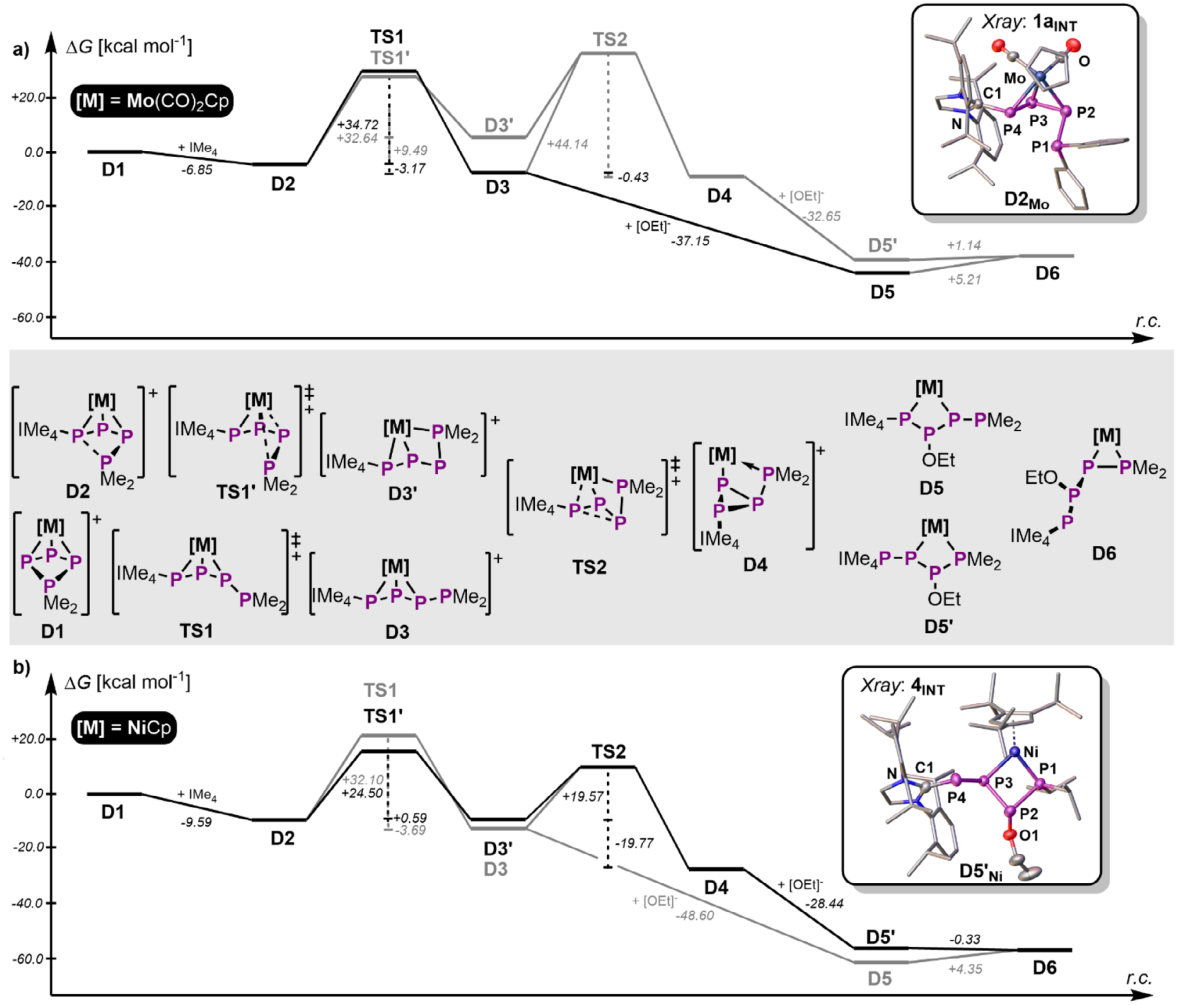
a,b) Computed reaction mechanisms for the addition of NHC to **D1_Mo_** and **D1_Ni_** as well as the reaction pathway (stippled connection indicates that potential transition states were not computed) of EtO^−^ addition to **D3_Mo_** and **D4_Ni_**, respectively; lowest energy pathway is indicated in black, while the energetically unfavorable structures (realized by the corresponding other TM unit) are shown in gray for comparison; *ω*B97X‐D3BJ/def2‐TZVP level of theory.

**Figure 5:**
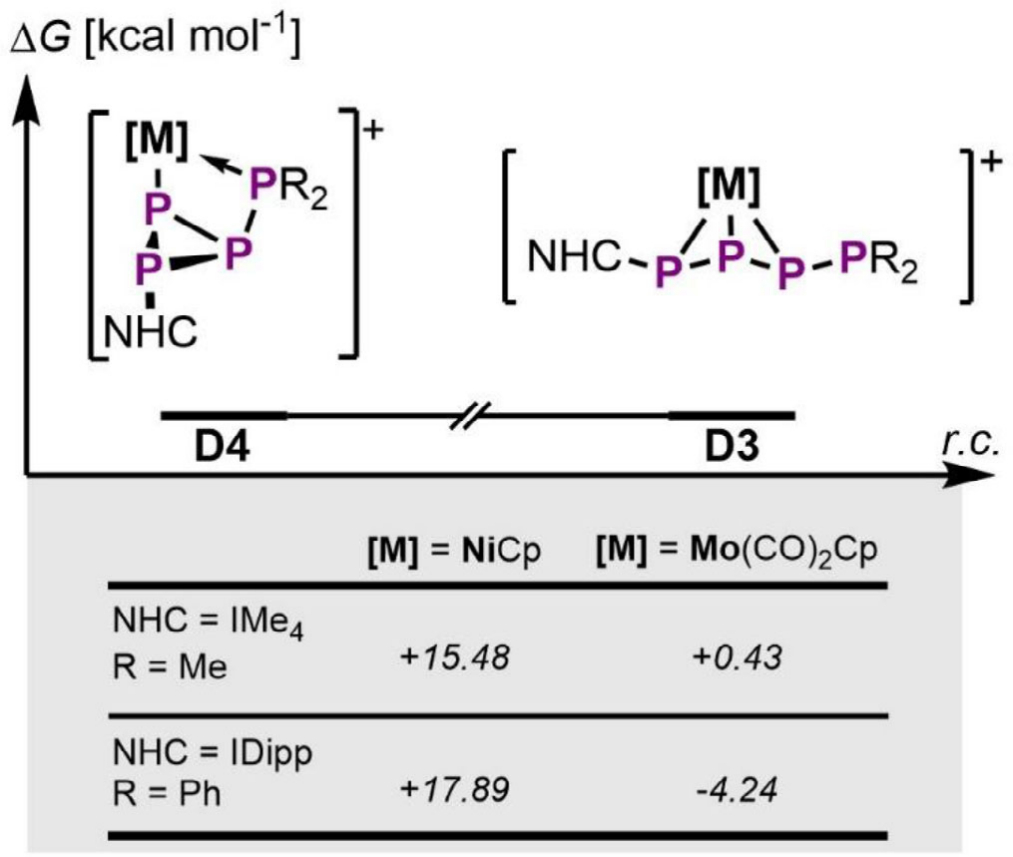
Energetic comparison of **D4** and **D3** depending on the steric influence of the substituents and the NHC; *ω*B97X‐D3BJ/def2‐TZVP level of theory.

**Scheme 3:**
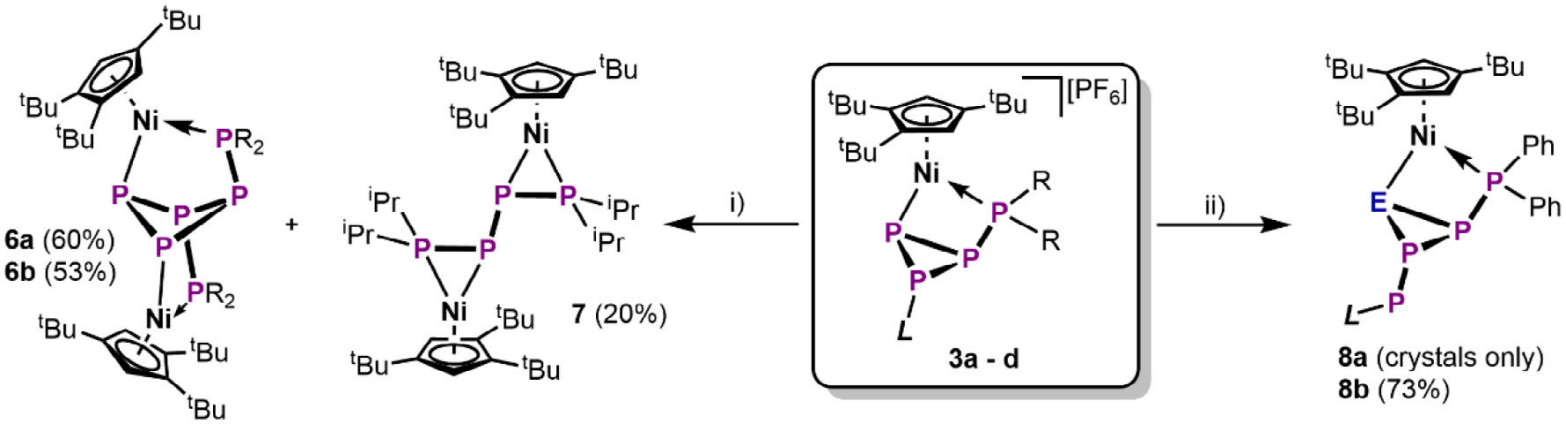
Synthesis of **6**, **7**, and **8**; i) [Et_4_N][CN], THF, 70 °C, 3 h; ii) M[ECO] (M = Na, E = P or M = K, E = As), THF, –80 °C – r.t., 2 h.

Furthermore, computation of the reaction mechanism, including transition states and potential intermediates should deliver an even clearer understanding of the observed reactivity. The initial addition of IMe_4_ to **D1_M_** (Figure 4a,b) is exothermic in both cases and occurs without a transition state (TS) leading to the intermediate [{L_n_M}(η^3^‐IMe_4_P_4_Me_2_)]^+^ (**D2_M_**). This intermediate shows the *cyclo*‐P_4_R_2_ ligand being partially opened and the P1─P4 bond being nearly broken. The corresponding intermediates can even be observed experimentally in the ^31^P NMR spectra of the crude reaction solutions of **1** and **3** at ‐80 °C (Figures S36 and S52). **1a_INT_** (Figure 4: inlet **D2_Mo_**) could even be crystallized revealing the partially ring‐opened ligand resulting from the NHC addition to P4. The latter finds its explanation in the low laying unoccupied frontier molecular orbitals of **D1_M_** (*vide supra*). As experimentally observed, **D2_M_** is not stable at room temperature for either Mo or Ni and thus rearranges. For **D2_Mo_** this occurs via **TS1**, corresponding to a rotation of the phosphino‐group away from the Mo center. This directly leads to the formation of product **D3_Mo_**, which overall is exergonic by 10.0 kcal mol^−1^ with respect to the starting materials. In contrast, the Ni center in **D2_Ni_** allows for a different course of reaction, involving coordination of the phosphino‐substituent within **TS1’**. The latter is much more favorable than **TS1** for Ni (7.6 kcal mol^−1^), while both TS are similar in energy in the case of Mo (2.1 kcal mol^−1^). From **TS1’** the intermediate **D3’_Ni_** is formed. **D3’_Ni_** reveals a catena *L*–P_3_–PMe_2_ ligand coordinated to the Ni center via the phosphino group as well as one of the P─P bonds (Figure 4). Although its formation is slightly endergonic (0.6 kcal mol^−1^) this allows for ring closure to take place via **TS2** (19.6 kcal mol^−1^), overall displaying the lowest energy pathway for the Ni system. Finally, this affords the housane‐type **D4_Ni_** derivative which is highly favored compared to the hypothetical **D3_Ni_** (15.5 kcal mol^−1^). The latter would be isostructural to the Mo system **D3_Mo_** bearing a chain‐type L–P_3_–PMe_2_ ligand. The model system suggests a similar energetic trend for the Mo system. Thus, **D4_Mo_** should also be energetically favorable compared to **D3_Mo_** (+0.4 kcal mol^−1^). However, inclusion of the experimentally relevant substituents (IDipp and Ph) corrects this issue and stabilizes **D3_Mo_** by 4.2 kcal mol^−1^ against **D4_Mo_** (Figure 5). In contrast, inclusion of ligand sterics into the Ni system even further destabilizes **D3_Ni_** against **D4_Ni_**_._ Additionally, the formation of **D4_Mo_** is kinetically inaccessible, as the corresponding **TS2** would be endergonic by 44.1 kcal mol^−1^ (from **D3_Mo_**) and the hypothetical **D3’_Mo_** is disfavored by 12.7 kcal mol^−1^. As **D3_Mo_** and **D4_Ni_** hold a positive charge they should be suitable for further functionalization. The distinct low‐laying unoccupied MOs, which correspond well with the NBOs of one σ*(P─Mo) (**D3_Mo_**) or a σ*(P─P) (**D4_Ni_**) bond, display prime points for nucleophilic functionalization (Figure 3, *vide supra*). Accordingly, addition of the prototypical EtO^–^ to **D3_Mo_** is exergonic (37.2 kcal mol^−1^) and affords the addition product [{CpMo(CO)_2_}(η^1:1^‐IMe_4_PP(OEt)P‐PMe_2_)] (**D5_Mo_**) with the expected regio‐isomer of the *L*–PP(OEt)P–PR_2_ ligand (compare **2**). Unfortunately, a TS for this reaction could not be located and thus the determination of an energetic barrier was unsuccessful. In contrast, addition of EtO^–^ to **D4_Ni_** initially leads to the formation of **D5’_Ni_** with an *L*–PPP(OEt)–PR_2_ ligand, which is in line with an orbital controlled reaction mechanism (see Figure 3). This intermediate [Cp'''Ni(η^2^‐IDippPPP(OEt)P*^i^* Pr_2_)] (**4_INT_**, corresponding to **D5’_Ni_** in the model system) could be experimentally observed in the ^31^P NMR spectrum at ‐80 °C (Figure S64) and is responsible for the color change during the formation of **4**. As **4_INT_** is stable up to ‐20 °C it was possible to grow turquoise single crystals of this compound and thus confirm its molecular structure in the solid state (Figure 2: inlet **D5’_Ni_**). Nevertheless, the rearrangement of **D5’_Ni_** to [{CpNi}(η^1:1^‐IMe_4_PP(OEt)P‐PMe_2_)] (**D6_Ni_**) is slightly exergonic (0.33 kcal mol^−1^), which is in line with the formation of **4** under experimental conditions. Again, a TS for this transformation could not be located due to the complexity of the system. Although the final *L*–PP(OEt)P–PR_2_ ligands in **D5_Mo_** and **D6_Ni_** are comparable, they coordinate the two TM units in two distinct ways. This may be influenced by the ligand sterics but most notably is attributed to the size difference between the respective TM (covalent single bond radii: 1.10 Å (Ni), 1.38 Å (Mo)) (scheme 3).^[^ ^47^ ^]^

### 2.3 Fragmentation and Expansion the *cyclo*‐P_4_R_2_ Complex **3**

**Figure 6:**
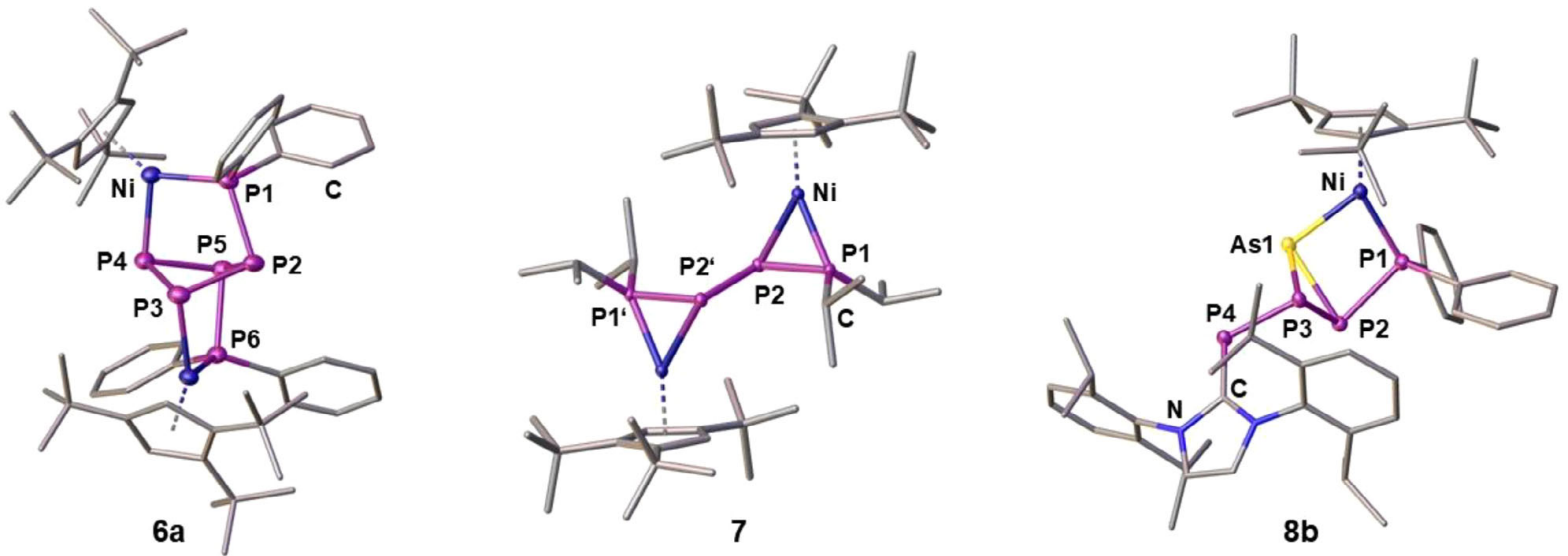
Crystal structures of **6a**, **7**, and **8b** (ellipsoids are drawn at the 50% probability level, anions and H atoms are omitted for clarity).

Lastly, the comparably simple and high‐yielding access to **1** and **3** inspired further investigations towards their nucleophilic functionalization beyond prototypical EtO^–^ as educt. However, the {CpMo(CO)_2_} fragment proved to be insufficient for this reactivity, affording inseparable product mixtures. In contrast, when **3a** – **d** are reacted with [Et_4_N][CN], formation of a comparably symmetrical product with three chemically inequivalent P environments is indicated by AA'MM'XX’ spin systems in the ^31^P NMR spectra (Figures S68 and S70). Additionally, a singlet at *δ*/ppm = –120.3 marks the formation of IDippP–CN (Figure S71),^[^ ^49^ ^]^ suggesting fragmentation of the P_4_ ligand in **3** and dimerization of the resulting P_3_ building block. Similar [3+1]‐fragmentation has recently been observed in the cyanolysis of an acylated *cyclo*‐P_4_ complex of Co.^[^ ^50^ ^]^ The cage‐type products [{Cp'''Ni}_2_(μ,η^1:1:1:1^‐*cyclo*‐P_4_(PR_2_)_2_)] (**6a**: R = Ph, **6b**: R  =  *^i^* Pr) can be isolated in good yields of 60% and 53%, respectively, after column chromatographic workup (Scheme 3). Additionally, the side‐product [{Cp'''Ni}_2_(μ,η^2:2^‐(PP*^i^* Pr_2_)_2_)] (**7**), showing further fragmentation, could be isolated from reactions involving **3b**/**d**. This reactivity seems to be governed by the same σ*(P–P) MO in **3** (Figure 3, **D4_Ni_**) as the reaction with EtO^–^. Instead of addition to P2, however, CN^–^ attacks at P4, which is followed by twofold P–P bond cleavage and dimerization of the hypothetical intermediate [Cp'''Ni(η^1:1^‐P_3_R_2_)] (Figure S79). The solid‐state structure of **6a** (Figure 6) reveals its *C*_2_ symmetric, butterfly shaped *cyclo*‐P_4_‐1,2‐(PPh_2_)_2_ ligand, which coordinates the Ni centers in a μ,η^1:1:1:1^‐mode. While the P─P bonds in **6a** (2.215(2) – 2.245(1) Å) are in the range of single bonds, the P–P bonds in **7** (Figure S18) are alternating in length (P1–P2: 2.128(1) Å, P2–P2’: 2.244(1) Å) indicating partial double bond character for the μ,η^2:2^‐*^i^* Pr_2_PP‐PP*^i^* Pr_2_ ligand.^[^ ^47^ ^]^ Lastly, **3a** was reacted with salts of the [ECO]^–^ anion (E = P, As) in the hopes of expanding the P_4_ ligand to a EP_4_ scaffold. Both reactions afforded the desired product [Cp'''Ni(η^1:1^‐EP_4_Ph_2_IDipp)] (**8a**: E = P, **8b**: E = As) under release of CO. Full conversion to the respective product was observed in both cases by ^31^P NMR spectroscopy. However, separation of **8a** from side products is possible only in amounts of a few single crystals, which were obtained from a vapor phase diffusion crystallization (see ESI). In contrast, **8b** can easily be purified by crystallization from MeCN/Et_2_O mixtures and isolated in 73% yield. Both compounds are isostructural and show a housane‐type scaffold, similar to **3a** – **d**, with the major difference being the replacement of the imidazolyl substituent by a –PIDipp unit. The As atom in **8b** is located at the position of the former P3 (in **3**) with only slight disorder across the central AsP_2_ ring (Figure 3b). Again, this suggests MO controlled addition of the [ECO]^–^ anions to P3, followed by CO release and rearrangement of the resulting EP_4_ scaffold. Notably, the As─P bond lengths (2.306(5) – 2.361(7) Å) in **8b** are in the range of single bonds as are the P─P bond lengths (2.184(6) – 2.266(1) Å) in **8a** and **8b**. Finally, the AMQX spin system in the ^31^P NMR spectrum of **8b** corroborates the structure of the main isomer in solution (see Figure S75).

## 3 Conclusion

In summary, this study compares the reactivity of three *cyclo*‐P_4_R_2_ complexes **A**, **B** and **C** bearing isolobal or iso‐VE TM units. The respective TM units are demonstrated to impact the reactivity of the corresponding complex. While the neutral **C** does not show any reactivity towards nucleophilic carbenes, the cationic complexes **A** and **B** undergo addition reactions with IDipp and I*^i^* Pr_2_Me_2_, respectively. However, the respective products **1a** – **d** and **3a** – **d** show different geometries for the *L*‐P_3_PR_2_ ligands, solely dependent on the TM unit. In a second functionalization step, the reactions of **1** and **3** with EtO^–^ result in a simple addition or a complex addition, ring‐opening, rearrangement sequence, respectively. Although the *L*–PP(OEt)P–PR_2_ ligand is similar in both **2** and **4**, its coordinating atoms and binding mode differ (η^1:1^ versus η^2^). Proving the synthetic value of these findings, the methylation of **4** affords complex **5**, bearing an *iso*‐tetraphosphine ligand. Notably, this marks the first incidence of complete functionalization of a P_n_ ligand to a complex phosphine. Additionally, the synthetic utility of **3** is exploited to access the unprecedented polyphosphorus compounds **6** – **8**. Lastly, a combination of experimental and computational studies elucidates the underlying reaction mechanism leading to the formation of **1** – **4**. In summary, the size of the used TM, as well as the charge of the respective complex, govern the reactivity of the coordinated polyphosphorus ligand. So far, such effects have found little to no attention in phosphorus and main group chemistry, especially where coordination to a TM is involved. This study exploits this distinct influence of isolobal TM units to stabilize novel polyphosphorus species and advance the TM mediated transformation of P_4_.

## Supporting Information

The authors have cited additional references within the Supporting Information.^[^ ^13^, ^14^, ^15^, ^44^, ^46^, ^51^, ^52^, ^53^, ^54^, ^55^, ^56^, ^57^, ^58^, ^59^, ^60^, ^61^, ^62^, ^63^, ^64^, ^65^, ^66^, ^67^, ^68^, ^69^, ^70^, ^71^, ^72^, ^73^, ^74^, ^75^, ^76^, ^77^ ^]^ Deposition Numbers CCDC‐2421953‐2421971 contain the supplementary crystallographic data for this paper. These data are provided free of charge by the joint Cambridge Crystallographic Data Centre (https://www.ccdc.cam.ac.uk/services/structures?id = https://doi.org/10.1002/chem.202402675) and Fachinformationszentrum Karlsruhe (http://www.ccdc.cam.ac.uk/structures).

## Conflict of Interests

The authors declare no conflict of interest.

## Supporting information



Supporting Information

## Data Availability

The data that support the findings of this study are available in the supplementary material of this article.

## References

[1] B. M. Cossairt , N. A. Piro , C. C. Cummins , Chem. Rev. 2010, 110, 4164. 20175534 10.1021/cr9003709

[2] M. Caporali , L. Gonsalvi , A. Rossin , M. Peruzzini , Chem. Rev. 2010, 110, 4178. 20170154 10.1021/cr900349u

[3] C. M. Hoidn , D. J. Scott , R. Wolf , Chem.‐Eur. J. 2021, 27, 1886. 33135828 10.1002/chem.202001854 PMC7894350

[4] U. Lennert , P. B. Arockiam , V. Streitferdt , D. J. Scott , C. Rödl , R. M. Gschwind , R. Wolf , Nat. Catal. 2019, 2, 1101. 31844839 10.1038/s41929-019-0378-4 PMC6914361

[5] D. J. Scott , J. Cammarata , M. Schimpf , R. Wolf , Nat. Chem. 2021, 13, 458. 33820964 10.1038/s41557-021-00657-7

[6] M. Donath , K. Schwedtmann , T. Schneider , F. Hennersdorf , A. Bauzá , A. Frontera , J. J. Weigand , Nat. Chem. 2022, 14, 384. 35379968 10.1038/s41557-022-00913-4

[7] J. Cammarata , F. F. Westermair , P. Coburger , D. Duvinage , M. Janssen , M. K. Uttendorfer , J. Beckmann , R. M. Gschwind , R. Wolf , D. J. Scott , Angew. Chem., Int. Ed. 2024, 63, 202408423. 10.1002/anie.202408423 38946592

[8] R. Hoffmann , Angew. Chem. Int. Ed. Engl. 1982, 21, 711.

[9] F. Mathey , Acc. Chem. Res. 1992, 25, 90.

[10] F. Mathey , Angew. Chem., Int. Ed. 2003, 42, 1578. 10.1002/anie.200200557 12698454

[11] F. Meurer , F. Kleemiss , C. Riesinger , G. Balázs , V. Vuković , I. G. Shenderovich , C. Jelsch , M. Bodensteiner , Chem.‐Eur. J. 2024, 30, 202303762. 10.1002/chem.202303762 38277228

[12] F. G. A. Stone , Angew. Chem., Int. Ed. Engl. 1984, 23, 89.

[13] O. J. Scherer , H. Sitzmann , G. Wolmershäuser , J. Organomet. Chem. 1984, 268, C9.

[14] E. Mädl , G. Balázs , E. V. Peresypkina , M. Scheer , Angew. Chem. Int. Ed. 2016, 55, 7702. 10.1002/anie.201601775 27097602

[15] C. Riesinger , L. Dütsch , G. Balázs , M. Bodensteiner , M. Scheer , Chem.‐Eur. J. 2020, 26, 17165. 32996637 10.1002/chem.202003291 PMC7839539

[16] O. J. Scherer , J. Vondung , G. Wolmershäuser , Angew. Chem. Int. Ed. Engl. 1989, 28, 1355.

[17] O. J. Scherer , R. Winter , G. Wolmershäuser , Z. Anorg. Allg. Chem. 1993, 619, 827.

[18] M. Herberhold , G. Frohmader , W. Milius , J. Organomet. Chem. 1996, 522, 185.

[19] F. Dielmann , A. Timoshkin , M. Piesch , G. Balázs , M. Scheer , Angew. Chem., Int. Ed. 2017, 56, 1671. 10.1002/anie.201610967 PMC5299486 28078794

[20] A. Cavaillé , N. Saffon‐Merceron , N. Nebra , M. Fustier‐Boutignon , N. Mézailles , Angew. Chem., Int. Ed. 2018, 57, 1874. 10.1002/anie.201711130 29243885

[21] K. A. Mandla , M. L. Neville , C. E. Moore , A. L. Rheingold , J. S. Figueroa , Angew. Chem., Int. Ed. 2019, 58, 15329. 10.1002/anie.201908885 31418523

[22] O. J. Scherer , T. Brück , Angew. Chem. Int. Ed. Engl. 1987, 26, 59.

[23] M. Baudler , T. Etzbach , Angew. Chem. Int. Ed. Engl. 1991, 30, 580.

[24] C. M. Knapp , B. H. Westcott , M. A. C. Raybould , J. E. McGrady , J. M. Goicoechea , Angew. Chem. Int. Ed. 2012, 51, 9097. 10.1002/anie.201203980 22847864

[25] O. J. Scherer , H. Sitzmann , G. Wolmershäuser , Angew. Chem. Int. Ed. Engl. 1985, 24, 351.

[26] O. J. Scherer , H. Swarowsky , G. Wolmershäuser , W. Kaim , S. Kohlmann , Angew. Chem. Int. Ed. Engl. 1987, 26, 1153.

[27] O. J. Scherer , J. Schwalb , H. Swarowsky , G. Wolmershäuser , W. Kaim , R. Gross , Chem. Ber. 1988, 121, 443.

[28] C. Riesinger , F. Dielmann , R. Szlosek , A. V. Virovets , M. Scheer , Angew. Chem., Int. Ed. 2023, 62, 202218828. 10.1002/anie.202218828 36692270

[29] O. J. Scherer , Angew. Chem., Int. Ed. Engl. 1990, 29, 1104.

[30] C. E. Laplaza , W. M. Davis , C. C. Cummins , Angew. Chem., Int. Ed. Engl. 1995, 34, 2042.

[31] G. Hierlmeier , A. Hinz , R. Wolf , J. M. Goicoechea , Angew. Chem., Int. Ed. 2018, 57, 431. 10.1002/anie.201710582 29152826

[32] M. Piesch , F. Dielmann , S. Reichl , M. Scheer , Chem.‐Eur. J. 2020, 26, 1518. 31860738 10.1002/chem.201905240 PMC7028134

[33] J. Sun , H. Verplancke , J. I. Schweizer , M. Diefenbach , C. Würtele , M. Otte , I. Tkach , C. Herwig , C. Limberg , S. Demeshko , M. C. Holthausen , S. Schneider , Chem 2021, 7, 1952.

[34] T. G. Saint‐Denis , T. A. Wheeler , Q. Chen , G. Balázs , N. S. Settineri , M. Scheer , T. D. Tilley , J. Am. Chem. Soc. 2024, 146, 4369. 38335065 10.1021/jacs.3c14779 PMC10885142

[35] B. P. Johnson , G. Balázs , M. Scheer , Coord. Chem. Rev. 2006, 250, 1178.

[36] S. Wang , J. D. Sears , C. E. Moore , A. L. Rheingold , M. L. Neidig , J. S. Figueroa , Science 2022, 375, 1393. 35324298 10.1126/science.abn7100 PMC9210196

[37] A. K. Adhikari , C. G. P. Ziegler , K. Schwedtmann , C. Taube , J. J. Weigand , R. Wolf , Angew. Chem., Int. Ed. 2019, 58, 18584. 10.1002/anie.201908998 PMC6916545 31559678

[38] C. Riesinger , A. Erhard , M. Scheer , Chem. Commun. 2023, 59, 10117. 10.1039/d3cc03369d 37530455

[39] S. Reichl , E. Mädl , F. Riedlberger , M. Piesch , G. Balázs , M. Seidl , M. Scheer , Nat. Commun. 2021, 12, 5774. 34599185 10.1038/s41467-021-26002-7 PMC8486752

[40] S. Reichl , G. Balázs , M. Scheer , Chem. Sci. 2023, 14, 3834. 37035692 10.1039/d3sc00580a PMC10074431

[41] C. M. Hoidn , K. Trabitsch , K. Schwedtmann , C. Taube , J. J. Weigand , R. Wolf , Chem.‐Eur. J. 2023, 29, 202301930. 10.1002/chem.202301930 37489883

[42] K. Trabitsch , S. Hauer , K. Schwedtmann , P. Royla , J. J. Weigand , R. Wolf , Inorg. Chem. Front. 2025, 12, 2013.

[43] C. G. P. Ziegler , T. M. Maier , S. Pelties , C. Taube , F. Hennersdorf , A. W. Ehlers , J. J. Weigand , R. Wolf , Chem. Sci. 2019, 10, 1302. 30809344 10.1039/c8sc04745f PMC6357856

[44] M. Piesch , S. Reichl , M. Seidl , G. Balázs , M. Scheer , Angew. Chem. Int. Ed. 2021, 60, 15101. 10.1002/anie.202103683 PMC8251822 33961722

[45] J. J. Weigand , M. Holthausen , R. Fröhlich , Angew. Chem., Int. Ed. 2009, 48, 295. 10.1002/anie.200804903 19065549

[46] A. R. Jupp , J. M. Goicoechea , Angew. Chem., Int. Ed. 2013, 52, 10064. 10.1002/anie.201305235 23913436

[47] P. Pyykkö , J. Phys. Chem. A 2015, 119, 2326. 25162610 10.1021/jp5065819

[48] R. Criegee , A. Rimmelin , Chem. Ber. 1957, 90, 414.

[49] Z. Li , J. E. Borger , F. Müller , J. R. Harmer , C.‐Y. Su , H. Grützmacher , Angew. Chem. Int. Ed. 2019, 58, 11429. 10.1002/anie.201904720 31157494

[50] S. Hauer , T. M. Horsley Downie , G. Balázs , K. Schwedtmann , J. J. Weigand , R. Wolf , Angew. Chem., Int. Ed. 2024, 63, e202317170. 10.1002/anie.202317170 38059391

[51] omics.pnl.gov/software/molecular‐weight‐calculator 2024.

[52] A. J. Arduengo , R. Krafczyk , R. Schmutzler , Tetrahedron 1999, 55, 14523.

[53] N. Kuhn , T. Kratz , Synth. 1993, 1993, 561.

[54] M. Gonsior , I. Krossing , N. Mitzel , Z. Anorg. Allg. Chem. 2002, 628, 1821.

[55] F. F. Puschmann , D. Stein , D. Heift , C. Hendriksen , Z. A. Gal , H.‐F. Grützmacher , H. Grützmacher , Angew. Chem., Int. Ed. 2011, 50, 8420. 10.1002/anie.201102930 21766405

[56] A. Hinz , J. M. Goicoechea , Angew. Chem., Int. Ed. 2016, 55, 15515. 10.1002/anie.201609309 PMC5299489 27862768

[57] S. Yao , Y. Grossheim , A. Kostenko , E. Ballestero‐Martínez , S. Schutte , M. Bispinghoff , H. Grützmacher , M. Driess , Angew. Chem., Int. Ed. 2017, 56, 7465. 10.1002/anie.201703731 28464389

[58] Agilent , CrysAlisPro, Agilent Technologies Ltd, Yarnton, Oxfordshire, England 2014.

[59] G. M. Sheldrick , Acta Cryst. A. 2015, 71, 3.

[60] G. M. Sheldrick , Acta Cryst. C 2015, 71, 3.

[61] O. V. Dolomanov , L. J. Bourhis , R. J. Gildea , J. A. K. Howard , H. Puschmann , J. Appl. Crystallogr. 2009, 42, 339. 10.1107/S0021889811041161 PMC3236671 22199401

[62] F. Neese , WIREs Comput. Mol. Sci. 2012, 2, 73.

[63] F. Neese , WIREs Comput. Mol. Sci. 2018, 8, 1327.

[64] F. Neese , F. Wennmohs , U. Becker , C. Riplinger , J. Chem. Phys. 2020, 152, 224108. 32534543 10.1063/5.0004608

[65] F. Neese , WIREs Comput. Mol. Sci. 2022, 12, 1606.

[66] F. Neese , J. Comput. Chem. 2023, 44, 381. 35678278 10.1002/jcc.26942

[67] Chemcraft – graphical software for visualization of quantum chemistry computations, https://www.chemcraftprog.com (accessed: 2024).

[68] D. Andrae , U. Huermann , M. Dolg , H. Stoll , H. Preu , Theoret. Chim. Acta 1990, 77, 123.

[69] V. Barone , M. Cossi , J. Phys. Chem. A 1998, 102, 1995.

[70] F. Weigend , R. Ahlrichs , Phys. Chem. Chem. Phys. 2005, 7, 3297. 16240044 10.1039/b508541a

[71] F. Weigend , Phys. Chem. Chem. Phys. 2006, 8, 1057. 16633586 10.1039/b515623h

[72] J.‐D. Chai , M. Head‐Gordon , Phys. Chem. Chem. Phys. 2008, 10, 6615. 18989472 10.1039/b810189b

[73] S. Grimme , S. Ehrlich , L. Goerigk , J. Comput. Chem. 2011, 32, 1456. 21370243 10.1002/jcc.21759

[74] Y.‐S. Lin , G.‐D. Li , S.‐P. Mao , J.‐D. Chai , J. Chem. Theory Comput. 2013, 9, 263. 26589028 10.1021/ct300715s

[75] E. D. Glendening , C. R. Landis , F. Weinhold , J. Comput. Chem. 2019, 40, 2234. 31172571 10.1002/jcc.25873

[76] A. E. Reed , L. A. Curtiss , F. Weinhold , Chem. Rev. 1988, 88, 899.

[77] V. Ásgeirsson , B. O. Birgisson , R. Bjornsson , U. Becker , F. Neese , C. Riplinger , H. Jónsson , J. Chem. Theory Comput. 2021, 17, 4929. 34275279 10.1021/acs.jctc.1c00462

