# Supplementary material for: Functionalization, Fragmentation, and Expansion of cyclo‐P4R2 Ligands
Source: Chemistry. 2025 May 24;31(36):e202501305. doi: 10.1002/chem.202501305 (PMC12202848; doi:10.1002/chem.202501305)
Supplement: Supplementary file 1 — Supporting Information [file CHEM-31-e202501305-s001.pdf]

Supporting Information

Functionalization, Fragmentation and Expansion of *cyclo*-P_4R_2 Ligands

Christoph Riesinger,† Lisa Zimmermann,† Robert Szlosek, Jan Wieneke, Lisa-Marie Orel, Gábor Balázs, Luis Dütsch, Manfred Scheer*

Author Contributions

Christoph Riesinger – Conceptualization, Synthesis of **B1**, **B2**, **3a-d**, **4** and **4**$_{\mathbf{INT}}$, **5**, **6a/b**, **7**, **8a/b** Interpretation of crystallographic data, Writing of original draft

Lisa Zimmermann – Synthesis of **A1**, **A2**, **1a-d** and **2**, Variable Temperature NMR spectroscopy for the Detection of **1a**$_{\mathbf{INT}}$ and **3a**$_{\mathbf{INT}}$, Reaction screening with **C**, Writing of original draft.

Jan Thomas Wieneke – Assistance in compound synthesis.

Lisa-Marie Orel – Assistance in compound synthesis.

Robert Szlosek, Gábor Balázs – Performance and interpretation of DFT computations.

Luis Dütsch – Initial reactivity studies of **A** towards phosphenium cations.

Manfred Scheer – project administration, funding acquisition.

All authors contributed in preparing the final manuscript.

Contents

Synthesis and analytical data 3
General Considerations 3
Complex Synthesis 4
Crystallographic Details 24
General Considerations 24
NMR Spectra 51
Computational Details 75
General Considerations 75
Frontier Molecular Orbital Analysis 75
Thermochemistry of individual compounds 78

Synthesis and Analytical Data

General Considerations

All manipulations were carried out using standard Schlenk techniques at a Stock apparatus under N_2 as an inert gas or in a glove box with Ar atmosphere. All glassware was dried with a heat gun (600 °C) for at least 30 min prior to use. *o*-DFB (1,2-difluorobenzene) was distilled from P_2O_5, CD_2Cl_2 was distilled from CaH_2 and other solvents were directly taken from an MBraun SPS-800 solvent purification system, degassed at room temperature and stored over molecular sieves. Solution ^{1}H (400.130 MHz), ^{19}F (376.498 MHz), and ^{31}P (161.976 MHz) spectra were recorded at an Avance400 (Bruker) spectrometer using $(H_3C)_4Si$ (^{1}H, ^{13}C), $CFCl_3$ (^{19}F) or 85% phosphoric acid (^{31}P), respectively, as external standards. Chemical shifts (δ) are provided in parts per million (ppm) and coupling constants (J) are reported in Hertz (Hz). Chemical shifts and coupling constants for ^{31}P{^{1}H} and ^{31}P NMR spectra were partly derived from spectral simulation using the built-in simulation package of TopSpin3.2. The following abbreviations are used: s = singlet, d = doublet, dd = doublet of doublets, dt = doublet of triplets, t = triplet, td = triplet of doublets br = broad and m = multiplet. Mass spectra were recorded at the internal mass spectrometry department using a ThermoQuest Finnigan TSQ 7000 (ESI), Finnigan MAT 95 (LIFDI) mass spectrometer or by the first authors on a Waters Micromass LCT ESI-TOF mass-spectrometer and peak assignment was performed using the Molecular weight calculator 6.50.[1] IR spectra were recorded as solids using a ThermoFisher Nicolet iS5 FT-IR spectrometer with an iD7 ATR module and an ITX Germanium or ITX Diamond crystal. Elemental analysis of the products was conducted by the elemental analysis department at the University of Regensburg using an Elementar Vario EL. The starting materials [Cp'''Ni(η^3-P_3)][2], [CpMo(CO)$_2$(η^3-P_3)][3], IDipp[4], I^{i}Pr$_2$Me$_2$[5] and Tl[TEF][6] were synthesized according to literature procedures. The synthesis of [Cp'''Ni(η^3-P_4R_2)][PF_6] (R = Ph, ^{i}Pr) is adapted from literature procedures.[7] All other chemicals were purchased from commercial vendors and used without further purification.

Complex Synthesis

Synthesis of [CpMo(CO)$_2$(η^3-P$_4$Ph$_2$)][OTf] (**A1**)

[CpMo(CO)$_2$(η^3-P$_3$)] (1.00 g, 3.23 mmol, 1 eq.) and Tl[OTf] (1.14 g, 3.23 mmol, 1 eq.) were mixed and dissolved in 15 mL of *o*-DFB. Ph$_2$PCl (0.58 mL, 3.23 mmol, 1 eq.) was added slowly leading to a slow change of color to yellow and the precipitation of colorless solid. The mixture was stirred for 18 h at room temperature, concentrated and precipitated by addition of *n*-hexane (50 mL). The yellow precipitate was washed with *n*-hexane (5 mL) and dried under reduced pressure (10^{-3} mbar). Afterwards, the solid was dissolved in 50 mL of *o*-DFB, filtered through a glass fiber filter paper and dried under reduced pressure (10^{-3} mbar). Crystals of **A1** suitable for single crystal X-ray measurements were obtained by layering a concentrated solution of **A1** in *o*-DFB with *n*-hexane (30 mL).

**Yield**:	1.48 g (2.24 mmol, 69%)
**ESI(+)-MS** (*o*-DFB):	*m/z* (%) = 496.9 (100, [**A1**]$^+$), 468.9 (50, [**A1**-CO]$^+$), 440.9 (5, [**A1**-2CO]$^+$)
**Elemental analysis**:	Calculated (%) for C$_{20}$H$_{15}$F$_3$P$_4$SMo: C: 37.29, H: 2.35, S: 4.98; found: C: 37.67, H:2.52, S: 5.17.
**^{1}H-NMR** (CD$_2$Cl$_2$, 300 K):	δ/ ppm = 5.82 (s, 5 H, C$_5\underline{H}_5$), 7.42 – 7.99 (several multiplets, 10 H, Ph)
**^{31}P{^{1}H}-NMR** (CD$_2$Cl$_2$, 300 K):	δ/ ppm = 63.7 (m, $^1J_{PA-PM/M'}$ = 275/276 Hz, $^2J_{PA-PX}$ = 16 Hz, 1 P, P^A), 1.9 (m, $^1J_{PM/M'-PA}$ = 275/276 Hz, $^1J_{PM/M'-PX}$ = 265/266 Hz, $^2J_{PM-PM'}$ = -2 Hz, 2 P, P$^{M/M'}$), -70.6 (m, $^1J_{PX-PM/M'}$ = 265/266 Hz, $^2J_{PX-PA}$ = 16 Hz, 1 P, P^X)
**^{31}P-NMR** (CD$_2$Cl$_2$, 300 K):	δ/ ppm = 63.7 (m (br), significant broadening due to unresolved $^nJ_{P-H}$ coupling, 1 P, P^A), 1.9 (m, $^1J_{PM/M'-PA}$ = 275/276 Hz, $^1J_{PM/M'-PX}$ = 265/266 Hz, $^2J_{PM-PM'}$ = -2 Hz, 2 P, P$^{M/M'}$), -70.6 (m, $^1J_{PX-PM/M'}$ = 265/266 Hz, $^2J_{PX-PA}$ = 16 Hz, 1 P, P^X)
**^{19}F{^{1}H}-NMR** (CD$_2$Cl$_2$, 300 K):	δ/ ppm = -78.6 (s, 3 F, [OTf]$^-$)
**IR**:	$\tilde{\nu}$(CO)/cm^{-1} = 2042 (m), 2000 (m)

Synthesis of [CpMo(CO)$_2$(η^3-P$_4$^{i}Pr$_2$)][TEF] (**A2**)

[CpMo(CO)$_2$(η^3-P$_3$)] (1.00 g, 3.23 mmol, 1 eq.) and Tl[TEF] (3.78 g, 3.23 mmol, 1 eq.) were mixed and dissolved in 10 mL of *o*-DFB. ^{i}Pr$_2$PCl (0.52 mL, 3.23 mmol, 1 eq.) was added slowly leading to a slow change of color to yellow and the precipitation of colorless solid. The mixture was stirred for 3 h at room temperature, concentrated and precipitated by addition of *n*-hexane (50 mL). The yellow precipitate was washed with *n*-hexane (5 mL) and dried under reduced pressure (10^{-3} mbar). Afterwards, the solid was dissolved in 20 mL of *o*-DFB, filtered through a glass fiber filter paper and precipitated again by addition of *n*-hexane (30 mL). The solvent was decanted and the yellow powder was dried under reduced pressure (10^{-3} mbar). Crystals of **A2** suitable for single crystal X-ray measurements were obtained by layering a concentrated solution of **A2** in CH_2Cl_2 with *n*-hexane (30 mL).

**Yield**:	3.95 g (2.83 mmol, 87%)
**ESI(+)-MS** (*o*-DFB):	*m/z* (%) = 428.9 (100, [**A2**]$^+$), 400.9 (60, [**A2**-CO]$^+$)
**Elemental analysis**:	Calculated (%) for $C_{29}H_{19}O_6F_{36}P_4AlMo$: C: 24.98, H: 1.37; found: C: 25.07, H: 1.71.
**^{1}H-NMR** (CD_2Cl_2, 300 K):	δ/ ppm = 1.10 (d, 3 H, CH<u>Me$_2$</u>), 1.15 (d, 3 H, CH<u>Me$_2$</u>), 1.65 (d, 3 H, CH<u>Me$_2$</u>), 1.70 (d, 3 H, CH<u>Me$_2$</u>), 2.74 (m (overlapping), 2 H, C<u>H</u>Me$_2$), 5.82 (s, 5 H, C$_5$<u>H$_5$</u>)
**^{31}P{^{1}H}-NMR** (CD_2Cl_2, 300 K):	δ/ ppm = 109.3 (m, $^1J_{PA-PM/M'}$ = 267/268 Hz, $^2J_{PA-PX}$ = 16 Hz, 1 P, P^A), -20.5 (m, $^1J_{PM/M'-PA}$ = 267/268 Hz, $^1J_{PM/M'-PX}$ = 266/271 Hz, $^2J_{PM-PM'}$ = 4 Hz, 2 P, P$^{M/M'}$), -71.2 (m, $^1J_{PX-PM/M'}$ = 266/271 Hz, $^2J_{PX-PA}$ = 16 Hz, 1 P, P^X)
**^{31}P-NMR** (CD_2Cl_2, 300 K):	δ/ ppm = 109.3 (m (br), significant broadening due to unresolved $^nJ_{P-H}$ coupling, 1 P, P^A), -20.5 (m, $^1J_{PM/M'-PA}$ = 267/268 Hz, $^1J_{PM/M'-PX}$ = 266/271 Hz, $^2J_{PM-PM'}$ = 4 Hz, 2 P, P$^{M/M'}$), -71.2 (m, $^1J_{PX-PM/M'}$ = 266/271 Hz, $^2J_{PX-PA}$ = 16 Hz, 1 P, P^X)
**^{19}F{^{1}H}-NMR** (CD_2Cl_2, 300 K):	δ/ ppm = -75.6 (s, 36F, [TEF]$^-$)
**IR**:	$\tilde{\nu}$(CO)/cm^{-1} = 2058 (m), 2018 (m)

Synthesis of [Cp'''Ni(η^3-P_4Ph_2)][PF_6] (**B1**)

[Cp'''Ni(η^3-P_3)] (154 mg, 0.4 mmol, 1 eq.) and Tl[PF_6] (140 mg, 0.4 mmol, 1 eq.) were mixed and dissolved in 6 mL of *o*-DFB. Ph_2PCl (88mg, 73 µL, 0.4 mmol, 1 eq.) dissolved in 2 mL of toluene was added leading to a slow change of color to intense red and the precipitation of colorless solid. The mixture was stirred for 21 h and then left for the precipitate to settle. Afterwards, the solution was decanted and directly layered below 25 mL of *n*-hexane. Storage of this mixture for three days afforded **B1** as dark red crystalline blocks of X-ray quality.

Notably, **B1** has been prepared with several other counter anions. The procedure described here can be expanded to multigram scale.

**Yield**:	215 mg (75%)
**Elemental analysis**:	Calculated (%) for $C_{29}H_{39}F_6P_5Ni$: C: 48.70, H: 5.50; found: C:48.80, H: 5.19.
**^{1}H-NMR** (CD_2Cl_2, 300 K):	δ/ ppm = 1.20 (s, 9 H, $C_5H_2{}^t\underline{Bu_3}$), 1.31 (s, 18 H, $C_5H_2{}^t\underline{Bu_3}$), 5.92 (s, 2 H, $C_5\underline{H_2}{}^tBu_3$), 7.5 – 7.9 (several multiplets, 10 H, Ph)
**^{31}P{^{1}H}-NMR** (CD_2Cl_2, 300 K):	δ/ ppm = 73.5 (m, $^1J_{PA\text{-}PM/M'}$ = 293/281 Hz, $^2J_{PA\text{-}PX}$ = 14 Hz, 1 P, P^A), 57.7 (m, $^1J_{PM/M'\text{-}PA}$ = 293/281 Hz, $^1J_{PM/M'\text{-}PX}$ = 271/270 Hz, $^2J_{PM\text{-}PM'}$ = 2 Hz, 2 P, $P^{M/M'}$), 12.3 (m, $^1J_{PX\text{-}PM/M'}$ = 271/270 Hz, $^2J_{PX\text{-}PA}$ = 14 Hz, 1 P, P^X), -143.9 (sept, $^1J_{P\text{-}F}$ = 710 Hz, $[PF_6]^-$)
**^{31}P-NMR** (CD_2Cl_2, 300 K):	δ/ ppm = 73.5 (m, $^1J_{PA\text{-}PM/M'}$ = 293/281 Hz, $^2J_{PA\text{-}PX}$ = 14 Hz, 1 P, P^A), 57.7 (m, $^1J_{PM/M'\text{-}PA}$ = 293/281 Hz, $^1J_{PM/M'\text{-}PX}$ = 271/270 Hz, $^2J_{PM\text{-}PM'}$ = 2 Hz, 2 P, $P^{M/M'}$), 12.3 (m (br), significant broadening due to unresolved $^nJ_{P\text{-}H}$ coupling, 1 P, P^X), -143.9 (sept, $^1J_{P\text{-}F}$ = 710 Hz, $[PF_6]^-$)
**^{19}F{^{1}H}-NMR** (CD_2Cl_2, 300 K):	δ/ ppm = -73.1 (d, $^1J_{F\text{-}P}$ = 710 Hz, $[PF_6]^-$)

Synthesis of [Cp'''Ni(η^3-$P_4{}^i Pr_2$)][PF_6] (**B2**)

[Cp'''Ni(η^3-P_3)] (154 mg, 0.4 mmol, 1 eq.) and Tl[PF_6] (140 mg, 0.4 mmol, 1 eq.) were mixed and dissolved in 10 mL of *o*-DFB. ${}^iPr_2PCl$ (61mg, 63 µL, 0.4 mmol, 1 eq.) dissolved in 2 mL of *o*-DFB was added leading to a slow change of color to red and the precipitation of colorless solid. The mixture was stirred for 3 d and then 30 mL of *n*-hexane were added to precipitate a red solid. The supernatant was decanted, the solid dried and then 4 mL *o*-DFB were added. The red solution was filtered and then layered with 50 mL of *n*-hexane. This mixture was stored for 5 d at room temperature to afford **B2** as dark red crystals in X-ray quality. The procedure described here can be expanded to multigram scale.

**Yield**:	225 mg (88%)
**Elemental analysis**:	Calculated (%) for $C_{23}H_{43}F_6P_5Ni$: C: 42.69, H: 6.70; found: C: 43.14, H: 6.80.
**^{1}H-NMR** (CD_2Cl_2, 300 K):	δ/ ppm = 1.25 (d, 3 H, CH$\underline{Me_2}$), 1.27 (s, 9 H, $C_5H_2\underline{{}^tBu_3}$), 1.30 (d, 3 H, CH$\underline{Me_2}$), 1.39 (d, 3 H, CH$\underline{Me_2}$), 1.43 (s, 18 H, $C_5H_2\underline{{}^tBu_3}$), 1.44 (d, 3 H, CH$\underline{Me_2}$), 2.74 (m (overlapping), 2 H, 2x C$\underline{H}$Me$_2$), 5.97 (s, 2 H, $C_5\underline{H_2}{}^tBu_3$)
**^{31}P{^{1}H}-NMR** (CD_2Cl_2, 300 K):	δ/ ppm = 81.0 (m, $^1J_{PA\text{-}PX/X'}$ = 293/286 Hz, $^2J_{PA\text{-}PM}$ = 16 Hz, 1 P, P^A), 58.6 (m, $^1J_{PM\text{-}PX/X'}$ = 264/262 Hz, $^2J_{PM\text{-}PA}$ = 16 Hz, 1 P, P^M), 34.5 (m, $^1J_{PX\text{-}PA}$ = 293/286 Hz, $^1J_{PX\text{-}PM}$ = 264/262 Hz, $^2J_{PX\text{-}PX'}$ = 3 Hz, 2 P, $P^{X/X'}$), -143.9 (sept, $^1J_{P\text{-}F}$ = 710 Hz, $[PF_6]^-$)
**^{31}P-NMR** (CD_2Cl_2, 300 K):	δ/ ppm = 81.0 (m, $^1J_{PA\text{-}PX/X'}$ = 293/286 Hz, $^2J_{PA\text{-}PM}$ = 16 Hz, 1 P, P^A), 58.6 (m (br), significant broadening due to unresolved $^nJ_{P\text{-}H}$ coupling, 1 P, P^M), 34.5 (m, $^1J_{PX\text{-}PA}$ = 293/286 Hz, $^1J_{PX\text{-}PM}$ = 264/262 Hz, $^2J_{PX\text{-}PX'}$ = 3 Hz, 2 P, $P^{X/X'}$), -143.9 (sept, $^1J_{P\text{-}F}$ = 710 Hz, $[PF_6]^-$)
**^{19}F{^{1}H}-NMR** (CD_2Cl_2, 300 K):	δ/ ppm = -73.1 (d, $^1J_{F\text{-}P}$ = 710 Hz, $[PF_6]^-$)

Synthesis of [CpMo(CO)$_2$(η^3-P$_4$Ph$_2$IDipp)][OTf] (**1a**)

A1 (570 mg, 0.88 mmol, 1 eq.) was dissolved in 15 mL of *o*-DFB and IDipp (341 mg, 0.88 mmol, 1 eq.) dissolved in 5 mL of *o*-DFB was added at room temperature to afford an immediate change of color from yellow to orange. The solution was stirred for 16 h. Afterwards, the solution was constrained to 3 mL and 50 mL of *n*-pentane were added to precipitate [CpMo(CO)$_2$(η^3-P$_4$Ph$_2$IDipp)][OTf] (**1a**) as an orange powder. The supernatant was decanted and the solid washed with 15 mL of *n*-pentane, each. Drying under reduced pressure afforded **1a** as an orange solid, which could be recrystallized from concentrated *o*-DFB solutions layered with *n*-hexane and storage at room temperature.

Notably, the intermediate **1a$_{INT}$** could not be isolated as a pure compound due to its rapid degradation above 0 °C. However, it was possible to study this highly sensitive species by means of low temperature NMR spectroscopy (*vide infra*). Furthermore, gas phase diffusion at -30 °C even allowed for the growth of a single crystal of **1a$_{INT}$** and thus its crystallographic study.

**Yield**:	0.81 g (0.78 mmol, 89%)
**ESI(+)-MS** (*o*-DFB):	*m/z* (%) = 883.2 (100, [**1a**]$^+$), 855.2 (5, [**1a**-CO]$^+$), 389.3 (80, [IDippH]$^+$)
**Elemental analysis**:	Calculated (%) for $C_{47}H_{51}MoP_4F_3N_2O_5S\cdot(C_6H_4F_2)$: C:55.50, H:4.83, N: 2.44, S: 2.80; found: C: 55.78, H:4.82, N: 2.84, S: 3.20
**^{1}H-NMR** (CD$_2$Cl$_2$, 300 K):	δ/ ppm = 1.23 (d, 6 H, CH<u>Me$_2$</u>), 1.29 (d, 6 H, CH<u>Me$_2$</u>), 1.37 (d, 6 H, CH<u>Me$_2$</u>), 1.44 (d, 6 H, CH<u>Me$_2$</u>), 2.56 (m (*br*), 4 H, C<u>H</u>Me$_2$), 4.56 (s, 5 H, C$_5$<u>H$_5$</u>), 7.28 – 7.63 (several overlapping multiplets, 16 H, Ph and Dipp groups), 7.65 (s, 2 H, (<u>H</u>CN(Dipp))$_2$C)
**^{31}P{^{1}H}-NMR** (CD$_2$Cl$_2$, 300 K):	δ/ ppm = 1.3 (ddd, $^2J_{PA\text{-}PM}$ = 145 Hz, $^1J_{PA\text{-}PQ}$ = 188 Hz, $^3J_{PA\text{-}PX}$ = 40 Hz, 1 P, P^A), -36.4 (td, $^2J_{PM\text{-}PA}$ = 145 Hz, $^1J_{PM\text{-}PQ/X}$ = 324 Hz, 1 P, P^M), -67.5 (ddd, $^1J_{PQ\text{-}PM}$ = 324 Hz, $^1J_{PQ\text{-}PA}$ = 188 Hz, $^2J_{PQ\text{-}PX}$ = 366 Hz, 1 P, P^Q), -101.7 (ddd, $^1J_{PX\text{-}PM}$ = 324 Hz, $^2J_{PX\text{-}PQ}$ = 366 Hz, $^3J_{PX\text{-}PA}$ = 39 Hz, 1 P, P^X)
**^{31}P-NMR** (CD$_2$Cl$_2$, 300 K):	δ/ ppm = 1.3 (m (br), significant broadening due to unresolved $^nJ_{P\text{-}H}$ coupling, 1 P, P^A), -36.4 (td, $^2J_{PM\text{-}PA}$ = 145 Hz, $^1J_{PG\text{-}PQ/X}$ = 324 Hz, 1 P, P^M), -67.5 (ddd, $^1J_{PQ\text{-}PM}$ = 324 Hz, $^1J_{PQ\text{-}PA}$ = 188 Hz, $^2J_{PQ\text{-}PX}$ = 366 Hz, 1 P, P^Q), -101.7 (ddd, $^1J_{PX\text{-}PM}$ = 324 Hz, $^2J_{PX\text{-}PQ}$ = 366 Hz, $^3J_{PX\text{-}PA}$ = 40 Hz, 1 P, P^X)
**^{19}F{^{1}H}-NMR** (CD$_2$Cl$_2$, 300 K):	δ/ ppm = -78.8 (s, [OTf]$^-$)
**IR**:	$\tilde{\nu}$(CO)/cm^{-1} = 2016 (m), 1966 (m)

Synthesis of [CpMo(CO)$_2$(η^3-P$_4$Ph$_2$I^{i}Pr$_2$Me$_2$)][OTf] (**1b**)

A1 (644 mg, 1.0 mmol, 1 eq.) was dissolved in 20 mL of THF and I^{i}Pr$_2$Me$_2$ (180 mg, 1.0 mmol, 1.0 eq.) dissolved in 5 mL of THF was added at -80 °C to afford an immediate change of color from yellow to red. The solution was allowed to reach room temperature and stirred for additional 16 h. Afterwards, the solvent was reduced *in vacuo* to 5 mL and *n*-pentane was added to precipitate an orange powder [CpMo(CO)$_2$(η^3-P$_4$Ph$_2$I^{i}Pr$_2$Me$_2$)][OTf] (**1b**) which was washed with 20 mL of *n*-pentane. Drying under reduced pressure afforded **1b** as an orange solid.

Yield: 0.76 g (0.92 mmol, 92%)

ESI(+)-MS (*o*-DFB): *m/z* (%) = 675.1 (100, [**1b**]$^+$), 647.1 (5, [**1b**-CO]$^+$), 181.2 (20, [I^{i}Pr$_2$Me$_2$H]$^+$)

Elemental analysis: Calculated (%) for $C_{31}H_{35}O_5N_2F_3P_4Mo\bullet(C_4H_8O)_{0.5}$:
C: 46.06, H: 4.57, N: 3.25, S: 3.72; found: C: 46.18, H: 4.55, N: 3.38, S: 4.03

^{1}H-NMR (CD$_2$Cl$_2$, 300 K): *δ*/ ppm = 1.67 (d, 6 H, CH$\underline{Me_2}$), 1.75 (d, 6 H, CH$\underline{Me_2}$), 2.42 (s, 6 H, ($\underline{Me}$CN(^{i}Pr))$_2$C), 5.32 (s, 5 H, C$_5\underline{H_5}$), 5.55 (m, 2 H, C$\underline{H}$Me$_2$), 7.39 – 7.78 (several overlapping multiplets, 10 H, Ph)

^{31}P{^{1}H}-NMR (CD$_2$Cl$_2$, 300 K): *δ*/ ppm = 2.0 (ddd, $^2J_{PA-PM}$ = 158 Hz, $^1J_{PA-PQ}$ = 189 Hz, $^3J_{PA-PX}$ = 39 Hz, 1 P, P^A), -25.5 (td, $^2J_{PM-PA}$ = 158 Hz, $^1J_{PM-PQ/X}$ = 327 Hz, 1 P, P^M), -87.6 (ddd, $^1J_{PQ-PM}$ = 327 Hz, $^1J_{PQ-PA}$ = 189 Hz, $^2J_{PQ-PX}$ = 361 Hz, 1 P, P^Q), -126.0 (ddd, $^1J_{PX-PM}$ = 327 Hz, $^2J_{PX-PQ}$ = 362 Hz, $^3J_{PX-PA}$ = 39 Hz, 1 P, P^X)

^{31}P-NMR (CD$_2$Cl$_2$, 300 K): *δ*/ ppm = 2.0 (m (br), significant broadening due to unresolved $^nJ_{P-H}$ coupling, 1 P, P^A), -25.5 (td, $^2J_{PM-PA}$ = 158 Hz, $^1J_{PM-PQ/X}$ = 327 Hz, 1 P, P^M), -87.6 (ddd, $^1J_{PQ-PM}$ = 327 Hz, $^1J_{PQ-PA}$ = 189 Hz, $^2J_{PQ-PX}$ = 361 Hz, 1 P, P^Q), -126.0 (ddd, $^1J_{PX-PM}$ = 327 Hz, $^2J_{PX-PQ}$ = 362 Hz, $^3J_{PX-PA}$ = 39 Hz, 1 P, P^X)

^{19}F{^{1}H}-NMR (CD$_2$Cl$_2$, 300 K): *δ*/ ppm = -78.7 (s, [OTf]$^-$)

IR: $\tilde{\nu}$(CO)/cm^{-1} = 2007 (m), 1965 (m)

Synthesis of [CpMo(CO)$_2$(η^3-P$_4$^{i}Pr$_2$IDipp)][TEF] (**1c**)

A2 (1.39 g, 1.0 mmol, 1 eq.) was dissolved in 15 mL of *o*-DFB and IDipp (390 mg, 1.0 mmol, 1 eq.) dissolved in 5 mL of *o*-DFB was added at room temperature to afford an immediate change of color from yellow to red. The solution was stirred for 3 h. Afterwards, the solution was concentrated to 3 mL and 50 mL of *n*-hexane were added to precipitate [CpMo(CO)$_2$(η^3-P$_4$^{i}Pr$_2$IDipp)][TEF] (**1c**) as an orange powder. The supernatant was decanted and the solid washed with 15 mL of *n*-hexane, each. Drying under reduced pressure afforded **1c** as an orange solid, which could be recrystallized from concentrated *o*-DFB solutions layered with *n*-hexane and storage at room temperature.

**Yield**:	1.61 g (0.90 mmol, 90%)
**ESI(+)-MS** (*o*-DFB):	*m/z* (%) = 815.2 (95, [**1c**]$^+$), 787.2 (5, [**1c**-CO]$^+$), 389.3 (100, [IDippH]$^+$)
**Elemental analysis**:	Calculated (%) for $C_{56}H_{55}MoP_4F_{36}N_2O_6Al$:<br>C:37.73, H:3.11, N: 1.57; found: C: 38.17 H:3.30, N: 1.54
**^{1}H-NMR** (CD_2Cl_2, 300 K):	δ/ ppm = 1.00 – 1.11 (several overlapping doublets, 15 H, CHMe$_2$), 1.22 (d, 6 H, CHMe$_2$), 1.31 (d, 3 H, CHMe$_2$), 1.38 (d, 6 H, CHMe$_2$), 1.44 (d, 6 H, CHMe$_2$), 1.83 (m, 1 H, CHMe$_2$), 1.90 (m, 1 H, CHMe$_2$), 2.58 (m, 4 H, CHMe$_2$), 4.99 (s, 5 H, C_5H_5), 7.43 (s, 2 H, (HCN(Dipp))$_2$C), 7.45-7.56 (m, 4 H, Dipp), 7.71 (m, 2 H, Dipp)
**^{31}P{^{1}H}-NMR** (CD_2Cl_2, 300 K):	δ/ ppm = 35.6 (ddd, $^2J_{PA\text{-}PM}$ = 129 Hz, $^1J_{PA\text{-}PQ}$ = 204 Hz, $^3J_{PA\text{-}PX}$ = 20 Hz, 1 P, P^A), -22.0 (td, $^2J_{PM\text{-}PA}$ = 129 Hz, $^1J_{PM\text{-}PQ/X}$ = 329 Hz, 1 P, P^M), -83.6 (ddd, $^1J_{PQ\text{-}PM}$ = 329 Hz, $^1J_{PQ\text{-}PA}$ = 204 Hz, $^2J_{PQ\text{-}PX}$ = 350 Hz, 1 P, P^Q), -95.5 (ddd, $^1J_{PX\text{-}PM}$ = 329 Hz, $^2J_{PX\text{-}PQ}$ = 350 Hz, $^3J_{PX\text{-}PA}$ = 20 Hz, 1 P, P^X)
**^{31}P-NMR** (CD_2Cl_2, 300 K):	δ/ ppm = 35.6 (m (br), significant broadening due to unresolved $^nJ_{P\text{-}H}$ coupling, 1 P, P^A), -22.0 (td, $^2J_{PM\text{-}PA}$ = 129 Hz, $^1J_{PM\text{-}PQ/X}$ = 329 Hz, 1 P, P^M), -83.6 (ddd, $^1J_{PQ\text{-}PM}$ = 329 Hz, $^1J_{PQ\text{-}PA}$ = 204 Hz, $^2J_{PQ\text{-}PX}$ = 350 Hz, 1 P, P^Q), -95.5 (ddd, $^1J_{PX\text{-}PM}$ = 329 Hz, $^2J_{PX\text{-}PQ}$ = 350 Hz, $^3J_{PX\text{-}PA}$ = 20 Hz, 1 P, P^X)
**^{19}F{^{1}H}-NMR** (CD_2Cl_2, 300 K):	δ/ ppm = -75.6 (s, [TEF]$^-$)
**IR**:	$\tilde{\nu}$(CO)/cm^{-1} = 2022 (m), 1979 (m)

Synthesis of $[CpMo(CO)_2(\eta^3\text{-}P_4{}^iPr_2I^iPr_2Me_2)][TEF]$ (**1d**)

A2 (1.39 g, 1.0 mmol, 1 eq.) was dissolved in 20 mL of THF and $I^iPr_2Me_2$ (180 mg, 1.0 mmol, 1.0 eq.) dissolved in 5 mL of THF was added at -80 °C to afford an immediate change of color from yellow to red. The solution was allowed to reach room temperature and stirred for additional 3 h. Afterwards, the solvent was removed *in vacuo.* 5 mL of *o*-DFB were added and then *n*-pentane was added to precipitate an orange powder $[CpMo(CO)_2(\eta^3\text{-}P_4{}^iPr_2I^iPr_2Me_2)][TEF]$ (**1d**) which was washed with 20 mL of *n*-hexane. Drying under reduced pressure afforded **1d** as an orange solid, which could be recrystallized from concentrated o-DFB solutions layered with *n*-hexane and storage at room temperature.

**Yield**:	1.44 g (0.91 mmol, 91%)
**ESI(+)-MS** (*o*-DFB):	*m/z* (%) = 607.1 (100, [**1d**]$^+$), 679.1 (10, [**1d**-CO]$^+$), 428.9 (10, [**A2**]$^+$) 181.2 (40, $[I^iPr_2Me_2H]^+$)
**Elemental analysis***:	Calculated (%) for $C_{40}H_{39}N_2F_{36}P_4MoO_6$:<br>C: 30.51, H: 2.50, N: 1.78; found: C: 31.45, H: 2.80, N: 2.01
^{1}**H-NMR** (CD_2Cl_2, 300 K):	δ/ ppm = 1.1 – 1.5 (several overlapping multiplets, 24 H, CH<u>Me</u>$_2$) 1.56 (d, 6 H, NCH<u>Me$_2$</u>), 1.64 (d, 6 H, NCH<u>Me$_2$</u>), 1.97 (m, 1 H, P(C<u>H</u>Me$_2$)$_2$), 2.15 (m, 1 H, P(C<u>H</u>Me$_2$)$_2$), 2.31 (s, 6 H, (<u>Me</u>$_2$CN(^{i}Pr))$_2$C),5.48 (m, 2 H, C<u>H</u>Me$_2$), 5.65 (s, 5 H, C$_5$<u>H$_5$</u>)
^{31}**P{^{1}H}-NMR** (CD_2Cl_2, 300 K):	δ/ ppm = 35.0 (ddd, $^2J_{PA\text{-}PM}$ = 117 Hz, $^1J_{PA\text{-}PQ}$ = 208 Hz, $^3J_{PA\text{-}PX}$ = 17 Hz, 1 P, P^A), -14.6 (td, $^2J_{PM\text{-}PA}$ = 117 Hz, $^1J_{PM\text{-}PQ/X}$ = 333 Hz, 1 P, P^M), -96.0 (ddd, $^1J_{PQ\text{-}PM}$ = 333 Hz, $^1J_{PQ\text{-}PA}$ = 208 Hz, $^2J_{PQ\text{-}PX}$ = 358 Hz, 1 P, P^Q), -120.1 (ddd, $^1J_{PX\text{-}PM}$ = 333 Hz, $^2J_{PX\text{-}PQ}$ = 358 Hz, $^3J_{PX\text{-}PA}$ = 17 Hz, 1 P, P^X)
^{31}**P-NMR** (CD_2Cl_2, 300 K):	δ/ ppm = 35.0 (m (br), significant broadening due to unresolved $^nJ_{P\text{-}H}$ coupling, 1 P, P^A), -14.6 (td, $^2J_{PM\text{-}PA}$ = 117 Hz, $^1J_{PM\text{-}PQ/X}$ = 333 Hz, 1 P, P^M), -96.0 (ddd, $^1J_{PQ\text{-}PM}$ = 333 Hz, $^1J_{PQ\text{-}PA}$ = 208 Hz, $^2J_{PQ\text{-}PX}$ = 358 Hz, 1 P, P^Q), -120.1 (ddd, $^1J_{PX\text{-}PM}$ = 333 Hz, $^2J_{PX\text{-}PQ}$ = 358 Hz, $^3J_{PX\text{-}PA}$ = 17 Hz, 1 P, P^X)
^{19}**F{^{1}H}-NMR** (CD_2Cl_2, 300 K):	δ/ ppm = -75.6 (s, [TEF]$^-$)
**IR**:	$\tilde{\nu}$(CO)/cm^{-1} = 2028 (m), 1979 (m)

* Notably, even after several tries and working under rigorous inert gas conditions, a perfectly fitting elemental analysis could not be obtained, which is presumably a reason of the high air sensitivity of **1d**.

Reactivity of **1b** towards KOEt

1b (82 mg, 0.1 mmol, 1eq.) was dissolved in 5 mL of THF and KOEt (9 mg, 0.1 mmol, 1 eq.) dissolved in 3 mL of THF was added at -80 °C affording an immediate change of color from yellow to light red. Upon letting the solution reach room temperature it changes in color to a intense red. After stirring the solution at room temperature for 1 h the solvent was removed *in vacuo*. The solid was washed with 20 mL of *n*-hexane and subsequently dried under reduced pressure (10^{-3} mbar). The solid was dissolved in 7 mL of toluene, filtered through a glass fiber filter paper, concentrated to 3 mL and stored at -30 °C to afford [CpMo(CO)$_2$($\eta^{1:1}$-I^{i}Pr$_2$Me$_2$PPP(OEt)PPh$_2$)] (**2**) as a crystalline material suitable for X-ray analysis.

**Crystalline Yield**:	13 mg (0.018 mmol, 18%)
**LIFDI-MS** (toluene/THF):	*m/z* (%) = 722.1 (10, [**2**]$^+$), 675.1 (10, [**2**-OEt]$^+$), 181.2 (100, [I^{i}Pr$_2$Me$_2$H]$^+$)
**Elemental analysis**:	Calculated (%) for $C_{32}H_{40}MoP_4N_2O_3$·(C_7H_8): C:57.64, H:5.95, N: 3.45; found: C: 57.90, H:6.05, N: 3.72
**^{1}H-NMR** (C_6D_6, 300 K):	*δ*/ ppm = 1.10-1.15 (m (*br*), 8 H, CH<u>Me$_2$</u> + <u>CH$_2$</u>CH$_3$), 1.19 (t, $^3J_{H-H}$ = 7 Hz, 3 H, CH$_2$<u>CH$_3$</u>) 1.31 (d, $^3J_{H-H}$ = 6 Hz, 6 H, CH<u>Me$_2$</u>), 1.42 (s, 6 H, (<u>Me</u>CN(^{i}Pr))$_2$C), 4.83 (s, 5 H, C$_5$<u>H$_5$</u>), 6.03 (s (*br*), 2 H, C<u>H</u>Me$_2$), 7.17, 8.12-8.24 (several overlapping multiplets, 10 H, Ph) Integrals are slightly off due to the presence of [I^{i}Pr$_2$Me$_2$H]$^+$.
**^{31}P{^{1}H}-NMR** (C_6D_6/THF, 300 K):	*δ*/ ppm = 80.8 (ddd, $^2J_{PA-PM}$ = 177 Hz, $^1J_{PA-PY}$ = 261 Hz, $^1J_{PA-PX}$ = 232 Hz, 1 P, P^A), 0.2 (ddd, $^3J_{PM-PY}$ = 44 Hz, $^2J_{PM-PA}$ = 177 Hz, $^1J_{PM-PX}$ = 191 Hz, 1 P, P^M), -70.7 (ddd, $^1J_{PX-PM}$ = 191 Hz, $^1J_{PX-PA}$ = 232 Hz, $^2J_{PX-PY}$ = 277 Hz, 1 P, P^X), -74.7 (ddd, $^1J_{PY-PA}$ = 261 Hz, $^2J_{PY-PX}$ = 277 Hz, $^3J_{PY-PM}$ = 44 Hz, 1 P, P^Y)
**^{31}P-NMR** (C_6D_6/THF, 300 K):	*δ*/ ppm = 78.0 (ddd, $^2J_{PA-PM}$ = 177 Hz, $^1J_{PA-PY}$ = 261 Hz, $^1J_{PA-PX}$ = 232 Hz, 1 P, P^A), 0.2 (m (br), significant broadening due to unresolved $^nJ_{P-H}$ coupling, 1 P, P^M), -70.7 (ddd, $^1J_{PX-PM}$ = 191 Hz, $^1J_{PX-PA}$ = 232 Hz, $^2J_{PX-PY}$ = 277 Hz, 1 P, P^X), -74.7 (ddd, $^1J_{PY-PA}$ = 261 Hz, $^2J_{PY-PX}$ = 277 Hz, $^3J_{PY-PM}$ = 44 Hz, 1 P, P^Y)
**IR**:	$\tilde{\nu}$(CO)/cm^{-1} = 1899 (s), 1824 (s)

Synthesis of [Cp'''Ni($\eta^{1:1}$-P_4Ph_2IDipp)][PF_6] (**3a**)

B1 (1 g, 1.4 mmol, 1 eq.) was dissolved in 4 mL of *o*-DFB and IDipp (544 mg, 1.4 mmol, 1 eq.) dissolved in 4 mL of *o*-DFB was added at -30 °C to afford an immediate change of color from dark red to yellowish brown. The solution was allowed to reach room temperature and stirred for additional 2 h. Afterwards, the solution was constrained to 3 mL and 50 mL of *n*-hexane were added to precipitate [Cp'''Ni($\eta^{1:1}$-P_4Ph_2IDipp)][PF_6] (**3a**) as an olive green powder. The supernatant was decanted and the solid washed three times with 15 mL of *n*-hexane, each. Drying under reduced pressure afforded **3a** as an olive green solid, which could be recrystallized from concentrated THF solutions layered with *n*-hexane and storage at room temperature.

Yield: 1.39 g (90%)

ESI(+)-MS (*o*-DFB): *m/z* (%) = 957.42 (100, [**3a**]$^+$), 389.30 (10, [IDippH]$^+$)

Elemental analysis: Calculated (%) for $C_{56}H_{75}NiP_5F_6N_2$: C:60.94, H:6.85, N: 2.54; found: C: 61.39, H:7.03, N: 2.37

^{1}H-NMR (CD_2Cl_2, 300 K): δ/ ppm = 0.90 (s, 9 H, $C_5H_2{}^t\underline{Bu_3}$), 0.97 (s, 9 H, $C_5H_2{}^t\underline{Bu_3}$), 1.13 (d, 6 H, $CH\underline{Me_2}$), 1.17 (d, 6 H, $CH\underline{Me_2}$), 1.20 (s, 9 H, $C_5H_2{}^t\underline{Bu_3}$), 1.34 (d, 6 H, $CH\underline{Me_2}$), 1.44 (d, 6 H, $CH\underline{Me_2}$), 2.20 (m, 2 H, $C\underline{H}Me_2$), 2.28 (m, 2 H, $C\underline{H}Me_2$), 4.47 (s, 1 H, $C_5\underline{H_2}{}^tBu_3$), 4.68 (s, 1 H, $C_5\underline{H_2}{}^tBu_3$), 6.95 (m, 2 H, Ph), 7.22 (m, 2 H, Ph), 7.35 – 7.75 (several overlapping multiplets, 12 H, Ph and Dipp groups), 7.43 (s, 2 H, ($\underline{H}$CN(Dipp))$_2$C)

^{31}P{^{1}H}-NMR (CD_2Cl_2, 300 K): δ/ ppm = -43.3 (ddd, $^1J_{PA-PM}$ = 167 Hz, $^2J_{PA-PX}$ = 99 Hz, $^2J_{PA-PQ}$ = 34 Hz, 1 P, P^A), -118.7 (ddd, $^1J_{PM-PA}$ = 167 Hz, $^1J_{PM-PX}$ = 155 Hz, $^1J_{PM-PQ}$ = 78 Hz, 1 P, P^M), -143.9 (sept, $^1J_{P-F}$ = 710 Hz, [PF_6]$^-$), -162.2 (ddd, $^1J_{PQ-PX}$ = 263 Hz, $^1J_{PQ-PM}$ = 78 Hz, $^2J_{PQ-PA}$ = 34 Hz, 1 P, P^Q), -175.0 (ddd, $^1J_{PX-PQ}$ = 263 Hz, $^1J_{PX-PM}$ = 155 Hz, $^2J_{PX-PA}$ = 99 Hz, 1 P, P^X)

^{31}P-NMR (CD_2Cl_2, 300 K): δ/ ppm = -43.3 (m (br), significant broadening due to unresolved $^nJ_{P-H}$ coupling, 1 P, P^A), -118.7 (ddd, $^1J_{PM-PA}$ = 167 Hz, $^1J_{PM-PX}$ = 155 Hz, $^1J_{PM-PQ}$ = 78 Hz, 1 P, P^M), -143.9 (sept, $^1J_{P-F}$ = 710 Hz, [PF_6]$^-$), -162.2 (ddd, $^1J_{PQ-PX}$ = 263 Hz, $^1J_{PQ-PM}$ = 78 Hz, $^2J_{PQ-PA}$ = 34 Hz, 1 P, P^Q), -175.0 (ddd, $^1J_{PX-PQ}$ = 263 Hz, $^1J_{PX-PM}$ = 155 Hz, $^1J_{PX-PA}$ = 99 Hz, 1 P, P^X)

^{19}F{^{1}H}-NMR (CD_2Cl_2, 300 K): δ/ ppm = -73.1 (d, $^1J_{F-P}$ = 710 Hz, [PF_6]$^-$)

Synthesis of [Cp'''Ni($\eta^{1:1}$-$P_4Ph_2I^iPr_2Me_2$)][PF_6] (**3b**)

B1 (1.43 g, 2 mmol, 1 eq.) was dissolved in 8 mL of THF and $I^iPr_2Me_2$ (380 mg, 2.1 mmol, 1.05 eq.) dissolved in 10 mL of THF was added at -80 °C to afford an immediate change of color from dark red to brownish red. The solution was allowed to reach room temperature and stirred for additional 16 h. Afterwards, the solvent was removed *in vacuo* and the solid washed two times with 20 mL of *n*-hexane, each. Drying under reduced pressure afforded [Cp'''Ni($\eta^{1:1}$-$P_4Ph_2I^iPr_2Me_2$)][PF_6] (**3b**) as a dark red solid, which could be recrystallized from concentrated THF solutions layered with *n*-hexane and storage at room temperature.

**Yield**:	1.62 g (91%)
**ESI(+)-MS** (*o*-DFB):	*m/z* (%) = 749.3 (100, [**3b**]$^+$), 181.2 (80, [$I^iPr_2Me_2H$]$^+$)
**Elemental analysis**:	Calculated (%) for $C_{40}H_{59}N_2F_6P_5Ni{\bullet}(C_6H_{14})_{0.5}$: C: 55.03, H: 7.09, N: 2.98; found: C: 55.12, H: 6.77, N: 3.02
**^{1}H-NMR** (CD_2Cl_2, 300 K):	δ/ ppm = 0.99 (s, 9 H, $C_5H_2{}^t\underline{Bu_3}$), 1.29 (s, 9 H, $C_5H_2{}^t\underline{Bu_3}$), 1.44 (d, 6 H, $CH\underline{Me_2}$), 1.46 (s, 9 H, $C_5H_2{}^t\underline{Bu_3}$), 1.48 (d, 6 H, $CH\underline{Me_2}$), 2.23 (s, 6 H, ($\underline{Me}$CN(^{i}Pr))$_2$C), 4.96 (s, 1 H, $C_5\underline{H_2}{}^tBu_3$), 5.05 (s, 1 H, $C_5\underline{H_2}{}^tBu_3$), 5.58 (m, 2 H, $C\underline{H}Me_2$), 7.4 – 7.8 (several overlapping multiplets, 10 H, Ph)
**^{31}P{^{1}H}-NMR** (CD_2Cl_2, 300 K):	δ/ ppm = -48.1(ddd, $^1J_{PA\text{-}PM}$ = 156 Hz, $^2J_{PA\text{-}PX}$ = 114 Hz, $^2J_{PA\text{-}PQ}$ = 35 Hz, 1 P, P^A), -93.4 (td, $^1J_{PM\text{-}PA/X}$ = 156 Hz, $^1J_{PM\text{-}PQ}$ = 94 Hz, 1 P, P^M), -138.8 (ddd, $^1J_{PQ\text{-}PX}$ = 247 Hz, $^2J_{PQ\text{-}PM}$ = 94 Hz, $^2J_{PQ\text{-}PA}$ = 35 Hz, 1 P, P^Q), -143.9 (sept, $^1J_{P\text{-}F}$ = 710 Hz, [PF_6]$^-$), -181.3 (ddd, $^1J_{PX\text{-}PQ}$ = 247 Hz, $^1J_{PX\text{-}PM}$ = 156 Hz, $^2J_{PX\text{-}PA}$ = 114 Hz, 1 P, P^X)
**^{31}P-NMR** (CD_2Cl_2, 300 K):	δ/ ppm = -48.1(m (br), significant broadening due to unresolved $^nJ_{P\text{-}H}$ coupling, 1 P, P^A), -93.4 (td, $^1J_{PM\text{-}PA/X}$ = 156 Hz, $^1J_{PM\text{-}PQ}$ = 94 Hz, 1 P, P^M), -138.8 (ddd, $^1J_{PQ\text{-}PX}$ = 247 Hz, $^2J_{PQ\text{-}PM}$ = 94 Hz, $^2J_{PQ\text{-}PA}$ = 35 Hz, 1 P, P^Q), -143.9 (sept, $^1J_{P\text{-}F}$ = 710 Hz, [PF_6]$^-$), -181.3 (ddd, $^1J_{PX\text{-}PQ}$ = 247 Hz, $^1J_{PX\text{-}PM}$ = 156 Hz, $^2J_{PX\text{-}PA}$ = 114 Hz, 1 P, P^X)
**^{19}F{^{1}H}-NMR** (CD_2Cl_2, 300 K):	δ/ ppm = -73.1 (d, $^1J_{F\text{-}P}$ = 710 Hz, [PF_6]$^-$)

Synthesis of [Cp'''Ni($\eta^{1:1}$-$P_4{}^iPr_2$IDipp)][PF_6] (**3c**)

B2 (1.04 g, 1.6 mmol, 1 eq.) was dissolved in 8 mL of THF and IDipp (652 mg, 1.68 mmol, 1.05 eq.) dissolved in 10 mL of THF was added at -80 °C to afford an immediate change of color from dark red to brownish red. The solution was allowed to reach room temperature, leading to a change of color to yellowish brown, and stirred for additional 16 h. Afterwards, the solvent was removed *in vacuo* and the solid washed two times with 15 mL of *n*-hexane, each. Drying under reduced pressure afforded [Cp'''Ni($\eta^{1:1}$-$P_4{}^iPr_2$IDipp)][PF_6] (**3c**) as a dark yellowish brown solid, which could be recrystallized from concentrated *o*-DFB solutions layered with *n*-hexane and storage at room temperature.

Yield: 1.62 g (91%)

ESI(+)-MS (*o*-DFB): *m/z* (%) = 889.5 (100, [**3c**]$^+$), 389.3 (40, [IDippH]$^+$)

Elemental analysis: Calculated (%) for $C_{50}H_{79}N_2F_6P_5Ni{\bullet}(C_6H_{14})_{0.5}$: C: 59.01, H: 8.04, N: 2.60; found: C: 59.46, H: 8.38, N: 2.60

^{1}H-NMR (CD_2Cl_2, 300 K): *δ*/ ppm = 0.66 (dd, 6 H, $CH\underline{Me_2}$), 1.05 – 1.23 (several overlapping doublets, 15 H, $CH\underline{Me_2}$), 1.11 (s, 9 H, $C_5H_2\underline{{}^tBu_3}$), 1.17 (s, 9 H, $C_5H_2\underline{{}^tBu_3}$), 1.21 (s, 9 H, $C_5H_2\underline{{}^tBu_3}$), 1.47 (dd, 6 H, $CH\underline{Me_2}$), 1.58 (dd, 3 H, $CH\underline{Me_2}$), 1.65 (m, 1 H, $C\underline{H}Me_2$), 2.21 (m, 3 H, $C\underline{H}Me_2$), 2.32 (m, 2 H, $C\underline{H}Me_2$), 4.41 (s, 1 H, $C_5\underline{H_2}{}^tBu_3$), 4.46 (s, 1 H, $C_5\underline{H_2}{}^tBu_3$), 7.39 (m, 4 H, Dipp), 7.50 (s, 2 H, $(\underline{H}CN(Dipp))_2C$), 7.61 (m, 2 H, 69

^{31}P{^{1}H}-NMR (CD_2Cl_2, 300 K): *δ*/ ppm = -12.8 (ddd, $^1J_{PA\text{-}PM}$ = 173 Hz, $^2J_{PA\text{-}PX}$ = 80 Hz, $^2J_{PA\text{-}PQ}$ 29 Hz, 1 P, P^A), -143.9 (sept, $^1J_{P\text{-}F}$ = 710 Hz, [PF_6]$^-$), -151.3 (ddd, $^1J_{PM\text{-}PA}$ = 173 Hz, $^1J_{PM\text{-}PX}$ = 154 Hz, $^1J_{PM\text{-}PQ}$ = 82 Hz, 1 P, P^M), -157.8 (ddd, $^1J_{PQ\text{-}PX}$ = 262 Hz, $^1J_{PQ\text{-}PM}$ = 82 Hz, $^2J_{PQ\text{-}PA}$ = 29 Hz, 1 P, P^Q), -181.8 (ddd, $^1J_{PX\text{-}PQ}$ = 262 Hz, $^1J_{PX\text{-}PM}$ = 154 Hz, $^2J_{PX\text{-}PA}$ = 80 Hz, 1 P, P^X)

^{31}P-NMR (CD_2Cl_2, 300 K): *δ*/ ppm = -12.8 (m (br), significant broadening due to unresolved $^nJ_{P\text{-}H}$ coupling, 1 P, P^A), -143.9 (sept, $^1J_{P\text{-}F}$ = 710 Hz, [PF_6]$^-$), -151.3 (ddd, $^1J_{PM\text{-}PA}$ = 173 Hz, $^1J_{PM\text{-}PX}$ = 154 Hz, $^1J_{PM\text{-}PQ}$ = 82 Hz, 1 P, P^M), -157.8 (ddd, $^1J_{PQ\text{-}PX}$ = 262 Hz, $^1J_{PQ\text{-}PM}$ = 82 Hz, $^2J_{PQ\text{-}PA}$ = 29 Hz, 1 P, P^Q), -181.8 (ddd, $^1J_{PX\text{-}PQ}$ = 262 Hz, $^1J_{PX\text{-}PM}$ = 154 Hz, $^2J_{PX\text{-}PA}$ = 80 Hz, 1 P, P^X)

^{19}F{^{1}H}-NMR (CD_2Cl_2, 300 K): *δ*/ ppm = -73.1 (d, $^1J_{F\text{-}P}$ = 710 Hz, [PF_6]$^-$)

Synthesis of [Cp'''Ni($\eta^{1:1}$-$P_4{}^iPr_2I^iPr_2Me_2$)][PF_6] (**3d**)

B2 (1.04 g, 1.6 mmol, 1 eq.) was dissolved in 8 mL of THF and $I^iPr_2Me_2$ (302 mg, 1.68 mmol, 1.05 eq.) dissolved in 10 mL of THF was added at -80 °C to afford an immediate change of color from dark red to brownish red. The solution was allowed to reach room temperature, leading to a change of color to yellowish brown, and stirred for additional 16 h. Afterwards, the solvent was removed *in vacuo* and the solid washed three times with 15 mL of *n*-hexane, each. Drying under reduced pressure afforded [Cp'''Ni($\eta^{1:1}$-$P_4{}^iPr_2I^iPr_2Me_2$)][PF_6] (**3d**) as a dark brown solid, which could be recrystallized from concentrated *o*-DFB solutions layered with *n*-hexane and storage at room temperature.

**Yield**:	1.08 g (81%)
**ESI(+)-MS** (*o*-DFB):	*m/z* (%) = 681.3 (100, [**3d**]$^+$), 181.2 (10, [$I^iPr_2Me_2H$]$^+$)
**Elemental analysis**:	Calculated (%) for $C_{34}H_{63}N_2F_6P_5Ni$: C: 49.35, H:7.67, N: 3.39; found: C: 49.07, H: 7.67, N: 4.00
**^{1}H-NMR** (CD_2Cl_2, 300 K):	*δ*/ ppm = 1.2 – 1.7 (several overlapping multiplets, 24 H, CH<u>Me</u>$_2$), 1.27 (s, 9 H, $C_5H_2{}^t\underline{Bu_3}$), 1.40 (s, 9 H, $C_5H_2{}^t\underline{Bu_3}$), 1.45 (s, 9 H, $C_5H_2{}^t\underline{Bu_3}$), 2.20 (m, 1 H, P(C$\underline{H}Me_2$)$_2$), 2.27 (s, 6 H, ($\underline{Me}$CN(^{i}Pr))$_2$C), 2.30 (m, 1 H, P(C$\underline{H}Me_2$)$_2$), 4.80 (s, 1 H, $C_5\underline{H_2}{}^tBu_3$), 4.92 (s, 1 H, $C_5\underline{H_2}{}^tBu_3$), 5.77 (m, 2 H, C$\underline{H}Me_2$), trace amounts of [$I^iPr_2Me_2$]$^+$ overlapping
**^{31}P{^{1}H}-NMR** (CD_2Cl_2, 300 K):	*δ*/ ppm = -11.9 (ddd, $^1J_{PA-PQ}$ = 163 Hz, $^2J_{PA-PX}$ = 79 Hz, $^2J_{PA-PM}$ = 30 Hz, 1 P, P^A), -130.7 (ddd, $^1J_{PM-PX}$ = 238 Hz, $^1J_{PM-PQ}$ = 97 Hz, $^2J_{PM-PA}$ = 30 Hz, 1 P, P^M), -135.8 (td, $^1J_{PQ-PA/X}$ = 163 Hz, $^1J_{PQ-PM}$ = 97 Hz, 1 P, P^Q), -186.0 (ddd, $^1J_{PX-PM}$ = 238 Hz, $^1J_{PX-PQ}$ = 163 Hz, $^2J_{PX-PA}$ = 79 Hz, 1 P, P^X)
**^{31}P-NMR** (CD_2Cl_2, 300 K):	*δ*/ ppm = -11.9 (m (br), significant broadening due to unresolved $^nJ_{P-H}$ coupling, 1 P, P^A), -130.7 (ddd, $^1J_{PM-PX}$ = 238 Hz, $^1J_{PM-PQ}$ = 97 Hz, $^2J_{PM-PA}$ = 30 Hz, 1 P, P^M), -135.8 (td, $^1J_{PN-PA/X}$ = 163 Hz, $^1J_{PQ-PM}$ = 97 Hz, 1 P, P^Q), -186.0 (ddd, $^1J_{PX-PM}$ = 238 Hz, $^1J_{PX-PQ}$ = 163 Hz, $^2J_{PX-PA}$ = 79 Hz, 1 P, P^X)
**^{19}F{^{1}H}-NMR** (CD_2Cl_2, 300 K):	*δ*/ ppm = -73.1 (d, $^1J_{F-P}$ = 710 Hz, [PF_6]$^-$)

Reactivity of **3c** towards KOEt

3c (207 mg, 0.2 mmol, 1eq.) was dissolved in 3 mL of THF and KOEt (18 mg, 0.2 mmol, 1 eq.) dissolved in 3 mL of THF was added at -80 °C affording an immediate change of color to an intense turquoise. Upon letting the solution reach room temperature it changes in color via greenish brown to a brownish red. After stirring the solution at room temperature for 1 h the solvent was removed *in vacuo*. 20 mL of *n*-hexane were added to the solid to afford a brownish red solution (and a colorless solid), which was filtered and then constrained to 0.1 mL upon which [Cp'''Ni(η^2-IDippPP(OEt)PP^{i}Pr$_2$)] (**4**) started to precipitate. The mixture was dissolved in 4 mL of toluene, layered with 30 mL of acetonitrile and stored at 4 °C to afford **4** as a crystalline material suitable for X-ray analysis. The crude product of **4** can be obtained by completely removing the *n*-hexane after filtration and not performing the recrystallization.

Notably, the intermediate $\mathbf{4_{INT}}$ could not be isolated as a pure compound due to its rapid degradation above -10 °C. However, it was possible to study this highly sensitive species by means of low temperature NMR spectroscopy (*vide infra*). Furthermore, gas phase diffusion at -30 °C even allowed for the growth of a single crystal of $\mathbf{4_{INT}}$ and thus its crystallographic study.

4

**Yield**:	125 mg (67%)
**LIFDI-MS** (toluene):	*m/z* (%) = 879.4 (15, unassigned fragmentation product), 389.4 (100, [IDipp]$^+$)
**Elemental analysis**:	Calculated (%) for $C_{52}H_{84}N_2OP_4Ni \cdot (C_2H_3N)_{0.2} \cdot (C_6H_{14})_{0.4}$: C: 67.26, H: 9.29, N: 3.15; found: C: 67.74, H: 9.70, N: 3.49
**^{1}H-NMR** (C_6D_6, 300 K):	δ/ ppm = 1.05 – 1.13 (overlapping doublets, 18 H, CH<u>Me$_2$</u>), 1.16 (t, 3 H, OCH$_2$<u>Me</u>), 1.37 (s, 9 H, C$_5$H$_2$<u>^{t}Bu$_3$</u>), 1.40 – 1.48 (overlapping doublets, 18 H, CH<u>Me$_2$</u>), 1.55 (s, 9 H, C$_5$H$_2$<u>^{t}Bu$_3$</u>), 1.56 (s, 9 H, C$_5$H$_2$<u>^{t}Bu$_3$</u>), 2.15 (m, 1 H, P(C<u>H</u>Me$_2$)$_2$), 2.54 (m, 1 H, P(C<u>H</u>Me$_2$)$_2$), 2.92 (m, 2 H, C<u>H</u>Me$_2$), 3.05 (m, 2 H, C<u>H</u>Me$_2$), 3.94 (br, 1 H, OC<u>H$_2$</u>Me), 4.08 (br, 1 H, OC<u>H$_2$</u>Me), 5.20 (m, 2 H, C$_5$<u>H$_2$</u>^{t}Bu$_3$), 6.18 (s, 2 H, (<u>H</u>CN(Dipp))$_2$C), 7.11 (m, 4 H, Dipp), 7.25 (m, 2 H, Dipp), integrals are slightly off due to the presence of isomers
**^{31}P{^{1}H}-NMR** (C_6D_6, 300 K):	δ/ ppm = 123.7 (ddd, $^1J_{PA-PQ}$ = 335 Hz, $^1J_{PA-PX}$ = 239 Hz, $^2J_{PA-PM}$ = 30 Hz, 1 H, P^A), 50.7 (dd, $^1J_{PM-PX}$ = 472 Hz, $^2J_{PM-PA}$ = 30 Hz, 1 H, P^M), -47.6 (dd, $^1J_{PQ-PA}$ = 335 Hz, $^2J_{PQ-PX}$ = 272 Hz, 1 H, P^Q), -121.2 (ddd, $^1J_{PX-PM}$ = 472 Hz, $^2J_{PX-PQ}$=272 Hz, $^1J_{PX-PA}$ = 239 Hz, 1 H, P^X)
**^{31}P-NMR** (C_6D_6, 300 K):	δ/ ppm = 123.7 (ddd, $^1J_{PA-PQ}$ = 335 Hz, $^1J_{PA-PX}$ = 239 Hz, $^2J_{PA-PM}$ = 30 Hz, 1 H, P^A), 50.7 (d (br), significant broadening due to $^nJ_{P-H}$ coupling,1 P, P^M), -47.6 (dd, $^1J_{PQ-PA}$ = 335 Hz, $^2J_{PQ-PX}$ = 272 Hz, 1 H, P^Q), -121.2 (ddd, $^1J_{PX-PM}$ = 472 Hz, $^2J_{PX-PQ}$=272 Hz, $^1J_{PX-PA}$ = 239 Hz, 1 H, P^X)

Spectroscopic Data for **4**$_{\mathbf{INT}}$

^{31}P{^{1}H}-NMR (C_6D_6, 300 K): δ/ ppm = 84.1 (ddd, $^{1}J_{PA-PM}$ = 249 Hz, $^{1}J_{PA-PX}$ = 233 Hz, $^{2}J_{PA-PY}$ = 94 Hz, 1 P, P^{A}), 23.8 (ddd, $^{1}J_{PM-PA}$ = 249 Hz, $^{2}J_{PM-PY}$ = 57 Hz, $^{2}J_{PM-PX}$ = 41 Hz, 1 P, P^{M}), -34.0 (ddd, $^{1}J_{PX-PY}$ = 339 Hz, $^{1}J_{PX-PA}$ = 233 Hz, $^{2}J_{PX-PM}$ = 41 Hz, 1 P, P^{X}), -40.1 (ddd, $^{1}J_{PY-PX}$ = 339 Hz, $^{2}J_{PY-PA}$ = 94 Hz, $^{2}J_{PY-PM}$ = 57 Hz, 1 P , P^{Y})

^{31}P-NMR (C_6D_6, 300 K): δ/ ppm = 84.1 (ddd, $^{1}J_{PA-PM}$ = 249 Hz, $^{1}J_{PA-PX}$ = 233 Hz, $^{2}J_{PA-PY}$ = 94 Hz, 1 P, P^{A}), 23.8 (br, significant broadening due to $^{n}J_{P-H}$ coupling, 1 P, P^{M}), -34.0 (ddd, $^{1}J_{PX-PY}$ = 339 Hz, $^{1}J_{PX-PA}$ = 233 Hz, $^{2}J_{PX-PM}$ = 41 Hz, 1 P, P^{X}), -40.1 (ddd, $^{1}J_{PY-PX}$ = 339 Hz, $^{2}J_{PY-PA}$ = 94 Hz, $^{2}J_{PY-PM}$ = 57 Hz, 1 P , P^{Y})

Reactivity of **4** towards MeOTf (Synthesis of **5**)

4 (93 mg, 0.1 mmol, 1eq.) was dissolved in a mixture of 2 mL of *o*-DFB and 2 mL of *m*-DFB (1,3-difluorobenzene) and MeOTf (11 µL, 0.1 mmol, 1 eq.) was added at -80 °C. The brownish red solution was stirred at -80 °C for 10 min. After letting the solution reach room temperature over the course of 2 h the solvent was removed under reduced pressure. The residue was washed with 5 mL of toluene and two times with 10 mL of *n*-hexane, each. Recrystallisation from a concentrated CH_2Cl_2 solution (3 mL) layered with 30 mL of *n*-pentane afforded purple plate shaped crystals of the product [Cp'''Ni(η^2-MeP(OEt)P(IDippP)P^{i}Pr$_2$)][OTf] (**5**). However, **5** is contaminated by co-crystallizing [IDippMe][OTf], which cannot be separated from the product. Thus, **5** could only be obtained in amounts of few single crystals hampering its characterization beyond mass spectrometry.

Yield: few single crystals

ESI(+)-MS (*o*-DFB): *m/z* (%) = 949.5 (60, [**5**]$^+$), 403.3 (100, [IDippMe]$^+$)

Cyanolysis of [Cp'''Ni($\eta^{1:1}$-P_4R_2IDipp)][PF_6] (R = Ph, ^{i}Pr)

3a/**3c** (663 mg (**3a**) or 621 mg (**3c**), 0.6 mmol, 1 eq.) and [Et_4N][CN] (94 mg, 0.6 mmol, 1 eq.) were mixed and dissolved in 10 mL of THF, resulting in a slow change of color towards reddish brown. The mixture was stirred at 70 °C for 3 h and then the solvent was removed *in vacuo*. The residue was dissolved in CH_2Cl_2 and SiO_2 (ca. 10 g) were added. The solvent was removed again, and the resulting free flowing solid was transferred onto a column (SiO_2/*n*-hexane, 2/20cm). Eluting with pure *n*-hexane yields a pale yellow and of [Cp'''Ni(η^3-P_3)] which was discarded. In case of **3c** (R = ^{i}Pr), increasing the polarity to *n*-hexane/toluene (10:1) affords an additional small red band of the decomposition product [{Cp'''Ni}$_2$(μ,$\eta^{2:2}$-(PP^{i}Pr$_2$)$_2$)] (**7**). Finally, a mixture of *n*-hexane/toluene (5:1) elutes the main products [{Cp'''Ni}$_2$(μ,$\eta^{1:1:1:1}$-*cyclo*-P_4(PR_2)$_2$)] (**6a**: R = Ph, **6b**: R = ^{i}Pr) as dark brownish red bands. Simple removing the solvent *in vacuo* affords the respective crude product. While **7** can be obtained as a pure compound by recrystallization from concentrated *n*-hexane solutions at 4 °C, **6a**/**b** were further purified by layering concentrated solutions in toluene with acetonitrile and storage at 4 °C.

Analytical Data:

6a:

**Yield**:	194 mg (60%, crude)/50 mg (23%, crystalline)
**LIFDI-MS** (toluene):	*m/z* (%) = 1076.6 (100, [**6a**]$^+$)
**Elemental analysis**:	Calculated (%) for $C_{58}H_{78}P_6Ni_2{\bullet}(C_7H_8)_{0.5}$: C:65.69, H: 7.35; found: C: 65.92, H: 7.52
**^{1}H-NMR** (C_6D_6, 300 K):	δ/ ppm = 1.21 (s, 9 H, $C_5H_2{}^t\underline{Bu_3}$), 1.35 (s, 9 H, $C_5H_2{}^t\underline{Bu_3}$), 1.44 (s, 9 H, $C_5H_2{}^t\underline{Bu_3}$), 5.35 (m, 1 H, $C_5\underline{H_2}{}^tBu_3$), 5.42 (m, 1 H, $C_5\underline{H_2}{}^tBu_3$), 6.90 – 7.15 (several multiplets overlapping with residual solvent signal (C_6D_6), 6 H, Ph), 8.11 (m, 2 H, Ph), 8.24 (m, 2 H, Ph)
**^{31}P{^{1}H}-NMR** (C_6D_6, 300 K):	δ/ ppm = 210.8 (m, 2 P, $P^{A/A'}$), 121.0 (m, 2 P, $P^{M/M'}$), 89.6 (m, 2 P, $P^{X/X'}$)
**^{31}P-NMR** (C_6D_6, 300 K):	δ/ ppm = 210.8 (m, 2 P, $P^{A/A'}$), 121.0 (m, 2 P, $P^{M/M'}$), 89.6 (m (broadening due to unresolved $^nJ_{P\text{-}H}$ coupling), 2 P, $P^{X/X'}$)

The resolution of these ^{31}P NMR spectra is not sufficient enough to reliably simulate the extremely complex spin system of compound **6a**, which is why the provision of coupling constants is neglected.

6b:

**Yield**:	151 mg (53%, crude)/44 mg (24%, crystalline)
**LIFDI-MS** (toluene):	*m/z* (%) = 940.6 (100, [**6b**]$^+$)
**Elemental analysis**:	Calculated (%) for $C_{46}H_{86}Ni_2P_6$: C: 58.63, H: 9.20; found: C: 59.22, H: 9.61
**^{1}H-NMR** (C_6D_6, 300 K):	δ/ ppm = 1.32 – 1.55 (several overlapping doublets/multiplets, 12 H, CH$\underline{Me_2}$), 1.38 (s, 9 H, $C_5H_2{}^t\underline{Bu_3}$), 1.51 (d, 18 H, $C_5H_2{}^t\underline{Bu_3}$), 2.74 (m, 1 H, C$\underline{H}Me_2$),

3.06 (m, 1 H, C<u>H</u>Me$_2$), 5.28 (m, 1 H, C$_5$<u>H$_2$</u>^{t}Bu$_3$), 5.40 (m, 1 H, C$_5$<u>H$_2$</u>^{t}Bu$_3$)

^{31}P{^{1}H}-NMR (C_6D_6, 300 K): δ/ ppm = 201.3 (m, 2 P, $P^{A/A'}$), 126.5 (m, 2 P, $P^{M/M'}$), 80.7 (m, 2 P, $P^{X/X'}$)

^{31}P-NMR (C_6D_6, 300 K): δ/ ppm = 201.3 (m, 2 P, $P^{A/A'}$), 126.5 (m (broadening due to unresolved $^nJ_{P-H}$ coupling), 2 P, $P^{M/M'}$), 80.7 (m, 2 P, $P^{X/X'}$)

The resolution of these ^{31}P NMR spectra is not sufficient enough to reliably simulate the extremely complex spin system of compound **6b**, which is why the provision of coupling constants is neglected.

7:

Yield: 55 mg (20%)

LIFDI-MS (toluene): *m/z* (%) = 894.6 (100, [**7**+O]$^+$)

Elemental analysis: Calculated (%) for $C_{46}H_{86}P_4Ni_2$: C: 62.75, H: 9.85; found: C: 62.96, H: 10.25

^{1}H-NMR (C_6D_6, 300 K): δ/ ppm = 1.04 (dd, 3 H, CH<u>Me$_2$</u>), 1.20 (dd, 3 H, CH<u>Me$_2$</u>), 1.38 (s, 9 H, C$_5$H$_2$<u>^{t}Bu$_3$</u>), 1.53 (s, 9 H, C$_5$H$_2$<u>^{t}Bu$_3$</u>), 1.54 (s, 9 H, C$_5$H$_2$<u>^{t}Bu$_3$</u>), 1.56 – 1.64 (overlapping doublets, 6 H, CH<u>Me$_2$</u>), 2.37 (m, 1 H, C<u>H</u>Me$_2$), 2.96 (m, 1 H, C<u>H</u>Me$_2$), 5.14 (d, 1 H, C$_5$<u>H$_2$</u>^{t}Bu$_3$), 5.28 (d, 1 H, C$_5$<u>H$_2$</u>^{t}Bu$_3$)

^{31}P{^{1}H}-NMR (C_6D_6, 300 K): δ/ ppm = 52.8 (m, $^1J_{PA/A'-PX/X'}$ = 482/481 Hz, $^2J_{PA/A'-PX'/X}$ = 21/22 Hz, $^3J_{PA-PA'}$ = 26 Hz, 2 P, $P^{A/A'}$), -101.3 (m, $^1J_{PX/X'-PA/A'}$ = 482/481 Hz, $^1J_{PX-PX'}$ = 324 Hz, $^2J_{PX/X'-PA'/A}$ = 21/22 Hz, 2 P, $P^{X/X'}$)

^{31}P-NMR (C_6D_6, 300 K): δ/ ppm = 52.8 (m (br), significant broadening due to unresolved $^nJ_{P-H}$ coupling, 2 P, $P^{A/A'}$), -101.3 (m, 2 P, $P^{X/X'}$)

Reactivity of **3a** towards $[ECO]^-$ salts (E = P, As)

3a (234 mg, 0.2 mmol, 1 eq.) was dissolved in 4 mL of THF and NaOCP•diox (34 mg, 0.2 mmol, 1 eq.) or KAsCO (28 mg, 0.2 mmol, 1 eq.) in 6 mL of THF was added at -80 °C affording a rapid change of color to intense red or dark brownish red, respectively. The solution was allowed to reach room temperature and stirred for 2 h. The solvent was removed *in vacuo* and 30 mL of *n*-hexane added to the solid residue. The dark red solution was filtered, and the solvent removed afterwards affording [Cp'''Ni($\eta^{1:1}$-EP_4Ph_2IDipp)] (**8a**: E = P, **8b**: E = As) as dark red powders. Crystals of **8a** could be obtained by gas phase diffusion of a saturated *n*-pentane solution into toluene at room temperature. In contrast, **8b** can simply be recrystallized by layering a concentrated solution in Et_2O upon acetonitrile and storing the mixture at -30 °C yielding the compound as dark red needles suitable for X-ray analysis.

8a:

Compound **8a** could only be obtained in amounts of few single crystals by the method described above. Separation of bulk **8a** from side products is hampered by the low stability of this compound and its decomposition during solvent based work-up.

8b:

**Yield**:	150 mg (73%)
**LIFDI-MS** (toluene):	*m/z* (%) = 950.6 (5, [**8b**-Me]$^+$), 889.6 (4, unassigned fragmentation), 858.6 (4, unassigned fragmentation), 389.4 (100, [IDipp]$^+$)
**Elemental analysis**:	Calculated (%) for $C_{56}H_{75}N_2P_4NiAs$: C: 65.07, H: 7.31, N: 2.71; found: C: 64.85, H: 7.50, N: 2.97
^{1}**H-NMR** (C_6D_6, 300 K):	δ/ ppm = 1.07 – 1.16 (overlapping doublets, 12 H, $CHMe_2$), 1.13 (s, 9 H, $C_5H_2{}^tBu_3$), 1.28 (s, 9 H, $C_5H_2{}^tBu_3$), 1.38 (s, 9 H, $C_5H_2{}^tBu_3$), 1.39 (d, 6 H, $CHMe_2$), 1.58 (d, 6 H, $CHMe_2$), 2.93 (m, 2 H, $CHMe_2$), 3.05(m, 2 H, $CHMe_2$), 4.87 (m, 2 H, $C_5H_2{}^tBu_3$), 6.13 (s, 2 H, $(HCN(Dipp))_2C$), 7.00 (overlapping multiplets, 5 H, Ph and Dipp), 7.16 (overlapping multiplets, 5 H, Ph and Dipp), 7.54 (m, 2 H, Ph), 7.94 (m, 2 H, Ph), integrals are slightly off due to the presence of isomers.
^{31}**P{^{1}H}-NMR** (C_6D_6, 300 K):	δ/ ppm = -31.3 (m, $^1J_{PA\text{-}PY}$ = 207 Hz, $^2J_{PA\text{-}PX}$ = 62 Hz, $^3J_{PA\text{-}PM}$ = 27Hz, 1 P, P^A), -67.5 (m, $^1J_{PM\text{-}PX}$ = 261 Hz, $^2J_{PM\text{-}PY}$ = 104 Hz, $^3J_{PM\text{-}PA}$ = 27 Hz, 1 P, P^M), -148.4 (m, $^1J_{PX\text{-}PM}$ = 261 Hz, $^1J_{PX\text{-}PY}$ = 220 Hz, $^2J_{PX\text{-}PA}$ = 62 Hz, 1 P, P^X), -151.6 (m, $^1J_{PY\text{-}PX}$ = 220 Hz, $^1J_{PY\text{-}PA}$ = 207 Hz, $^2J_{PY\text{-}PM}$ = 104 Hz, 1 P, P^Y)

^{31}P-NMR (C_6D_6, 300 K): δ/ ppm = -31.3 (m (br), significant broadening due to $^{n}J_{P\text{-}H}$ coupling, 1 P, P^{A}), -67.5 (m, $^{1}J_{PM\text{-}PX}$ = 261 Hz, $^{2}J_{PM\text{-}PY}$ = 104 Hz, $^{3}J_{PM\text{-}PA}$ = 27 Hz, 1 P, P^{M}), -148.4 (m, $^{1}J_{PX\text{-}PM}$ = 261 Hz, $^{1}J_{PX\text{-}PY}$ = 220 Hz, $^{2}J_{PX\text{-}PA}$ = 62 Hz, 1 P, P^{X}), -151.6 (m, $^{1}J_{PY\text{-}PX}$ = 220 Hz, $^{1}J_{PY\text{-}PA}$ = 207 Hz, $^{2}J_{PY\text{-}PM}$ = 104 Hz, 1 P, P^{Y})

Crystallographic Details

General Considerations

The crystallographic data for all synthesized compounds was collected either on an Xcalibur Gemini with an AtlasS2 detector using Cu-K radiation$_{\alpha}$ (**B1**), on a GV1000 diffractometer (AT) with a TitanS2 detector using Cu-K$_{\alpha}$ radiation (**A1**, **2**, **4**, **5**, **6a**, **8b**) or on a XtaLAB Synergy R, DW system with HyPix-Arc 150 detector applying Cu-Kα radiation (**A2**, **B2**, **1a**, **1a$_{INT}$**, **1c**, **1d**, **3a**, **3b**, **3c**, **3d**, **4$_{INT}$**, **7**, **8a**) from a rotating anode X-ray source. All measurements were performed at 123 K. Data collection, data reduction and absorption correction were performed with the CrysAlisPro software package.[8] Structure solution and refinement was conducted in Olex2 with ShelXT[9] and ShelXL[10] (full-matric least-squares method against F^2). All non-hydrogen atoms were refined with anisotropic displacement parameters and hydrogen atoms were treated as riding models with isotropic displacement parameters and fixed C-H bond lengths (sp^3: 0.96 (CH_3), 0.97 (CH_2), sp^2: 0.93 (CH)). Visualization of the crystal structures was performed with Olex2.[11]

CCDC entries 2421953-2421971, contain the supplementary crystallographic data for this paper. These data can be obtained free of charge at www.ccdc.cam.ac.uk/conts/retrieving.html (or from the Cambridge Crystallographic Data Centre, 12 Union Road, Cambridge CB2 1EZ, UK; Fax: + 44-1223-336-033; e-mail: deposit@ccdc.cam.ac.uk).

**Compound**	**A1**	**A2**	**B1**	**B2**
CCDC Entry	2421953	2421954	2421955	2421956
Empirical formula	$C_{40}H_{30}F_6Mo_2O_{10}P_8S_2$	$C_{118}H_{80}Al_4Cl_4F_{144}Mo_4O_{24}P_{16}$	$C_{29}H_{39}F_6P_5Ni$	$C_{23}H_{43}F_6NiP_5$
Formula weight	1288.40	5746.81	715.16	647.13
Temperature/K	123.15	123.15	293(2)	100.00(10)
Crystal system	triclinic	triclinic	monoclinic	monoclinic
Space group	$P\bar{1}$	$P\bar{1}$	$P2_1/n$	$P2_1/c$
a/Å	10.3127(2)	15.73390(10)	10.4531(5)	15.5378(2)
b/Å	10.7357(2)	19.6253(2)	24.9776(9)	13.9119(2)
c/Å	24.2951(4)	31.8836(3)	13.0061(6)	14.4028(2)
α/°	88.244(2)	82.0160(10)	90	90
β/°	84.235(2)	79.9250(10)	108.778(5)	104.226(2)
γ/°	64.221(2)	83.5890(10)	90	90
Volume/Å^3	2409.61(8)	9560.72(15)	3215.1(3)	3017.84(8)
Z	2	2	4	4
ρ_{calc}g/cm^3	1.776	1.996	1.477	1.424
μ/mm^{-1}	8.285	5.941	0.907	3.876
F(000)	1280.0	5608.0	1480.0	1352.0
Crystal size/mm^3	0.2 × 0.14 × 0.12	0.666 × 0.136 × 0.094	1.027 × 0.157 × 0.118	0.35 × 0.29 × 0.12
Radiation	Cu Kα (λ = 1.54184)	Cu Kα (λ = 1.54184)	Mo Kα (λ = 0.71073)	Cu Kα (λ = 1.54184)
2Θ range for data collection/°	7.316 to 133.542	4.566 to 147.476	6.564 to 64.9	5.868 to 143.734
Index ranges	-12 ≤ h ≤ 12, -12 ≤ k ≤ 12, -19 ≤ l ≤ 28	-19 ≤ h ≤ 19, -16 ≤ k ≤ 24, -39 ≤ l ≤ 39	-9 ≤ h ≤ 15, -37 ≤ k ≤ 35, -19 ≤ l ≤ 18	-18 ≤ h ≤ 18, -16 ≤ k ≤ 16, -17 ≤ l ≤ 16
Reflections collected	40387	130920	18134	41024
Independent reflections	8493 [R_{int} = 0.0813, R_{sigma} = 0.0462]	36813 [R_{int} = 0.0562, R_{sigma} = 0.0530]	10126 [R_{int} = 0.0222, R_{sigma} = 0.0466]	5810 [R_{int} = 0.0580, R_{sigma} = 0.0286]
Data/restraints/parameters	8493/605/844	36813/6611/4813	10126/0/376	5810/54/536
Goodness-of-fit on F^2	1.026	1.032	1.037	1.051
Final R indexes [I>=2σ (I)]	R_1 = 0.0573, wR_2 = 0.1523	R_1 = 0.0660, wR_2 = 0.1896	R_1 = 0.0386, wR_2 = 0.0760	R_1 = 0.0517, wR_2 = 0.1162
Final R indexes [all data]	R_1 = 0.0626, wR_2 = 0.1602	R_1 = 0.0832, wR_2 = 0.2026	R_1 = 0.0585, wR_2 = 0.0842	R_1 = 0.0586, wR_2 = 0.1195
Largest diff. peak/hole / e Å^{-3}	0.91/-1.37	1.59/-0.83	0.66/-0.33	0.56/-0.69

Compound	1a	$1a_{INT}$	1c	1d
CCDC Entry	2421957	/	2421958	2421959
Empirical formula	$C_{59}H_{59}F_7MoN_2O_5P_4S$	$C_{100}H_{106}F_8Mo_2N_4O_{10}P_8S_2$	$C_{56}H_{55}AlF_{36}MoN_2O_6P_4$	$C_{40}H_{39}N_2O_6F_{36}AlP_4Mo$
Formula weight	1260.96	2179.64	1782.82	1574.53
Temperature/K	123.15	123(2)	123.15	123.01(10)
Crystal system	triclinic	triclinic	monoclinic	triclinic
Space group	$P\bar{1}$	$P\bar{1}$	$P2_1/c$	$P\bar{1}$
a/Å	13.2210(2)	10.4767(3)	11.26790(10)	10.85720(10)
b/Å	14.2245(2)	22.1496(5)	17.93300(10)	16.6570(2)
c/Å	18.6479(3)	22.5564(7)	36.1886(3)	18.4604(2)
α/°	102.0130(10)	91.011(2)	90	113.4040(10)
β/°	96.9240(10)	99.963(2)	94.4380(10)	105.3990(10)
γ/°	112.378(2)	97.994(2)	90	92.0270(10)
Volume/Å^3	3093.49(9)	5100.6(2)	7290.60(10)	2916.20(6)
Z	2	-	4	2
ρ_{calc}g/cm^3	1.354	-	1.624	1.793
μ/mm^{-1}	3.607	-	3.704	4.531
F(000)	1296.0	-	3568.0	1560.0
Crystal size/mm^3	0.16 × 0.13 × 0.09	-	0.16 × 0.1 × 0.08	0.16 × 0.14 × 0.11
Radiation	Cu Kα (λ = 1.54184)	-	Cu Kα (λ = 1.54184)	Cu Kα (λ = 1.54184)
2Θ range for data collection/°	4.968 to 143.27	-	4.898 to 143.232	5.478 to 147.262
Index ranges	-16 ≤ h ≤ 16, -17 ≤ k ≤ 16, -22 ≤ l ≤ 22	-	-13 ≤ h ≤ 13, -20 ≤ k ≤ 22, -43 ≤ l ≤ 44	-11 ≤ h ≤ 13, -20 ≤ k ≤ 20, -22 ≤ l ≤ 22
Reflections collected	47984	-	46128	38567
Independent reflections	11777 [R_{int} = 0.0300, R_{sigma} = 0.0294]	-	13843 [R_{int} = 0.0279, R_{sigma} = 0.0304]	11262 [R_{int} = 0.0228, R_{sigma} = 0.0231]
Data/restraints/parameters	11777/589/847	-	13843/739/1194	11262/1532/1291
Goodness-of-fit on F^2	1.065	-	1.033	1.031
Final R indexes [I>=2σ (I)]	R_1 = 0.0433, wR_2 = 0.1314	-	R_1 = 0.0399, wR_2 = 0.1021	R_1 = 0.0290, wR_2 = 0.0732
Final R indexes [all data]	R1 = 0.0521, wR2 = 0.1374	-	R_1 = 0.0485, wR_2 = 0.1068	R_1 = 0.0317, wR_2 = 0.0747
Largest diff. peak/hole / e Å^{-3}	0.56/-0.53	-	0.46/-0.34	0.68/-0.39

**Compound**	**2**
CCDC Entry	2421960
Empirical formula	$C_{37.6}H_{46.4}MoN_2O_3P_4$
Formula weight	794.18
Temperature/K	159.15
Crystal system	triclinic
Space group	$P\bar{1}$
a/Å	12.1481(2)
b/Å	12.4558(2)
c/Å	14.9933(2)
α/°	86.9260(10)
β/°	81.9380(10)
γ/°	61.533(2)
Volume/Å^3	1974.46(6)
Z	2
ρ_{calc}g/cm^3	1.336
μ/mm^{-1}	4.537
F(000)	824.0
Crystal size/mm^3	0.22 × 0.18 × 0.12
Radiation	Cu Kα (λ = 1.54184)
2Θ range for data collection/°	8.076 to 133.39
Index ranges	-13 ≤ h ≤ 14, -14 ≤ k ≤ 14, -17 ≤ l ≤ 16
Reflections collected	34276
Independent reflections	6945 [R_{int} = 0.0478, R_{sigma} = 0.0253]
Data/restraints/parameters	6945/0/397
Goodness-of-fit on F^2	1.058
Final R indexes [I>=2σ (I)]	R1 = 0.0303, wR2 = 0.0842
Final R indexes [all data]	R_1 = 0.0309, wR_2 = 0.0847
Largest diff. peak/hole / e Å^{-3}	0.46/-0.80

Compound	**3a**	**3b**[OTf]	**3c**	**3d**
CCDC Entry	2421961	2421962	2421963	2421964
Empirical formula	$C_{112}H_{150}F_{12}N_4Ni_2P_{10}$	$C_{41}H_{59}N_2O_3F_3P_4SNi$	$C_{53}H_{86}F_6N_2NiP_5$	$C_{34}H_{63}N_2F_6P_5Ni$
Formula weight	2207.47	899.55	1078.79	827.42
Temperature/K	210(120)	123.00(10)	123.00(10)	123.01(10)
Crystal system	triclinic	orthorhombic	monoclinic	orthorhombic
Space group	$P\bar{1}$	*Pbca*	$P2_1/n$	$Pna2_1$
a/Å	17.5318(2)	15.77900(10)	14.48490(10)	22.7672(2)
b/Å	17.6671(2)	16.73710(10)	17.85970(10)	15.11330(10)
c/Å	19.0793(3)	34.2446(2)	23.26710(10)	26.6638(2)
α/°	81.9830(10)	90	90	90
β/°	86.0820(10)	90	105.0590(10)	90
γ/°	85.5260(10)	90	90	90
Volume/Å^3	5824.07(13)	9043.82(9)	5812.40(6)	9174.68(12)
Z	2	8	4	11
ρ_{calc}g/cm^3	1.259	1.321	1.233	1.647
μ/mm^{-1}	2.237	2.806	2.224	3.668
F(000)	2328.0	3792.0	2300.0	4818.0
Crystal size/mm^3	0.49 × 0.16 × 0.11	0.263 × 0.069 × 0.033	0.42 × 0.3 × 0.11	0.37 × 0.3 × 0.14
Radiation	Cu Kα (λ = 1.54184)	Cu Kα (λ = 1.54184)	Cu Kα (λ = 1.54184)	Cu Kα (λ = 1.54184)
2Θ range for data collection/°	5.064 to 148.204	5.162 to 147.328	6.322 to 143.24	6.63 to 146.216
Index ranges	-19 ≤ h ≤ 21, -22 ≤ k ≤ 21, -23 ≤ l ≤ 23	-19 ≤ h ≤ 14, -20 ≤ k ≤ 20, -42 ≤ l ≤ 41	-17 ≤ h ≤ 17, -20 ≤ k ≤ 21, -27 ≤ l ≤ 28	-21 ≤ h ≤ 27, -18 ≤ k ≤ 17, -26 ≤ l ≤ 32
Reflections collected	65155	146284	45552	44608
Independent reflections	22252 [R_{int} = 0.0184, R_{sigma} = 0.0171]	9105 [R_{int} = 0.0281, R_{sigma} = 0.0123]	10976 [R_{int} = 0.0224, R_{sigma} = 0.0171]	14134 [R_{int} = 0.0249, R_{sigma} = 0.0233]
Data/restraints/parameters	22252/833/1942	9105/0/511	10976/0/598	14134/216/1031
Goodness-of-fit on F^2	1.179	1.042	1.032	1.050
Final R indexes [I>=2σ (I)]	R_1 = 0.0434, wR_2 = 0.1001	R_1 = 0.0313, wR_2 = 0.0809	R_1 = 0.0334, wR_2 = 0.0885	R_1 = 0.0370, wR_2 = 0.1013
Final R indexes [all data]	R_1 = 0.0455, wR_2 = 0.1011	R_1 = 0.0342, wR_2 = 0.0826	R_1 = 0.0354, wR_2 = 0.0897	R_1 = 0.0394, wR_2 = 0.1030
Largest diff. peak/hole / e Å^{-3}	0.38/-0.28	0.87/-0.31	0.69/-0.42	0.50/-0.40

**Compound**	**4**	**4_{INT}**	**5**
CCDC Entry	2421965	2421966	2421967
Empirical formula	$C_{57}H_{94}N_3NiOP_4$	$C_{113}H_{189}N_4Ni_2O_2P_8$	$C_{59}H_{99}F_3N_2NiO_4P_4S$
Formula weight	1019.94	2000.85	1172.05
Temperature/K	123.00(10)	123.00(10)	123.00(10)
Crystal system	monoclinic	triclinic	monoclinic
Space group	$P2_1/c$	$P\bar{1}$	$P2_1/n$
a/Å	15.2032(2)	13.8094(2)	22.0586(2)
b/Å	38.7912(4)	20.4395(3)	13.41130(10)
c/Å	10.37670(10)	21.9281(4)	24.1491(2)
α/°	90	96.889(2)	90
β/°	107.8070(10)	95.901(2)	116.4940(10)
γ/°	90	105.303(2)	90
Volume/Å^3	5826.48(12)	5868.08(17)	6393.87(10)
Z	4	2	4
ρ_{calc}g/cm^3	1.163	1.132	1.218
μ/mm^{-1}	1.805	1.777	2.107
F(000)	2212.0	2174.0	2520.0
Crystal size/mm^3	0.61 × 0.22 × 0.11	0.11 × 0.04 × 0.02	0.36 × 0.33 × 0.17
Radiation	Cu Kα (λ = 1.54184)	Cu Kα (λ = 1.54184)	Cu Kα (λ = 1.54184)
2Θ range for data collection/°	7.62 to 133.878	4.536 to 143.37	7.758 to 133.568
Index ranges	-18 ≤ h ≤ 17, -46 ≤ k ≤ 45, -10 ≤ l ≤ 12	-16 ≤ h ≤ 16, -21 ≤ k ≤ 24, -27 ≤ l ≤ 26	-26 ≤ h ≤ 26, -15 ≤ k ≤ 15, -28 ≤ l ≤ 28
Reflections collected	57284	71952	67359
Independent reflections	10321 [R_{int} = 0.0518, R_{sigma} = 0.0263]	22138 [R_{int} = 0.0504, R_{sigma} = 0.0762]	11260 [R_{int} = 0.0583, R_{sigma} = 0.0290]
Data/restraints/parameters	10321/0/621	22138/114/1185	11260/832/900
Goodness-of-fit on F^2	1.040	1.028	1.032
Final R indexes [I>=2σ (I)]	R_1 = 0.0352, wR_2 = 0.0940	R_1 = 0.0588, wR_2 = 0.1394	R1 = 0.0450, wR2 = 0.1238
Final R indexes [all data]	R_1 = 0.0374, wR_2 = 0.0959	R_1 = 0.1087, wR_2 = 0.1589	R1 = 0.0482, wR2 = 0.1273
Largest diff. peak/hole / e Å^{-3}	0.51/-0.36	0.65/-0.56	0.36/-0.41

Compound	6a	7	8a	8b
CCDC Entry	2421968	2421969	2421970	2421971
Empirical formula	$C_{61.5}H_{82}Ni_2P_6$	$C_{46}H_{86}Ni_2P_4$	$C_{56}H_{75}N_2P_5Ni$	$C_{60.5}H_{84}AsN_3NiP_4$
Formula weight	1078.44	880.44	989.74	1110.81
Temperature/K	123.15	293(2)	123.01(10)	122.97(10)
Crystal system	monoclinic	triclinic	monoclinic	monoclinic
Space group	I2/a	$P\bar{1}$	$P2_1/c$	$C2/c$
a/Å	20.3249(6)	9.19120(10)	22.6390(5)	25.2732(3)
b/Å	10.6940(3)	10.7346(2)	10.4198(2)	10.53900(10)
c/Å	54.5604(15)	14.0769(2)	24.9039(5)	45.1650(5)
α/°	90	99.3150(10)	90	90
β/°	93.485(2)	104.2670(10)	111.938(2)	104.2430(10)
γ/°	90	110.4560(10)	90	90
Volume/Å^3	11837.0(6)	1213.22(3)	5449.3(2)	11660.1(2)
Z	8	1	4	8
ρ_{calc}g/cm^3	1.210	1.205	1.206	1.266
μ/mm^{-1}	2.573	2.409	2.172	2.441
F(000)	4576.0	478.0	2112.0	4712.0
Crystal size/mm^3	0.132 × 0.055 × 0.038	0.3 × 0.08 × 0.06	0.17 × 0.03 × 0.03	0.39 × 0.12 × 0.05
Radiation	CuKα (λ = 1.54184)	Cu Kα (λ = 1.54184)	Cu Kα (λ = 1.54184)	Cu Kα (λ = 1.54184)
2Θ range for data collection/°	8.426 to 134.168	6.736 to 143.152	7.226 to 148.582	7.352 to 134.156
Index ranges	-24 ≤ h ≤ 24, -12 ≤ k ≤ 12, -63 ≤ l ≤ 65	-10 ≤ h ≤ 11, -13 ≤ k ≤ 12, -16 ≤ l ≤ 17	-27 ≤ h ≤ 25, -12 ≤ k ≤ 12, -29 ≤ l ≤ 30	-30 ≤ h ≤ 29, -12 ≤ k ≤ 11, -53 ≤ l ≤ 53
Reflections collected	115687	14164	33659	49384
Independent reflections	10497 [R_{int} = 0.1583, R_{sigma} = 0.0699]	4540 [R_{int} = 0.0191, R_{sigma} = 0.0175]	10310 [R_{int} = 0.0515, R_{sigma} = 0.0538]	10287 [R_{int} = 0.0486, R_{sigma} = 0.0267]
Data/restraints/parameters	10497/78/675	4540/0/248	10310/0/594	10287/271/709
Goodness-of-fit on F^2	1.017	1.051	1.067	1.043
Final R indexes [I>=2σ (I)]	R_1 = 0.0489, wR_2 = 0.1108	R_1 = 0.0234, wR_2 = 0.0609	R_1 = 0.0609, wR_2 = 0.1677	R_1 = 0.0346, wR_2 = 0.0922
Final R indexes [all data]	R_1 = 0.0784, wR_2 = 0.1252	R_1 = 0.0242, wR_2 = 0.0613	R_1 = 0.0789, wR_2 = 0.1813	R_1 = 0.0367, wR_2 = 0.0939
Largest diff. peak/hole / e Å^{-3}	0.40/-0.28	0.30/-0.23	1.26/-0.78	0.46/-0.41

$[CpMo(CO)_2(\eta^3\text{-}P_4Ph_2)][OTf]$ (**A1**)

A1 crystallizes as light yellow blocks from concentrated solutions in *o*-DFB layered with *n*-hexane and upon storage at room temperature for several days (Figure S1). **A1** crystallizes in the triclinic space group $P\overline{1}$ with two anions and two cations in the asymmetric unit. Disorders within the anions and the C_6H_5 group of the cation were treated with adequate restraints.

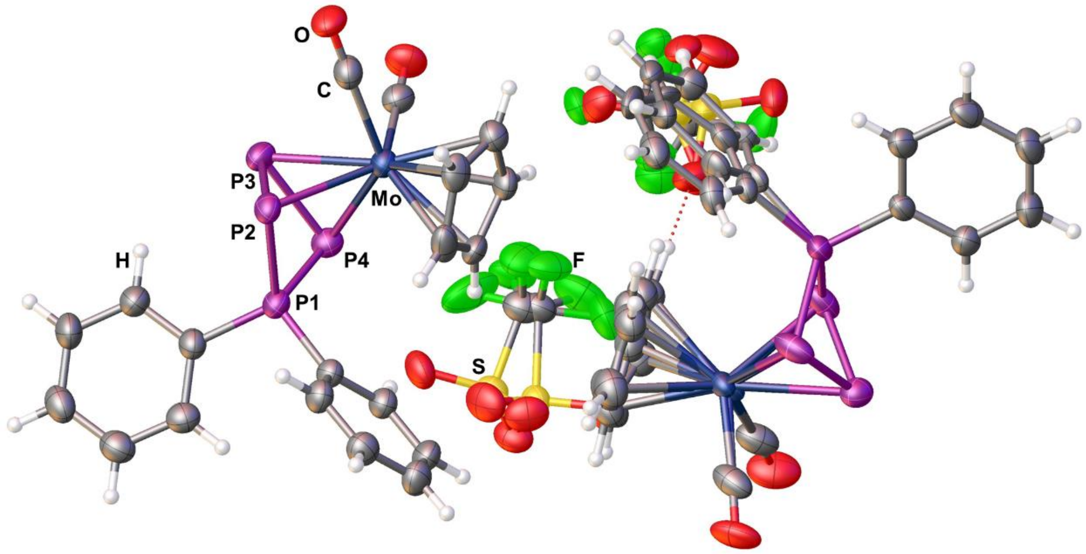

*Figure S 1: Solid state structure of **A1**. Depicted is the asymmetric unit and ADPs (anisotropic displacement parameters) are drawn at 50 % probability.*

[CpMo(CO)$_2$(η^3-P$_4{}^i$Pr$_2$)][TEF] (**A2**)

A2 crystallizes as light yellow sticks from concentrated solutions in CH_2Cl_2 layered with *n*-hexane and upon storage at room temperature for several days (Figure S2). **A2** crystallizes in the triclinic space group $P\bar{1}$ with four anions, four cations and two molecules CH_2Cl_2 in the asymmetric unit. Disorders within all four cations as well as anions were treated with adequate restraints.

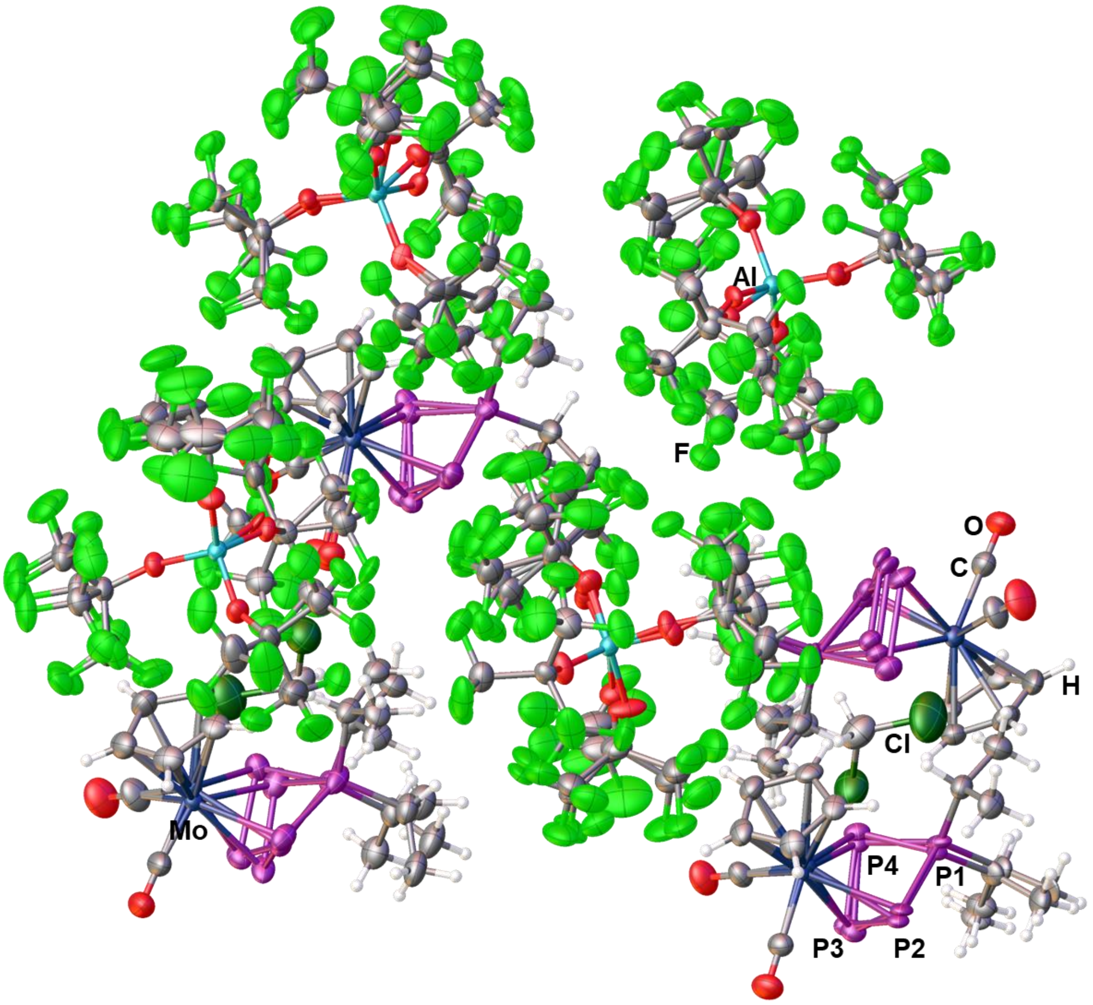

*Figure S 2: Solid state structure of **A2**. Depicted is the asymmetric unit and ADPs (anisotropic displacement parameters) are drawn at 50 % probability.*

[Cp'''Ni(η^3-P_4Ph_2)][PF_6] (**B1**)

B1 crystallizes as red blocks from concentrated solutions in *o*-DFB layered with *n*-hexane and upon storage at room temperature for several days (Figure S3). **B1** crystallizes in the monoclinic space group $P2_1/n$ with one anion and one cation in the asymmetric unit.

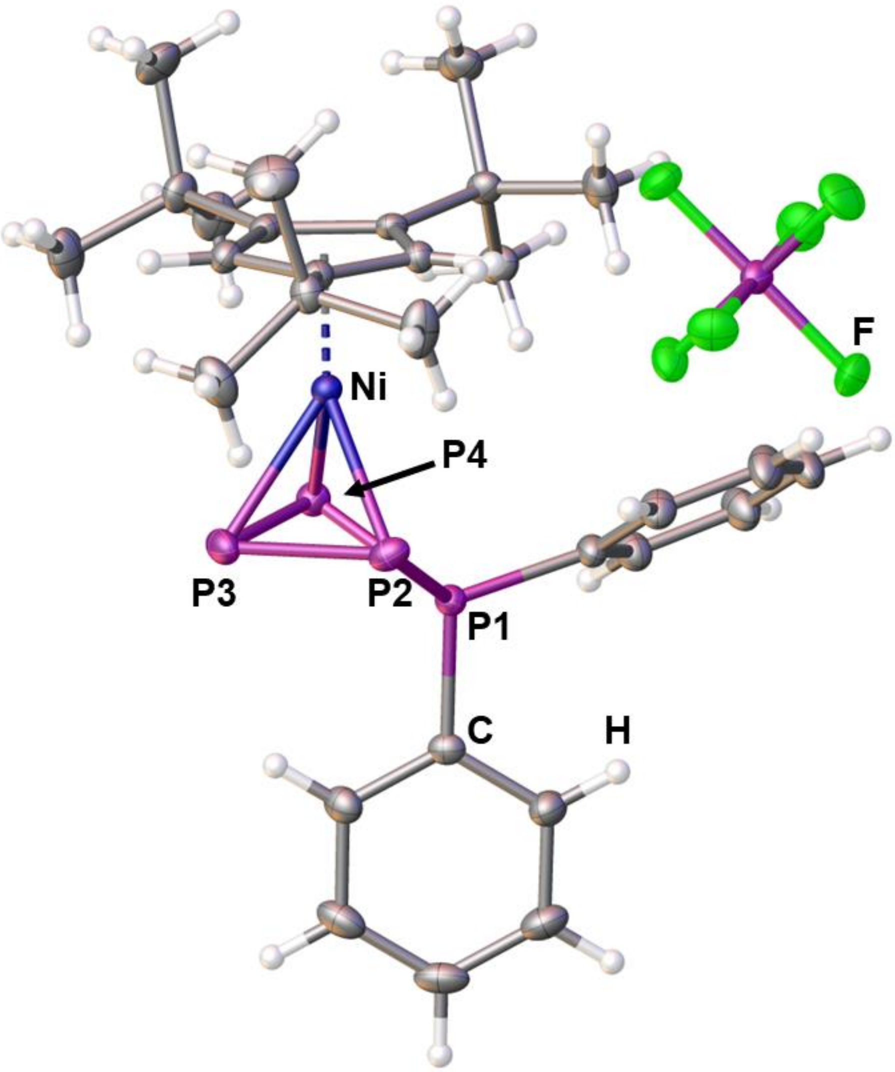

*Figure S 3: Solid state structure of **B1**. Depicted is the asymmetric unit and ADPs (anisotropic displacement parameters) are drawn at 50 % probability.*

[Cp'''Ni(η^3-$P_4{}^i Pr_2$)][PF_6] (**B2**)

B2 crystallizes as dark red plates from concentrated solutions in *o*-DFB layered with *n*-hexane and upon storage at room temperature for several days (Figure S4). **B2** crystallizes in the monoclinic space group $P2_1/n$ with one anion and one cation in the asymmetric unit. Disorder was treated with adequate restraints.

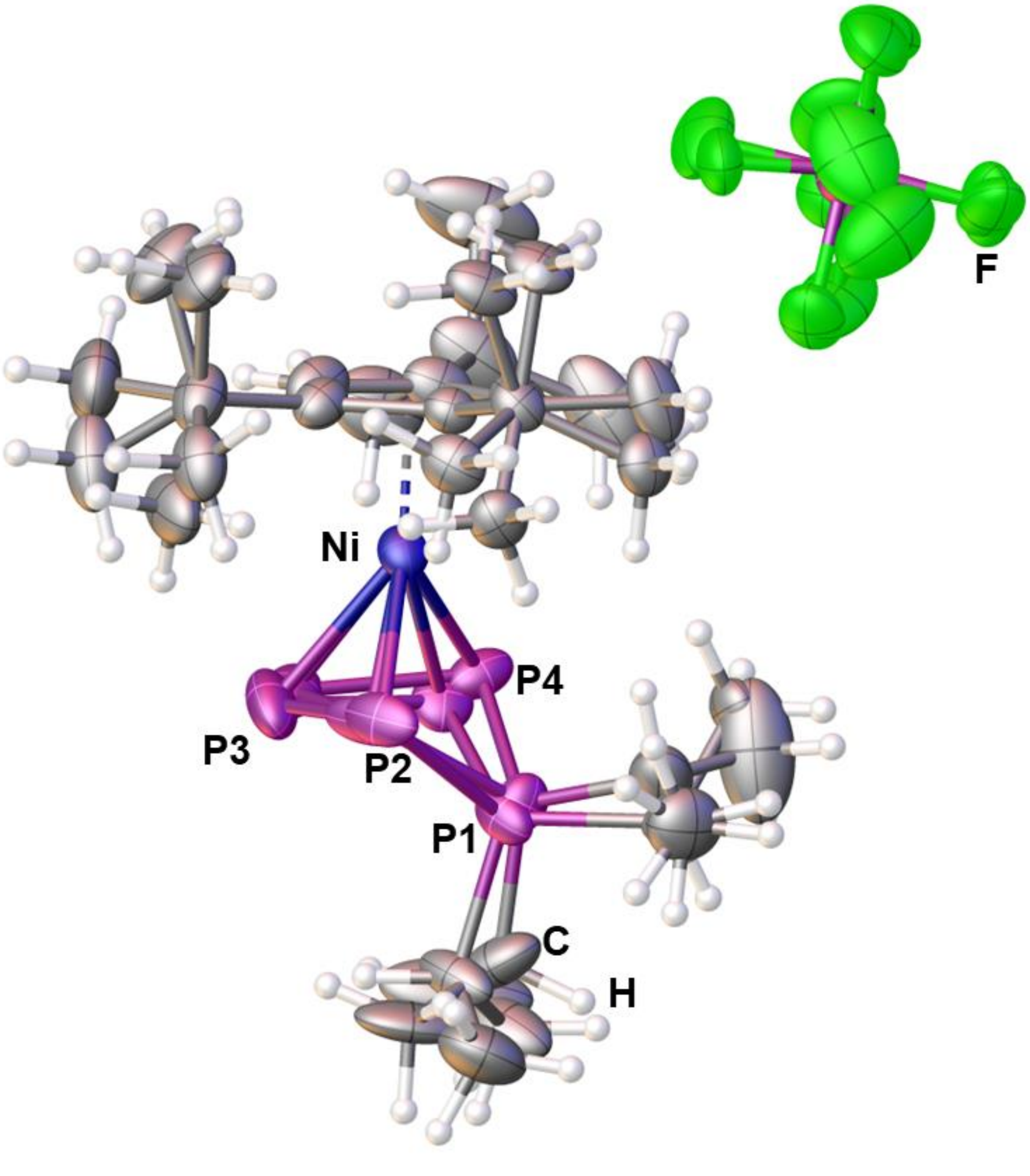

*Figure S 4: Solid state structure of **B2**. Depicted is the asymmetric unit and ADPs (anisotropic displacement parameters) are drawn at 50 % probability.*

[CpMo(CO)$_2$(η^3-P$_4$Ph$_2$IDipp)][OTf] (**1a**)

1a crystallizes as light yellow plates from concentrated solutions in *o*-DFB layered with *n*-hexane and upon storage at room temperature for several days (Figure S5). **1a** crystallizes in the triclinic space group $P\bar{1}$ with one anion, one cation and two molecules *o*-DFB in the asymmetric unit. Disorder was treated with adequate restraints. The *o*-DFB was treated with a solvent mask.

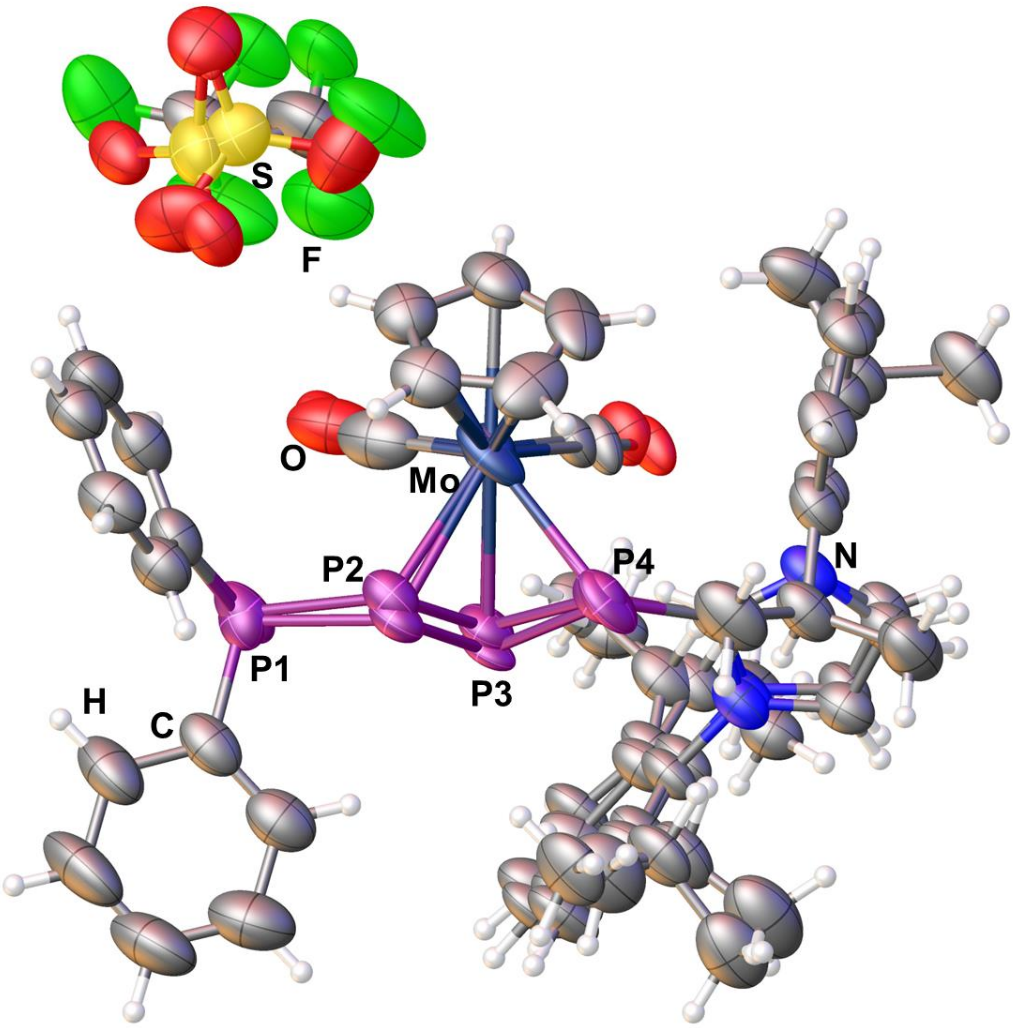

*Figure S 5: Solid state structure of **1a**. Depicted is the asymmetric unit and ADPs (anisotropic displacement parameters) are drawn at 50 % probability.*

[CpMo(CO)$_2$(η^3-P$_4$Ph$_2$IDipp)][OTf] (**1a$_{INT}$**)

1a$_{INT}$ must be handled with great care as interconverts to **1a** at room temperature or within time. **1a$_{INT}$** crystallizes as yellow sticks via gas diffusion from a concentrated *n*-hexane solution into toluene at -30 °C (Figure S6). It crystallizes in the triclinic space group $P\bar{1}$ with two cations, two anions and one *o*-DFB molecule and in the asymmetric unit. Mounting crystals of **1a$_{INT}$** had to be performed at low temperatures and with great care, no publishable measurement could be performed, since **1a$_{INT}$** occurred as lots of tiny plates stick together as one stick. Even twin refinement did not allow to obtain a publishable structure. Nevertheless, already the structure solution gives a clear picture of the composition of **1a$_{INT}$** in the solid state. (Figure S6)

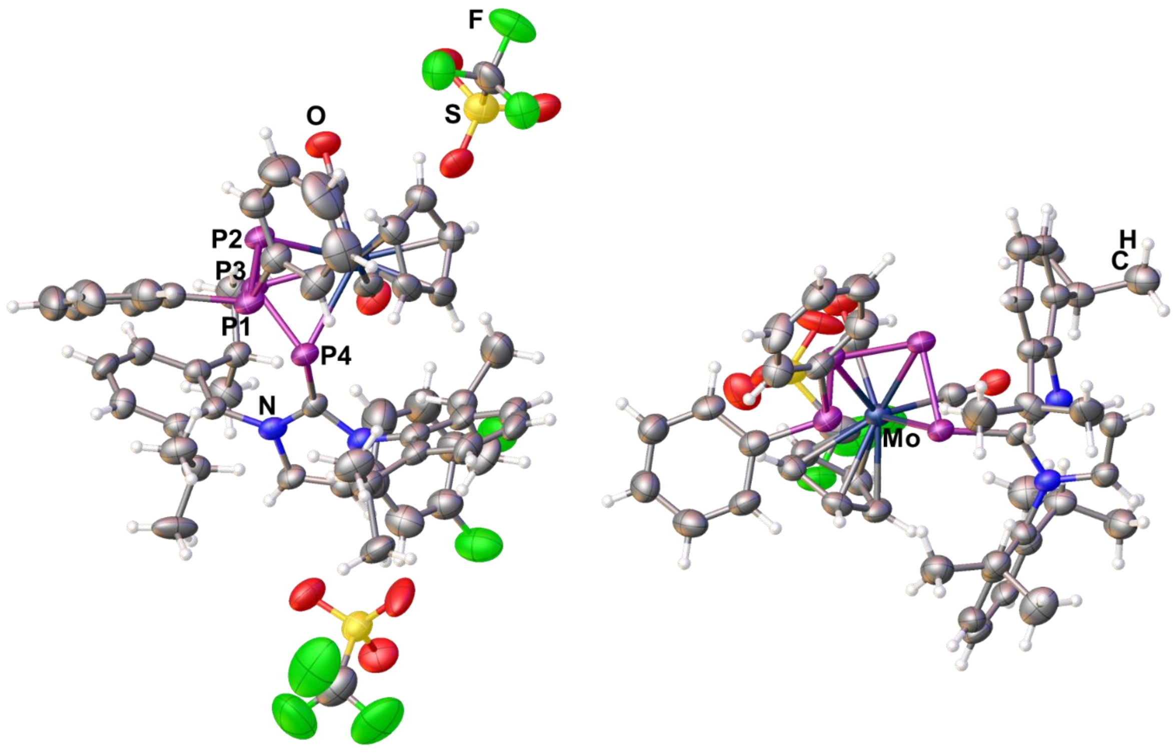

*Figure S 6: Solid state structure of **1a$_{INT}$**. Depicted is the asymmetric unit and ADPs (anisotropic displacement parameters) are drawn at 50 % probability.*

[CpMo(CO)$_2$(η^3-P$_4$^{i}Pr$_2$IDipp)][TEF] (**1c**)

1c crystallizes as light yellow plates from concentrated solutions in *o*-DFB layered with *n*-hexane and upon storage at room temperature for several days (Figure S7). **1c** crystallizes in the monoclinic space group *P*2$_1$/*c* with one anion and one cation in the asymmetric unit. Disorder was treated with adequate restraints.

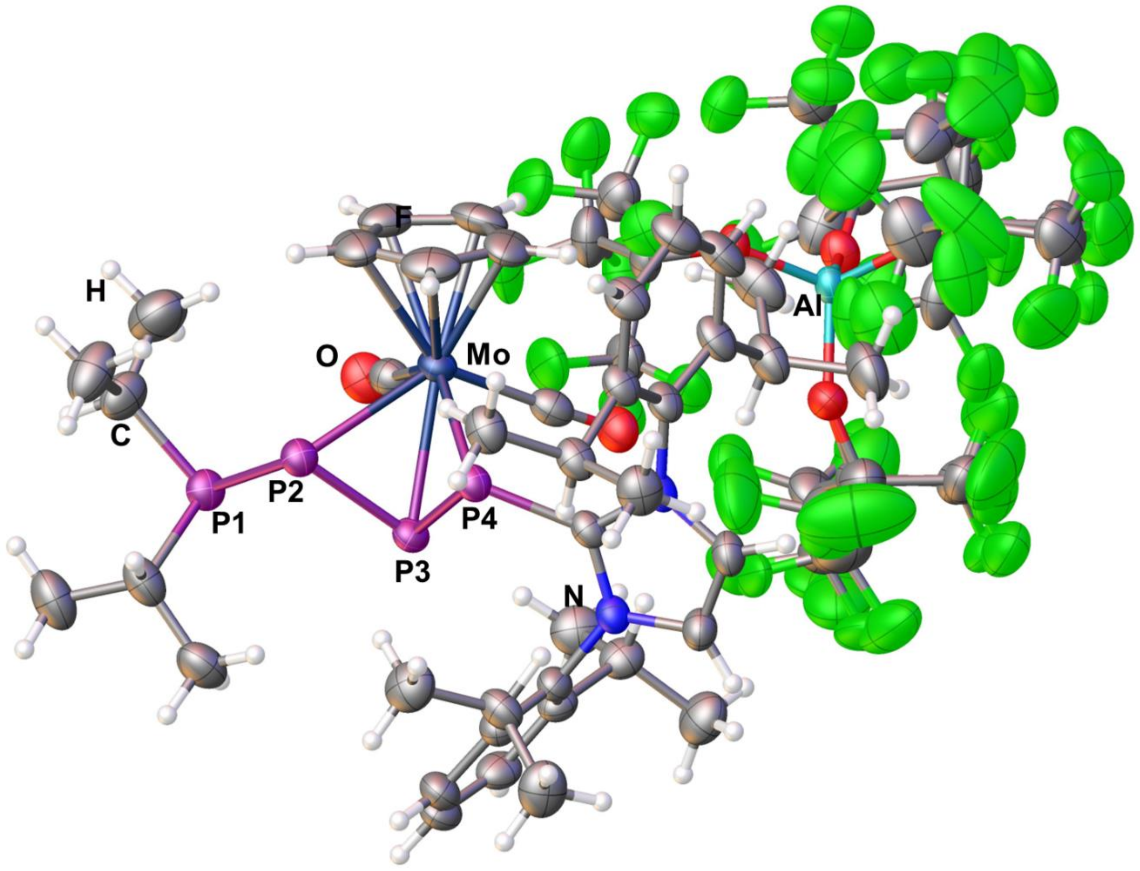

*Figure S 7: Solid state structure of **1c**. Depicted is the asymmetric unit and ADPs (anisotropic displacement parameters) are drawn at 50 % probability.*

[CpMo(CO)$_2$(η^3-P$_4$^{i}Pr$_2$I^{i}Pr$_2$Me$_2$)][TEF] (**1d**)

1d crystallizes as light yellow plates from concentrated solutions in *o*-DFB layered with *n*-hexane and upon storage at room temperature for several days (Figure S8). **1d** crystallizes in the triclinic space group $P\bar{1}$ with one anion and one cation in the asymmetric unit. Disorder within the anion was treated with adequate restraints.

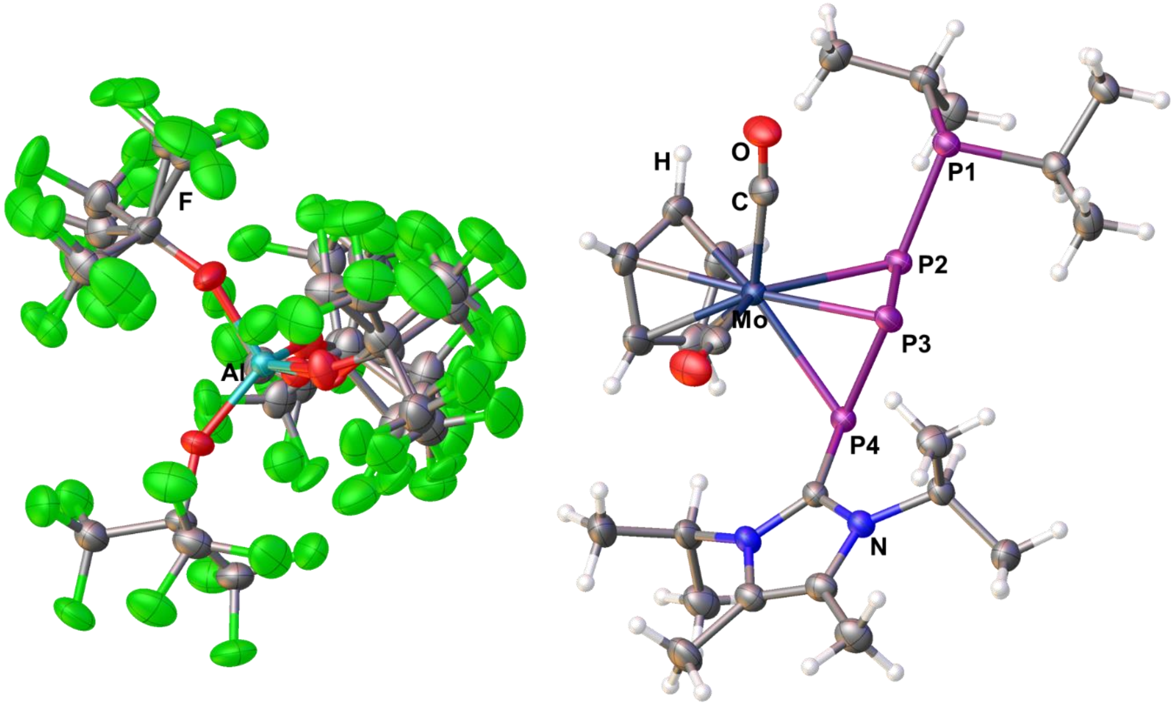

*Figure S 8: Solid state structure of **1d**. Depicted is the asymmetric unit and ADPs (anisotropic displacement parameters) are drawn at 50 % probability.*

[CpMo(CO)$_2$($\eta^{1:1}$-I^{i}Pr$_2$Me$_2$PPP(OEt)PPh$_2$)] (**2**)

2 crystallizes as intense orange blocks from concentrated toluene solutions and storage at 4 °C for several days (Figure S9). **2** crystallizes in the triclinic space group *P*$\bar{1}$ with one molecule and 0.8 molecules of toluene in the asymmetric unit. The toluene was treated with a solvent mask. Disorders within the OEt group was treated with adequate restraints.

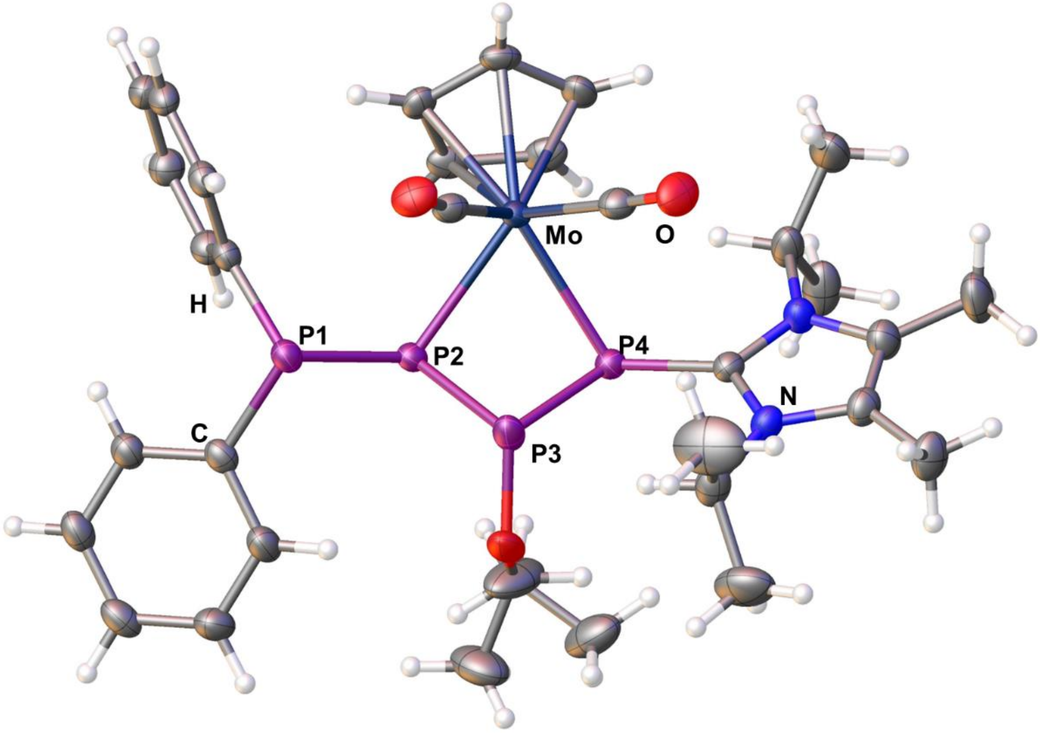

*Figure S 9: Solid state structure of **2**. Depicted is the asymmetric unit and ADPs (anisotropic displacement parameters) are drawn at 50 % probability.*

[Cp'''Ni($\eta^{1:1}$-P_4Ph_2IDipp)][PF_6] (**3a**)

3a crystallizes as dark greenish brown sticks from concentrated solutions in THF layered with *n*-hexane and upon storage at room temperature for several days (Figure S10). **3a** crystallizes in the monoclinic space group $P2_1/n$ with one anion and one cation in the asymmetric unit. Disorder was treated with adequate restraints.

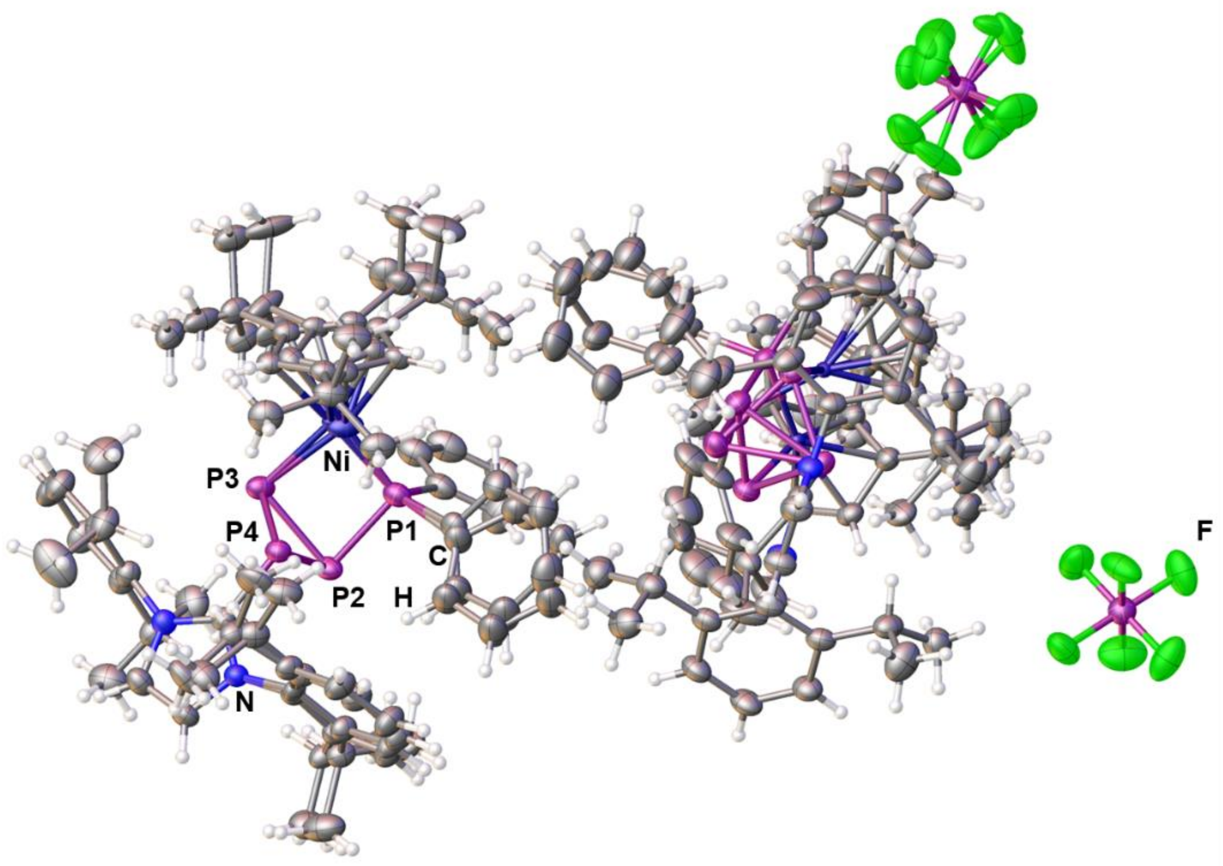

*Figure S 10: Solid state structure of **3a**. Depicted is the asymmetric unit and ADPs (anisotropic displacement parameters) are drawn at 50 % probability.*

[Cp'''Ni($\eta^{1:1}$-$P_4Ph_2I^iPr_2Me_2$)][OTf] (**3b**[OTf])

3b crystallizes from concentrated solutions in THF layered with *n*-hexane and upon storage at room temperature for several days. **3b** crystallizes in the orthorhombic space group *Pbcn* with one anion, one cation and one molecule of *n*-hexane in the asymmetric unit. However, the crystal quality of **3b** could not be improved to a satisfactory limit. Thus, the $[PF_6]^-$ anion was exchanged for $[OTf]^-$. **3b**[OTf] also crystallizes from concentrated THF solutions layered with *n*-hexane and storage at room temperature forming brownish sticks (Figure S11). However, it crystallizes in the orthorhombic space group *Pbca* with only an anion and a cation being present in the asymmetric unit.

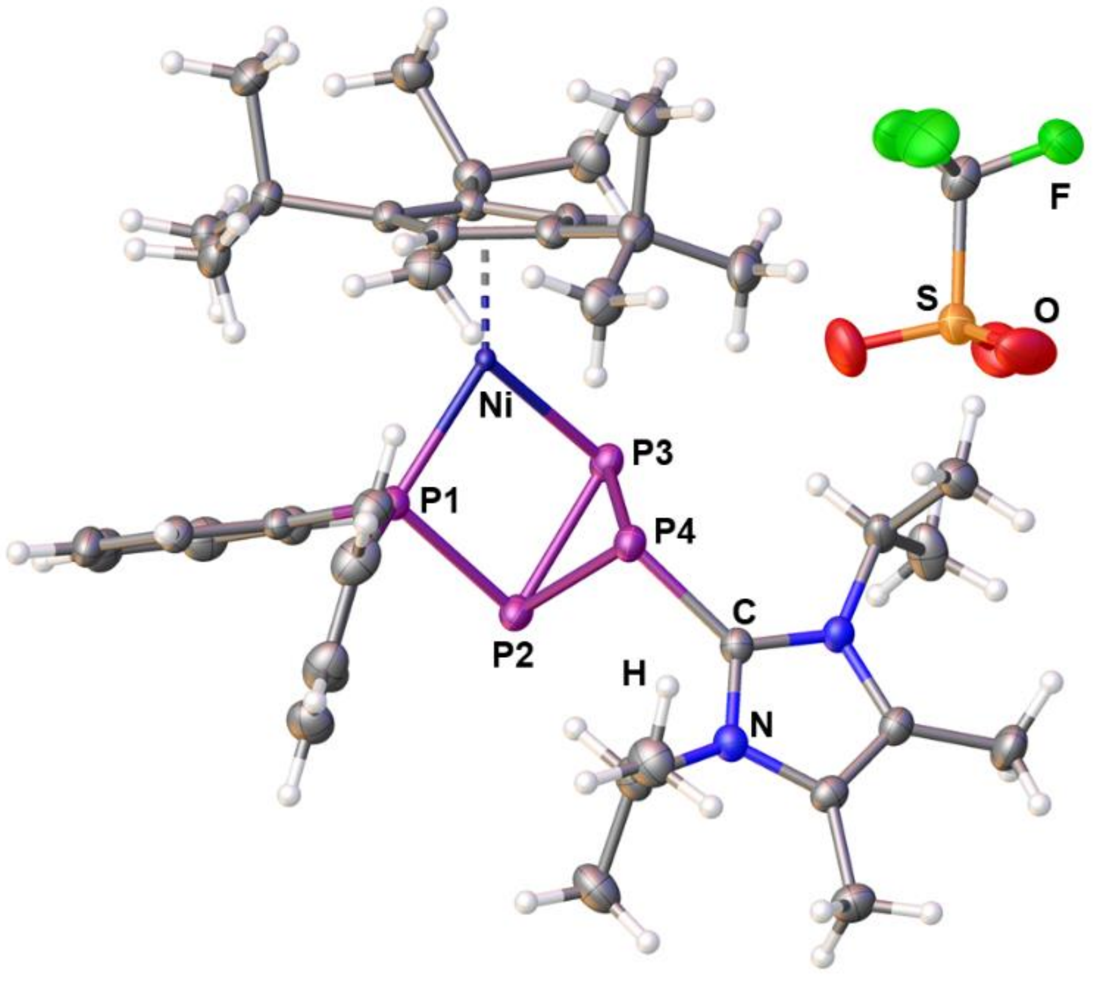

*Figure S 11: Solid state structure of **3b**[OTf]. Depicted is the asymmetric unit and ADPs (anisotropic displacement parameters) are drawn at 50 % probability.*

[Cp'''Ni($\eta^{1:1}$-$P_4{}^{i}Pr_2$IDipp)][PF_6] (**3c**)

3c crystallizes as greenish plates from concentrated solutions in *o*-DFB layered with *n*-hexane and upon storage at room temperature for several days (Figure S12). **3c** crystallizes in the monoclinic space group $P2_1/n$ with one anion and one cation in the asymmetric unit.

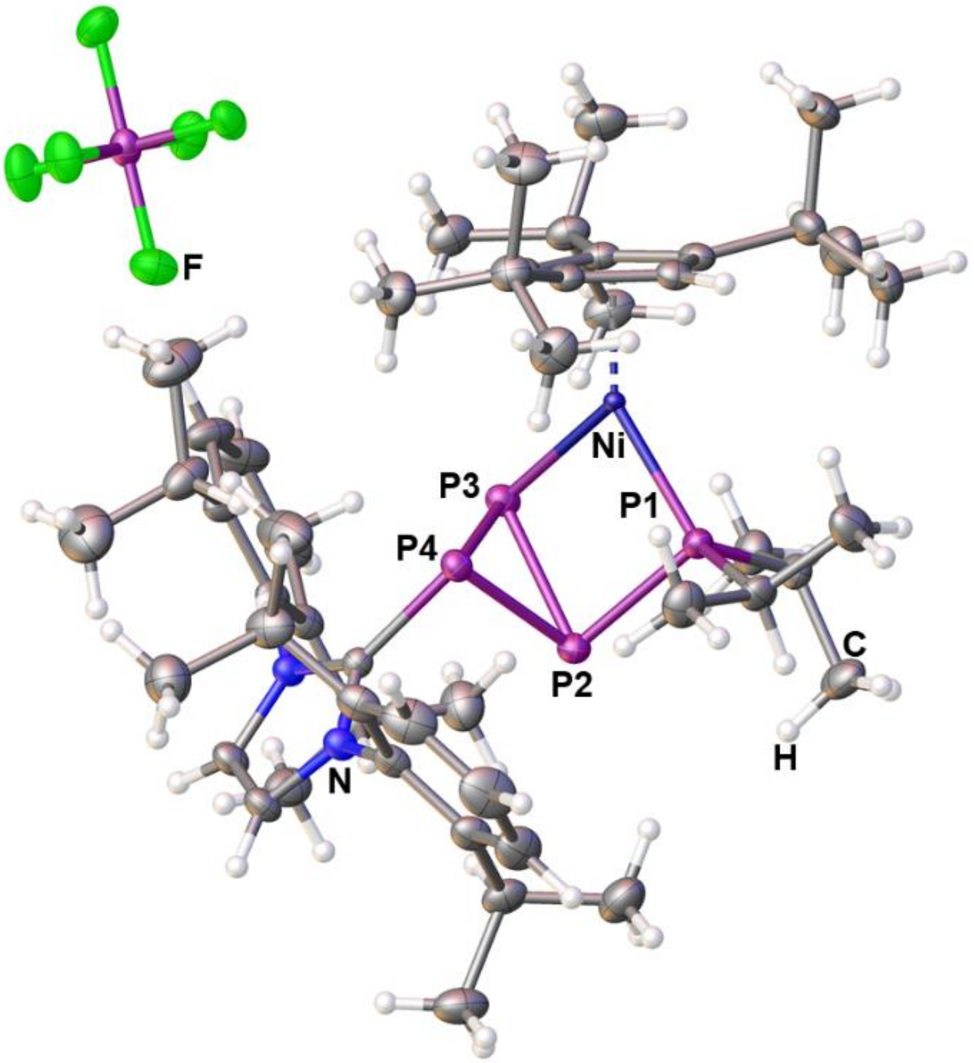

*Figure S 12: Solid state structure of **3c**. Depicted is the asymmetric unit and ADPs (anisotropic displacement parameters) are drawn at 50 % probability.*

[Cp'''Ni($\eta^{1:1}$-$P_4{}^iPr_2I^iPr_2Me_2$)][PF_6] (**3d**)

3d crystallizes as greenish brown plates from concentrated solutions in *o*-DFB layered with *n*-hexane and upon storage at room temperature for several days (Figure S13). **3d** crystallizes in the orthorhombic space group *Pna*2_1 with two anions, two cations and one molecule of *n*-hexane in the asymmetric unit. Disorder was treated with adequate restraints and the *n*-hexane was treated with a solvent mask.

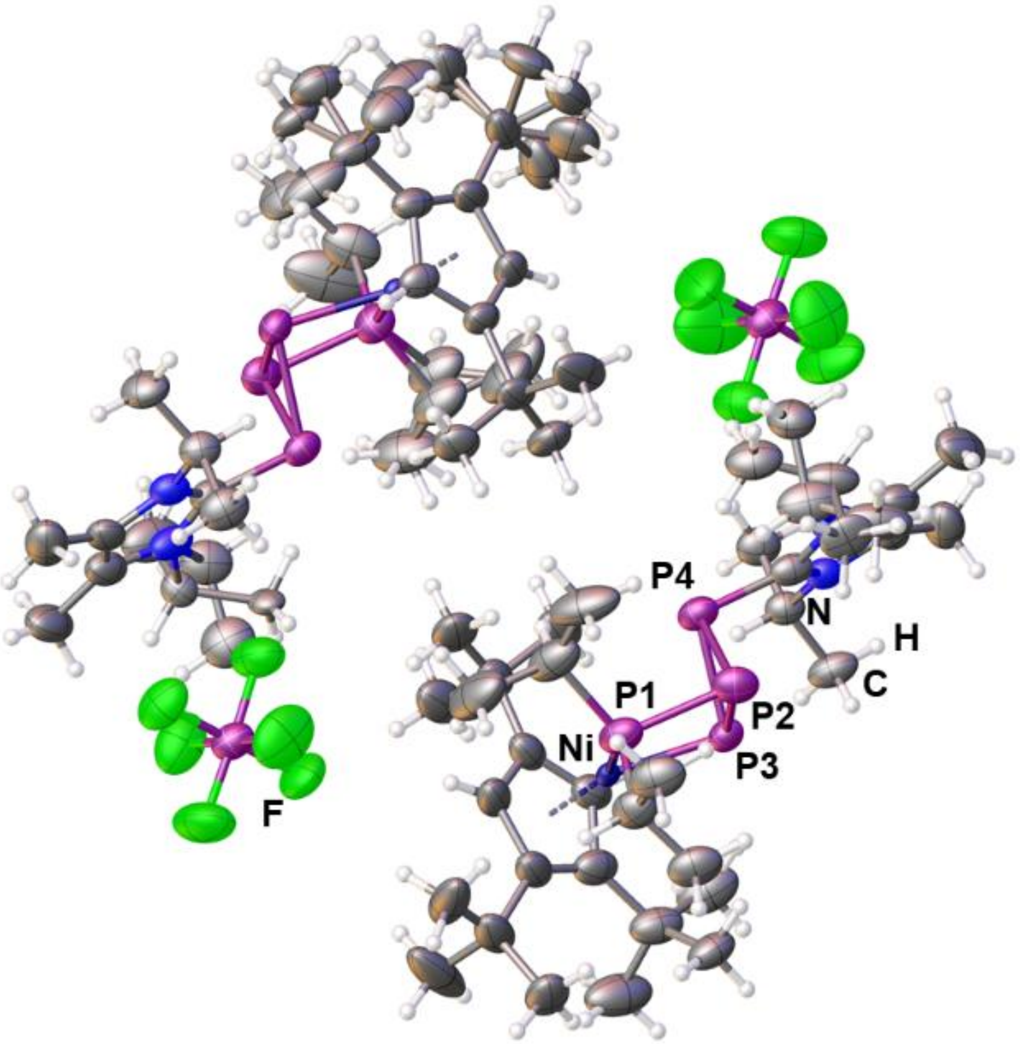

Figure S 13: Solid state structure of ***3d****. Depicted is the asymmetric unit and ADPs (anisotropic displacement parameters) are drawn at 50 % probability.*

[Cp'''Ni(η^2-IDippPP(OEt)PP^{i}Pr$_2$)] (**4**)

4 crystallizes as dark yellowish brown sticks from concentrated toluene solutions layered with acetonitrile and storage at 4 °C for several days (Figure S14). **4** crystallizes in the monoclinic space group $P2_1/c$ with one molecule, one acetonitrile and half of a molecule of *n*-hexane in the asymmetric unit. Disorder was treated with adequate restraints and the *n*-hexane was treated with a solvent mask.

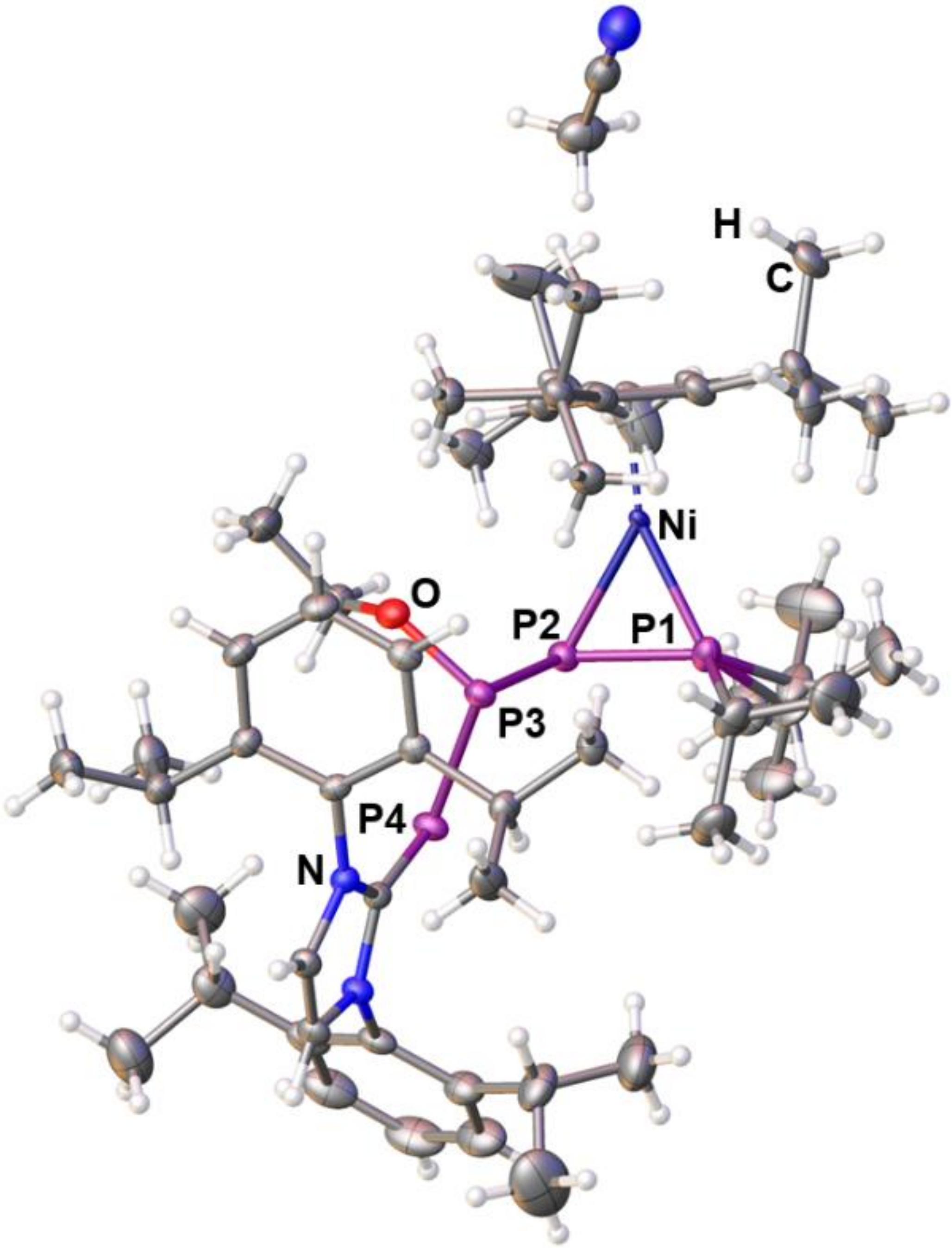

*Figure S 14: Solid state structure of **4**. Depicted is the asymmetric unit and ADPs (anisotropic displacement parameters) are drawn at 50 % probability.*

[Cp'''Ni($\eta^{1:1}$-IDippPPP(OEt)P^{i}Pr$_2$)] (**4**$_{\mathbf{INT}}$)

4$_{\mathbf{INT}}$ must be handled with great care as it decomposes at temperatures higher than -10 °C. **4**$_{\mathbf{INT}}$ crystallizes as dark turquoise platelets via inverse gas diffusion from a concentrated *n*-hexane solution into toluene at -30 °C (Figure S1**5**). It crystallizes in the triclinic space group $P\bar{1}$ with two molecules and 1.5 *n*-hexane molecules in the asymmetric unit. Disorder was treated with adequate restraints and the *n*-hexane was treated with a solvent mask.

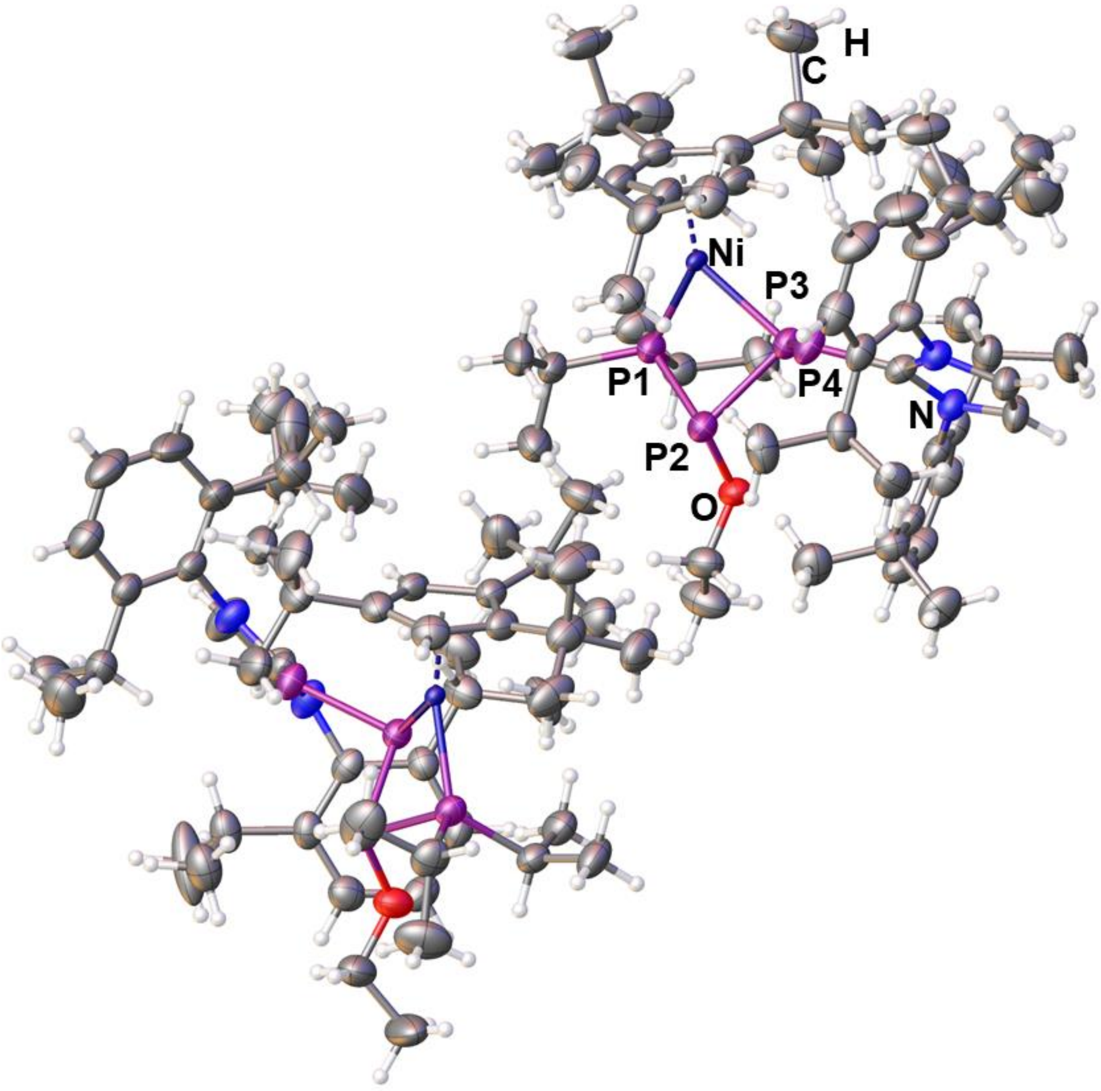

*Figure S 15: Solid state structure of **4**$_{\mathbf{INT}}$. Depicted is the asymmetric unit and ADPs (anisotropic displacement parameters) are drawn at 50 % probability.*

[Cp'''Ni($\eta^{1:1}$-MeP(OEt)P(IDippP)P^{i}Pr$_2$)] (**5**)

5 crystallizes as dark red to purple plates from concentrated CH_2Cl_2 solutions layered with *n*-pentane and storage at room temperature for several days (Figure S16). **5** crystallizes in the monoclinic space group *P*2$_1$/*n* with one cation, one anion and one molecule of *n*-pentane in the asymmetric unit. Disorder was treated with adequate restraints and the *n*-pentane was treated with a solvent mask.

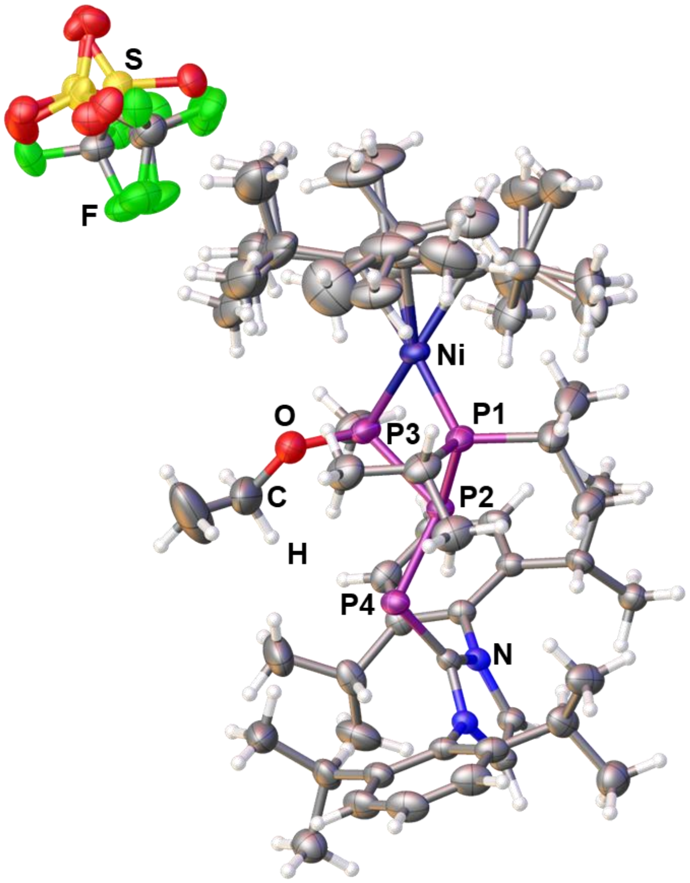

*Figure S 16: Solid state structure of **5**. Depicted is the asymmetric unit and ADPs (anisotropic displacement parameters) are drawn at 50 % probability.*

[{Cp'''Ni}$_2$(μ,η$^{1:1:1:1}$-*cyclo*-P$_4$(PPh$_2$)$_2$)] (**6a**)

6a crystallizes as dark brownish red sticks from concentrated toluene solutions layered with acetonitrile and storage at 4 °C for several days (Figure S17). 6a crystallizes in the monoclinic space group *I*2/*a* with one molecule in the asymmetric unit. Disorder was treated with adequate restraints.

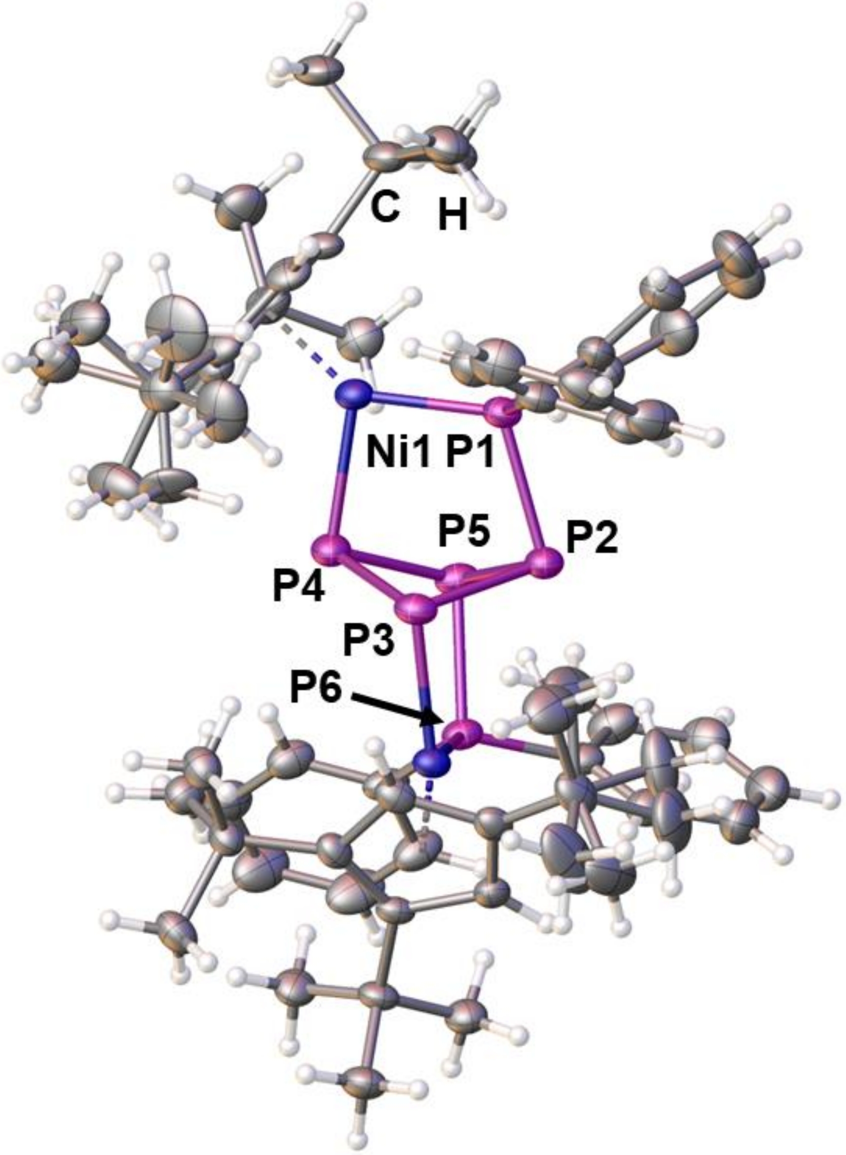

*Figure S 17: Solid state structure of **6a**. Depicted is the asymmetric unit and ADPs (anisotropic displacement parameters) are drawn at 50 % probability.*

[{Cp'''Ni}$_2$(μ,$\eta^{2:2}$-(PP^{i}Pr$_2$)$_2$)] (**7**)

7 crystallizes as clear red sticks by storing a concentrated solution in *n*-hexane at 4°C for 2 days (Figure S18). It crystallizes in the triclinic space group $P\overline{1}$ with half of a molecule in the asymmetric unit.

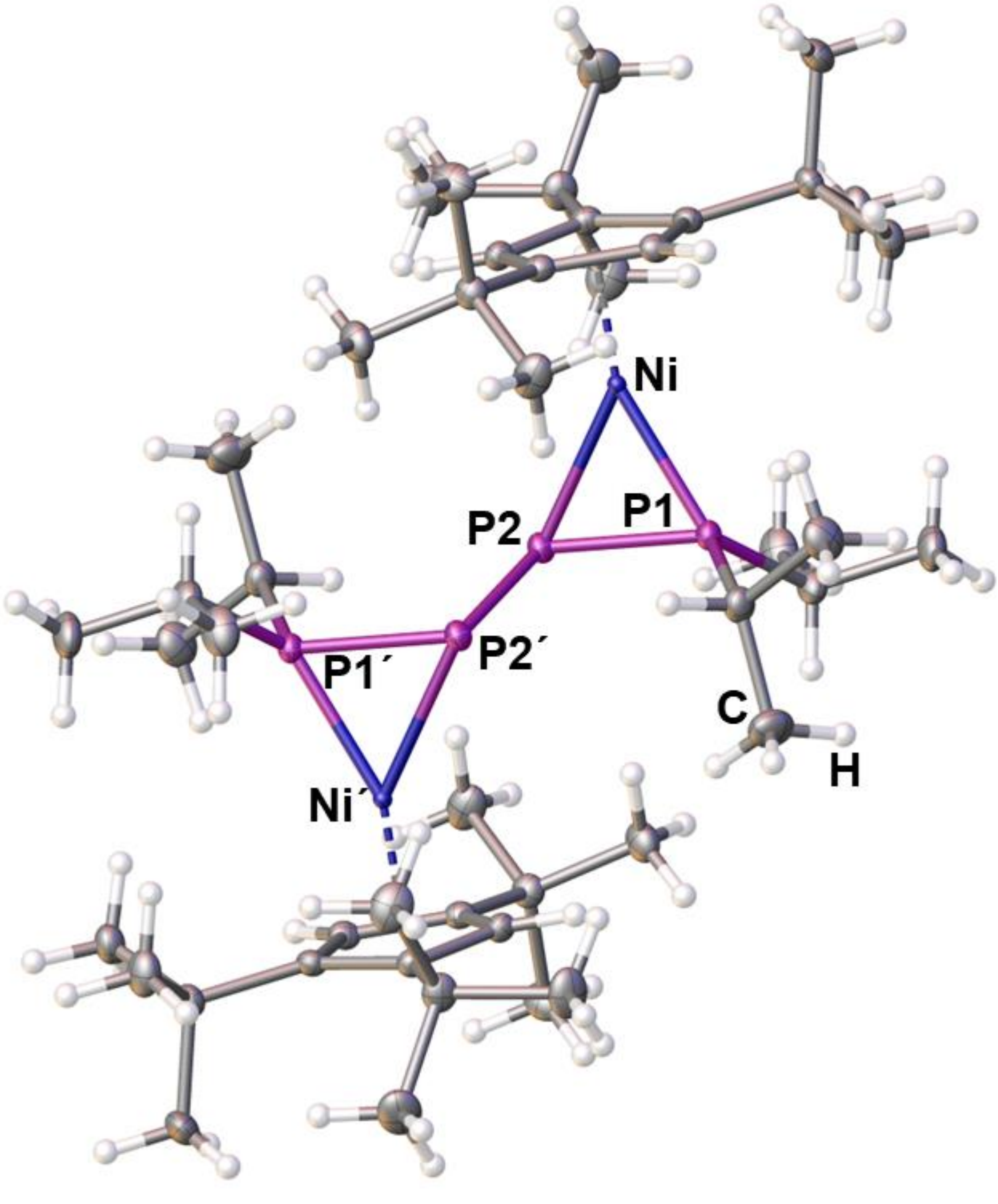

*Figure S 18: Solid state structure of **7**. Depicted is the asymmetric unit and its symmetry equivalent, and ADPs (anisotropic displacement parameters) are drawn at 50 % probability.*

[Cp'''Ni($\eta^{1:1}$-P_5Ph_2IDipp)] (**8a**)

8a crystallizes as yellow needles via inverse gas diffusion from a concentrated *n*-hexane solution into toluene at room temperature (Figure S19). It crystallizes in the monoclinic space group $P2_1/c$ with one molecule in the asymmetric unit.

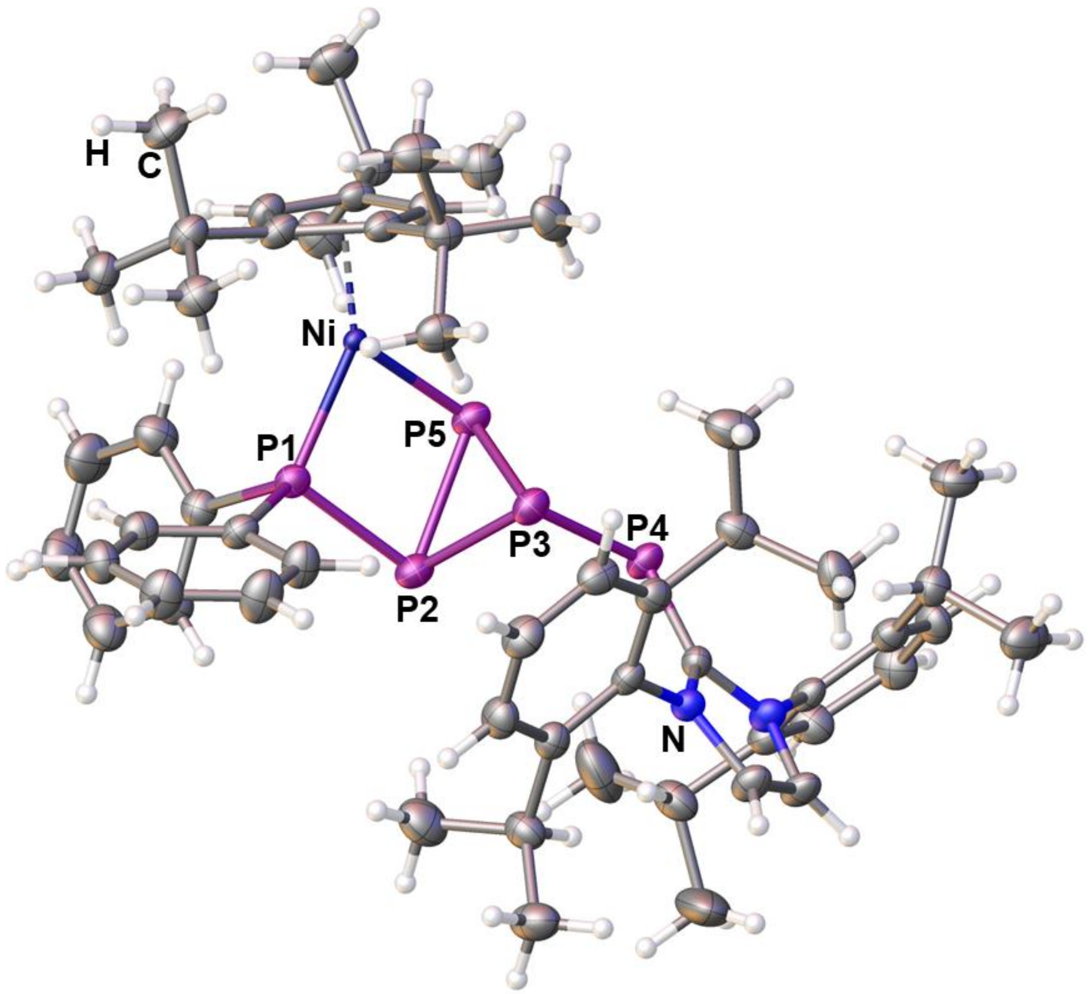

*Figure S 19: Solid state structure of **8a**. Depicted is the asymmetric unit and ADPs (anisotropic displacement parameters) are drawn at 50 % probability.*

[Cp'''Ni($\eta^{1:1}$-As$P_4$$Ph_2$IDipp)] (**8b**)

8b crystallizes as dark red needles from concentrated Et_2O solutions layered with acetonitrile and storage at -30 °C for several days (Figure S20). **8b** crystallizes in the monoclinic space group *C*2/*c* with one molecule, half of a molecule of *n*-hexane and one acetonitrile molecule in the asymmetric unit. Disorder was treated with adequate restraints. Notably, the As atom is disordered across all three positions within the three membered ring. However, it occupies the unsubstituted position to 87%, while the others are equally occupied to 6/7%, each.

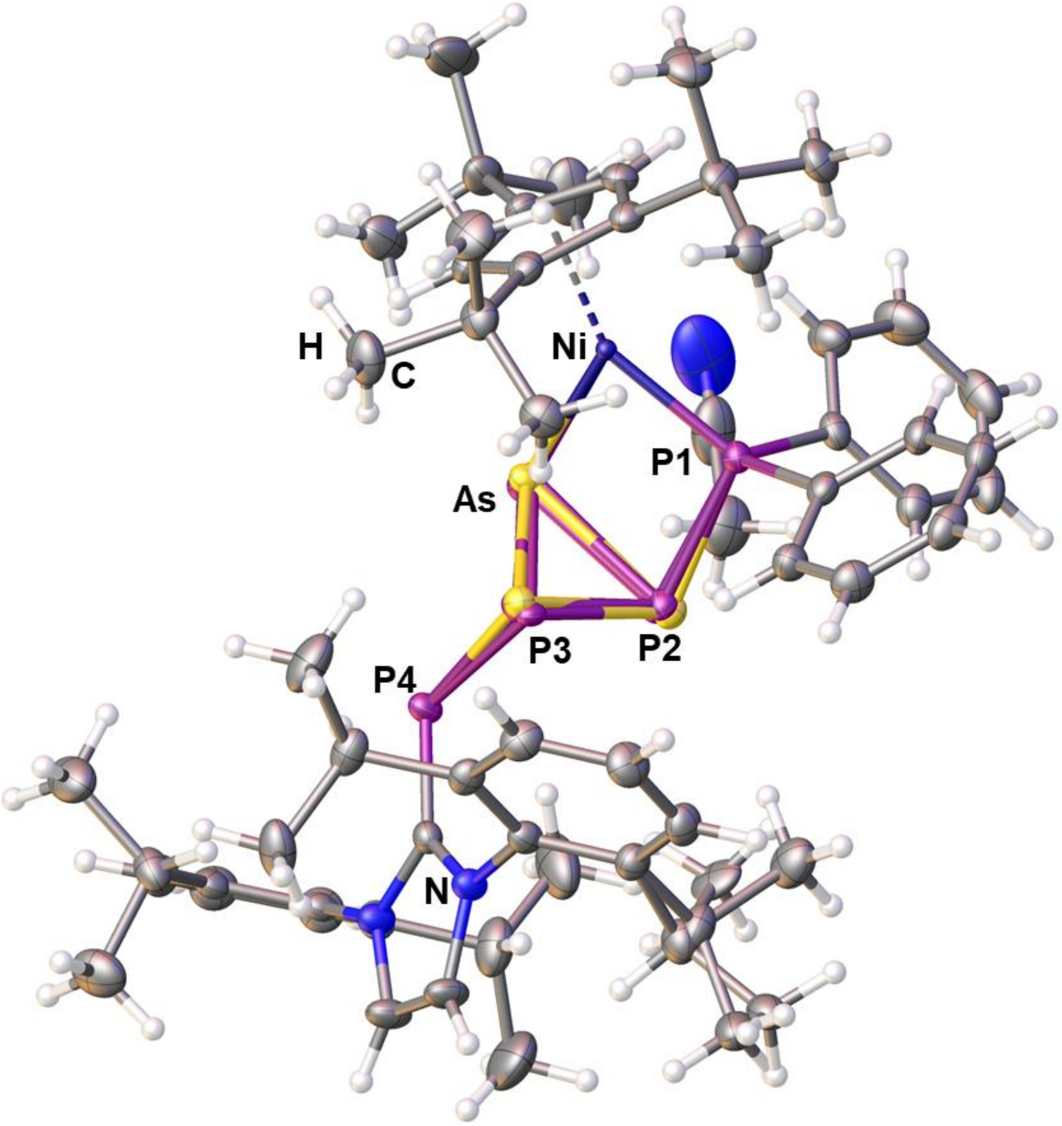

*Figure S 20: Solid state structure of **8b**. Depicted is the asymmetric unit and ADPs (anisotropic displacement parameters) are drawn at 50 % probability.*

NMR Spectra

[CpMo(CO)$_2$(η^3-P$_4$Ph$_2$)][OTf] (**A1**)

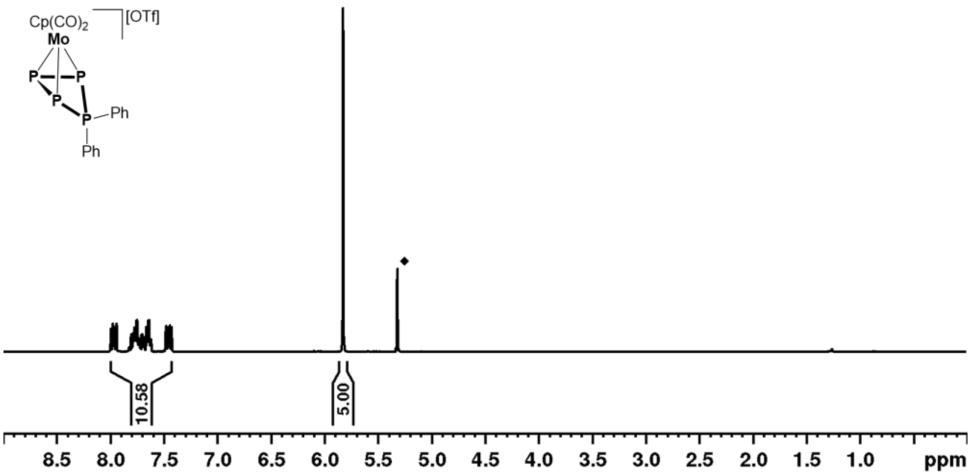

*Figure S 21: ^{1}H NMR spectrum of **A1** in CD$_2$Cl$_2$ recorded at room temperature; ◆ = residual solvent signal of CD$_2$Cl$_2$.*

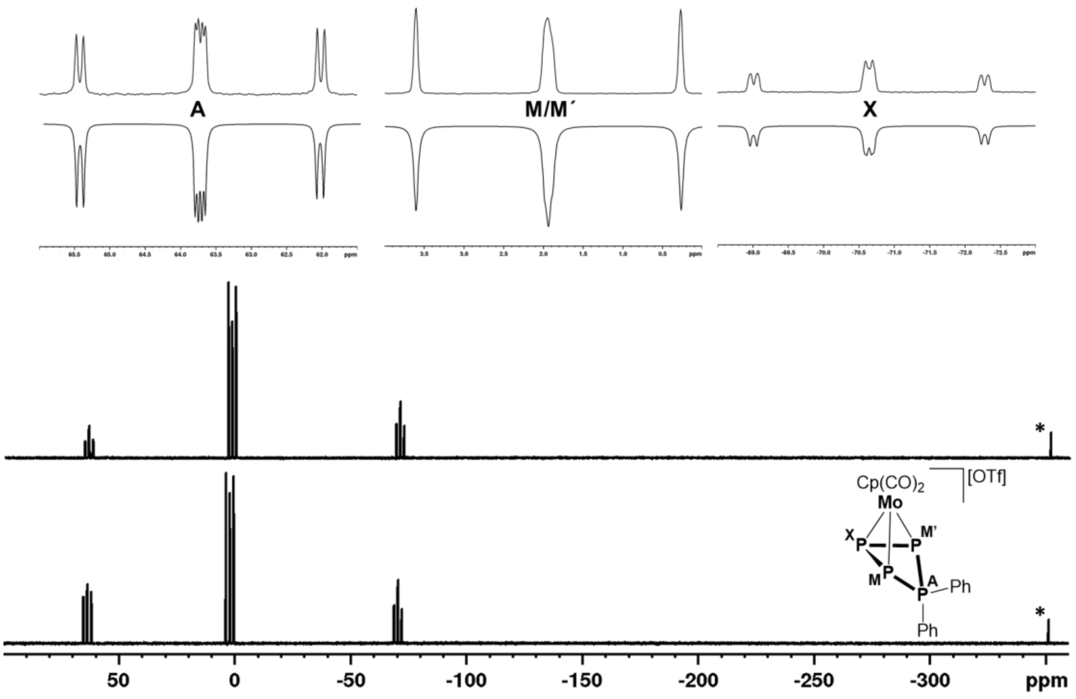

*Figure S 22: ^{31}P{^{1}H} (bottom) and ^{31}P (middle) NMR spectrum of **A1** in CD$_2$Cl$_2$ recorded at room temperature as well as enlarged signals within the measured (top) and simulated (inverted) ^{31}P{^{1}H} NMR spectrum. * = residual [CpMo(CO)$_2$(η^3-P$_3$)] (3 %).*

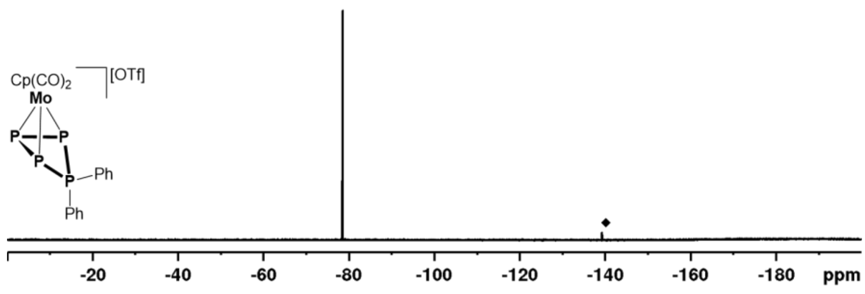

*Figure S 23: ^{19}F{^{1}H} NMR spectrum of **A1** in CD$_2$Cl$_2$ recorded at room temperature. ◆: residual o-DFB.*

$[CpMo(CO)_2(\eta^3\text{-}P_4{}^iPr_2)][TEF]$ (**A2**)

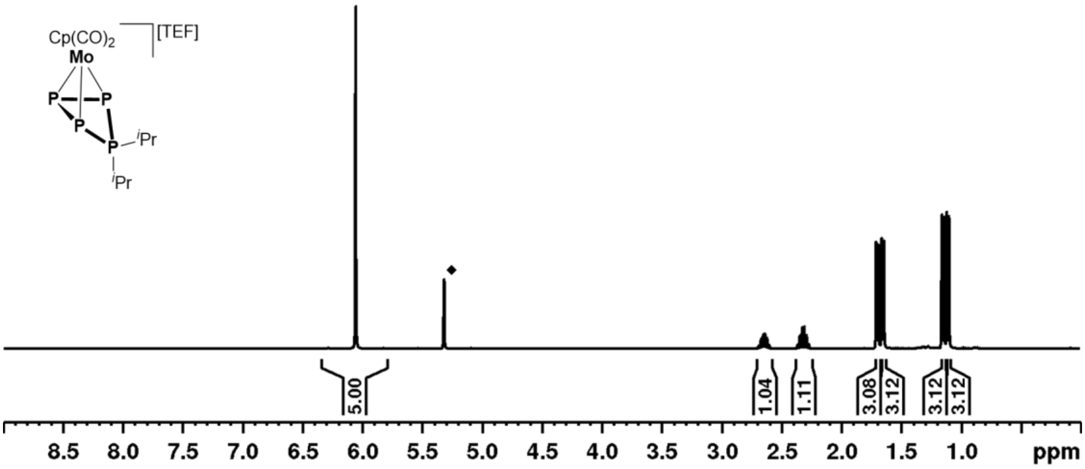

*Figure S 24: ^{1}H NMR spectrum of **A2** in CD_2Cl_2 recorded at room temperature; ◆ = residual solvent signal of CD_2Cl_2.*

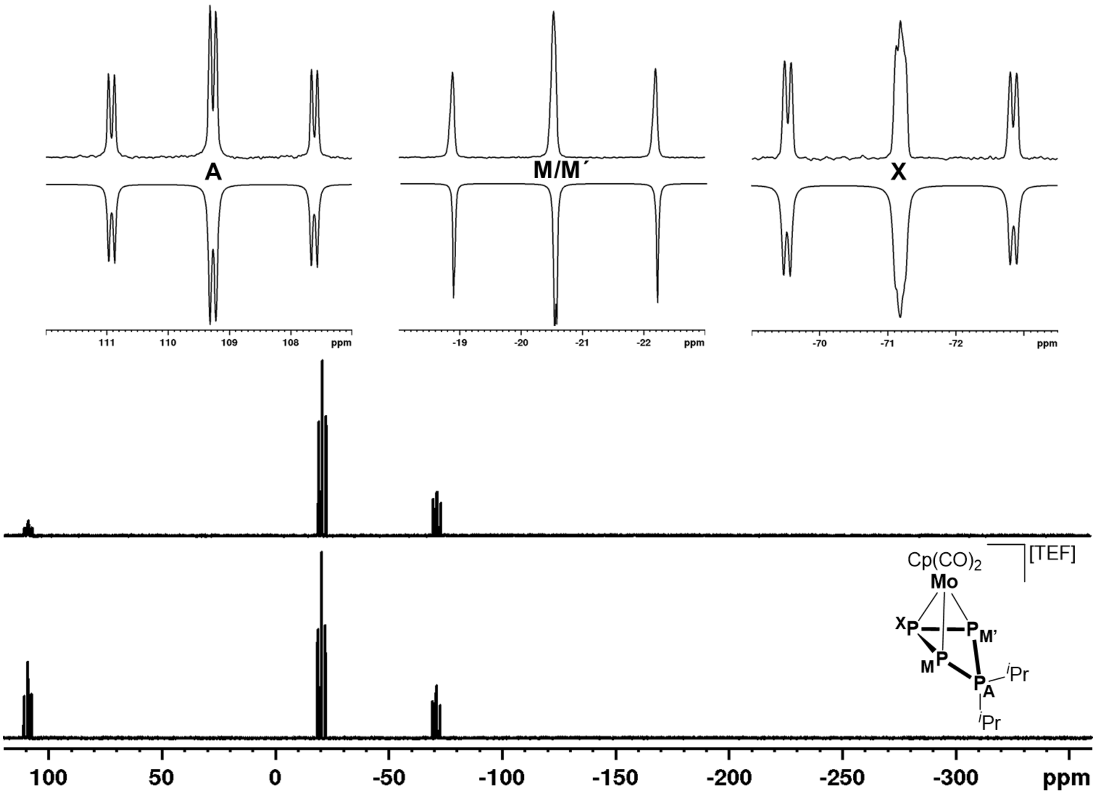

*Figure S 25: $^{31}P\{^{1}H\}$ (bottom) and ^{31}P (middle) NMR spectrum of **A2** in CD_2Cl_2 recorded at room temperature as well as enlarged signals within the measured (top) and simulated (inverted) $^{31}P\{^{1}H\}$ NMR spectrum.*

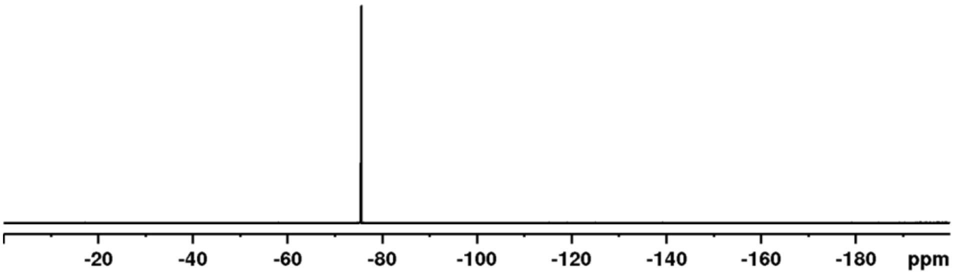

*Figure S 26: $^{19}F\{^{1}H\}$ NMR spectrum of **A2** in CD_2Cl_2 recorded at room temperature.*

[Cp'''Ni(η^3-P_4Ph_2)][PF_6] (**B1**)

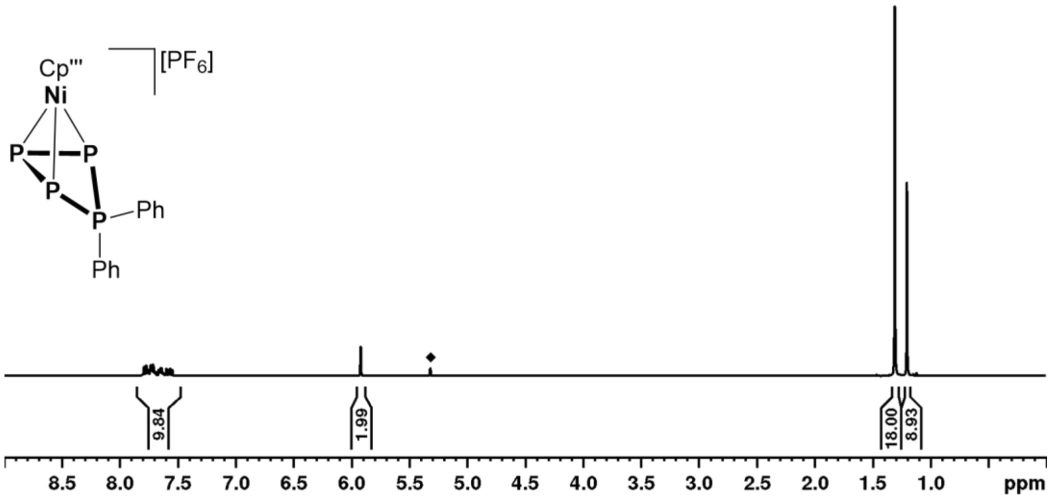

*Figure S 27: ^{1}H NMR spectrum of **B1** in CD_2Cl_2 recorded at room temperature; ◆ = residual solvent signal of CD_2Cl_2.*

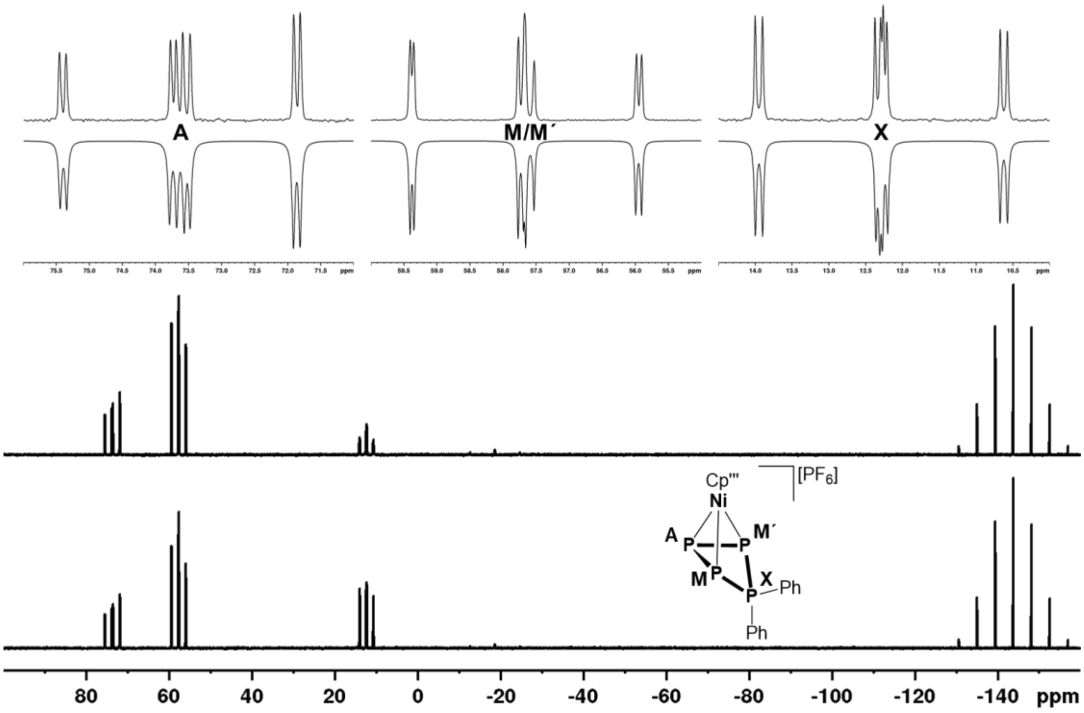

*Figure S 28: [31]P{[1]H} (bottom) and ^{31}P (middle) NMR spectrum of **B1** in CD_2Cl_2 recorded at room temperature as well as enlarged signals within the measured (top) and simulated (inverted) ^{31}P{^{1}H} NMR spectrum.*

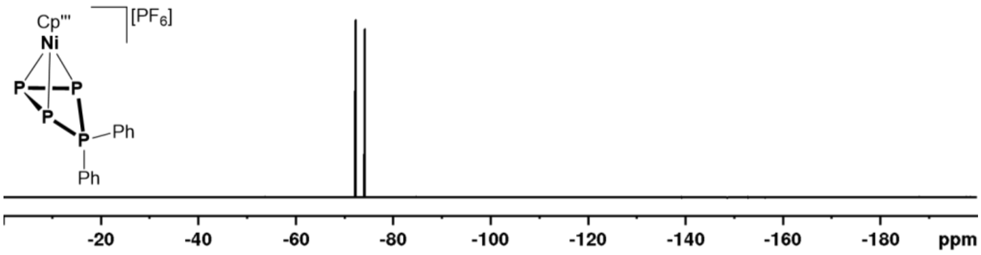

*Figure S 29: ^{19}F{^{1}H} NMR spectrum of **B1** in CD_2Cl_2 recorded at room temperature.*

[Cp'''Ni(η^3-$P_4{}^i Pr_2$)][PF_6] (**B2**)

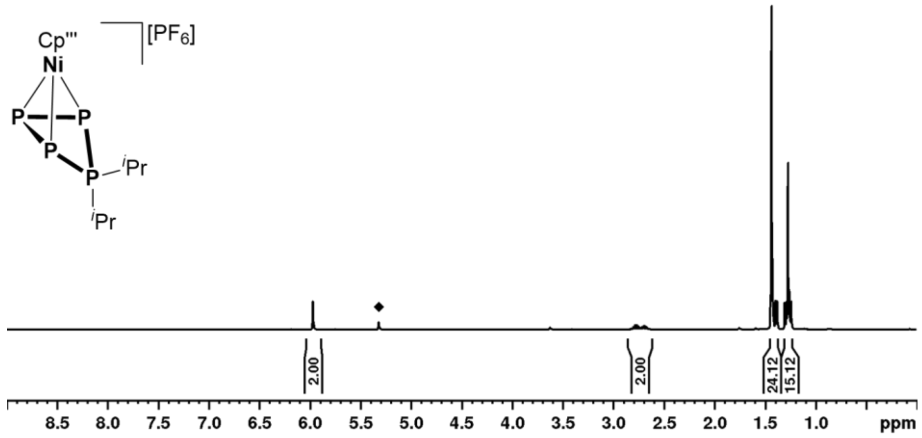

*Figure S 30: ^{1}H NMR spectrum of **B2** in CD_2Cl_2 recorded at room temperature; ◆ = residual solvent signal of CD_2Cl_2.*

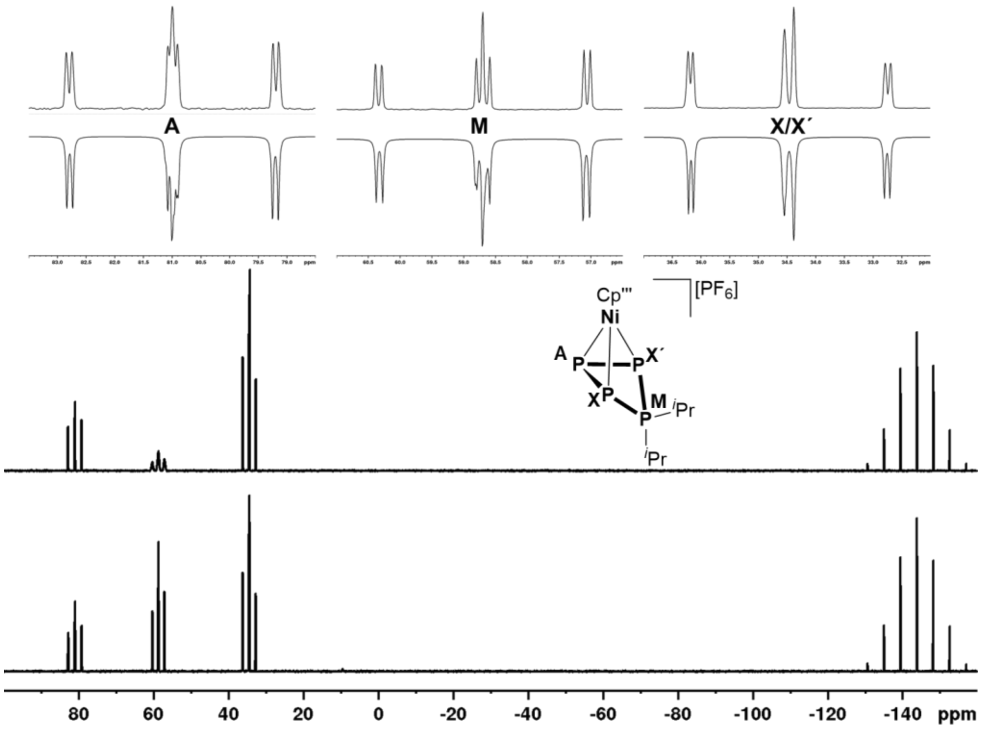

*Figure S 31: $^{31}P\{^1H\}$ (bottom) and ^{31}P (middle) NMR spectrum of **B2** in CD_2Cl_2 recorded at room temperature as well as enlarged signals within the measured (top) and simulated (inverted) $^{31}P\{^1H\}$ NMR spectrum.*

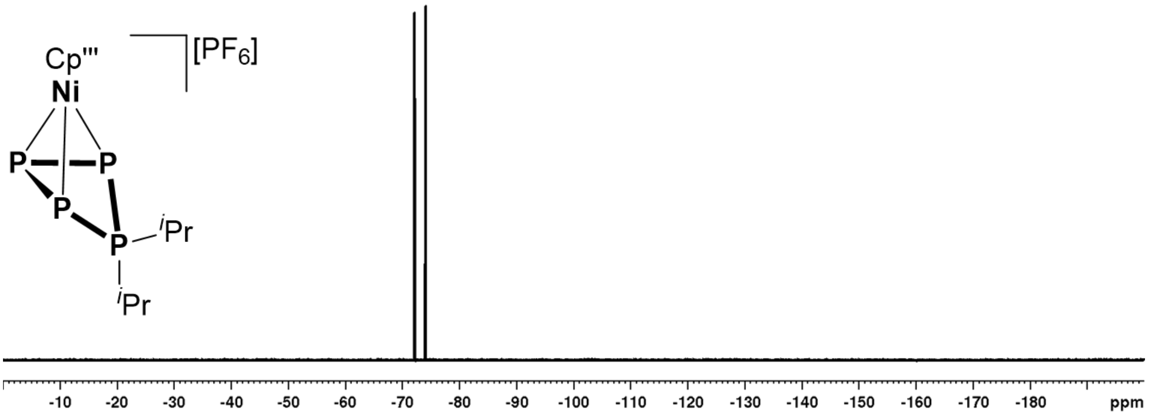

*Figure S 32: $^{19}F\{^1H\}$ NMR spectrum of **B2** in CD_2Cl_2 recorded at room temperature.*

[CpMo(CO)$_2$(η^3-P$_4$Ph$_2$IDipp)][OTf] (**1a**)

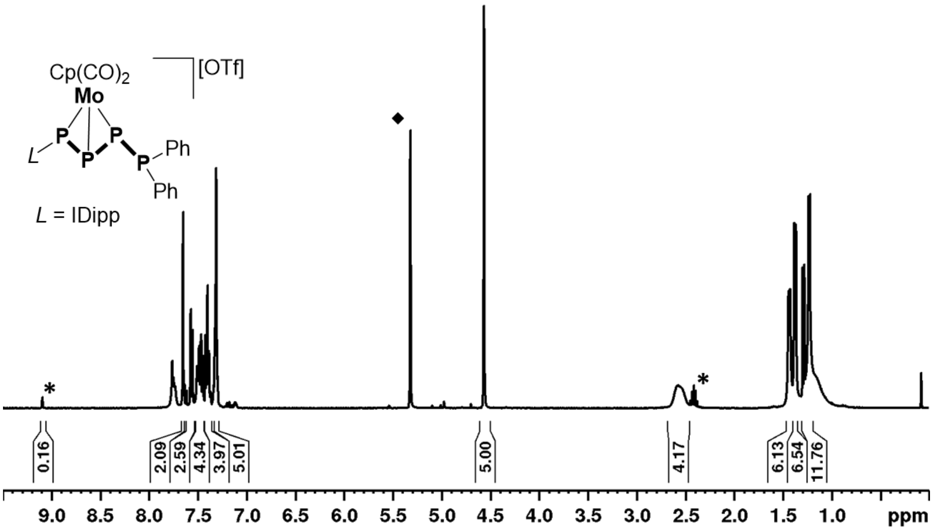

*Figure S 33: ^{1}H NMR spectrum of **1a** in CD_2Cl_2 recorded at room temperature; ◆ = residual solvent signal of CD_2Cl_2. * = trace impurities of IDippH$^+$.*

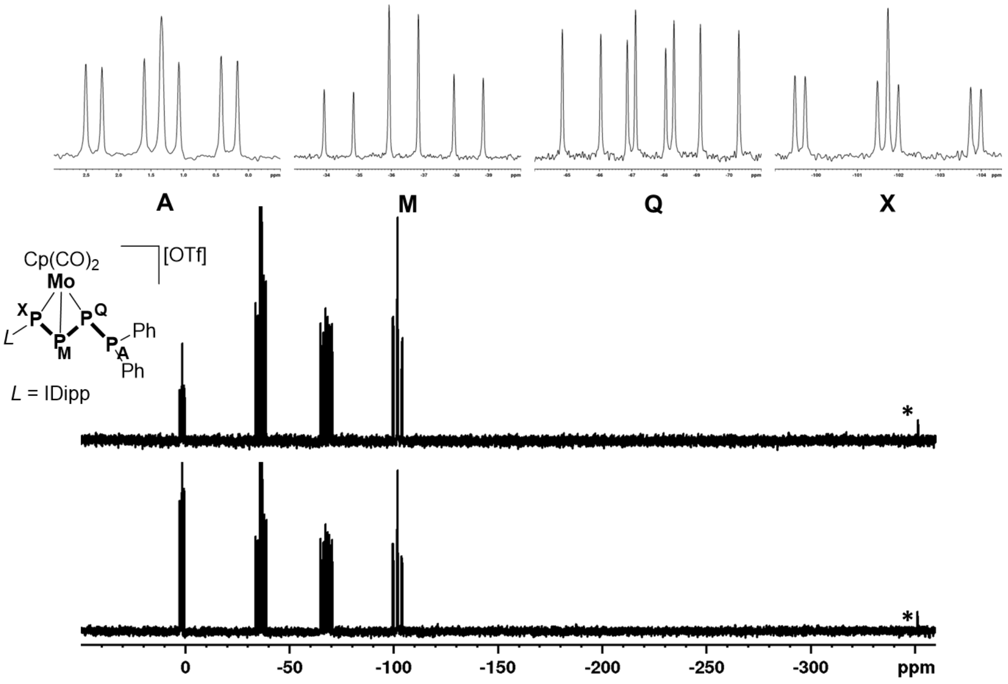

*Figure S 34: ^{31}P{^{1}H} (bottom) and ^{31}P NMR spectrum of **1a** in CD_2Cl_2 recorded at room temperature as well as enlarged signals within the measured ^{31}P{^{1}H} NMR spectrum. * = residual [CpMo(CO)$_2$(η^3-P$_3$)] (<1 %).*

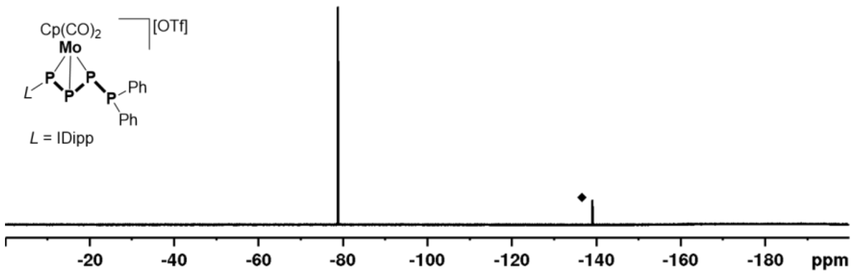

*Figure S 35: ^{19}F{^{1}H} NMR spectrum of **1a** in CD_2Cl_2 recorded at room temperature. ◆ = residual o-DFB.*

As to get further insight into the formation pathway of **1a**, variable temperature ^{31}P NMR spectroscopy was employed. Dissolving **A1** (32 mg, 0.05 mmol) and IDipp (19 mg, 0.05 mmol) in 0.7 mL of -80°C cold THF-d^8 and then subjected to NMR spectroscopy (Figure S36). The formation of **1a$_{INT}$** appears to be immediate as indicated by its presence of in the ^{31}P NMR spectra at -80 °C. Furthermore **1a$_{INT}$** appears to be temperature stable up to r.t. °C at which it slowly starts to rearrange to **1a**. This rearrangement process drastically speeds up upon further warming the sample to 60 °C, at which there is only product **1a** left in solution. To ensure that no equilibrium of **1a$_{INT}$** and **1a** is present, a control spectrum at room temperature was measured at room temperature after heating to 60 °C, which only showed **1a**. During the heating process to 60 °C, small amounts of decomposition products are observable.

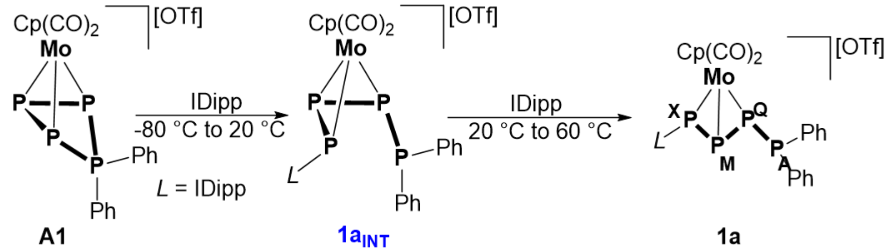

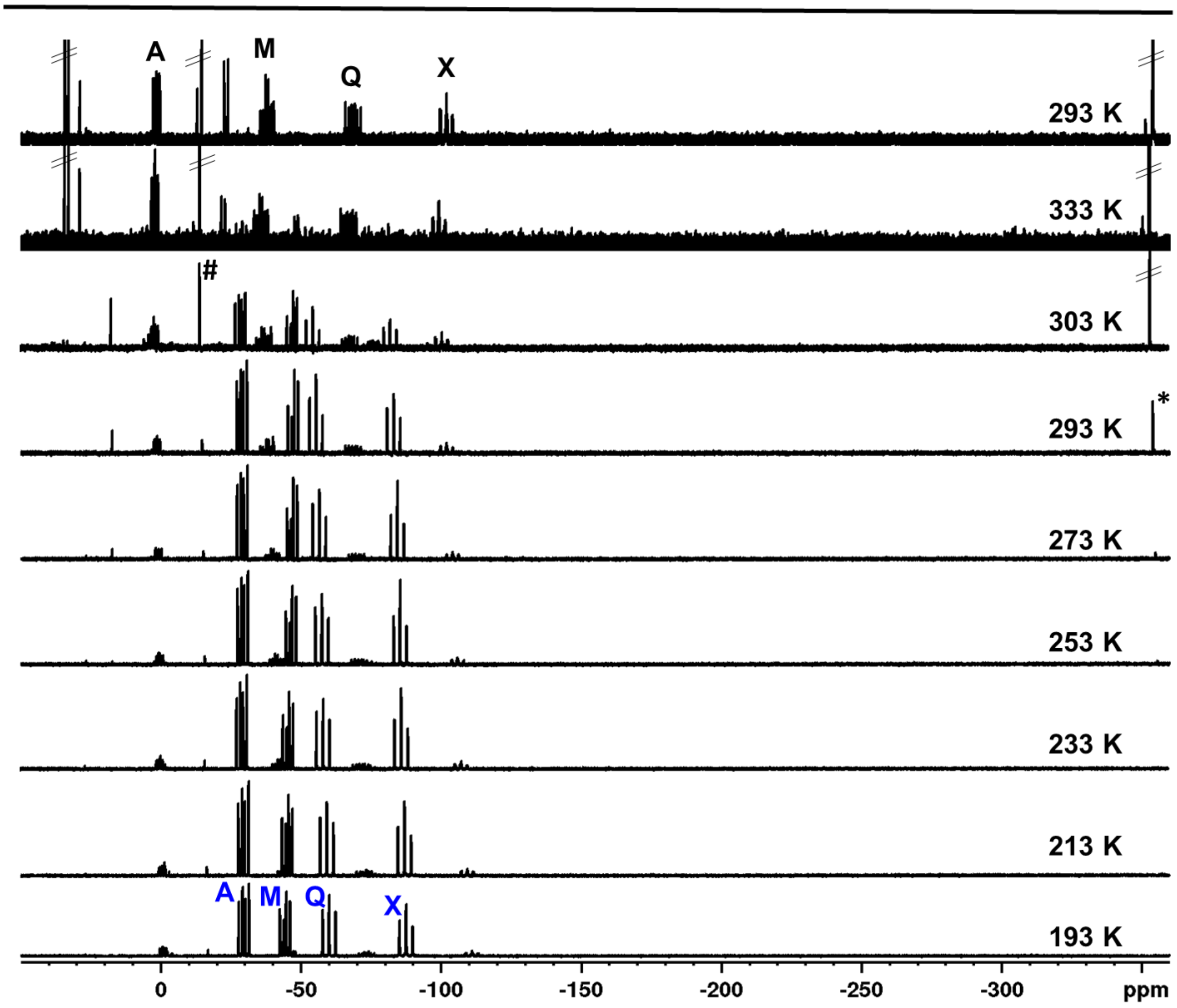

*Figure S 36: $^{31}P\{^1H\}$ NMR spectra of the reaction of **A1** with IDipp intermediately forming **1a$_{INT}$**, which rearranges at room temperature to afford **1a**; # marks $(PPh_2)_2$ and * marks $[CpMo(CO)_2(\eta^3\text{-}P_3)]$.*

[CpMo(CO)$_2$(η^3-P$_4$Ph$_2$I^{i}Pr$_2$Me$_2$)][OTf] (**1b**)

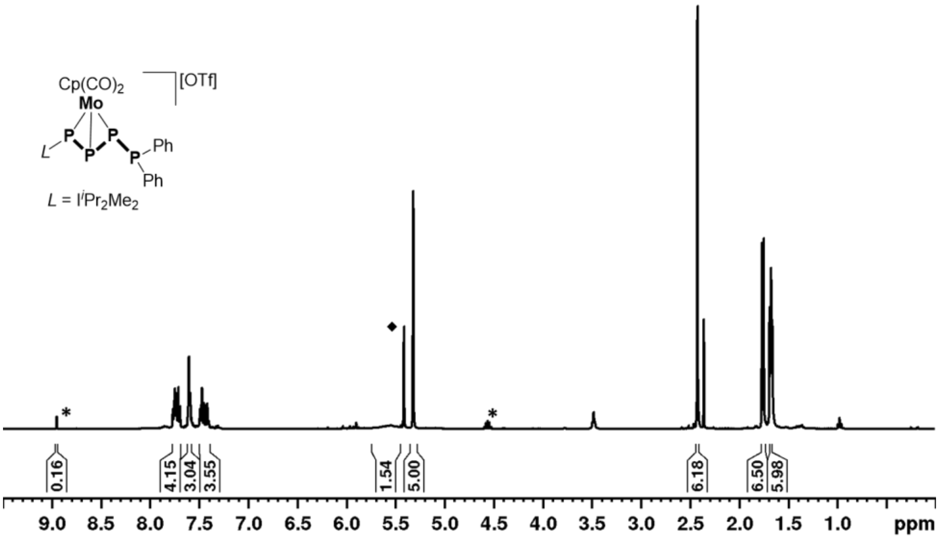

*Figure S 37: ^{1}H NMR spectrum of **1b** in CD_2Cl_2 recorded at room temperature; ◆ = residual solvent signal of CD_2Cl_2. * = trace impurities of I^{i}Pr$_2$Me$_2$H$^+$.*

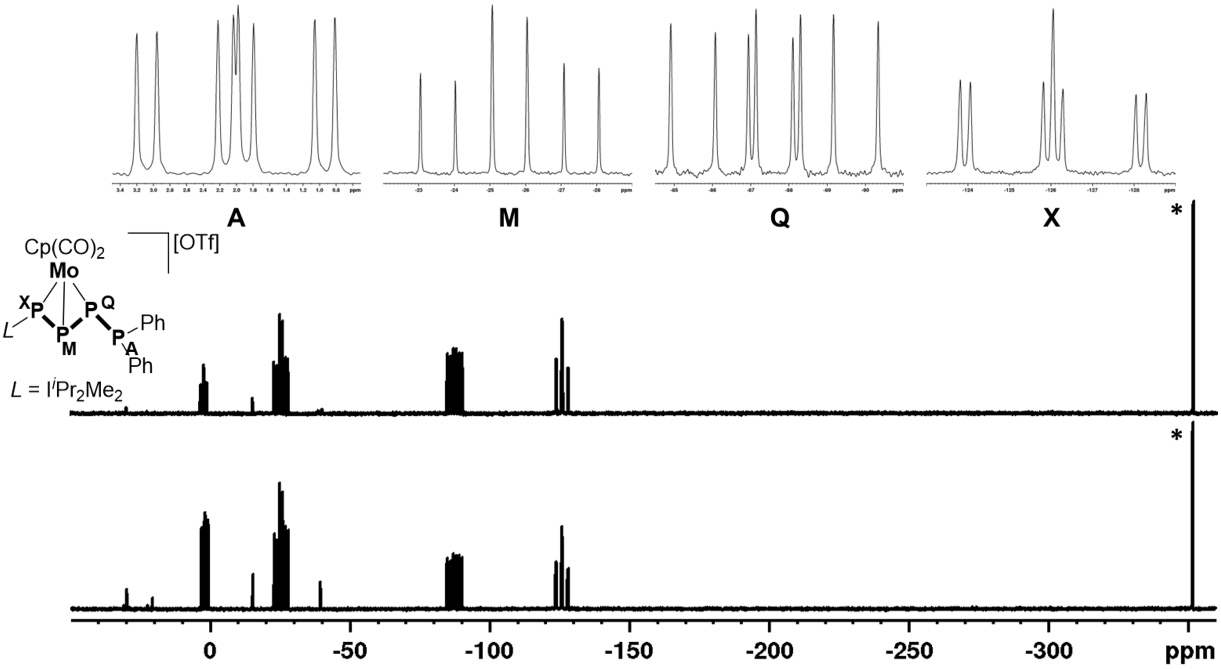

*Figure S 38: ^{31}P{^{1}H} (bottom) and ^{31}P NMR spectrum of **1b** in CD_2Cl_2 recorded at room temperature as well as enlarged signals within the measured ^{31}P{^{1}H} NMR spectrum. * = residual [CpMo(CO)$_2$(η^3-P$_3$)] (<1 %).*

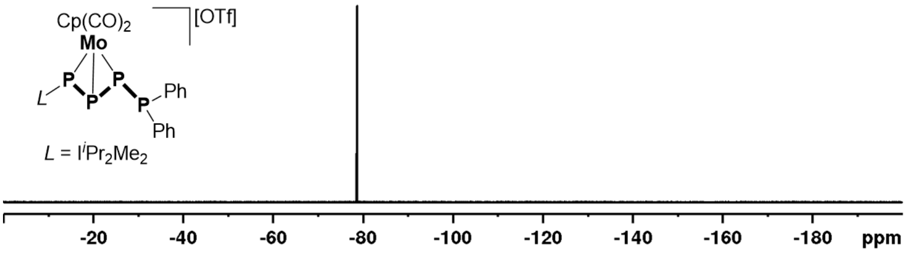

*Figure S 39: ^{19}F{^{1}H} NMR spectrum of **1b** in CD_2Cl_2 recorded at room temperature.*

[CpMo(CO)$_2$(η^3-P$_4$^{i}Pr$_2$IDipp)][TEF] (**1c**)

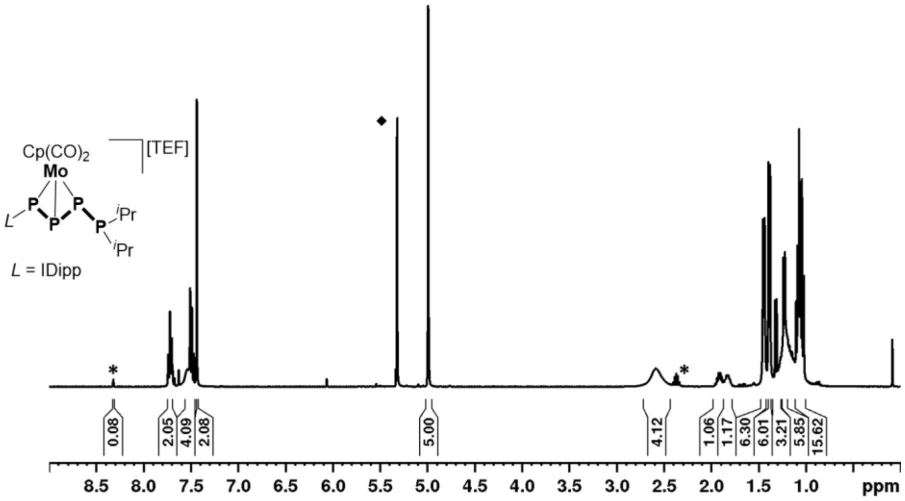

*Figure S 40: ^{1}H NMR spectrum of **1c** in CD_2Cl_2 recorded at room temperature; ◆ = residual solvent signal of CD_2Cl_2. * = trace impurities of IDippH$^+$.*

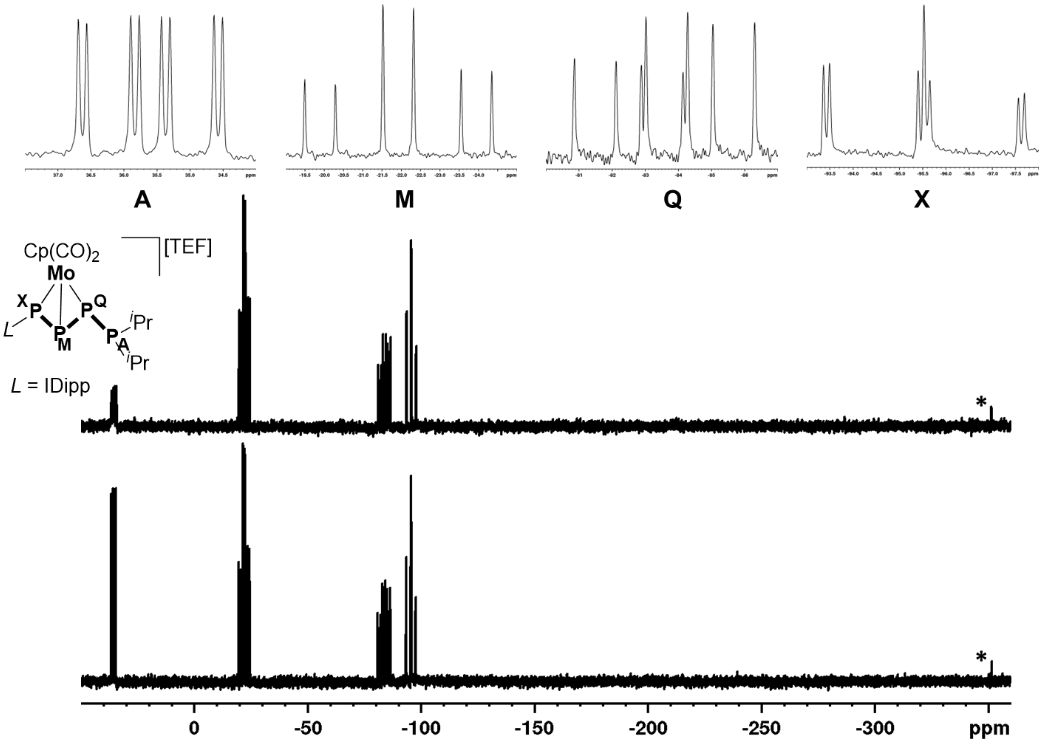

*Figure S 41: ^{31}P{^{1}H} (bottom) and ^{31}P NMR spectrum of **1c** in CD_2Cl_2 recorded at room temperature as well as enlarged signals within the measured ^{31}P{^{1}H} NMR spectrum. * = residual [CpMo(CO)$_2$(η^3-P$_3$)] (<1 %).*

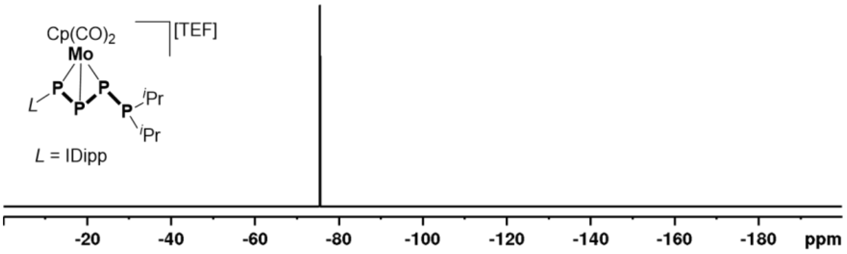

*Figure S 42: ^{19}F{^{1}H} NMR spectrum of **1c** in CD_2Cl_2 recorded at room temperature.*

[CpMo(CO)$_2$(η^3-P$_4{}^i$Pr$_2$I^{i}Pr$_2$Me$_2$)][TEF] (**1d**)

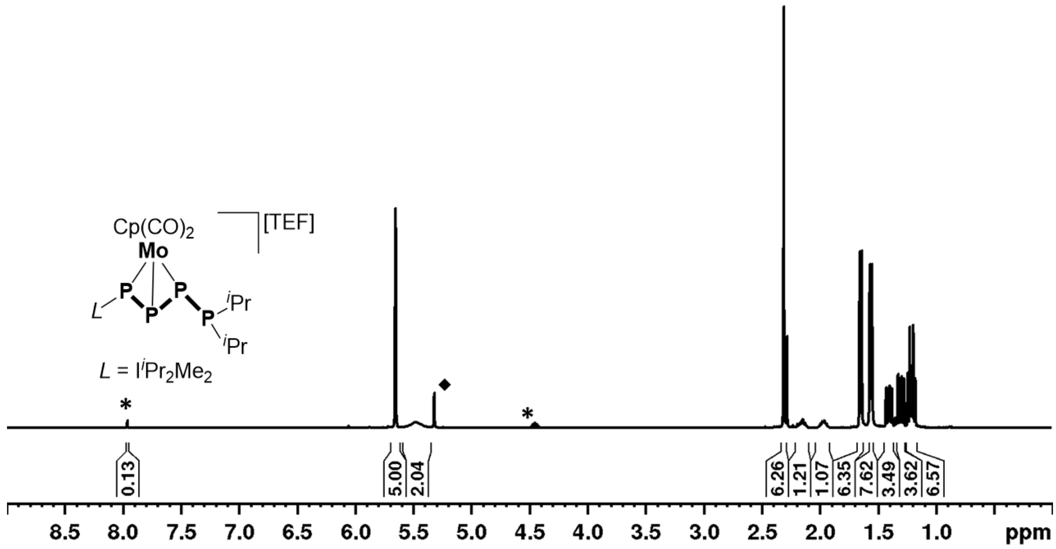

*Figure S 43: ^{1}H NMR spectrum of **1d** in CD$_2$Cl$_2$ recorded at room temperature; ◆ = residual solvent signal of CD$_2$Cl$_2$. * = trace impurities of I^{i}Pr$_2$Me$_2$H$^+$.*

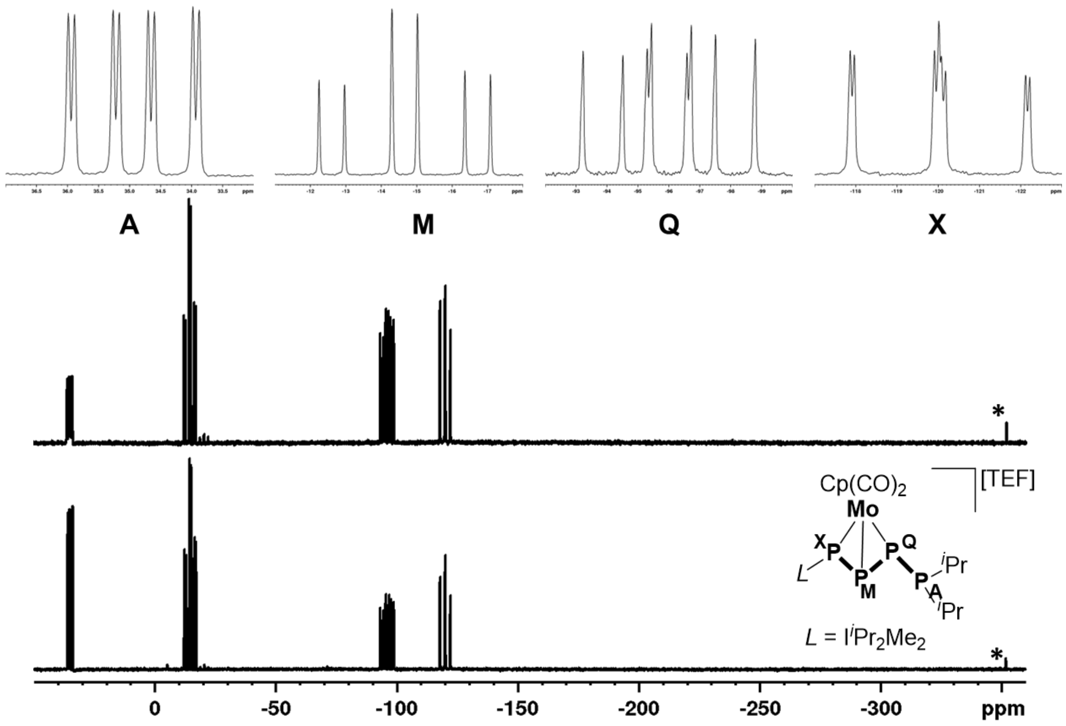

*Figure S 44: ^{31}P{^{1}H} (bottom) and ^{31}P NMR spectrum of **1d** in CD$_2$Cl$_2$ recorded at room temperature as well as enlarged signals within the measured ^{31}P{^{1}H} NMR spectrum. * = residual [CpMo(CO)$_2$(η^3-P$_3$)] (<1 %).*

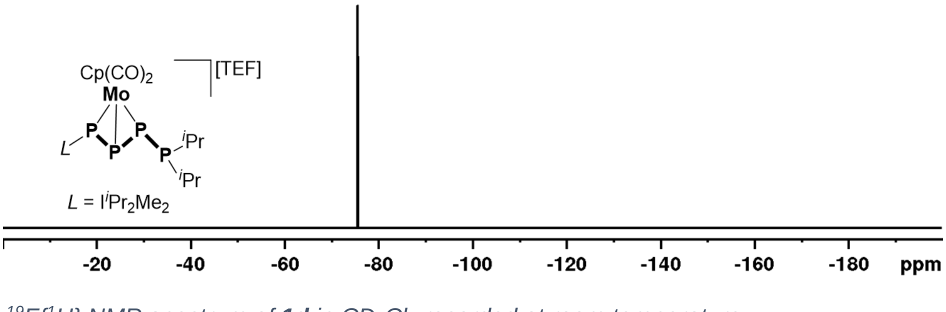

*Figure S 45: ^{19}F{^{1}H} NMR spectrum of **1d** in CD$_2$Cl$_2$ recorded at room temperature.*

[CpMo(CO)$_2$($\eta^{1:1}$-I^{i}Pr$_2$Me$_2$PPP(OEt)PPh$_2$)] (**2**)

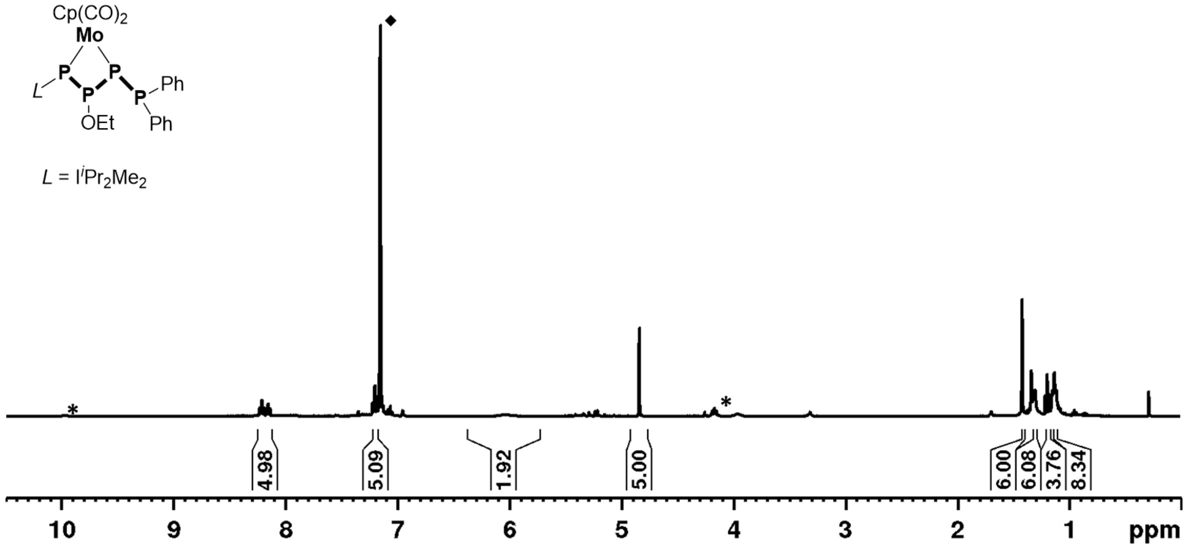

*Figure S 46: ^{1}H NMR spectrum of **2** in C_6D_6 recorded at room temperature; ◆ = residual solvent signal of C_6D_6 * = trace impurities of I^{i}Pr$_2$Me$_2$H$^+$. Integrals in the range of 1-2 ppm are slightly off due to the presence of I^{i}Pr$_2$Me$_2$H$^+$.*

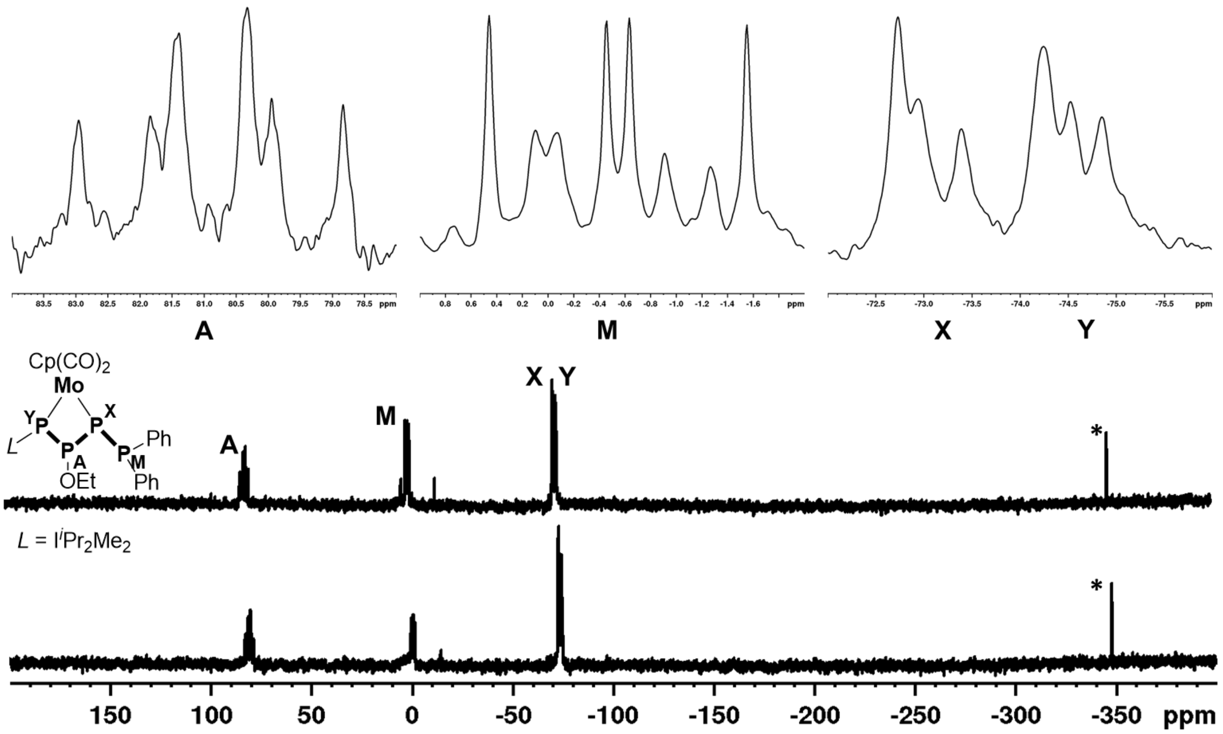

*Figure S 47: ^{31}P (bottom) and ^{31}P{^{1}H} (middle) NMR spectra as well as enlarged signals (top) of **2** in C_6D_6 recorded at room temperature; * = residual [CpMo(CO)$_2$(η^3-P$_3$)] (4 %).*

Unfortunately, only a very tiny amount of crystals of **2** could be obtained. The ^{31}P{^{1}H} NMR spectrum depicted in Figure S47 did not allow us to determine the coupling constants. This is on the one hand attributed to solvent effects, low signal to noise ratio as well as high sensibility of the compound. Nevertheless, to get further insight into the behavior of **2** in solution and to provide the actual coupling constants of **2**, we decided to analyse and depict the crude NMR spectrum of **2** measured in THF with C_6D_6 capillary (see Figure S48). Here, the signals are fully resolved and allowed us to analyze **2** in detail. The measured crude spectrum of **2** fits very well the simulated spectrum.

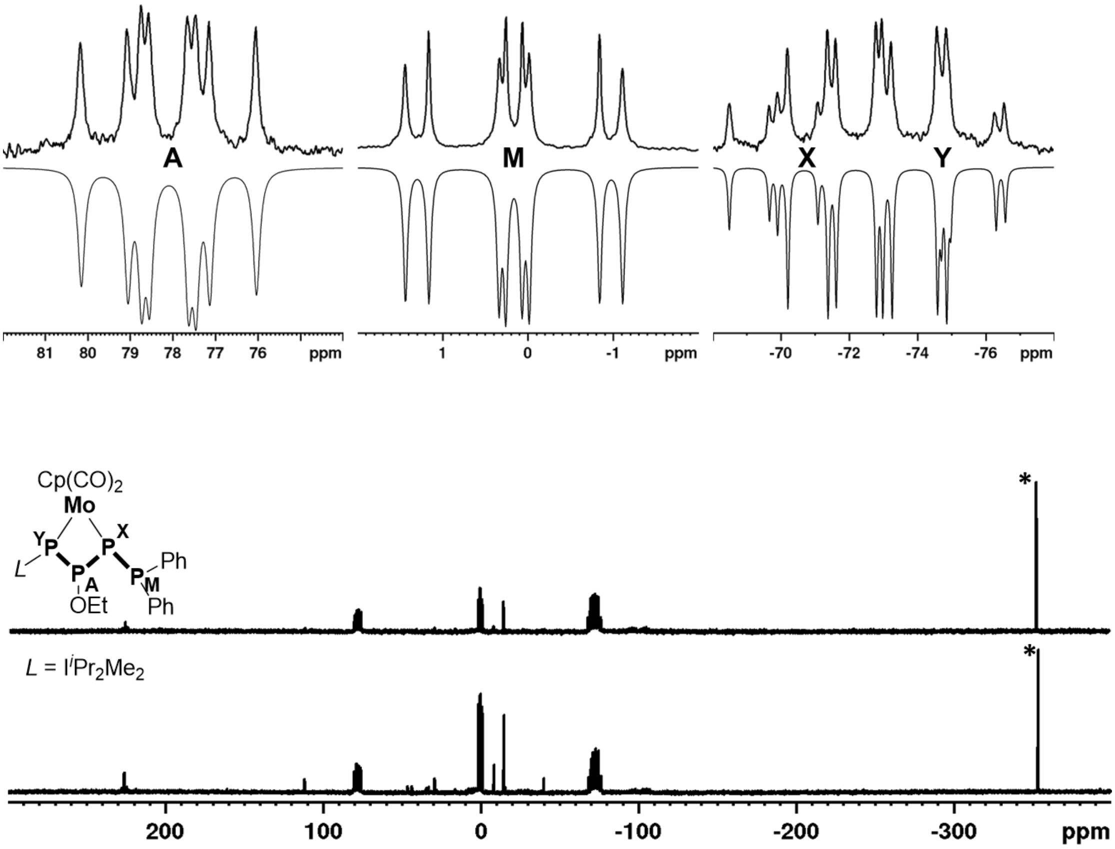

*Figure S 48: $^{31}P\{^1H\}$ (bottom) and ^{31}P (middle) crude NMR spectra of **2** in THF with C_6D_6 capillary recorded at room temperature as well as enlarged signals within the measured (top) and simulated (inverted) $^{31}P\{^1H\}$ NMR spectrum; * = residual [CpMo(CO)$_2$(η^3-P_3)].*

[Cp'''Ni($\eta^{1:1}$-P_4Ph_2IDipp)][PF_6] (**3a**)

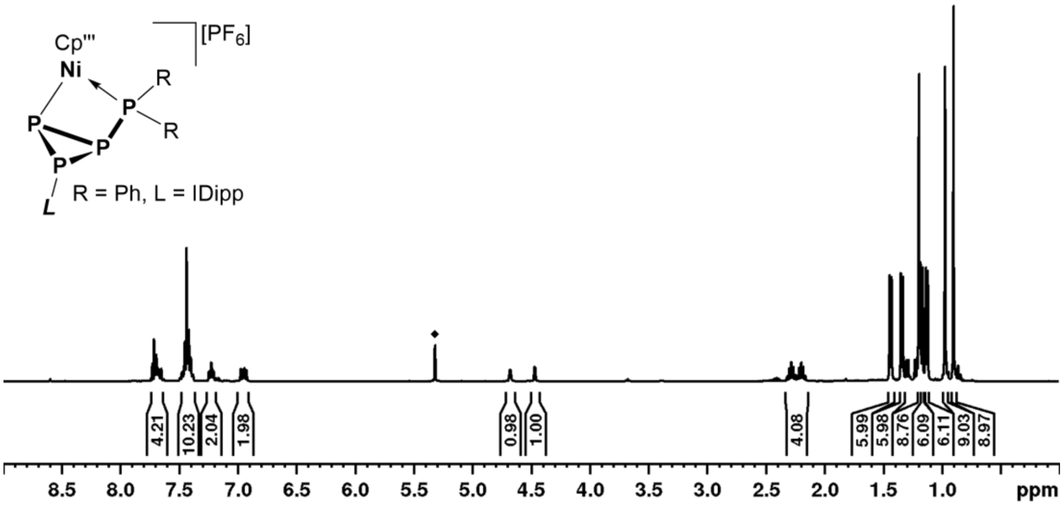

*Figure S 49: ^{1}H NMR spectrum of **3a** in CD_2Cl_2 recorded at room temperature; ◆ = residual solvent signal of CD_2Cl_2.*

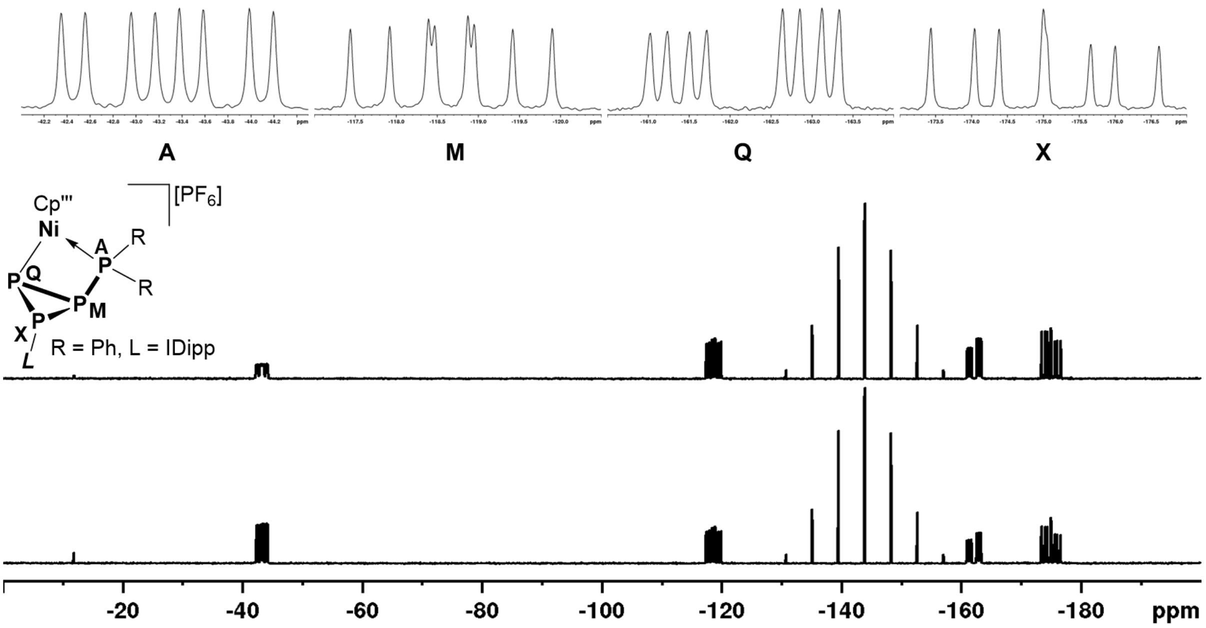

*Figure S 50: $^{31}P\{^{1}H\}$ (bottom) and ^{31}P NMR spectrum of **3a** in CD_2Cl_2 recorded at room temperature as well as enlarged signals within the measured $^{31}P\{^{1}H\}$ NMR spectrum.*

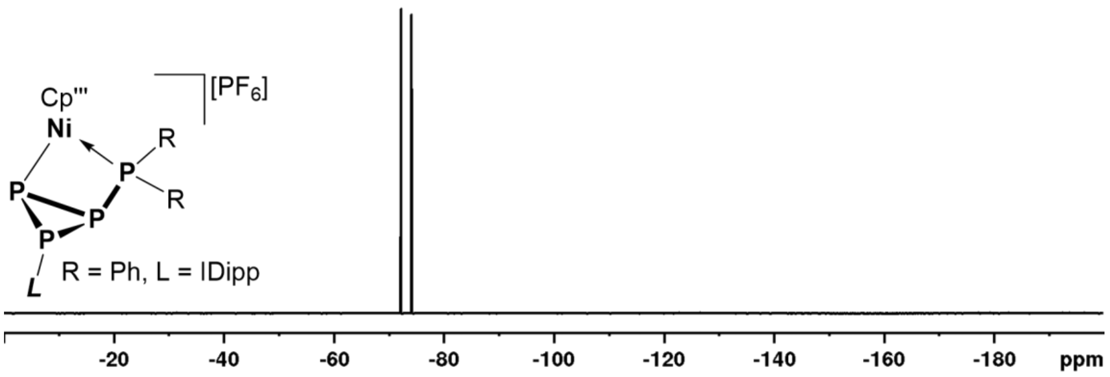

*Figure S 51: $^{19}F\{^{1}H\}$ NMR spectrum of **3a** in CD_2Cl_2 recorded at room temperature.*

As to get further insight into the formation pathway of **3a**, variable temperature ^{31}P NMR spectroscopy was employed. Dissolving **B1** (71 mg, 0.1 mmol) and IDipp (39 mg, 0.1 mmol) in 0.7 mL of -80°C cold THF-d^8 and then subjected to NMR spectroscopy (Figure S52). The formation of **3a$_{INT}$** appears to be immediate as indicated by its presence in the ^{31}P NMR spectra at -80 °C. Furthermore **3a$_{INT}$** appears to partly rearrange immediately (already at -80 °C) to **1a**. This rearrangement process drastically speeds up upon further warming the sample to -20 °C, at which there is only product **3a** left in solution.

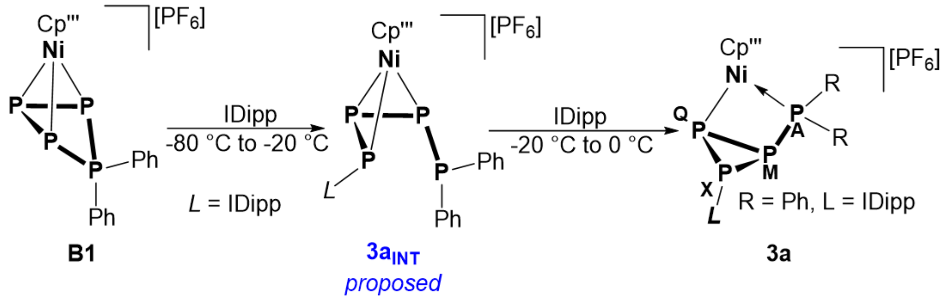

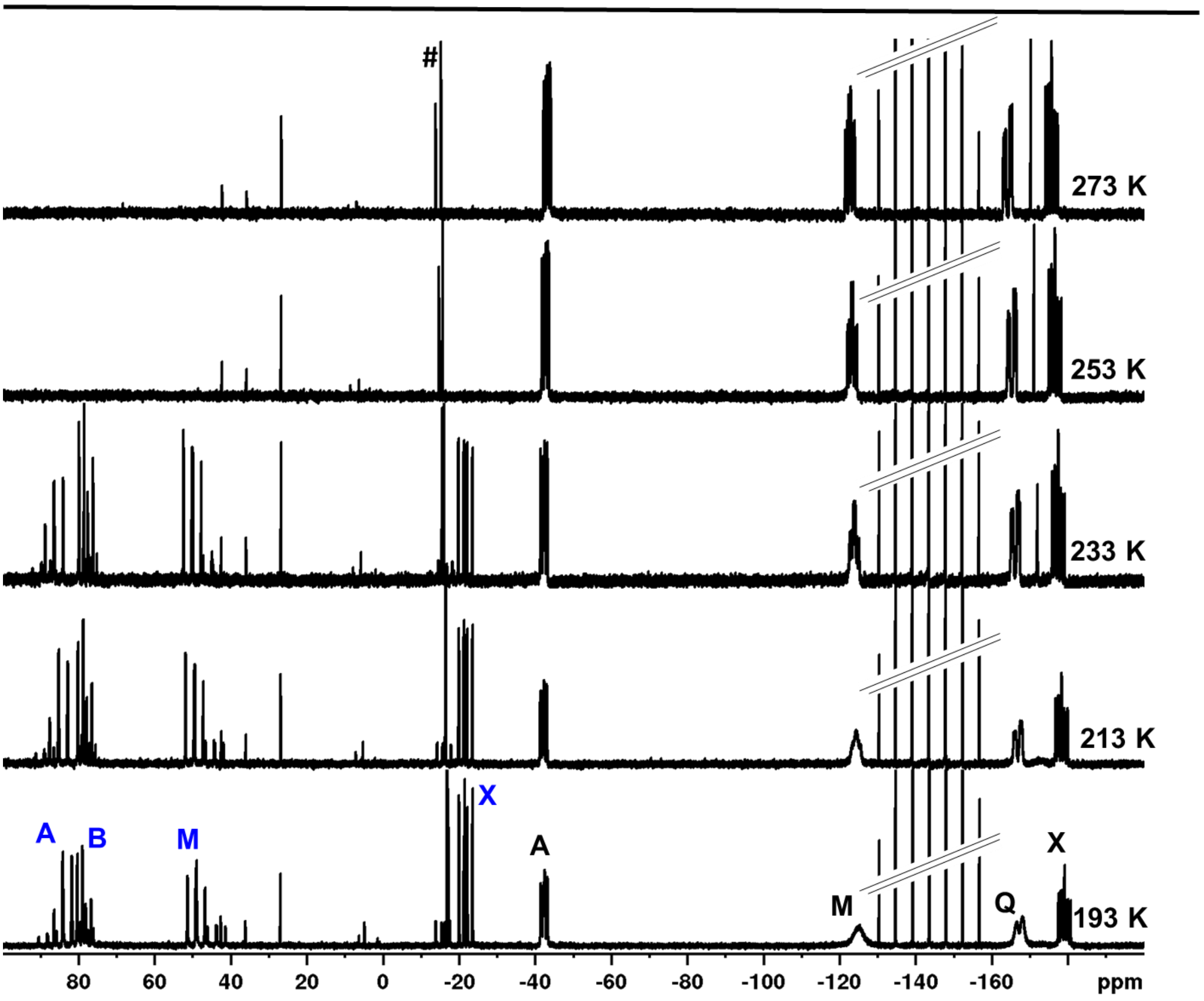

*Figure S 52: ^{31}P{^{1}H} NMR spectra of the reaction of **B1** with IDipp intermediately forming **3a$_{INT}$** and **3a**, which rearranges at -20 °C fully to **3a**; # marks ($(PPh_2)_2$).*

$[Cp'''Ni(\eta^{1:1}-P_4Ph_2I^iPr_2Me_2)][PF_6]$ (**3b**)

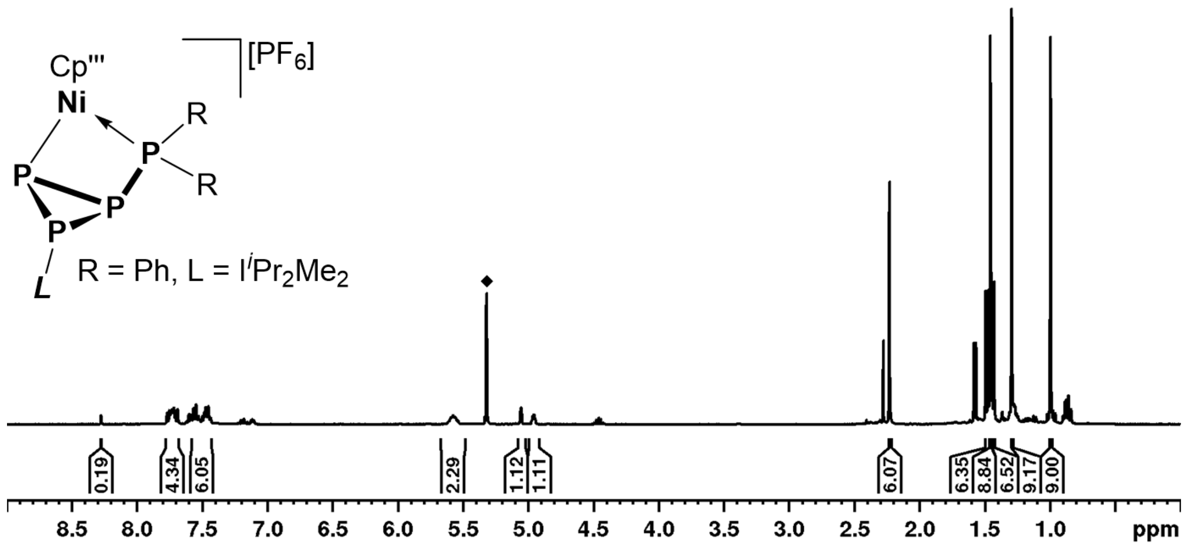

*Figure S 53: ^1H NMR spectrum of **3b** in CD_2Cl_2 recorded at room temperature; ◆ = residual solvent signal of CD_2Cl_2, several small unassigned signals mark trace impurities of $I^iPr_2Me_2H^+$.*

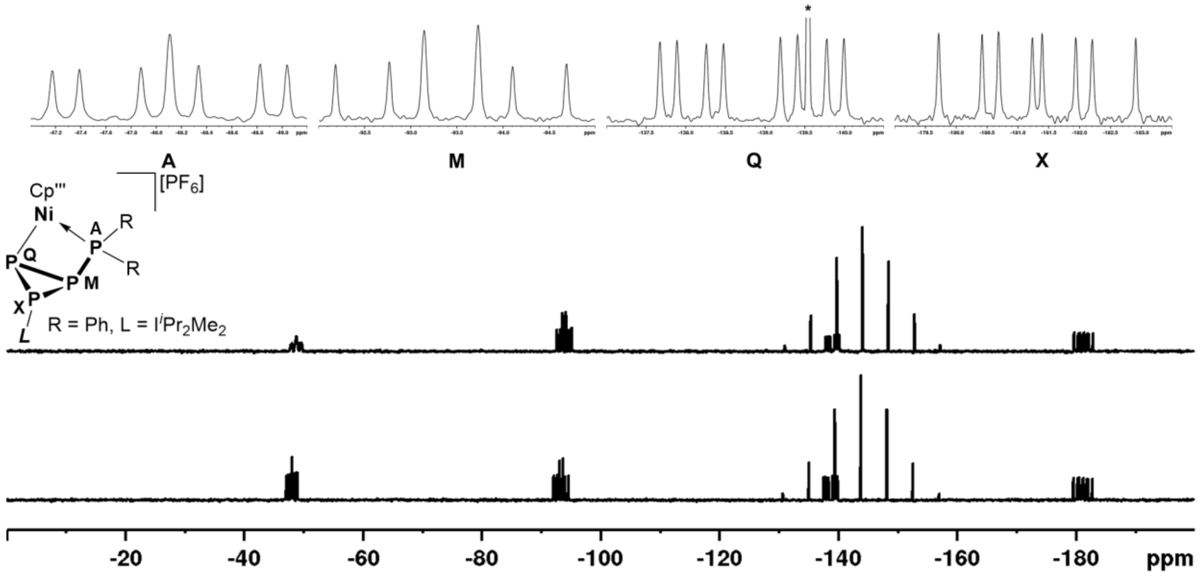

*Figure S 54: $^{31}P\{^1H\}$ (bottom) and ^{31}P NMR spectrum of **3b** in CD_2Cl_2 recorded at room temperature as well as enlarged signals within the measured $^{31}P\{^1H\}$ NMR spectrum; * marks one of the lines of the $[PF_6]^-$ signal.*

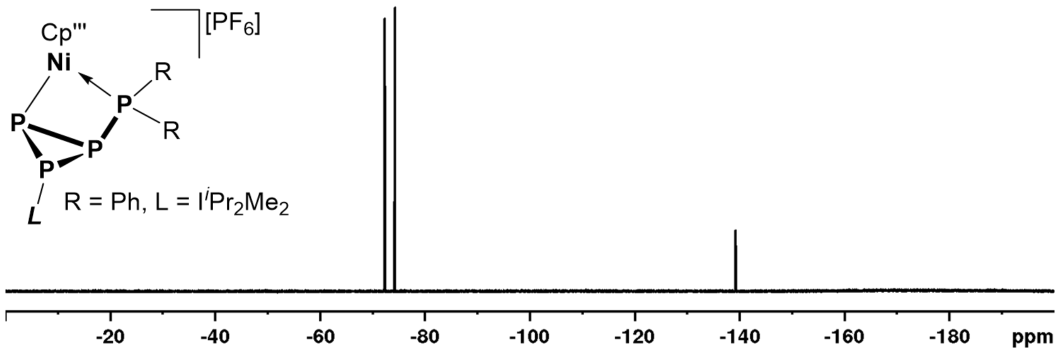

*Figure S 55: $^{19}F\{^1H\}$ NMR spectrum of **3b** in CD_2Cl_2 recorded at room temperature.*

[Cp'''Ni($\eta^{1:1}$-$P_4{}^iPr_2$IDipp)][PF_6] (**3c**)

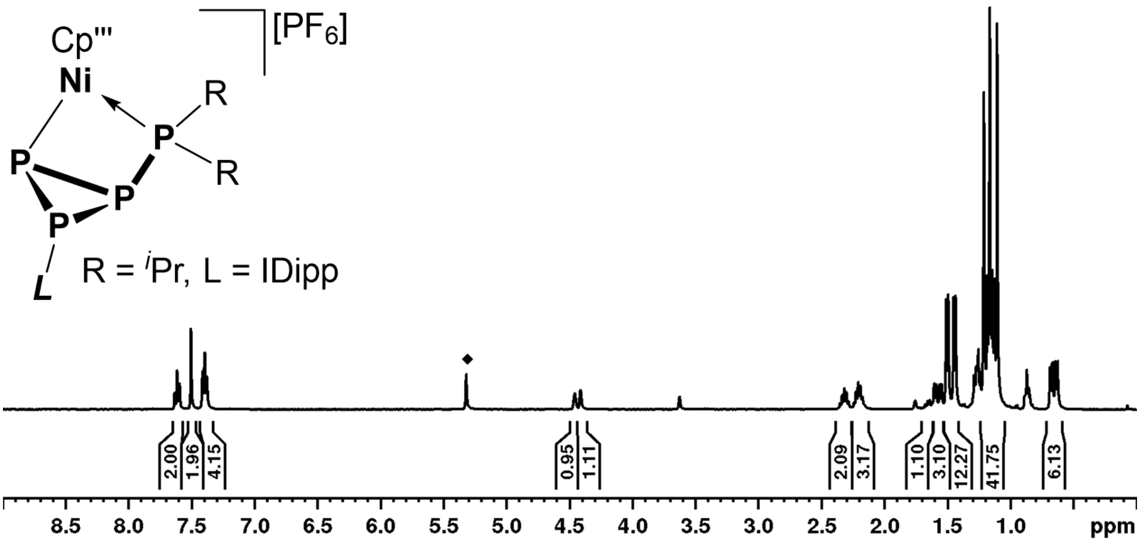

*Figure S 56: ^{1}H NMR spectrum of **3c** in CD_2Cl_2 recorded at room temperature; ◆ = residual solvent signal of CD_2Cl_2.*

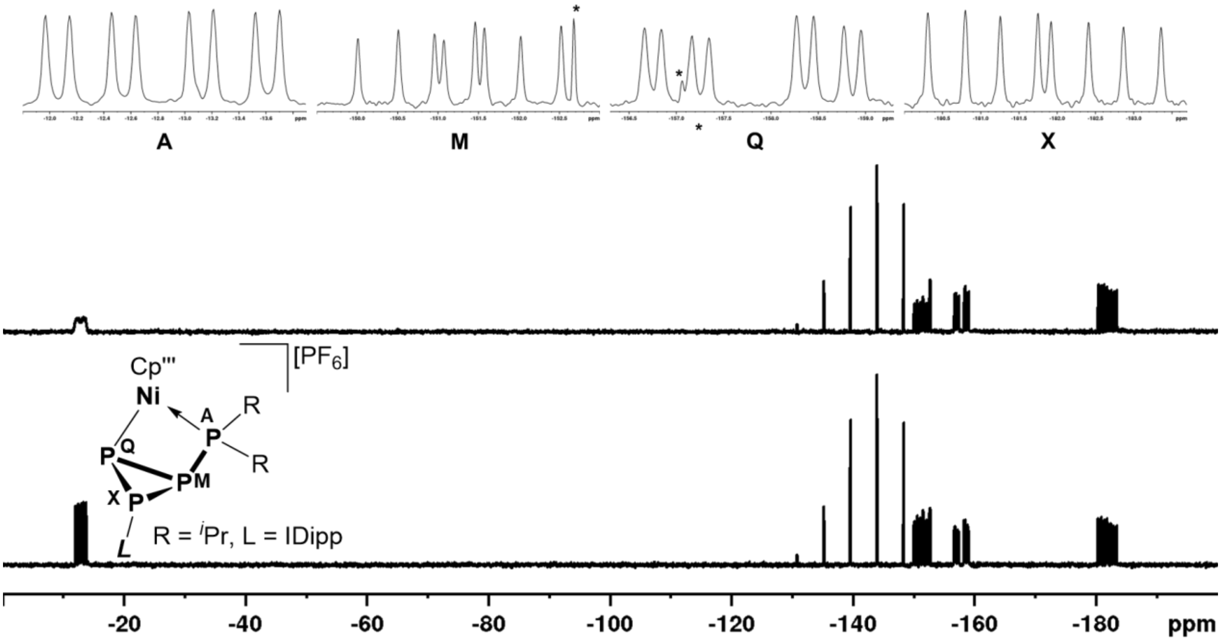

*Figure S 57: ^{31}P{^{1}H} (bottom) and ^{31}P NMR spectrum of **3c** in CD_2Cl_2 recorded at room temperature as well as enlarged signals within the measured ^{31}P{^{1}H} NMR spectrum; * marks lines of the [PF_6]$^-$ signal.*

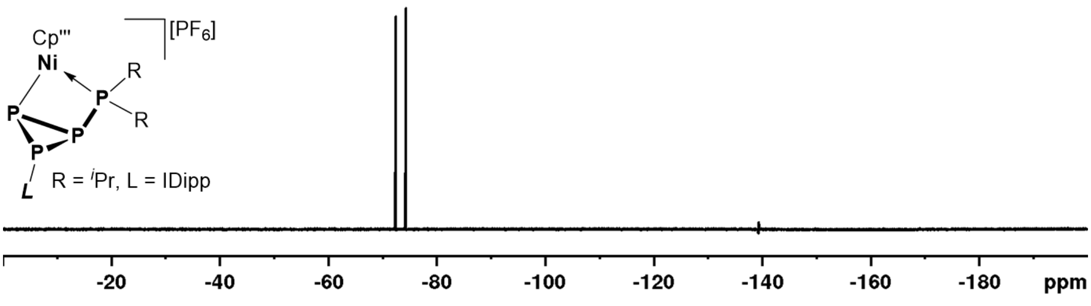

*Figure S 58: ^{19}F{^{1}H} NMR spectrum of **3c** in CD_2Cl_2 recorded at room temperature.*

[Cp'''Ni($\eta^{1:1}$-$P_4{}^iPr_2I^iPr_2Me_2$)][PF_6] (**3d**)

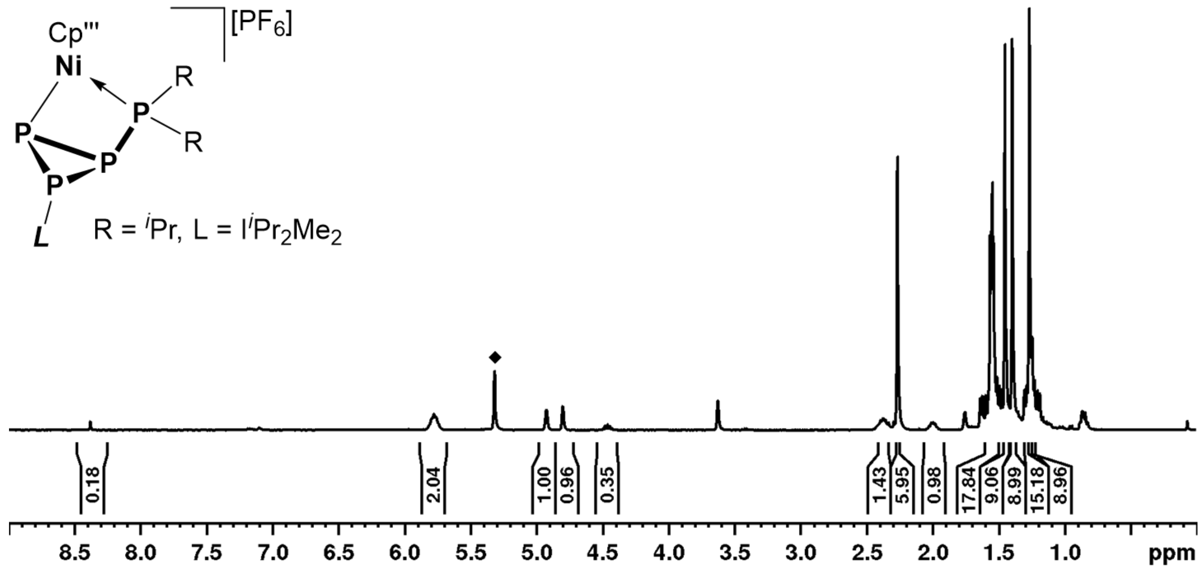

*Figure S 59: ^1H NMR spectrum of **3d** in CD_2Cl_2 recorded at room temperature; ◆ = residual solvent signal of CD_2Cl_2, several small unassigned signals mark trace impurities of $I^iPr_2Me_2H^+$.*

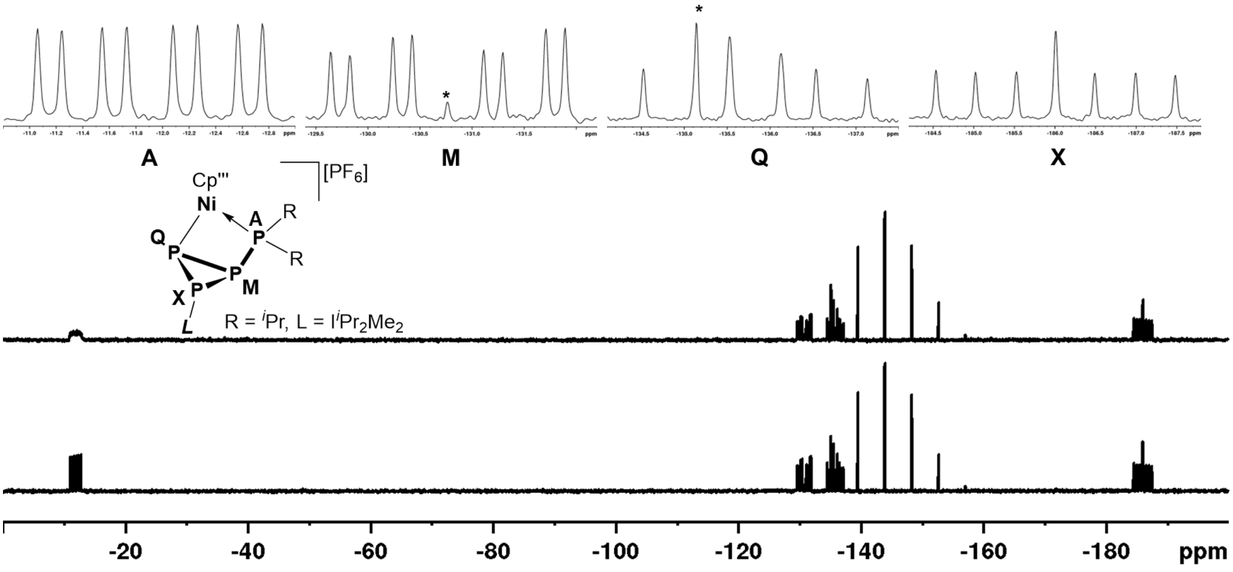

*Figure S 60: $^{31}P\{^1H\}$ (bottom) and ^{31}P NMR spectrum of **3d** in CD_2Cl_2 recorded at room temperature as well as enlarged signals within the measured $^{31}P\{^1H\}$ NMR spectrum; * marks lines of the $[PF_6]^-$ signal.*

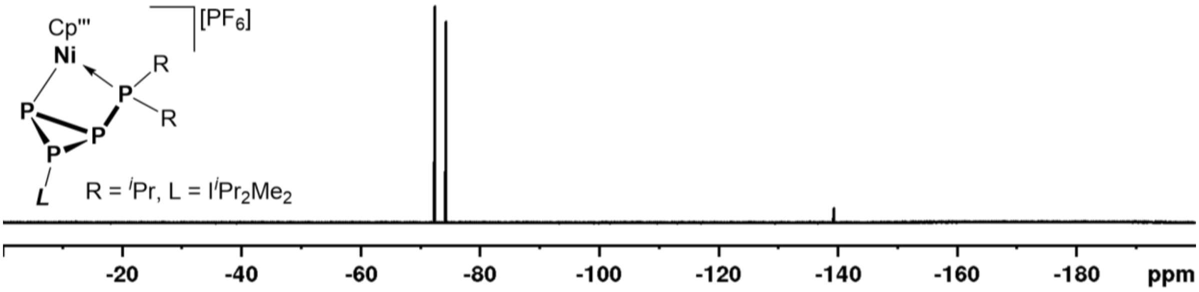

*Figure S 61: $^{19}F\{^1H\}$ NMR spectrum of **3d** in CD_2Cl_2 recorded at room temperature.*

[Cp'''Ni(η^2-IDippPP(OEt)PP^{i}Pr$_2$)] (**4**)

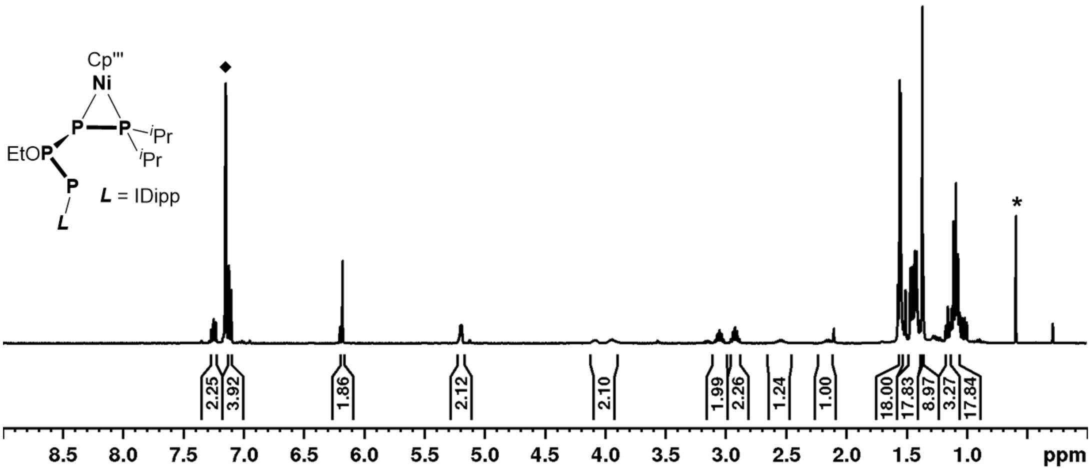

*Figure S 62: ^{1}H NMR spectrum of **4** in C_6D_6 recorded at room temperature; ◆ = residual solvent signal of C_6D_6, * = residual MeCN from the dissolved crystals.*

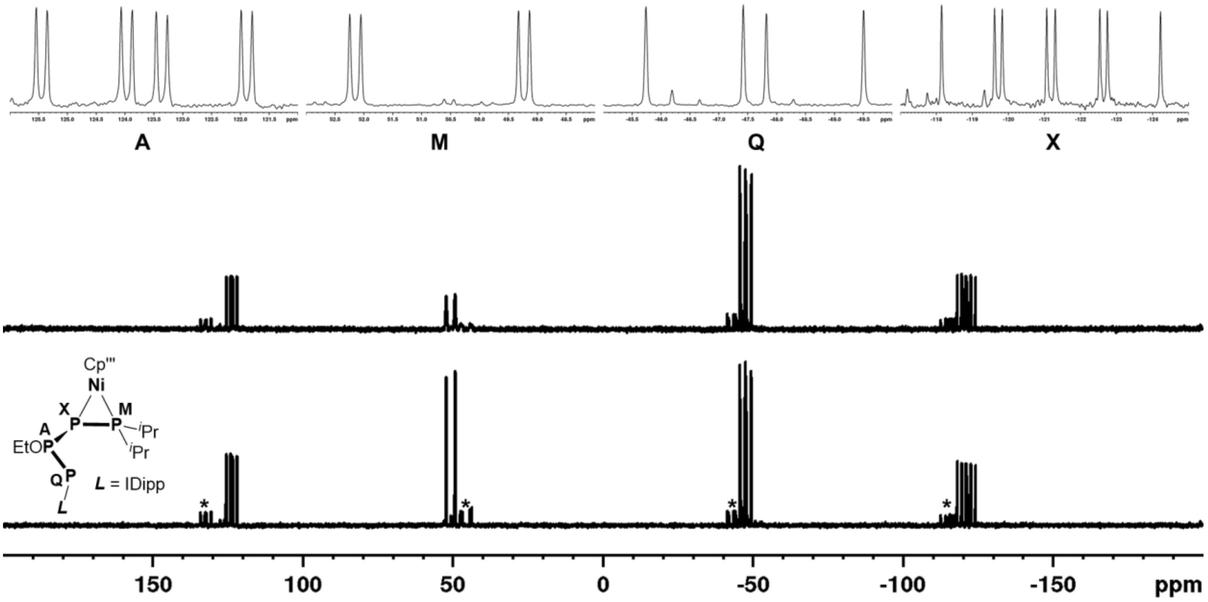

*Figure S 63: ^{31}P{^{1}H} (bottom) and ^{31}P (middle) NMR spectra of **4** in C_6D_6 recorded at room temperature as well as enlarged signals within the measured (top) ^{31}P{^{1}H} NMR spectrum; * = second isomer of **4**.*

As to get further insight into the intriguing color change during the synthesis of **4**, variable temperature ^{31}P NMR spectroscopy was employed. Dissolving **3c** (52 mg, 0.05 mmol) and KOEt (5 mg, 0.05 mmol) in 0.6 mL of THF-d^8 affords a dark turquoise solution of $\mathbf{4_{INT}}$ which was then subjected to NMR spectroscopy (Figure S64). As complete dissolution of KOEt was not possible under the given conditions (the NMR tube was shaken several times outside the cold bath to guarantee it not warming up beyond -80 °C), some staring material **3c** is still present within the mixture. The formation of $\mathbf{4_{INT}}$ appears to be immediate as indicated by its sole presence in the ^{31}P NMR spectra at -80 °C. Furthermore, $\mathbf{4_{INT}}$ appears to be temperature stable up to -20 °C at which it slowly starts to rearrange to **4**. This rearrangement process

drastically speeds up upon further warming the sample to 0 °C and then room temperature, at which there is only product **4** left in solution.

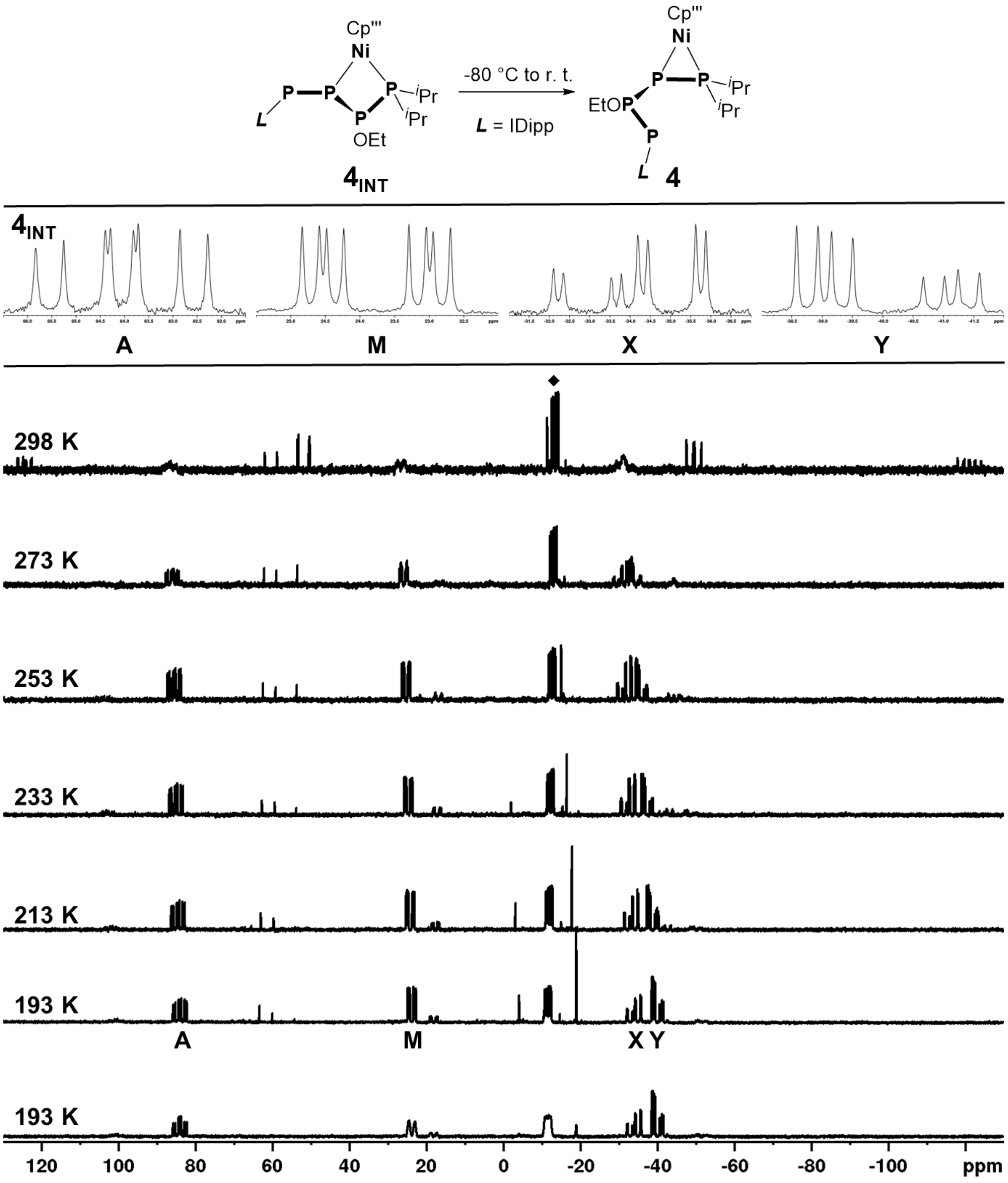

*Figure S 64: ^{31}P (bottom) and ^{31}P{^{1}H} NMR spectra of the reaction of **3c** (52 mg, 0.05 mmol) with KOEt (5 mg, 0.05 mmol) intermediately forming **4_{INT}**, which rapidly rearranges at around -10 °C to afford **4**; Enlarged signals of **4_{INT}** at -80 °C (top); ♦ marks residual starting material **3c**.*

Reaction of [Cp'''Co(η^3-P_4Ph_2)] (**C**) with IDipp

As to get further insight in the reaction behavior of *cyclo*-P_4R_2 ligands bearing different transition metal fragments towards NHCs, reaction of the neutral [Cp'''Co(η^3-P_4Ph_2)][12] (**C**) with IDipp was carried out. Surprisingly, the crude $^{31}P\{^1H\}$ NMR spectrum (Figure S 65) of the reaction mixture shows only starting material, even after stirring for 2 h at room temperature. Additionally, a second set of signals arises due to the formation of [Cp'''Co($\eta^{2:1}$-P_4Ph_2)][12] (**C'**). However, **C'** has already been described as the thermodynamic product of a rearrangement reaction of **C** and does not result from the presence of IDipp in the reaction mixture. Full conversion to **C'** can be achieved by heating the reaction solution or stirring for extended periods of time.

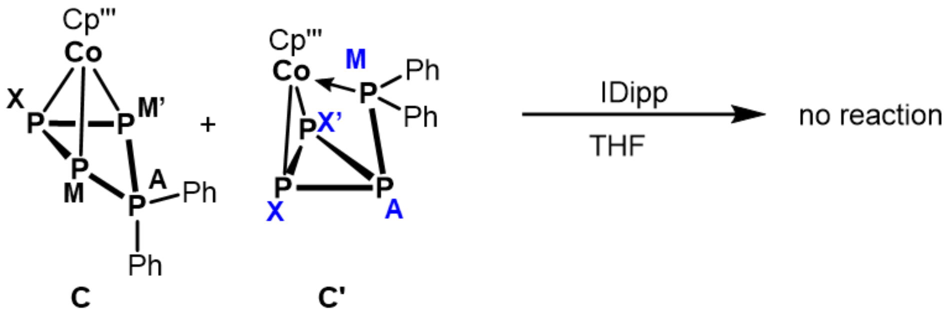

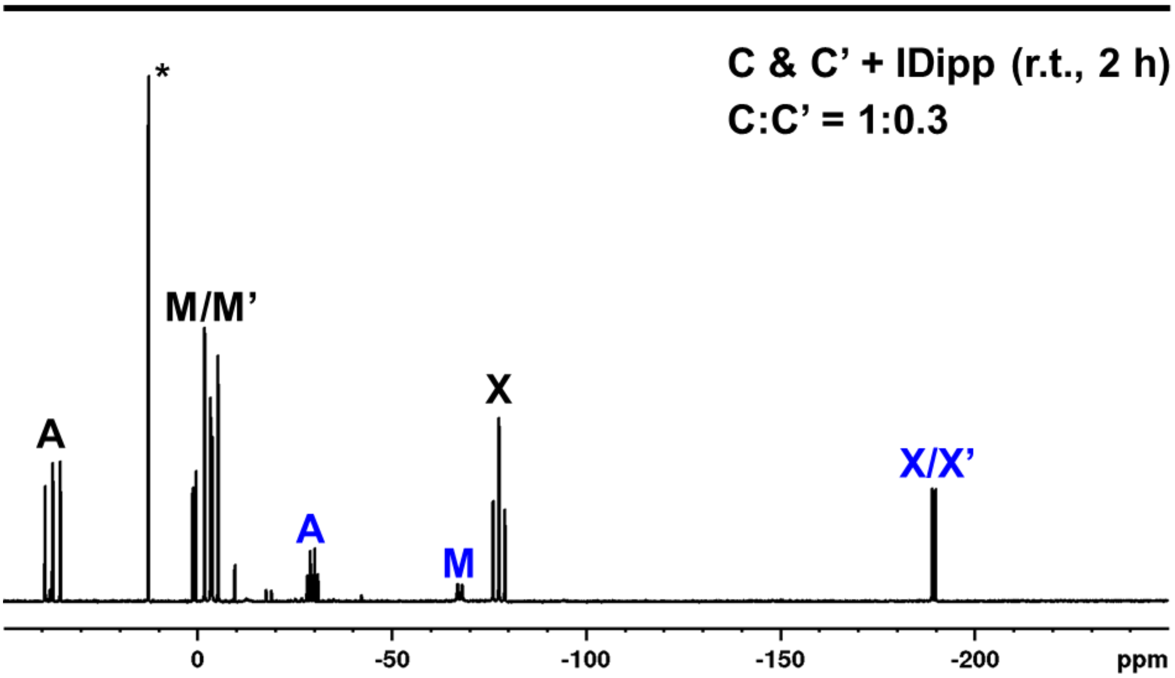

*Figure S 65: $^{31}P\{^1H\}$ NMR spectra of the reaction of **C** with IDipp in THF (C_6D_6 capillary); * marks the presence of trace amounts of $(PPh)_5$ formed as a side product during the synthesis of **C**.*

Reaction of [Cp'''Co($\eta^{2:1}$-P_4Ph_2)] (**C'**) with IDipp

Even when pure [Cp'''Co($\eta^{2:1}$-P_4Ph_2)][12] (**C'**) was reacted with IDipp no conversion was observed, even after heating to 60 °C for 3 h (Figure S 66). This is surprising, as the structure of the $\eta^{2:1}$-P_4Ph_2 ligand in **C'** is already very similar to the one observed for the imidazolyl substituted structural motif in **3a – d** and would only require simple addition of the carbene. After the substantially different structural outcome between compounds **1a – d** and **3a – d**, this further highlights the drastic impact of different, yet isolobal, TM units on the transformation of coordinated poly-phosphorus species.

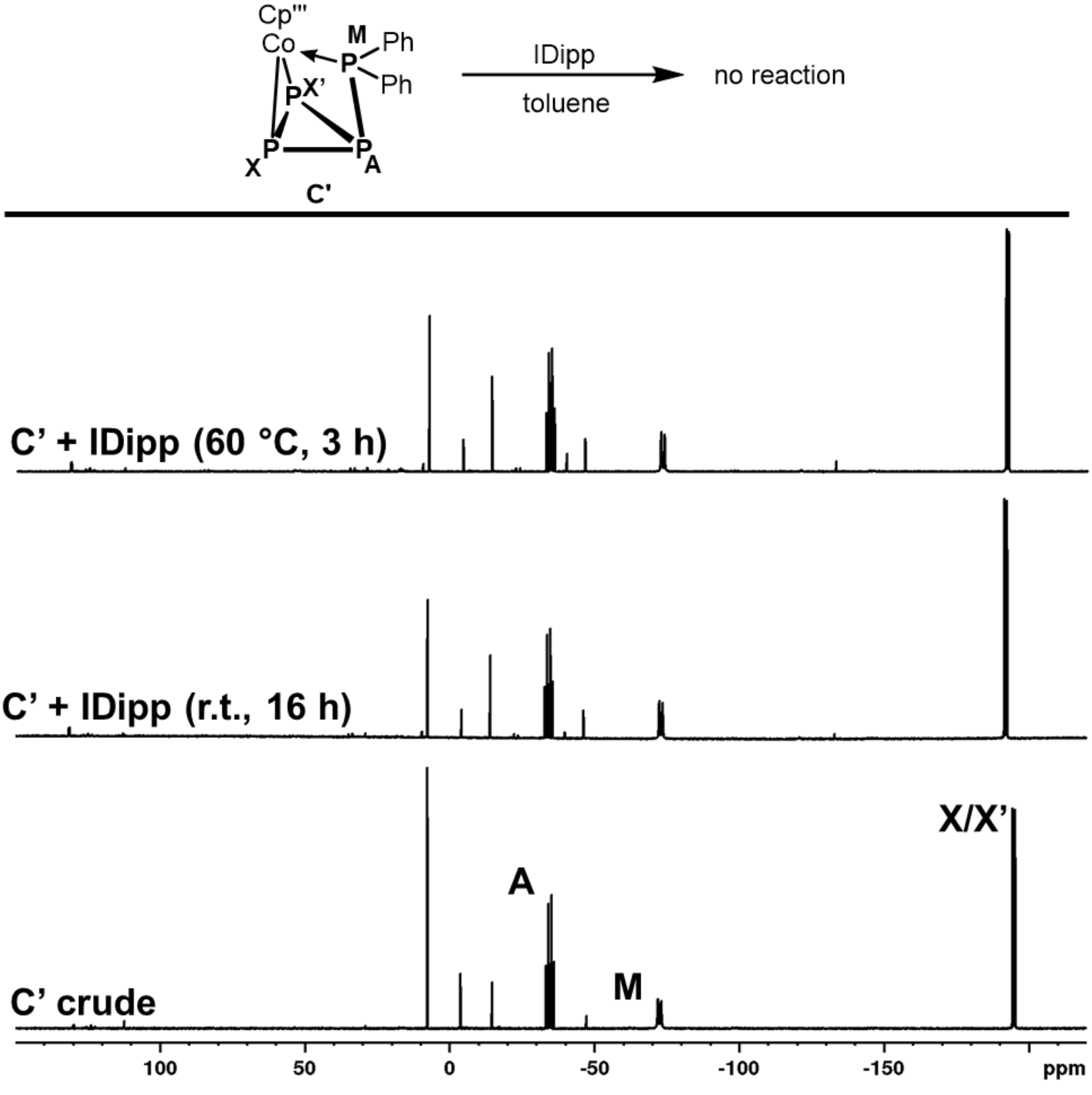

*Figure S 66: $^{31}P\{^1H\}$ NMR spectra of the reaction of **C'** with IDipp in toluene (C_6D_6 capillary); bottom: crude **C'**, middle: reaction solution after 16 h at r. t., top: reaction solution after heating to 60 °C for 3 h.*

[{Cp'''Ni}$_2$(μ,η$^{1:1:1:1}$-*cyclo*-P$_4$(PPh$_2$)$_2$)] (**6a**)

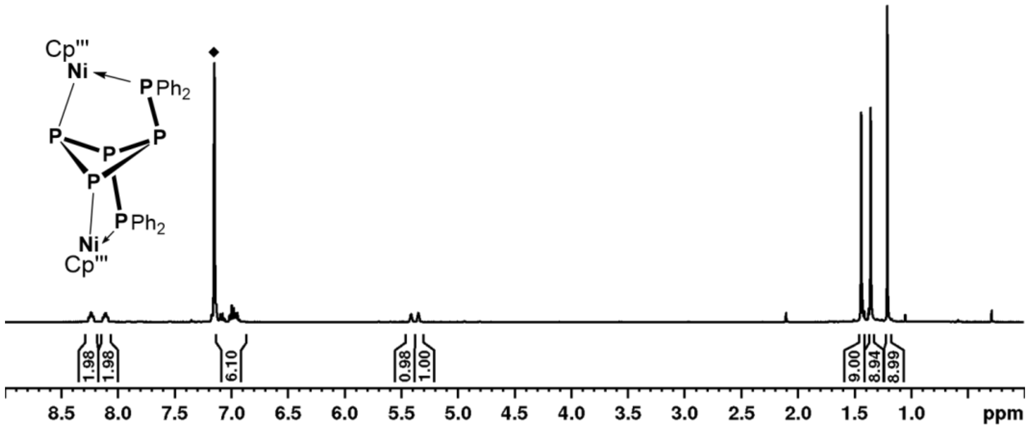

*Figure S 67: ^{1}H NMR spectrum of **6a** in C_6D_6 recorded at room temperature; ◆ = residual solvent signal of C_6D_6.*

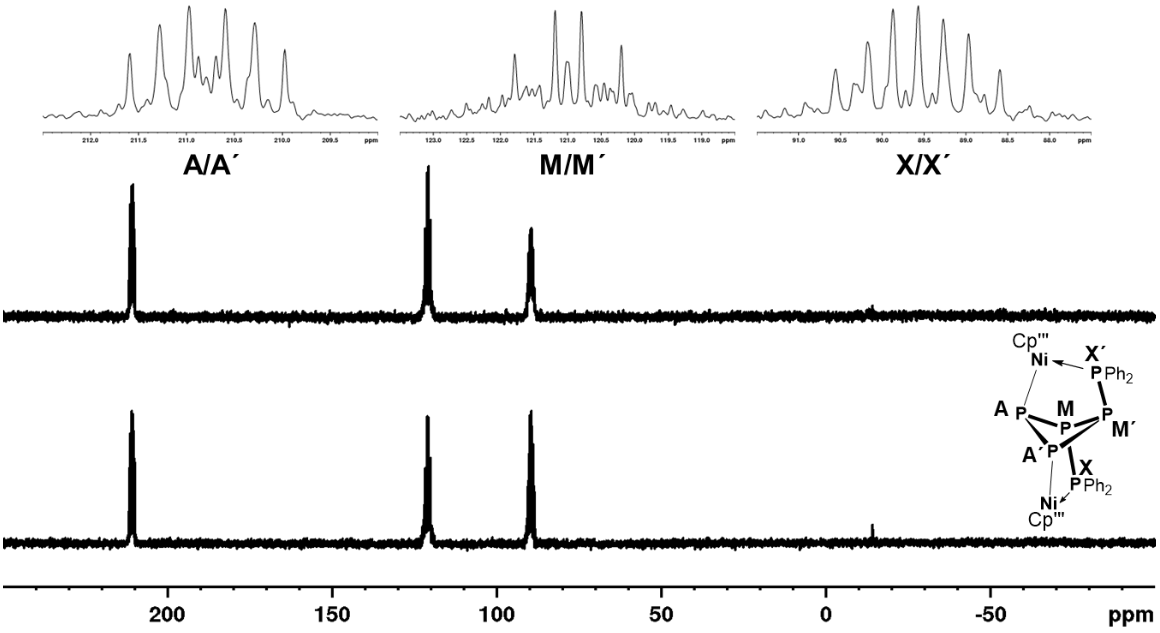

*Figure S 68: ^{31}P{^{1}H} (bottom) and ^{31}P (middle) NMR spectra of **6a** in C_6D_6 recorded at room temperature as well as enlarged signals within the measured (top) and simulated (inverted) ^{31}P{^{1}H} NMR spectrum.*

[{Cp'''Ni}$_2$(μ,$\eta^{1:1:1:1}$-*cyclo*-P$_4$(P^{i}Pr$_2$)$_2$)] (**6b**)

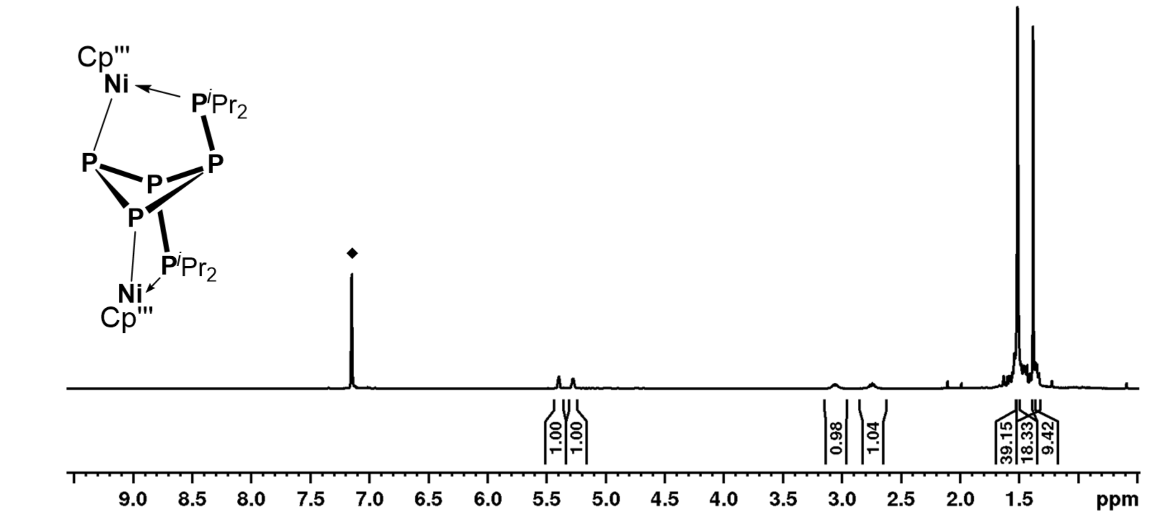

*Figure S 69: ^{1}H NMR spectrum of **6b** in C_6D_6 recorded at room temperature; ◆ = residual solvent signal of C_6D_6.*

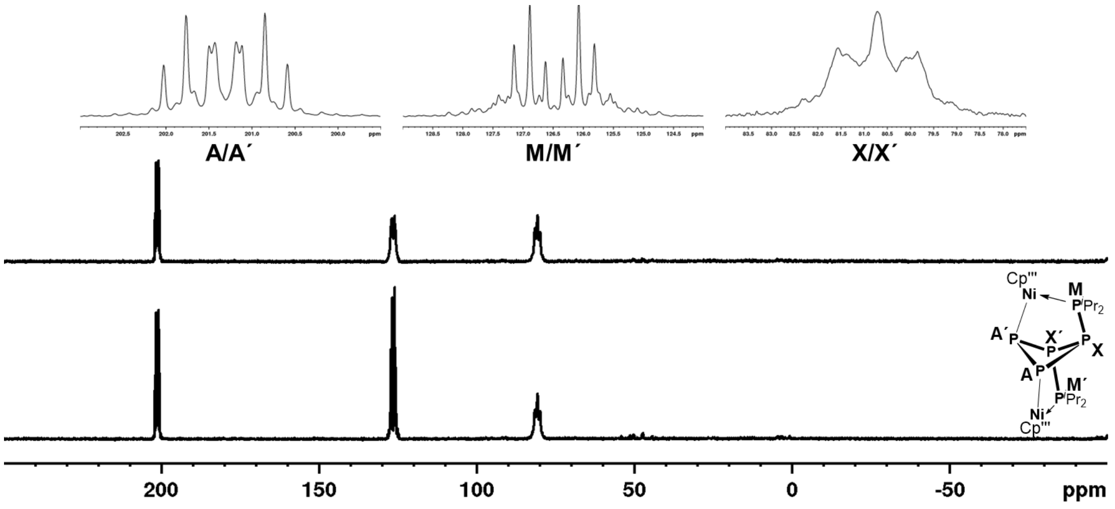

*Figure S 70: ^{31}P{^{1}H} (bottom) and ^{31}P (middle) NMR spectra of **6b** in C_6D_6 recorded at room temperature as well as enlarged signals within the measured (top) and simulated (inverted) ^{31}P{^{1}H} NMR spectrum.*

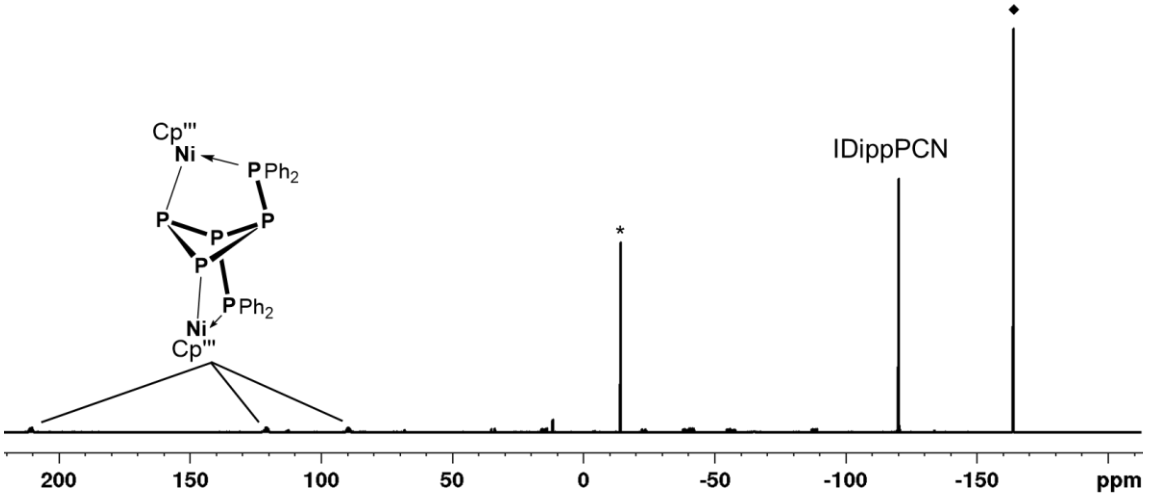

*Figure S 71: ^{31}P{^{1}H} NMR spectrum of the C_6D_6 extract of the crude reaction mixture containing equimolar amounts of **3a** and [Et$_4$N][CN]; the signal centered at δ/ ppm = –120.3 is attributed to IDippPCN; ◆ marks the presence of [Cp'''Ni(h^3-P$_3$)] and * the presence of a yet unidentified side-product.*

[{Cp'''Ni}$_2$(μ,η$^{2:2}$-(PP^{i}Pr$_2$)$_2$)] (**7**)

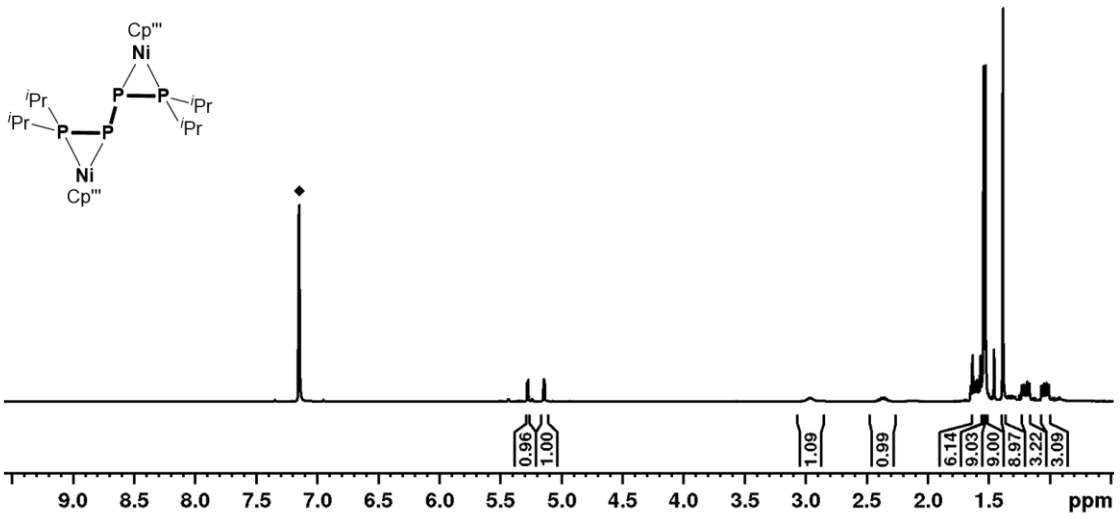

*Figure S 72: ^{1}H NMR spectrum of **7** in C_6D_6 recorded at room temperature; ◆ = residual solvent signal of C_6D_6.*

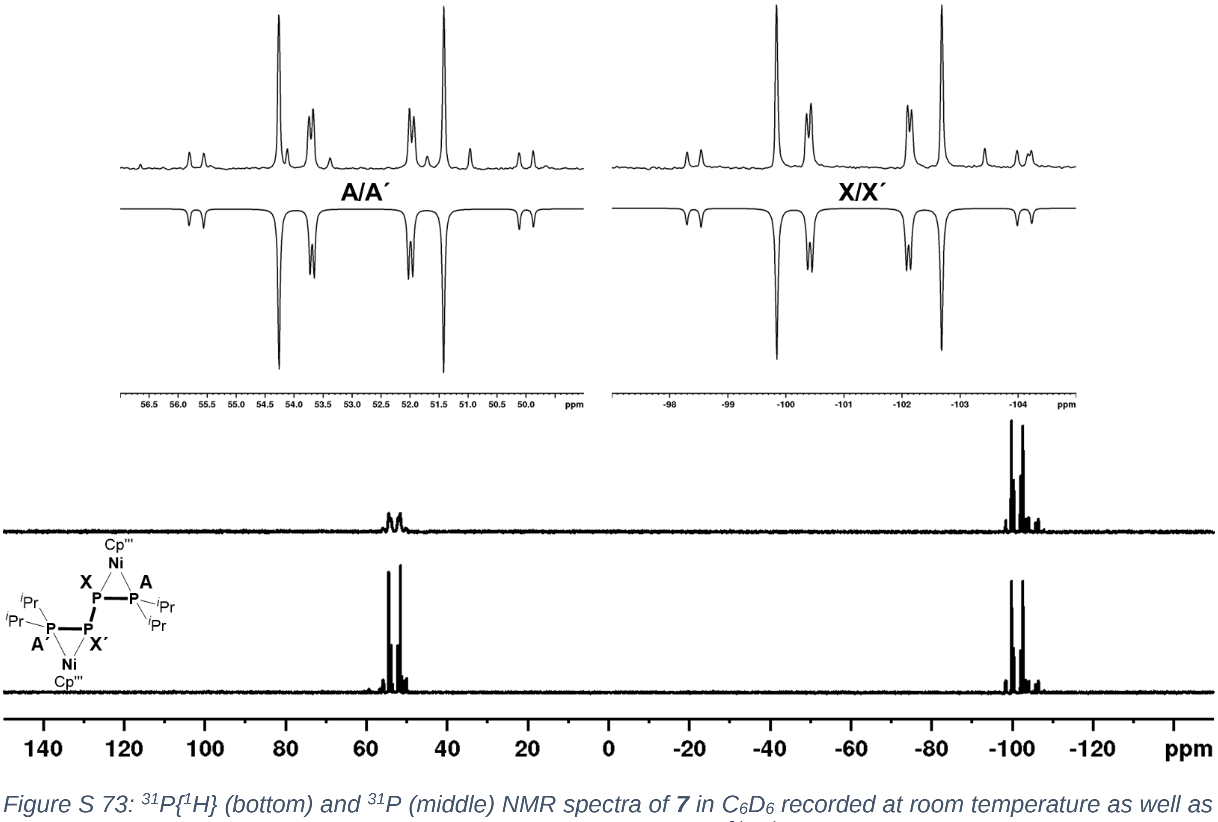

*Figure S 73: ^{31}P{^{1}H} (bottom) and ^{31}P (middle) NMR spectra of **7** in C_6D_6 recorded at room temperature as well as enlarged signals within the measured (top) and simulated (inverted) ^{31}P{^{1}H} NMR spectrum.*

[Cp'''Ni($\eta^{1:1}$-AsP_4Ph_2IDipp)] (**8b**)

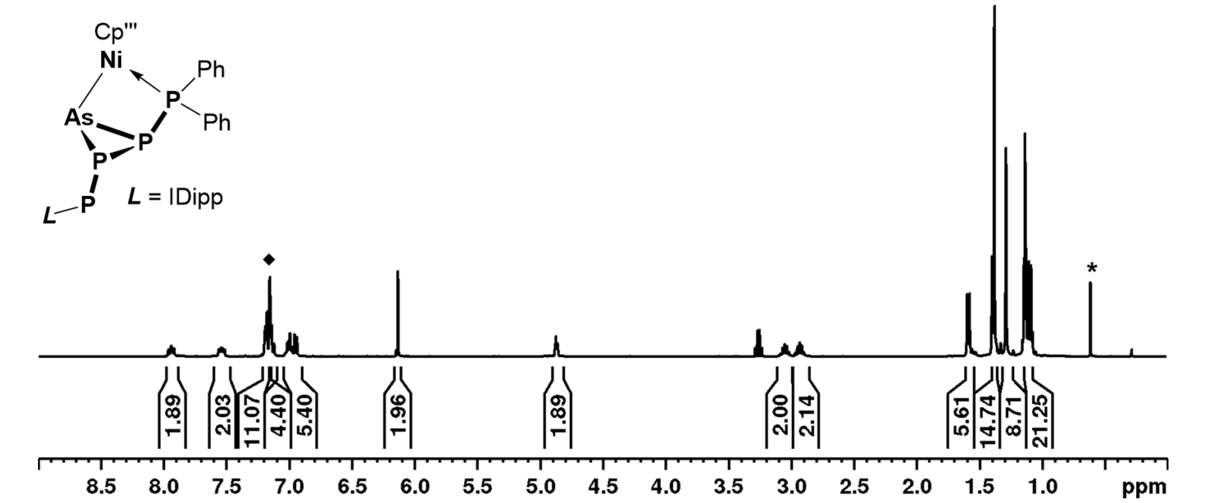

*Figure S 74: ^{1}H NMR spectrum of **8b** in C_6D_6 recorded at room temperature; ◆ = residual solvent signal of C_6D_6, * = residual MeCN from the dissolved crystals.*

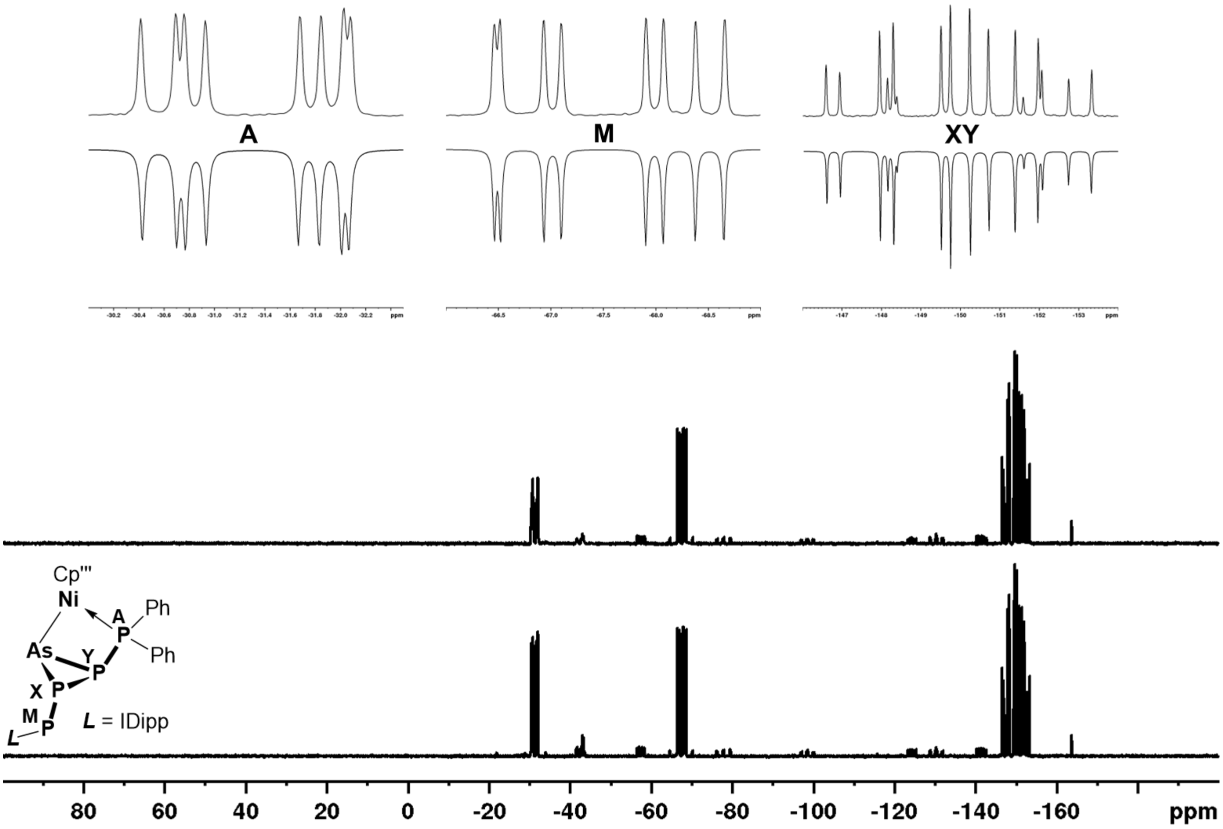

*Figure S 75: $^{31}P\{^{1}H\}$ (bottom) and ^{31}P (middle) NMR spectra of **8b** in C_6D_6 recorded at room temperature as well as enlarged signals within the measured (top) and simulated (inverted) $^{31}P\{^{1}H\}$ NMR spectrum; The smaller resonances in this spectrum are due to minor amounts of decomposition products, which form during the dissolution of **8b**.*

Computational Details

General Considerations

Quantum chemical calculations were performed using ORCA version 5.0.4 developed by Neese *et al*.[13] Images of calculated structures were generated with ChemCraft.[14] Molecular geometries for the NHC and KOEt reaction pathways were optimized at the *ω*B97X-D3BJ/def2-TZVP level of theory including implicit solvent correction (CPCM=THF) and the methylation pathway at the *ω*B97X-D3BJ/def2-TZVP // BP86-D3BJ/def2-TZVP (CPCM=CH_2Cl_2) level of theory due to the size of the molecular structures.[15] For all structures, subsequent analytical frequency analysis was carried out in order to determine the nature of stationary points on the potential energy surface. *Natural Bond Orbital* analysis (NBO 7.0)[16] was carried out at the abovementioned level of theory and used to describe electronic densities and to calculate the *Wiberg Bond Index* matrix (WBI).[17] Approximate transition states were found using the *Nudged Elastic Band* method (NEB-TS) as implemented in ORCA followed by a geometry optimization and frequency analysis of the saddle point.[18]

Frontier Molecular Orbital Analysis

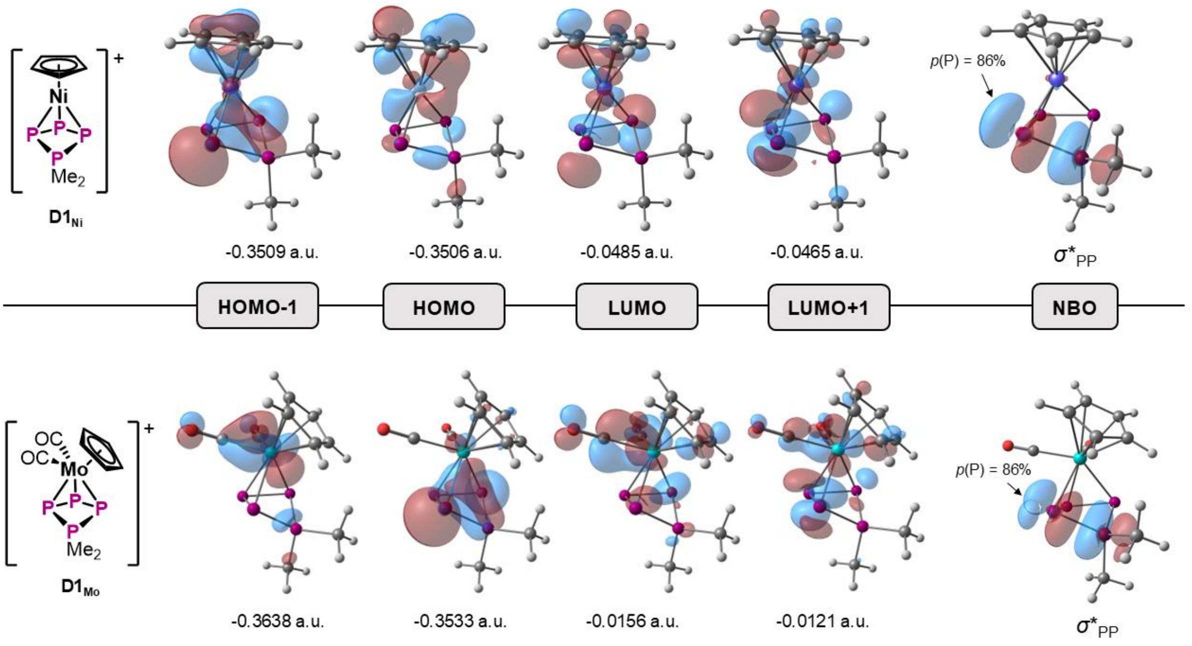

*Figure S 76: Depiction of the frontier Kohn-Sham orbitals and selected NBOs of $[CpNi(P_4Me_2)]^+$ (**$D1_{Ni}$**) and $[CpMo(CO)_2(P_4Me_2)]^+$ (**$D1_{Mo}$**, ωB97X-D3BJ/def2-TZVP level of theory).*

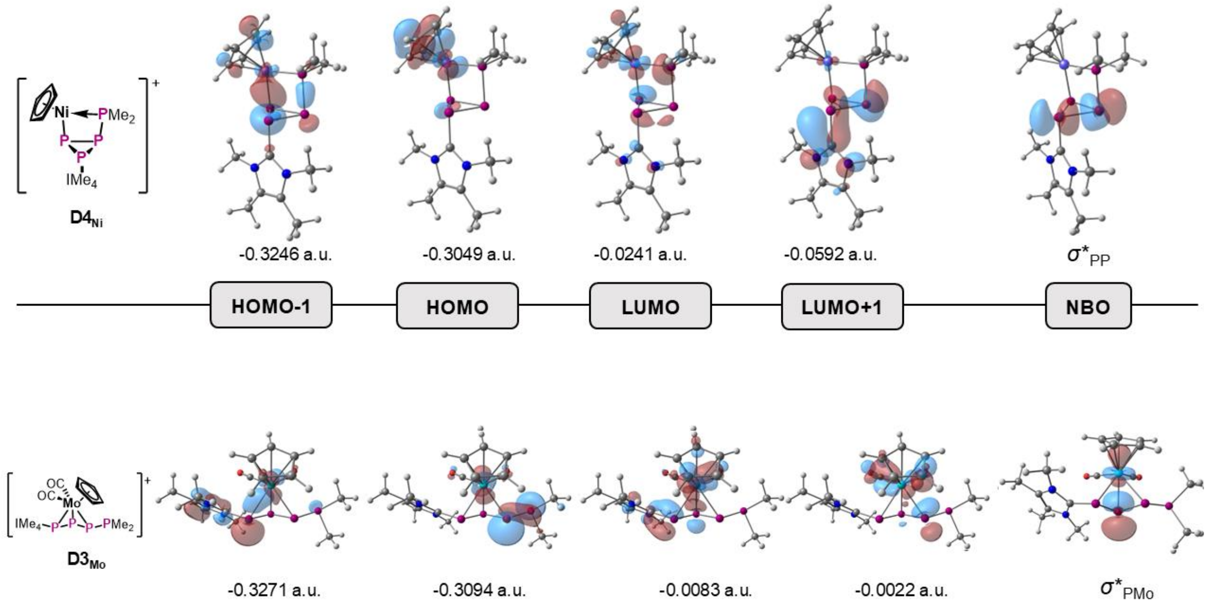

Figure S 77: Depiction of the frontier Kohn-Sham orbitals and selected NBOs of ***D4$_{Ni}$*** *and* ***D3$_{Mo}$*** *(ωB97X-D3BJ/def2-TZVP level of theory).*

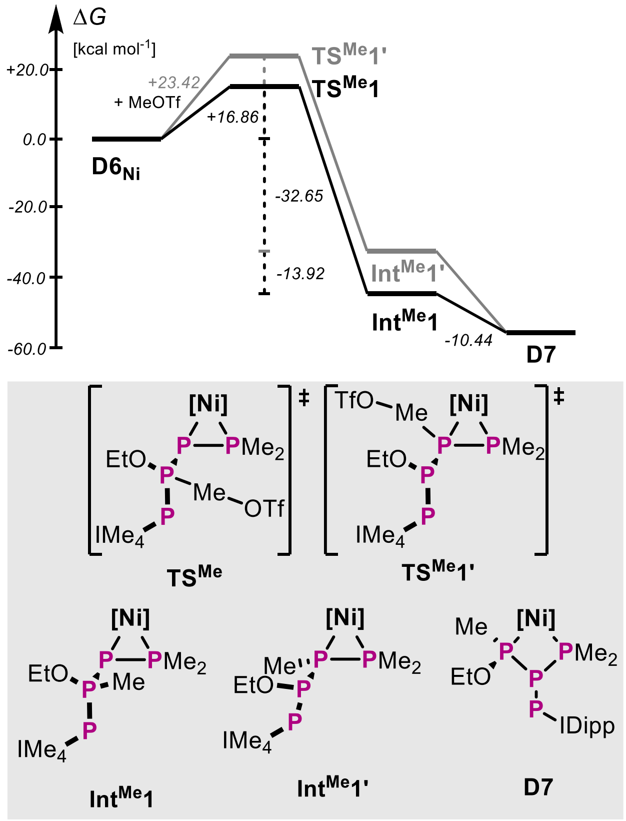

Figure S 78: Calculated reaction pathway for the methylation of ***D6$_{Ni}$*** *(ωB97X-D3BJ/def2-TZVP // BP86-D3BJ/def2-TZVP level of theory).*

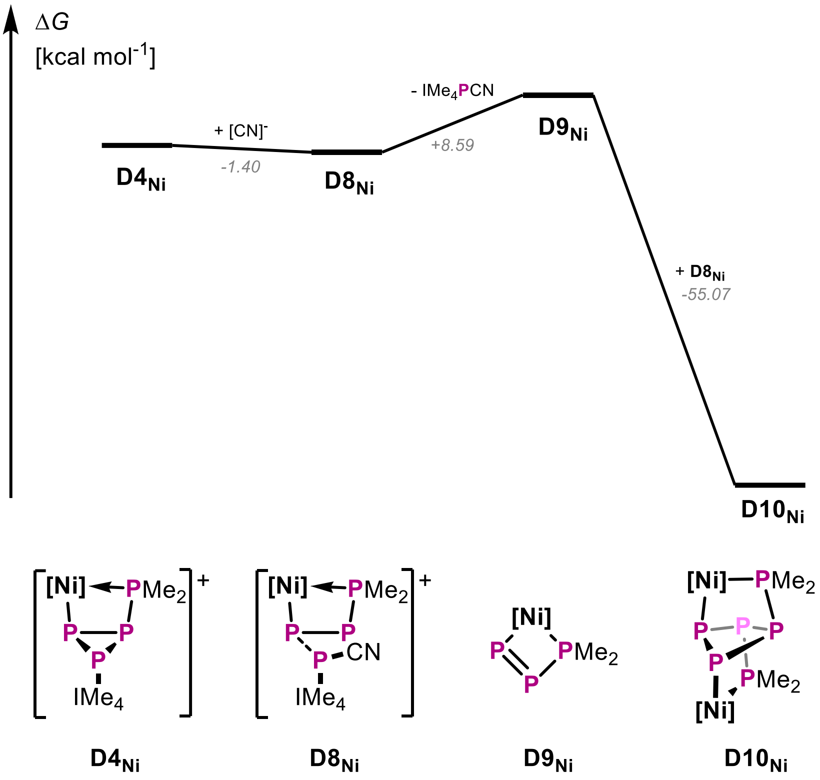

*Figure S 79: Calculated reaction pathway of the reaction of **D4$_{Ni}$** and [CN]$^{–}$ (ωB97X-D3BJ/def2-TZVP level of theory, CPCM = THF).*

Thermochemistry of individual compounds

Table S 1: Summary of thermodynamic data for all investigated individual compounds (values given in /Eh).

Compound	E^0_{el}	U^0_{298}	H^0_{298}	S^0_{298}	G^0_{298}
$[OEt]^-$	-154.63593715	-154.56632768	-154.56538347	0.02970547	-154.59508894
$[OTf]^-$ (CPCM=CH2Cl2)	-962.00817795	-961.97504524	-961.97410103	0.04043849	-962.01453952
	Refined Single Point Energy: -961.999175866573				
D1 (Mo)	-1934.25495147	-1934.04823673	-1934.04729252	0.06349983	-1934.11079235
D1 (Ni)	-3147.39948782	-3147.21519830	-3147.21425409	0.05503347	-3147.26928756
D2 (Mo)	-2318.06231564	-2317.65715703	-2317.65621282	0.08645298	-2317.74266579
D2 (Ni)	-3531.21109945	-3530.82802332	-3530.82707911	0.07844926	-3530.90552837
D3 (Mo)	-2318.06716851	-2317.66196559	-2317.66102138	0.08668969	-2317.74771107
D3' (Mo)	-2318.04901540	-2317.64301334	-2317.64206913	0.08515781	-2317.727537
D3 (Mo) (IDipp and PPh2)	-3479.17752372	-3478.24718349	-3478.24623928	0.13675897	-3478.382998
D3 (Ni)	-3531.21650115	-3530.83359071	-3530.83264650	0.07876534	-3530.91141184
D3' (Ni)	-3531.21165792	-3530.82820023	-3530.82725602	0.07732686	-3530.904583
D3 (Ni) (IDipp and PPh2)	-4692.31997730	-4691.41266322	-4691.41171901	0.12782898	-4691.539548
D4 (Mo)	-2318.06808917	-2317.66294082	-2317.66199661	0.08640558	-2317.74840219
D4 (Ni)	-3531.23996530	-3530.85738191	-3530.85643770	0.07964428	-3530.93608199
D4 (Ni) (IDipp and PPh2)	-4692.34866046	-4691.44187453	-4691.44093032	0.12712233	-4691.568053
D4(Mo) (IDipp and PPh2)	-3479.17170470	-3478.24125422	-3478.24031001	0.13592372	-3478.376234
D5 (Mo)	-2472.78627484	-2472.30634091	-2472.30539670	0.09661222	-2472.40200892
D5' (Mo)	-2472.77943053	-2472.29969795	-2472.29875374	0.09676661	-2472.39552035
D5' (Ni)	-3685.94464872	-3685.48736955	-3685.48642534	0.09058493	-3685.57649073
D5' (Ni)	-3685.95404930	-3685.49640265	-3685.49545844	0.08848719	-3685.58394563
D6 (Mo)	-2472.77533955	-2472.29575169	-2472.29480748	0.09889980	-2472.39370728
D6 (Ni)	-3685.93757136	-3685.48008956	-3685.47914536	0.09021668	-3685.57701027
D6 (Ni) (CPCM=CH2Cl2)	-3686.04676180	-3685.60646574	-3685.60552153	0.09313026	-3685.69865179
	Refined Single Point Energy: -3685.934045127093				
D7 (CPCM=CH2Cl2)	-3725.86677072	-3725.38721254	-3725.38626834	0.09361130	-3725.47987964
	Refined Single Point Energy: -3725.787646244726				
IMe_4	-383.77061519	-383.57643864	-383.57549443	0.04546700	-383.62096143
$Int^{Me}1$ (CPCM=CH2Cl2)	-3725.85211176	-3725.37124273	-3725.37029852	0.09547442	-3725.46577294
	Refined Single Point Energy: -3725.770449104203				
$Int^{Me}1'$ (CPCM=CH2Cl2)	-3725.83309136	-3725.35394829	-3725.35300408	0.09521999	-3725.44822407
	Refined Single Point Energy: -3725.746805381213				
MeOTf (CPCM=CH2Cl2)	-1001.74631017	-1001.67232152	-1001.67137731	0.04552330	-1001.71690061
	Refined Single Point Energy: -1001.758910504013				
TS1 (Mo)	-2318.00718461	-2317.60412005	-2317.60317584	0.08416345	-2317.68733929
TS1' (Mo)	-2318.01052674	-2317.60643625	-2317.60549204	0.08515781	-2317.69065
TS1 (Ni)	-3531.15950948	-3530.77890083	-3530.77795662	0.07642012	-3530.85437674
TS1' (Ni)	-3531.17004291	-3530.78833833	-3530.78739413	0.07909470	-3530.866489
TS2 (Mo)	-2317.97446064	-2317.57097458	-2317.57003037	0.08715784	-2317.657188
TS2 (Ni)	-3531.17799984	-3530.79674380	-3530.79579959	0.07759656	-3530.873396
$TS^{Me}1$ (CPCM=CH2Cl2)	-4687.79930606	-4687.28386651	-4687.28292230	0.11469618	-4687.39761848
	Refined Single Point Energy: -4687.690258756327				
$TS^{Me}1'$ (CPCM=CH2Cl2)	-4687.78928444	-4687.27550030	-4687.27455609	0.11490368	-4687.38945977
	Refined Single Point Energy: -4687.677938503774				

D8 (Ni)	-3624.27688608	-3623.88397599	-3623.88303178	0.08495194	-3623.96798372
D9 (Ni)	-2806.13422505	-2805.95460175	-2805.95365754	0.05449779	-2806.00815533
D10 (Ni)	-5612.38535700	-5612.02265812	-5612.02171391	0.08235126	-5612.10406517
$[CN]^-$	-93.01561731	-93.00827710	-93.00733289	0.02233401	-93.02966691
IMe_4-PCN	-818.10191326	-817.89160623	-817.89066202	0.05323716	-817.94389918

Coordinates of Optimized Geometries

Table S 2: Cartesian coordinates of all optimized structures.

**D1 (Ni)**			
28	0.67187732	16.9713718	6.3698074
15	1.99839799	19.4944629	5.91751215
15	0.46811608	18.5198161	4.76582927
15	1.12772461	18.7043363	7.71684326
15	-0.69063918	18.6958715	6.56838246
6	1.69620644	15.4133709	5.47988562
1	2.36481513	15.5186658	4.63896453
6	0.27325834	15.1922588	5.40033815
6	-0.23857725	15.0874142	6.71662106
1	-1.27167984	14.9329166	6.98862904
6	0.8337448	15.3283811	7.61110902
6	2.04201605	15.4955446	6.84316334
1	-0.29583699	15.1061668	4.48679876
1	0.76745092	15.3635856	8.68832541
1	3.02663751	15.674016	7.24798281
6	1.96285005	21.2890411	5.81296711
1	2.65890176	21.6976113	6.54862236
1	2.26894234	21.5890307	4.80867308
1	0.95180076	21.6403189	6.01837405
6	3.68418698	18.9743402	5.58145316
1	4.34724814	19.4432697	6.31209074
1	3.75194764	17.8897018	5.65613062
1	3.9606339	19.299693	4.57625393
**D1 (Mo)**			
42	9.35145414	7.47649356	21.5699534
15	12.2165685	6.82359606	23.0438815
15	10.5059572	5.57313343	22.7188853
15	9.55457791	7.04913428	23.9987343
15	10.9340961	8.5312883	23.2075219
8	7.48930621	9.76031107	22.7031152
8	6.86111193	5.60507592	22.0458339
6	9.48914387	8.8974788	19.7750482
1	9.29965468	9.95615626	19.8676009
6	9.19776212	6.63925384	19.4466458
1	8.74985518	5.67843303	19.2434247
6	10.5786979	6.8788115	19.6718586
1	11.35039	6.12543515	19.6690739
6	10.7580768	8.27030519	19.8752901
1	11.691671	8.77755317	20.0615757
6	8.51589146	7.89111588	19.5018896
1	7.46164927	8.05097249	19.3362633
6	7.76477869	6.27350467	21.8957843
6	8.16479868	8.93740582	22.3145277
6	13.096938	6.44876253	24.5700249
1	12.3869637	6.38607194	25.3944384
1	13.6200995	5.49690084	24.4554145
1	13.8219547	7.24252933	24.764251
6	13.5006149	6.85260892	21.7840823
1	14.2391944	7.60394318	22.07277
1	13.9775035	5.87041807	21.75721
1	13.0993332	7.09232622	20.8037168
**IMe_4**			
7	6.77102394	-4.95164674	11.9246226
6	6.73267993	-3.80812448	12.6547182
6	6.75639558	-6.10095643	12.7143251
6	6.70490259	-5.6680021	13.9965655
7	6.69225676	-4.27515673	13.928206
6	6.81883869	-4.97670097	10.4745445
1	7.72771678	-5.47240246	10.1262221
1	5.95213313	-5.50354872	10.0691464
1	6.81177268	-3.94748981	10.1217893
6	6.79535837	-7.47581652	12.1389546
1	7.69852955	-7.63107332	11.5418412
1	6.78555392	-8.2177572	12.9377805
1	5.93322062	-7.6609853	11.4916419
6	6.66343399	-6.41329175	15.2872329
1	5.75703409	-6.17885628	15.8530356
1	6.67809234	-7.48744866	15.1012937
1	7.5217751	-6.16508107	15.9182879
6	6.63966886	-3.41649716	15.0969239
1	5.7272902	-3.59874649	15.6694402

1	7.50256553	-3.59148374	15.7434425
1	6.64923611	-2.38383134	14.7541885

D2 (Mo)

42	1.4597386	13.7085017	21.025061
15	1.40746458	15.8235032	19.6778926
15	-0.28830853	15.5172733	20.9284741
15	-1.25314203	14.0237753	18.3535808
15	0.93690491	14.0574213	18.5880452
7	-0.05718589	16.6571078	23.5336759
6	1.6238573	11.4392043	20.8657483
1	2.24573448	10.9720208	20.1169611
7	0.2798619	18.1222672	21.998664
6	0.39235583	18.7873086	23.2150541
6	0.23899758	11.7350912	20.7288264
1	-0.37296783	11.5247293	19.8668793
6	-1.43200508	15.5097455	17.2817853
6	2.03568702	11.8348415	22.1733548
1	3.01916229	11.7089461	22.5995155
6	0.03097016	16.8178155	22.2040492
6	0.17878197	17.8602954	24.1849989
6	-1.22976036	12.7210108	17.0449749
6	0.89992727	12.3920611	22.827453
1	0.88015641	12.7673669	23.8386696
8	4.30412735	13.6183033	19.7063316
8	2.86153486	15.6061853	23.1099374
6	3.27191411	13.6726936	20.1797492
6	-0.20409166	12.3248303	21.9358815
1	-1.20701744	12.6697982	22.135381
6	2.33888026	14.9410486	22.3490424
6	0.37248829	18.7929386	20.7046988
1	1.41217771	18.8607006	20.3855578
1	-0.20393802	18.2368129	19.9701469
1	-0.04720111	19.7916367	20.8035723
6	-0.37032426	15.4162555	24.2276716
1	-0.71161308	14.6818706	23.5044335
1	0.51431559	15.0452579	24.7462169
1	-1.16408199	15.6019293	24.9493431
6	0.17505378	17.9679252	25.6693117
1	-0.80297831	17.7064546	26.0808599
1	0.91911432	17.2997085	26.1104115
1	0.4105039	18.9887756	25.9667065
6	0.68661592	20.2437789	23.3053316
1	-0.12926341	20.8395722	22.8882052
1	0.81607859	20.5270314	24.3490068
1	1.60241168	20.4931678	22.7641414
1	-0.66041897	15.5448402	16.5085423
1	-1.3662787	16.408913	17.8967174
1	-2.41508051	15.480381	16.8047528
1	-2.24127078	12.6191527	16.6440497
1	-0.54459706	12.9710057	16.2314421
1	-0.93496011	11.7619034	17.4761914

D5 (Mo)

42	12.833397	5.16063	5.39327
15	11.388216	6.770293	4.058732
15	10.448728	4.379685	5.172843
15	10.202294	2.231863	4.82674
15	10.632596	4.971614	3.114852
8	13.51442	2.322046	4.366592
7	12.030553	7.766412	1.416217
8	14.699168	5.615652	2.964127
7	13.323289	8.509979	2.96018
8	9.101323	5.223818	2.500142
6	12.297759	7.66975	2.731826
6	14.396511	5.045522	7.059156
1	15.327364	4.522414	6.901605
6	13.192792	4.48337	7.572443
1	13.045033	3.455813	7.867957
6	13.989465	5.459969	3.867022
6	12.231417	5.527148	7.673022
1	11.214162	5.418924	8.015798
6	13.198324	3.373015	4.728756
6	12.917788	8.649752	0.803397
6	13.732393	9.119221	1.779058
6	14.160665	6.426945	6.820454
1	14.886355	7.134976	6.452383
6	12.825769	6.719952	7.210695
1	12.339128	7.681691	7.141948
6	13.932579	8.781134	4.251916
1	13.257084	8.457709	5.037511
6	10.938681	7.112558	0.705715
1	10.069791	7.047016	1.353414
6	12.877327	8.947535	-0.655659
1	12.951787	8.032433	-1.247907
1	13.712981	9.594704	-0.91948
1	11.951804	9.458722	-0.933628
6	14.856714	10.094346	1.737217
1	14.624529	10.989316	2.320628
1	15.043493	10.398768	0.708138
1	15.773461	9.655908	2.13936
1	14.103938	9.852593	4.344119
1	14.879554	8.248419	4.339148
6	8.095165	5.929921	3.225936
1	8.46121	6.935897	3.461467
1	7.908472	5.417758	4.174997

1	10.685494	7.714227	-0.16409
1	11.230595	6.112471	0.386965
6	6.836567	5.993096	2.384622
1	7.02818	6.506046	1.438572
1	6.056529	6.539172	2.922083
1	6.467794	4.987756	2.166265
6	8.358612	2.214356	4.763709
1	8.032228	2.722659	3.853263
1	8.004325	1.180622	4.725548
1	7.917871	2.717585	5.628331
6	10.464768	1.641104	6.553018
1	9.906041	2.24154	7.276172
1	10.143893	0.599144	6.629982
1	11.528814	1.691966	6.79298

[OEt]$^-$

6	11.151044	6.427999	2.502585
1	11.151039	7.457981	2.120541
1	12.036539	5.912834	2.112732
1	10.265551	5.912827	2.112733
6	11.151043	6.377456	4.04358
1	10.269279	6.992744	4.369054
8	11.15104	5.116088	4.551516
1	12.032811	6.992739	4.369054

D2 (Ni)

28	1.89514843	14.557254	19.7712454
15	2.35637694	16.38542	18.6102976
15	0.4042948	16.1819279	19.3965829
15	0.08705472	14.4424902	16.7783383
15	2.15049163	14.5576052	17.5496478
7	-0.14352818	16.9763701	22.0323896
6	3.32449745	13.1343948	20.2678655
1	4.15517818	12.8690082	19.631214
7	1.06247145	18.4698887	21.0616307
6	0.82545612	18.9257321	22.3545372
6	2.01455329	12.5336467	20.2222019
1	1.70970403	11.7414136	19.5564266
6	0.55189851	13.7256389	15.1448614
6	3.33852433	14.1058275	21.29574
1	4.17247665	14.7350441	21.5676134
6	0.4888247	17.2692222	20.8840533
6	0.06195256	17.9820852	22.9661182
6	-0.5026669	12.9203421	17.6259947
6	2.02715393	14.1853654	21.819706
1	1.70402903	14.8588957	22.5988843
6	1.21410322	13.1810487	21.1801751
1	0.17555748	12.9759914	21.39005
6	1.78809575	19.24649	20.0596928
1	2.85580667	19.0367796	20.1156383
1	1.41082483	19.0002579	19.0708537
1	1.61036882	20.3025138	20.2493596
6	-0.98877066	15.8137323	22.2800555
1	-1.99963713	16.1500776	22.5094099
1	-1.01164434	15.1885283	21.3916498
1	-0.59496356	15.2420178	23.1197859
6	-0.50155564	17.9082592	24.3417078
1	-1.59221322	17.843685	24.3161992
1	-0.12188303	17.0313064	24.8722939
1	-0.22486808	18.7985399	24.9042881
6	1.3653891	20.2190784	22.8573353
1	0.92138621	21.0671984	22.3299366
1	1.13992327	20.3223318	23.9178239
1	2.44935558	20.2666371	22.7297793
1	1.16817912	12.8295357	15.2475714
1	1.09464851	14.4717906	14.5612371
1	-0.36573114	13.4707828	14.6085141
1	-1.41354733	12.5892087	17.1203826
1	0.23566747	12.1170049	17.5911574
1	-0.74075057	13.1491532	18.6653612

D3 (Mo)

42	3.292769	5.239694	3.858154
15	2.728075	2.793813	3.542496
15	2.115662	3.652013	5.395748
15	0.837775	4.786277	4.138818
15	0.020176	6.147477	5.650341
7	5.234039	1.601113	3.105725
8	3.247183	7.024915	6.449045
8	5.861463	4.124453	5.287227
7	4.536301	1.106452	5.077372
6	4.224929	1.800115	3.970039
6	6.214648	0.804568	3.679468
6	5.776943	0.494327	4.927466
6	3.193629	6.354426	5.532642
6	4.912959	4.505922	4.786326
6	3.261867	7.278218	2.796658
1	2.880995	8.166638	3.276637
6	4.603249	6.80436	2.863228
1	5.420852	7.275431	3.386676
6	2.520255	6.40262	1.960308
1	1.47653	6.501745	1.707591
6	4.675016	5.614936	2.079573
1	5.563058	5.028228	1.906915
6	3.387325	5.376838	1.521239
1	3.112259	4.55746	0.875635
6	3.713969	0.967079	6.275678

1	2.664588	0.975163	5.99481
1	3.946973	0.013369	6.742736
1	3.919377	1.778206	6.974218
6	6.408078	-0.317319	6.004504
1	5.824389	-1.215299	6.221926
1	7.403978	-0.627019	5.690676
1	6.502374	0.261474	6.926391
6	7.458974	0.437726	2.950225
1	8.096179	-0.173195	3.587787
1	7.231151	-0.132088	2.045888
1	8.017631	1.330273	2.657288
6	5.308065	2.094889	1.736365
1	6.166075	2.758078	1.630882
1	5.413885	1.249859	1.056695
1	4.394319	2.630781	1.496995
6	-1.692326	5.473583	5.675129
1	-2.115299	5.394381	4.670662
1	-1.685076	4.486249	6.141249
1	-2.320648	6.134112	6.278145
6	-0.239658	7.624659	4.58177
1	-0.895612	8.320749	5.110406
1	0.71425	8.123093	4.403485
1	-0.696856	7.364741	3.623717

D5' (Ni)

28	-0.351719	12.696763	18.268994
15	-2.093287	12.166497	19.537464
15	0.605415	12.567489	20.149136
15	-3.779539	13.342187	18.836088
15	-1.156251	13.369242	21.09262
8	-1.368045	12.547607	22.521295
7	-6.169874	11.866899	18.630781
7	-5.324625	11.698837	20.603282
6	-5.11164	12.250405	19.385329
6	0.227285	11.913024	16.341297
6	-1.048221	12.519894	16.301137
1	-1.979574	12.036362	16.045008
6	0.946656	10.91782	20.827538
1	1.192867	10.993114	21.888403
6	1.138546	12.89973	16.803945
1	0.45159	10.8743	16.14886
6	-0.900899	13.921368	16.607853
1	0.061154	10.295098	20.701137
6	0.44748	14.157765	16.9005
1	0.887131	15.096246	17.205222
1	-1.705857	14.640169	16.641278
6	2.098199	13.537717	20.508021
1	2.93322	13.14182	19.925555
6	-7.041822	11.072635	19.370902
1	1.783798	10.470927	20.287433
6	-6.502586	10.953932	20.608019
6	-1.125777	13.271822	23.734098
1	-1.356035	14.332632	23.581149
1	-0.062586	13.191149	23.988085
1	2.193207	12.75104	16.988454
1	1.923129	14.574671	20.219064
6	-1.985252	12.689836	24.836877
1	-1.769803	11.626994	24.971153
1	-3.04528	12.805418	24.597839
1	-1.782478	13.205907	25.779081
1	2.340224	13.487284	21.571534
6	-6.370789	12.227731	17.238346
1	-5.397171	12.45715	16.807309
1	-6.826601	11.391675	16.710027
1	-7.014637	13.105555	17.155895
6	-4.524511	11.97112	21.785456
1	-5.167689	11.935127	22.663384
1	-3.711378	11.255357	21.891516
1	-4.098708	12.968381	21.683297
6	-8.291404	10.509932	18.787288
1	-8.072574	9.809164	17.976955
1	-8.853293	9.979047	19.555129
1	-8.927532	11.30235	18.383995
6	-6.968595	10.204666	21.809019
1	-7.860231	9.628714	21.563238
1	-6.199032	9.512353	22.16015
1	-7.215573	10.87906	22.633314

D6 (Mo)

15	9.312823	23.665643	7.519929
15	7.483084	24.782311	6.999939
15	6.69072	23.258016	5.695019
15	8.617061	22.845752	9.311502
8	8.105957	26.017627	6.078936
7	4.013882	22.664939	6.339101
7	4.259902	24.765238	5.945625
6	2.756491	23.24532	6.481178
6	4.938659	23.598532	6.014773
6	2.913513	24.570066	6.244916
6	9.015788	25.771975	5.010239
1	8.637167	24.948306	4.394221
1	9.983719	25.466963	5.425428
6	6.852845	22.943907	9.709035
1	6.516717	23.976403	9.637885
6	9.158229	27.03905	4.192169
1	9.866413	26.880267	3.374571

1	9.525233	27.858931	4.814618
1	8.19501	27.329431	3.764857
1	6.278176	22.321265	9.021145
6	9.009563	21.07702	9.434314
1	8.277799	20.495399	8.869973
1	6.715913	22.586181	10.732663
1	10.004544	20.904462	9.024534
1	8.992279	20.768481	10.482183
6	4.821323	26.042184	5.534525
1	5.088975	26.646886	6.40134
1	5.71645	25.850805	4.946485
1	4.087	26.572432	4.929532
6	1.933091	25.690776	6.271331
1	1.802379	26.131857	5.279615
1	0.964375	25.326196	6.611898
1	2.261241	26.481224	6.951235
6	1.550134	22.450461	6.844614
1	1.375081	21.648017	6.123242
1	1.656177	21.997123	7.833868
1	0.672279	23.095561	6.857648
6	4.278412	21.246322	6.511871
1	5.349555	21.117246	6.655901
1	3.739716	20.881449	7.385778
1	3.960972	20.688748	5.628855
42	10.247053	24.513435	9.751406
8	8.144777	25.416035	11.842154
8	9.4833	27.283671	8.574584
6	8.925118	25.069197	11.059048
6	9.735069	26.232957	8.97509
6	11.813736	23.943061	11.36547
1	11.530597	23.781377	12.394814
6	12.183894	25.188238	10.782205
1	12.25059	26.136325	11.293385
6	11.912069	22.941739	10.364804
1	11.703078	21.890838	10.497944
6	12.499933	24.94674	9.411718
1	12.833419	25.683038	8.695704
6	12.337311	23.559632	9.162338
1	12.490037	23.062404	8.215965

D6 (Ni, CPCM = THF)

28	10.185555	24.023339	9.298107
15	9.338987	22.974623	7.512895
15	7.537384	24.232571	7.302766
15	6.617594	23.002221	5.794899
15	8.9865	22.327802	9.477625
8	8.209878	25.591675	6.614005
7	3.836548	22.841482	6.307094
7	4.437838	24.861315	5.885767
6	2.682268	23.616249	6.352611
6	4.915367	23.605019	6.022693
6	10.655836	25.244253	10.93911
6	3.062869	24.892451	6.097924
1	10.220076	25.143285	11.922949
6	11.021716	25.874044	8.777678
1	10.908515	26.333541	7.806841
6	12.092675	24.996851	9.165103
6	11.86664	24.606488	10.49498
1	12.472886	23.929515	11.079391
6	10.179382	26.082469	9.898976
1	9.295711	26.702898	9.928022
1	12.904159	24.674696	8.528779
6	9.054646	25.520049	5.470534
1	8.566847	24.914661	4.698012
1	9.99283	25.02315	5.74445
6	7.293817	22.255038	10.121517
1	6.801088	23.212921	9.963811
6	9.322767	26.926293	4.97442
1	9.976075	26.896116	4.098397
1	9.811451	27.521537	5.750347
1	8.388731	27.420215	4.694195
1	6.737139	21.465175	9.613427
6	9.685709	20.685919	9.802996
1	9.016252	19.905602	9.436188
1	7.339957	22.037021	11.191103
1	10.650461	20.610597	9.3004
1	9.832402	20.562587	10.878374
6	5.2272	26.021331	5.501681
1	5.53451	26.589143	6.37967
1	6.114504	25.671191	4.979428
1	4.631011	26.652592	4.844236
6	2.272299	26.153062	6.025846
1	2.290675	26.578976	5.019178
1	1.234633	25.954239	6.291759
1	2.666119	26.903453	6.71628
6	1.344441	23.030832	6.645336
1	1.099265	22.237771	5.934197
1	1.309301	22.604219	7.651387
1	0.578035	23.801909	6.57448
6	3.857368	21.405378	6.533919
1	4.891998	21.101637	6.677051
1	3.275037	21.169765	7.424475
1	3.437974	20.880513	5.67403

D6 (Ni, CPCM = CH2Cl2)

28	10.235887	24.021246	9.302534

15	9.297252	23.084827	7.481086
15	7.54168	24.472494	7.194204
15	6.654071	23.188875	5.655811
15	8.825907	22.482961	9.465515
8	8.293426	25.806996	6.482634
7	3.910981	22.759943	6.217898
7	4.336668	24.877532	5.986058
6	2.699553	23.430528	6.379826
6	4.932231	23.643276	5.976638
6	10.838228	25.195223	10.957147
6	2.970501	24.769076	6.247138
1	10.395723	25.142725	11.948671
6	11.260407	25.779246	8.774699
1	11.196202	26.256616	7.800547
6	12.232268	24.779738	9.157816
6	11.968497	24.415755	10.501132
1	12.508852	23.6791	11.089399
6	10.436952	26.072578	9.90516
1	9.62666	26.794988	9.939527
1	13.010791	24.37378	8.517896
6	9.174589	25.645873	5.354236
1	8.655694	25.054533	4.578889
1	10.064751	25.075342	5.674314
6	7.112357	22.660211	10.082673
1	6.757312	23.674837	9.868913
6	9.564708	27.016858	4.838375
1	10.245973	26.915365	3.98083
1	10.076942	27.597318	5.619066
1	8.677701	27.577845	4.510852
1	6.456866	21.927815	9.591232
6	9.257368	20.737698	9.810194
1	8.477746	20.066655	9.423968
1	7.111402	22.486186	11.168052
1	10.217013	20.509299	9.330974
1	9.354266	20.598029	10.896224
6	5.019103	26.128817	5.692495
1	5.333728	26.63394	6.614469
1	5.910733	25.899578	5.09743
1	4.345271	26.782667	5.127023
6	2.074853	25.952112	6.342487
1	2.016766	26.5	5.389249
1	1.060649	25.632081	6.608787
1	2.422405	26.658808	7.111276
6	1.42206	22.719128	6.649651
1	1.184307	22.001474	5.849757
1	1.460509	22.159185	7.59679
1	0.598625	23.439671	6.715613
6	4.07176	21.317289	6.309681
1	5.13822	21.111049	6.464892
1	3.487606	20.935038	7.155085
1	3.738904	20.827478	5.384526

MeOTf (CPCM = CH_2Cl_2)

16	6.574445	21.99457	-3.603062
8	7.465393	22.94768	-4.224937
8	6.917301	21.778906	-2.068471
8	6.344311	20.681247	-4.151041
6	4.883334	22.825181	-3.497312
9	4.998259	24.029891	-2.911117
9	4.404755	22.981309	-4.741256
9	4.03977	22.064226	-2.785793
6	7.553025	22.866716	-1.302829
1	8.006191	22.360413	-0.446374
1	8.315469	23.355661	-1.916778
1	6.782996	23.573867	-0.977791

$[OTf]^-$ (CPCM = CH_2Cl_2)

16	11.550144	23.303059	2.568235
8	11.445537	24.206822	1.414343
8	12.386281	23.794671	3.672152
8	11.727718	21.88268	2.236061
6	9.820924	23.349799	3.299022
9	9.470424	24.610519	3.660475
9	8.89592	22.90653	2.409668
9	9.733343	22.567249	4.405095

D5 (Ni)

28	12.402679	5.248204	4.973644
15	11.109052	6.826604	4.112218
15	10.398884	4.383536	5.3208
15	10.550762	2.25457	4.828496
15	10.176226	5.056785	3.280321
7	11.778405	7.584303	1.385772
7	13.083276	8.394939	2.888455
8	8.578822	5.385859	2.946867
6	12.018973	7.590355	2.709315
6	14.123468	4.165822	4.223382
1	14.118121	3.626868	3.287571
6	13.692711	3.669975	5.480172
1	13.356341	2.663089	5.675415
6	13.882981	4.693459	6.472483
1	13.672409	4.594679	7.527482
6	12.724088	8.36335	0.718515
6	13.54288	8.877407	1.668283
6	14.430002	5.531891	4.407436
1	14.75408	6.213923	3.634662
6	14.327526	5.842973	5.811769

1	14.537053	6.801245	6.263634
6	13.631178	8.812534	4.171205
1	13.127696	8.270645	4.965806
6	10.65971	6.935782	0.709377
1	9.794607	6.923185	1.366842
6	12.734152	8.521248	-0.762578
1	12.771922	7.549788	-1.261411
1	13.610644	9.094269	-1.062891
1	11.844984	9.050267	-1.115251
6	14.717629	9.786857	1.572335
1	14.53759	10.717831	2.116342
1	14.913435	10.032812	0.529529
1	15.612981	9.319221	1.990149
1	13.471953	9.883777	4.300268
1	14.698103	8.594639	4.206777
6	7.726052	6.025131	3.897096
1	8.188639	6.962971	4.22401
1	7.624058	5.381237	4.776709
1	10.416616	7.513733	-0.179767
1	10.918164	5.916495	0.425869
6	6.380309	6.280044	3.250077
1	6.486702	6.935201	2.381664
1	5.707738	6.760055	3.965977
1	5.925402	5.34123	2.924476
6	8.768318	1.92588	4.483297
1	8.489857	2.395152	3.537376
1	8.612928	0.84739	4.392252
1	8.125377	2.316148	5.276917
6	10.637674	1.570168	6.540995
1	9.844801	1.976712	7.174253
1	10.536839	0.482539	6.49732
1	11.604284	1.806676	6.99043

Int^{Me}1' (CPCM = CH_2Cl_2)

28	10.890565	24.609243	9.366277
15	9.483149	23.806181	7.96654
15	7.698043	24.992708	7.523481
15	7.023689	23.82939	5.865292
15	9.461651	23.182352	9.981864
8	8.440149	26.389407	7.050871
7	4.263777	23.337339	6.235556
7	4.663399	25.442527	6.057308
6	3.034451	23.981648	6.310368
6	5.261142	24.233613	6.086274
6	11.629163	26.045811	10.717572
6	3.287205	25.311195	6.20821
1	11.12884	26.344375	11.627005
6	12.292991	25.876278	8.536005
1	12.383647	26.071157	7.47706
6	12.999626	24.872423	9.260784
6	12.543314	24.929648	10.593023
1	12.840816	24.275753	11.399751
6	11.502026	26.645191	9.457786
1	10.874674	27.485748	9.201993
1	13.692357	24.155464	8.846641
6	9.322921	26.423514	5.926015
1	8.7676	26.133785	5.028793
1	10.123309	25.687505	6.077782
6	7.962086	23.577505	10.905632
1	7.661588	24.598817	10.676197
6	9.898859	27.817051	5.8011
1	10.5591	27.868645	4.931844
1	10.475815	28.077649	6.691506
1	9.09972	28.55124	5.674479
1	7.163763	22.881631	10.644075
6	9.831366	21.431973	10.230127
1	8.952935	20.825063	10.005823
1	8.189273	23.495549	11.970796
1	10.664512	21.143166	9.589871
1	10.119143	21.289393	11.273997
6	5.341304	26.713823	5.846043
1	5.549781	27.202627	6.798113
1	6.277641	26.522358	5.326812
1	4.707365	27.354751	5.235919
6	2.372421	26.485947	6.23042
1	2.3801	27.012918	5.27276
1	1.35318	26.157246	6.429798
1	2.66423	27.194781	7.009367
6	1.757693	23.234655	6.48068
1	1.611928	22.516715	5.669456
1	1.745189	22.684515	7.425211
1	0.91824	23.928777	6.478337
6	4.421255	21.891397	6.295385
1	5.47935	21.666419	6.406303
1	3.869244	21.502467	7.150641
1	4.043289	21.436318	5.37893
6	9.963856	22.491752	6.808362
1	10.841939	21.984722	7.209591
1	9.14498	21.789042	6.66306
1	10.221467	22.956457	5.855168

Int^{Me}1 (CPCM = CH_2Cl_2)

28	9.837494	27.616072	8.480658
15	9.989428	25.883669	7.09654
15	8.056857	24.824967	7.345799
15	8.510315	22.77132	7.589465

15	10.803558	25.859743	9.076211
8	7.208949	25.25805	6.00292
7	6.30135	22.115657	9.280442
7	5.77157	22.002877	7.180108
6	4.968473	21.728209	9.228553
6	6.804621	22.29498	8.023626
6	9.617603	29.194766	9.834465
6	4.631106	21.660207	7.895196
1	9.915335	29.157323	10.879096
6	8.382416	29.026275	7.90701
1	7.577108	28.826788	7.205072
6	9.694747	29.533779	7.55361
6	10.451895	29.646096	8.740719
1	11.47977	29.986842	8.824245
6	8.321288	28.872703	9.32068
1	7.469007	28.52721	9.897407
1	10.031731	29.763879	6.546901
6	7.775849	24.987935	4.687852
1	8.101273	23.936043	4.650853
1	8.662601	25.626506	4.552927
6	10.247934	24.692539	10.363141
1	9.168233	24.775772	10.516791
6	6.717289	25.289464	3.650051
1	7.125125	25.099111	2.647205
1	6.406092	26.341785	3.703131
1	5.833833	24.6522	3.790883
1	10.505856	23.664736	10.076486
6	12.61935	25.665537	9.033154
1	12.886084	24.603934	8.941499
1	10.767621	24.96238	11.294113
1	13.019201	26.229571	8.182329
1	13.037809	26.070088	9.965481
6	5.842602	22.033062	5.725328
1	5.239718	22.862397	5.335994
1	6.891281	22.174028	5.443988
1	5.471433	21.084713	5.319137
6	3.349812	21.305964	7.231761
1	3.457817	20.410371	6.601592
1	2.579798	21.101823	7.984141
1	2.994526	22.12502	6.588801
6	4.167753	21.463276	10.451559
1	4.59513	20.635216	11.036902
1	4.129978	22.348077	11.104494
1	3.141299	21.194337	10.178414
6	7.041535	22.324343	10.51844
1	8.065657	22.603054	10.250609
1	6.57447	23.129946	11.098698

1	7.046332	21.400685	11.109765
6	6.902532	25.522376	8.559116
1	5.976951	24.930749	8.54879
1	7.351383	25.494259	9.556919
1	6.689017	26.560833	8.281393

D4 (Ni)

28	16.015005	18.981575	-3.284247
15	15.237543	16.659771	-5.144703
15	16.233686	16.789564	-3.218332
15	13.959748	18.550124	-3.065442
15	14.054368	16.402205	-3.31365
7	16.448438	14.09714	-5.550499
7	14.318046	14.055156	-5.847184
6	15.341342	14.851934	-5.504739
6	14.773099	12.764822	-6.076597
6	16.120572	12.790888	-5.888203
6	16.798276	20.468639	-4.620553
6	17.834728	19.67163	-4.104533
1	18.514527	19.055036	-4.674945
6	17.866633	19.837014	-2.668101
6	16.119923	21.045349	-3.503488
1	15.256425	21.693185	-3.55764
6	16.838531	20.717186	-2.306164
1	18.568567	19.358625	-2.000685
1	16.584063	21.035532	-1.306104
1	16.510777	20.562913	-5.657183
6	17.816375	14.555363	-5.32305
1	18.131181	14.306529	-4.309999
1	18.468463	14.068758	-6.045818
1	17.863549	15.631106	-5.466761
6	17.144245	11.715454	-5.990757
1	17.7216	11.636697	-5.066552
1	16.655899	10.759215	-6.172828
1	17.839482	11.908118	-6.811937
6	13.856708	11.655375	-6.456368
1	13.400254	11.838219	-7.432592
1	14.411914	10.719942	-6.508635
1	13.055619	11.540787	-5.722308
6	12.920431	14.457948	-5.959882
1	12.499075	14.015142	-6.860743
1	12.364424	14.121029	-5.084671
1	12.86384	15.540676	-6.0348
6	12.72431	19.26148	-4.185731
1	13.025867	19.073937	-5.216832
1	11.744534	18.817619	-3.998839
1	12.678322	20.339857	-4.017814
6	13.276862	18.781732	-1.400934

1	12.262402	18.382049	-1.344042
1	13.917713	18.273633	-0.679292
1	13.264917	19.85008	-1.175616

D3 (Ni)

28	3.17534	5.065113	3.680202
15	2.772138	2.925895	3.170006
15	1.988265	3.72249	4.974667
15	0.936938	5.057224	3.722545
15	0.366962	6.436012	5.338777
7	5.346598	1.910269	2.855846
7	4.622988	1.380922	4.811833
6	4.300037	2.041268	3.688555
6	6.359651	1.186077	3.465535
6	5.904044	0.851261	4.702288
6	3.761366	6.970446	4.264661
1	3.184708	7.618805	4.906829
6	4.77041	6.07442	4.684595
1	5.076775	5.889918	5.702979
6	3.656804	6.903915	2.827217
1	2.985639	7.48995	2.217728
6	5.222131	5.380576	3.534333
1	5.973637	4.605153	3.523424
6	4.56315	5.927885	2.376338
1	4.721161	5.621112	1.353545
6	3.777582	1.203714	5.988946
1	2.740445	1.110048	5.678327
1	4.077258	0.288314	6.492729
1	3.890292	2.049858	6.666365
6	6.550949	0.079119	5.798981
1	6.015893	-0.852458	5.999397
1	7.5724	-0.170623	5.515502
1	6.582877	0.661244	6.722909
6	7.647091	0.897131	2.776729
1	8.303527	0.333231	3.437713
1	7.482638	0.307442	1.871445
1	8.154904	1.821984	2.491627
6	5.44499	2.439505	1.499164
1	6.259901	3.161485	1.447569
1	5.637225	1.619408	0.808298
1	4.511504	2.926091	1.228899
6	-1.355663	5.822563	5.55497
1	-1.891489	5.760222	4.604858
1	-1.332706	4.837065	6.02422
1	-1.885011	6.509364	6.220226
6	0.038326	7.928442	4.311298
1	-0.475641	8.663528	4.93571
1	0.97977	8.365469	3.974137
1	-0.584769	7.699779	3.443599

D4 (Mo)

15	15.33959	16.713129	-5.070623
15	16.285071	16.79506	-3.119271
15	13.997666	18.62787	-2.994808
15	14.104437	16.483032	-3.278705
7	16.515152	14.157314	-5.522499
7	14.376657	14.108373	-5.756304
6	15.406649	14.906915	-5.439172
6	14.831073	12.821071	-6.007025
6	16.183252	12.85206	-5.85878
6	17.885935	14.622337	-5.32762
1	18.226846	14.371791	-4.323507
1	18.522255	14.142211	-6.068599
1	17.924698	15.698954	-5.468834
6	17.208842	11.782972	-6.002727
1	17.824294	11.708026	-5.103299
1	16.719918	10.823527	-6.165535
1	17.868123	11.980714	-6.852015
6	13.908799	11.709762	-6.366554
1	13.425467	11.895029	-7.329283
1	14.465551	10.776486	-6.437558
1	13.128337	11.589394	-5.611515
6	12.975425	14.50701	-5.840455
1	12.536406	14.056461	-6.728884
1	12.439678	14.174697	-4.95108
1	12.914242	15.588785	-5.922146
6	12.824245	19.297687	-4.210573
1	13.236888	19.191599	-5.21409
1	11.876524	18.76077	-4.141416
1	12.658726	20.357414	-4.004158
6	13.055655	18.736594	-1.440375
1	12.040668	18.367281	-1.600571
1	13.544172	18.133515	-0.674545
1	13.020989	19.776881	-1.110847
42	16.335503	19.316483	-2.879341
8	15.270988	21.362344	-4.972177
8	18.128618	18.750841	-5.349252
6	15.644037	20.598084	-4.197671
6	17.448718	18.95257	-4.442355
6	16.403516	20.909999	-1.214488
1	15.751044	21.770124	-1.218222
6	17.684625	20.822407	-1.835816
1	18.180116	21.606364	-2.387731
6	16.157651	19.680575	-0.546977
1	15.284742	19.438903	0.037986
6	18.216785	19.531231	-1.553597

1	19.178222	19.155005	-1.869914
6	17.272764	18.834894	-0.754825
1	17.381277	17.828378	-0.378235

D7 (CPCM = CH_2Cl_2)

28	10.476777	11.427798	13.365179
15	11.993901	9.744813	11.069178
15	11.428758	8.038321	9.871563
15	11.272805	9.447288	13.135228
15	10.397526	11.27019	11.2511
7	13.583421	8.892031	8.158352
7	13.783496	6.840289	8.8407
8	8.934506	10.73678	10.7532
6	12.989989	7.948131	8.950834
6	14.746682	8.389595	7.578099
6	14.864293	7.0864	7.997636
6	11.192763	12.978417	14.644792
6	10.718387	11.860189	15.396399
6	10.105806	13.430154	13.84374
1	10.133342	14.248714	13.129772
6	12.652108	8.864998	14.182138
1	13.447052	9.620049	14.181884
6	9.301252	11.690142	15.138036
6	8.925795	12.649915	14.177726
6	10.03979	8.100058	13.215647
1	9.676772	8.020326	14.249473
1	13.038063	7.915196	13.785988
1	10.496358	7.150122	12.906949
6	10.7824	12.699557	10.183434
1	10.843899	12.39506	9.130249
1	11.748999	13.107448	10.504426
1	10.00084	13.461112	10.307509
1	12.20477	13.370839	14.642493
1	9.201639	8.342049	12.551331
1	7.941077	12.777607	13.737985
1	12.288689	8.714139	15.207584
1	8.664068	10.931721	15.582788
6	8.679163	10.479951	9.331293
1	9.62976	10.241053	8.832778
1	8.261921	11.400479	8.897121
6	7.717199	9.318681	9.230339
1	7.473157	9.133245	8.174285
1	6.784224	9.532855	9.769587
1	8.171613	8.40839	9.64592
6	13.542557	5.574235	9.518897
1	13.153364	4.827684	8.814328
1	14.47812	5.209163	9.958549
1	12.802209	5.752047	10.308247
6	15.888296	6.053234	7.692776
1	16.417564	5.728092	8.601412
1	15.434044	5.161136	7.235838
1	16.630556	6.4533	6.992649
6	15.615684	9.204974	6.689406
1	15.96252	10.116968	7.197843
1	16.496059	8.624446	6.391007
1	15.087842	9.513937	5.774186
6	13.052179	10.224537	7.910057
1	11.966819	10.193026	8.061574
1	13.489498	10.958587	8.599365
1	13.275151	10.513891	6.877391
1	11.307206	11.26029	16.08501

TS1 (Ni)

28	0.547974	-1.002177	-0.857685
15	0.401056	0.859288	-2.233546
15	-1.121957	0.477774	-0.793828
15	-0.687157	-1.898086	-4.764415
15	0.001675	-1.04051	-2.939643
7	-1.241721	1.142619	1.918686
6	2.263604	-2.22796	-0.694952
1	2.853722	-2.549256	-1.540233
7	0.075006	2.524929	0.928467
6	0.043415	2.894916	2.267959
6	1.079464	-2.902339	-0.199714
1	0.664601	-3.815054	-0.600191
6	0.633058	-3.11885	-5.137587
6	2.501329	-1.101946	0.10661
1	3.294545	-0.38033	-0.018651
6	-0.700949	1.447921	0.726391
6	-0.789359	2.01896	2.892618
6	-2.016139	-3.02663	-4.187606
6	1.424946	-1.010382	1.037154
1	1.301279	-0.236778	1.781288
6	0.5912	-2.170249	0.895922
1	-0.280693	-2.404791	1.486767
6	0.853783	3.215129	-0.09459
1	1.715216	2.611031	-0.378784
1	0.227452	3.405243	-0.964026
1	1.194927	4.164086	0.309077
6	-2.14019	0.02737	2.200785
1	-3.029748	0.402596	2.704985
1	-2.430189	-0.449977	1.268308
1	-1.632025	-0.695354	2.839484
6	-1.220298	1.911337	4.313292
1	-2.297277	2.070989	4.408888
1	-0.985938	0.923911	4.718629

1	-0.707974	2.660258	4.91511
6	0.807211	4.055253	2.805096
1	0.46602	4.993178	2.359687
1	0.662868	4.119414	3.88262
1	1.876462	3.949929	2.608032
1	0.824965	-3.785959	-4.295597
1	1.5487	-2.592651	-5.408654
1	0.298393	-3.711366	-5.993973
1	-2.347677	-3.614174	-5.048721
1	-1.667832	-3.703689	-3.40589
1	-2.855751	-2.437623	-3.817569

TS1 (Mo)

42	1.005921	-1.214476	-0.756189
15	0.339053	0.992376	-1.900861
15	-0.926735	0.365304	-0.309899
15	-1.473961	-1.126324	-4.521316
15	-0.264248	-0.789372	-2.813096
7	-0.536045	1.139647	2.401097
6	1.488878	-3.430785	-0.958604
1	2.039404	-3.814146	-1.804374
7	-0.181737	2.805314	1.093543
6	-0.019505	3.276903	2.392488
6	0.070481	-3.359783	-0.85482
1	-0.634079	-3.668489	-1.610763
6	-0.448494	-2.253295	-5.550016
6	2.048867	-2.942468	0.255406
1	3.097028	-2.913865	0.509837
6	-0.476289	1.493377	1.106549
6	-0.246921	2.223122	3.218274
6	-2.726872	-2.331662	-3.919971
6	0.962167	-2.539532	1.09347
1	1.054137	-2.152448	2.09558
8	3.349761	-0.86284	-2.836104
8	2.717224	0.711714	1.069476
6	2.498523	-0.96304	-2.091529
6	-0.253655	-2.812858	0.404939
1	-1.252866	-2.634223	0.769129
6	2.091185	0.033339	0.404871
6	-0.086024	3.66053	-0.086138
1	0.916822	3.616117	-0.509412
1	-0.815913	3.339294	-0.824784
1	-0.312322	4.681435	0.210956
6	-0.921877	-0.165106	2.919312
1	-1.198088	-0.809689	2.091289
1	-0.092319	-0.603152	3.473619
1	-1.780501	-0.04838	3.579924
6	-0.227403	2.110893	4.702349

1	-1.207697	1.818604	5.087504
1	0.501979	1.365247	5.028661
1	0.040352	3.070209	5.142627
6	0.329271	4.691431	2.698827
1	-0.473804	5.371592	2.403521
1	0.491289	4.802834	3.770111
1	1.241627	4.993602	2.179663
1	-0.120485	-3.131855	-4.991098
1	0.420949	-1.711488	-5.923452
1	-1.056277	-2.577971	-6.399892
1	-3.316785	-2.659629	-4.781229
1	-2.266701	-3.205126	-3.455038
1	-3.387315	-1.84088	-3.204436

D1 (Ni) (NHC = IDipp, PR_2 = PPh_2)

28	16.020785	18.849574	-3.400038
15	15.234895	16.414274	-4.948092
15	16.442195	16.702514	-3.179494
15	14.086606	18.268275	-2.793534
15	14.300791	16.127676	-2.990463
7	16.405426	13.784189	-5.313576
7	14.366963	13.956059	-5.977966
6	15.352446	14.614062	-5.3436
6	13.048599	14.487196	-6.235201
6	17.686554	14.057994	-4.707385
6	14.802413	12.699509	-6.354666
1	14.162973	12.015803	-6.886836
6	18.620392	14.798698	-5.431192
6	16.083508	12.590279	-5.934636
1	16.798411	11.788418	-6.013904
6	17.911832	13.568942	-3.418351
6	16.890937	19.953818	-5.023777
6	17.867859	19.5167	-4.081939
1	18.700656	18.861876	-4.29561
6	12.879327	15.327692	-7.33914
6	17.623342	20.168466	-2.825399
6	15.966935	20.747614	-4.318528
1	15.068975	21.193859	-4.721027
6	16.863174	12.781381	-2.651922
1	15.905842	12.886673	-3.168353
6	16.435698	20.898409	-2.959749
6	19.841418	15.054564	-4.810856
1	20.594823	15.631728	-5.334932
6	14.026899	15.70308	-8.259427
1	14.966943	15.484651	-7.750629
6	18.351064	15.322795	-6.830198
1	17.339183	15.035027	-7.12237
6	13.718955	18.459199	-1.031648

6	20.105478	14.575643	-3.536104
1	21.063827	14.782089	-3.071678
6	12.007674	14.114886	-5.378675
1	18.215447	20.055797	-1.929307
6	14.697007	18.946094	-0.168873
1	15.659811	19.245961	-0.567833
6	13.987107	14.847505	-9.530944
1	14.040045	13.782298	-9.291043
1	14.829831	15.096873	-10.180679
1	13.06112	15.027757	-10.084193
1	15.939545	21.468597	-2.187811
6	19.153137	13.835017	-2.849494
1	19.373287	13.471697	-1.852591
6	10.735929	14.594657	-5.683072
1	9.897723	14.324509	-5.052268
6	16.666631	13.316002	-1.230402
1	15.837194	12.789002	-0.751973
1	16.437908	14.383688	-1.240257
1	17.557863	13.159848	-0.618124
6	14.434082	19.045302	1.193604
1	15.196704	19.425704	1.864211
6	17.226005	11.292182	-2.629102
1	18.175038	11.140477	-2.107268
1	17.327399	10.890003	-3.640157
1	16.453535	10.721894	-2.106829
6	12.222652	13.197918	-4.187856
1	13.285563	13.212644	-3.933164
6	10.528351	15.411462	-6.784732
1	9.528827	15.769562	-7.007428
6	11.588851	15.783228	-7.597299
1	11.410563	16.430278	-8.447856
6	13.195463	18.66019	1.690911
1	12.989338	18.740403	2.752801
6	12.475441	18.068914	-0.529256
1	11.713209	17.687336	-1.201832
6	18.429384	16.85219	-6.874039
1	19.444524	17.198422	-6.66302
1	17.758279	17.303413	-6.140614
1	18.146809	17.21342	-7.866233
6	12.215448	18.172031	0.829
1	11.249054	17.871352	1.218609
6	11.446911	13.657335	-2.950268
1	10.371922	13.507644	-3.076227
1	11.626359	14.712724	-2.734798
1	11.762542	13.071887	-2.083297
6	14.032849	17.196732	-8.592346
1	13.174477	17.473481	-9.208874
1	14.938002	17.445039	-9.151977
1	14.013593	17.800865	-7.68204
6	11.835253	11.757619	-4.5463
1	12.011825	11.096984	-3.693609
1	12.407887	11.381145	-5.396131
1	10.773854	11.707121	-4.805191
6	19.312137	14.69266	-7.843412
1	19.082893	15.049668	-8.85072
1	19.230739	13.602724	-7.835619
1	20.347417	14.960282	-7.616694
6	12.627631	18.893246	-3.661572
6	11.872029	18.101861	-4.521506
1	12.122202	17.056902	-4.671806
6	12.302199	20.240825	-3.486385
1	12.897076	20.859972	-2.821459
6	10.463053	19.994157	-5.017491
1	9.618702	20.422257	-5.546962
6	10.78859	18.656025	-5.196804
1	10.200812	18.03801	-5.864856
6	11.221471	20.788246	-4.160496
1	10.970209	21.833892	-4.020448
1	16.825402	19.656043	-6.059568

D4 (Mo) (NHC = IDipp, PR_2 = PPh_2)

15	15.032149	16.400001	-5.365286
15	16.410732	17.001441	-3.823997
15	13.934388	18.257942	-3.000039
15	14.414764	16.1719	-3.285289
7	16.324676	13.756428	-5.403292
7	14.354738	13.861332	-6.258288
6	15.279939	14.576924	-5.596297
6	13.046113	14.355647	-6.614459
6	17.54587	14.043151	-4.686342
6	14.817035	12.581312	-6.494133
1	14.224756	11.852963	-7.02173
6	18.606878	14.618149	-5.387835
6	16.05422	12.514362	-5.954303
1	16.771124	11.712222	-5.90367
6	17.604673	13.698971	-3.33457
6	12.919425	15.125376	-7.773155
6	16.425402	13.094088	-2.592863
1	15.519943	13.280874	-3.175091
6	19.788879	14.829334	-4.684653
1	20.641779	15.267709	-5.188053
6	14.101402	15.438643	-8.673451
1	15.022323	15.235669	-8.123017
6	18.494335	14.982414	-6.857034
1	17.435715	15.105888	-7.098791

6	13.888977	18.212392	-1.170684
6	19.890484	14.482298	-3.344471
1	20.822216	14.650054	-2.814739
6	11.976121	14.036023	-5.772043
6	15.046674	17.820958	-0.490101
1	15.949474	17.58783	-1.041695
6	14.086634	14.516602	-9.898368
1	14.106647	13.464756	-9.600925
1	14.956701	14.713984	-10.529839
1	13.184735	14.686796	-10.492849
6	18.810023	13.926894	-2.675964
1	18.903827	13.670068	-1.62755
6	10.717782	14.499686	-6.146547
1	9.857411	14.27004	-5.529382
6	16.219425	13.733369	-1.216768
1	15.277895	13.382367	-0.786978
1	16.179847	14.822376	-1.28605
1	17.021543	13.46092	-0.526833
6	15.052273	17.720006	0.892131
1	15.956236	17.407867	1.403752
6	16.593243	11.575596	-2.460648
1	17.488548	11.342804	-1.877163
1	16.690277	11.095243	-3.436938
1	15.729203	11.143276	-1.949299
6	12.152546	13.187681	-4.523946
1	13.209052	13.202738	-4.245441
6	10.552938	15.256158	-7.298239
1	9.564498	15.609421	-7.572139
6	11.640816	15.570701	-8.0993
1	11.494069	16.164919	-8.993346
6	13.902634	18.02081	1.617988
1	13.907512	17.949155	2.700117
6	12.737971	18.495781	-0.440402
1	11.823714	18.781432	-0.943932
6	19.186229	16.307452	-7.181845
1	20.272562	16.223133	-7.100744
1	18.849596	17.101667	-6.512414
1	18.952531	16.602208	-8.207707
6	12.749681	18.407247	0.949135
1	11.847577	18.63757	1.505441
6	11.36321	13.724065	-3.327526
1	10.286044	13.626454	-3.482145
1	11.59006	14.774264	-3.134922
1	11.622777	13.150213	-2.434508
6	14.140167	16.910626	-9.090362
1	13.30079	17.163962	-9.74212
1	15.062021	17.110791	-9.642036
1	14.113065	17.568224	-8.218518
6	11.754827	11.734151	-4.810063
1	11.914249	11.117699	-3.921671
1	12.335123	11.308936	-5.631349
1	10.696458	11.679425	-5.080234
6	19.046118	13.848349	-7.730048
1	18.935474	14.09715	-8.788662
1	18.524109	12.906995	-7.542152
1	20.108872	13.694466	-7.522044
6	12.232458	18.606333	-3.510823
6	11.498969	17.682218	-4.248287
1	11.921173	16.71314	-4.489337
6	11.691629	19.867269	-3.248737
1	12.273156	20.603149	-2.701157
6	9.682175	19.254104	-4.422193
1	8.689354	19.506458	-4.778578
6	10.224506	18.006387	-4.7016
1	9.660916	17.28202	-5.278332
6	10.417753	20.185698	-3.693681
1	10.001135	21.164261	-3.481902
42	15.79863	19.462352	-3.996938
8	13.517519	20.542042	-5.815691
8	16.742454	18.821714	-6.888855
6	14.332138	20.122994	-5.121276
6	16.385482	19.022513	-5.814888
6	15.995548	21.54352	-2.984412
1	15.205001	22.277334	-3.035403
6	17.042679	21.371037	-3.932117
1	17.201599	21.964374	-4.819615
6	16.177623	20.593665	-1.949778
1	15.542357	20.471163	-1.086276
6	17.870431	20.30131	-3.476016
1	18.766166	19.938451	-3.957191
6	17.330492	19.826166	-2.249374
1	17.736287	19.026547	-1.647936

D3 (Mo) (NHC = IDipp, PR_2 = PPh_2)

42	3.307182	5.44413	3.461783
15	3.155965	2.95371	3.325149
15	2.493718	3.777137	5.167232
15	0.990722	4.589866	3.920838
15	0.076422	5.910461	5.41468
7	5.67376	1.698199	2.967978
8	3.093326	7.475623	5.84862
8	5.988922	4.904631	5.011647
7	4.758274	1.142788	4.836804
6	5.912441	2.307534	1.679889
6	6.982094	3.20082	1.550727

6	4.653114	1.993818	3.796794	6	8.299068	4.996621	2.774525
6	6.423658	0.657895	3.489517	1	8.925184	5.294078	1.930307
1	7.278864	0.261634	2.970437	1	7.418106	5.639708	2.788567
6	5.85199	0.313269	4.663125	1	8.86677	5.182233	3.689151
1	6.106386	-0.439451	5.39032	6	2.095397	0.85869	8.056442
6	3.842874	1.036531	5.949807	1	1.40264	0.79036	8.888367
6	3.094736	6.703294	5.013942	6	9.210323	2.674264	2.568794
6	-0.432284	7.257291	4.286929	1	9.874947	2.87536	3.412757
6	5.089269	1.932723	0.611829	1	9.000776	1.60358	2.546456
6	6.363952	3.449378	-0.77475	1	9.739576	2.932158	1.647107
1	6.53815	3.905783	-1.743176	6	2.283337	-0.331223	4.447883
6	-1.127805	7.019972	3.098264	1	2.977189	0.007892	3.677914
1	-1.429332	6.00906	2.843173	6	6.502089	1.541535	8.145706
6	7.190473	3.764158	0.293923	1	6.696006	0.581776	7.660547
1	8.009173	4.458804	0.149494	1	7.451604	2.072819	8.250731
6	4.046182	0.834567	0.730041	1	6.109463	1.341941	9.14674
1	3.820079	0.675949	1.783944	6	5.295726	3.750006	8.007339
6	7.930407	3.512539	2.695962	1	4.975094	3.640177	9.045969
1	7.440272	3.240387	3.632245	1	6.23376	4.310185	8.004777
6	-1.439822	8.067631	2.244724	1	4.542293	4.336969	7.477505
1	-1.981129	7.873106	1.324913	6	2.246291	6.161081	1.490452
6	4.201269	1.635365	7.159728	1	1.212155	5.95513	1.265677
6	-1.478285	4.97595	5.667727	6	4.626242	-0.478179	0.188865
6	-1.058974	9.368481	2.569164	1	5.543821	-0.75105	0.716593
1	-1.303858	10.187529	1.901373	1	3.902668	-1.288196	0.311868
6	5.020246	5.074029	4.438791	6	4.533371	5.993352	1.608047
6	5.329192	2.539671	-0.618715	1	5.538528	5.635965	1.455856
1	4.711822	2.281347	-1.470669	6	3.302515	1.521792	8.217119
6	2.728971	1.178528	0.033583	1	3.544168	1.969212	9.17396
1	1.987345	0.407603	0.257032	6	-2.724135	5.603837	5.674474
1	2.336695	2.137289	0.379575	1	-2.79872	6.66211	5.449644
1	2.846339	1.220235	-1.051862	6	3.354911	5.370149	1.115177
6	2.641009	0.349431	5.756445	1	3.318098	4.447501	0.558228
6	2.729354	7.279136	2.22308	6	-2.560853	2.890573	6.251391
1	2.119577	8.069524	2.635244	1	-2.489581	1.831311	6.475399
6	5.507149	2.386861	7.342134	6	-3.87662	4.881263	5.962614
1	5.938575	2.569619	6.35619	1	-4.838561	5.383037	5.959839
6	-0.058503	8.561506	4.605319	6	-3.799714	3.523166	6.250197
1	0.481383	8.754328	5.526801	1	-4.70009	2.96091	6.473173
6	4.152894	7.188423	2.283963	6	0.870987	0.025019	3.976661
1	4.819357	7.906027	2.737531	1	0.111705	-0.392596	4.642286
6	-1.408693	3.612259	5.966962	1	0.732454	1.107445	3.92983
1	-0.448924	3.105082	5.97703	1	0.701863	-0.386439	2.97825
6	1.767472	0.281143	6.838129	6	2.454269	-1.848927	4.580031
1	0.822702	-0.238136	6.728178	1	2.220609	-2.338813	3.631112
6	-0.369986	9.614995	3.749157	1	3.479106	-2.106979	4.859728
1	-0.07586	10.62647	4.007972	1	1.780984	-2.245386	5.34515

1	4.858562	-0.383488	-0.875532

D3' (Ni)

28	-0.259039	0.842229	1.474982
15	-1.033608	-1.15085	0.733852
15	0.188148	-1.150184	2.455759
15	-1.812569	0.84418	2.96417
15	-1.387346	-0.981285	3.932279
7	1.234763	-2.00799	-0.880296
7	-0.312762	-0.971735	-1.957761
6	0.060024	-1.374731	-0.730365
6	0.642334	-1.333915	-2.894972
6	1.622954	-1.986432	-2.214792
6	0.031822	2.173653	-0.162834
6	1.223551	1.428753	0.085359
1	1.720767	0.780308	-0.62046
6	1.686443	1.724919	1.397726
6	-0.290468	2.861856	1.022641
1	-1.14562	3.50233	1.179834
6	0.740201	2.579148	1.998521
1	2.565853	1.313401	1.869256
1	0.783396	2.976187	3.000439
1	-0.539868	2.177534	-1.077423
6	2.022845	-2.61994	0.18485
1	1.363309	-3.158881	0.860142
1	2.724426	-3.318151	-0.263158
1	2.567056	-1.853379	0.735836
6	2.893741	-2.595774	-2.695228
1	2.906705	-3.674651	-2.522373
1	2.999422	-2.42186	-3.764958
1	3.75657	-2.156184	-2.189259
6	0.496067	-1.01306	-4.340888
1	0.48053	0.067758	-4.503235
1	1.331007	-1.432045	-4.90029
1	-0.430658	-1.430901	-4.741679
6	-1.548348	-0.273714	-2.298459
1	-2.073063	-0.002896	-1.385975
1	-1.308883	0.629421	-2.85879
1	-2.179385	-0.924359	-2.903657
6	-3.476805	1.039486	2.267391
1	-3.645932	0.283572	1.50212
1	-4.2088	0.917517	3.069772
1	-3.574567	2.03723	1.833517
6	-1.71164	2.215177	4.148863
1	-2.502344	2.072573	4.890141
1	-0.745909	2.191193	4.651699
1	-1.854321	3.174501	3.647706

TS1' (Ni)

28	0.796429	1.051078	1.700119
15	-0.67635	0.149965	0.351858
15	-0.042047	-0.974193	2.047957
15	-2.650751	1.079941	2.823721
15	-0.76798	0.516214	3.373185
7	1.318943	-1.342226	-1.226506
7	-0.612835	-1.086546	-2.134766
6	0.100807	-0.808068	-1.025265
6	0.154394	-1.797372	-3.041197
6	1.375785	-1.961395	-2.466178
6	1.33149	2.976492	1.109757
6	2.394183	2.078952	0.730056
1	2.727406	1.89558	-0.281149
6	2.910553	1.490428	1.898173
6	1.20896	2.939197	2.515555
1	0.503466	3.495551	3.114036
6	2.140577	1.966653	3.001707
1	3.699993	0.755185	1.950661
1	2.272679	1.682398	4.035345
1	0.75103	3.58647	0.433679
6	2.458965	-1.335827	-0.317415
1	2.691453	-2.359839	-0.025482
1	3.316726	-0.892596	-0.822691
1	2.215902	-0.751674	0.560861
6	2.60228	-2.650734	-2.951948
1	2.885126	-3.465165	-2.280374
1	2.423512	-3.068343	-3.941665
1	3.44441	-1.957598	-3.017419
6	-0.379001	-2.238725	-4.358749
1	-0.745976	-1.387325	-4.93697
1	0.406952	-2.729246	-4.93105
1	-1.204438	-2.943793	-4.231634
6	-2.004842	-0.727229	-2.393967
1	-2.429633	-0.269501	-1.505572
1	-2.049804	-0.024961	-3.22639
1	-2.564569	-1.628624	-2.641039
6	-3.779512	0.302981	1.635075
1	-3.851987	0.878227	0.712029
1	-3.417798	-0.699612	1.409419
1	-4.759962	0.235965	2.112132
6	-3.438345	2.317561	3.891617
1	-4.311788	1.889603	4.386396
1	-2.708516	2.616075	4.646076
1	-3.735884	3.196076	3.31801

D3 (Ni) (NHC = IDipp, PR_2 = PPh_2)

28	3.129297	5.019391	3.888178
15	3.13944	2.924363	3.160469

15	2.080678	3.366585	4.94399	1	-0.88288	3.029788	5.815552
15	0.932818	4.629877	3.701694	6	2.086507	0.069854	6.999583
15	0.012813	5.725262	5.376526	1	1.270398	-0.643543	6.989323
7	5.620683	1.685034	2.864503	6	-0.254012	9.764401	4.881847
7	4.824351	1.230195	4.811867	1	-0.16752	10.629803	5.529913
6	5.767066	2.215037	1.526719	6	8.078384	5.124243	2.264601
6	6.755696	3.178265	1.293051	1	8.726103	5.309846	1.404778
6	4.620767	1.986243	3.71844	1	7.19639	5.756846	2.165268
6	6.445048	0.726037	3.418921	1	8.624652	5.433837	3.158725
1	7.289398	0.326977	2.885077	6	2.291771	0.864208	8.116643
6	5.947196	0.442474	4.642526	1	1.634742	0.767407	8.974196
1	6.266382	-0.249273	5.404097	6	9.015382	2.812313	2.324039
6	3.949095	1.113458	5.953115	1	9.695539	3.135014	3.11637
6	-0.226965	7.360983	4.593022	1	8.83468	1.744333	2.445649
6	4.929722	1.70702	0.526721	1	9.51524	2.961416	1.362792
6	6.028266	3.212214	-1.013418	6	2.686616	-0.710174	4.675422
1	6.127401	3.611883	-2.017001	1	3.406398	-0.440331	3.900812
6	-0.475471	7.538604	3.230197	6	6.594997	2.391568	7.564499
1	-0.573591	6.677041	2.578228	1	6.914867	1.532464	6.972211
6	6.865329	3.669091	-0.00579	1	7.387164	3.14406	7.529648
1	7.615375	4.416381	-0.23382	1	6.478699	2.064486	8.601665
6	3.957075	0.566157	0.77405	6	4.909672	4.233233	7.853803
1	3.77338	0.487436	1.845618	1	4.884034	4.025469	8.92606
6	7.721492	3.638402	2.373328	1	5.660456	5.009693	7.688325
1	7.249698	3.471896	3.345598	1	3.93361	4.621831	7.555449
6	-0.601762	8.814011	2.69759	6	3.395015	6.84923	2.923177
1	-0.78886	8.939416	1.636481	1	2.735984	7.230749	2.158315
6	4.173795	1.945482	7.053525	6	4.588556	-0.756672	0.32262
6	-1.648844	4.96091	5.235175	1	5.540448	-0.935075	0.82985
6	-0.490511	9.929763	3.52315	1	3.917198	-1.58976	0.546197
1	-0.588302	10.925931	3.105114	6	5.071498	5.730544	4.036979
6	5.076372	2.238341	-0.752071	1	5.938888	5.117989	4.226583
1	4.447212	1.876675	-1.556292	6	3.325057	1.791541	8.144373
6	2.599673	0.782438	0.10334	1	3.456422	2.414118	9.02022
1	1.910032	-0.00583	0.415482	6	-2.796567	5.676277	4.897223
1	2.170896	1.745774	0.386947	1	-2.731205	6.733576	4.669113
1	2.676906	0.741554	-0.985638	6	4.522935	6.036561	2.733338
6	2.920596	0.17313	5.888079	1	4.89569	5.668766	1.789384
6	3.230246	7.037763	4.342694	6	-2.996051	2.963614	5.473689
1	2.445769	7.613908	4.807643	1	-3.067331	1.905111	5.700372
6	5.279028	2.986603	7.048929	6	-4.033175	5.040186	4.847625
1	5.430328	3.302477	6.012457	1	-4.918033	5.609096	4.582347
6	-0.119303	8.48496	5.412319	6	-4.136335	3.683758	5.129702
1	0.080077	8.357342	6.471869	1	-5.100453	3.188919	5.085301
6	4.308026	6.405552	5.012692	6	1.284548	-0.503814	4.092746
1	4.465249	6.382328	6.078519	1	0.514347	-0.807059	4.806216
6	-1.763792	3.5993	5.534195	1	1.113734	0.542267	3.829012

1	1.165459	-1.107913	3.189729
6	2.928447	-2.182119	5.025166
1	2.795323	-2.804916	4.136912
1	3.941738	-2.333557	5.406446
1	2.222355	-2.522053	5.78718
1	4.773027	-0.743348	-0.755168

TS2 (Ni)

28	-0.173948	1.268476	1.76957
15	-0.985936	-0.151692	0.050497
15	-0.369783	-0.960246	1.887338
15	-2.130178	1.083298	2.596389
15	-2.609752	-0.864999	1.965487
7	1.300702	-1.575799	-1.219014
7	-0.436613	-1.360639	-2.465174
6	0.059784	-1.061681	-1.25125
6	0.485134	-2.081624	-3.201501
6	1.589301	-2.214439	-2.412524
6	0.381395	3.122301	0.899315
6	1.423616	2.209407	0.640383
1	1.773766	1.895615	-0.332378
6	1.91178	1.736946	1.900951
6	0.23641	3.23053	2.322617
1	-0.472458	3.865689	2.833869
6	1.219178	2.408412	2.937866
1	2.707261	1.018831	2.039553
1	1.372411	2.281984	3.998978
1	-0.216673	3.640128	0.164451
6	2.245121	-1.475552	-0.112418
1	2.234189	-2.39392	0.474475
1	3.241336	-1.305612	-0.516767
1	1.972945	-0.632795	0.514348
6	2.894637	-2.882189	-2.668206
1	3.113057	-3.623079	-1.895677
1	2.867189	-3.38782	-3.632334
1	3.711829	-2.156479	-2.684816
6	0.20291	-2.560558	-4.582211
1	0.024024	-1.721167	-5.258764
1	1.051971	-3.128773	-4.958895
1	-0.679175	-3.205474	-4.59943
6	-1.769506	-1.024872	-2.96097
1	-1.674961	-0.511639	-3.917011
1	-2.350691	-1.938194	-3.087634
1	-2.265838	-0.37334	-2.246736
6	-3.377392	2.319177	2.15358
1	-3.486194	2.339884	1.068836
1	-4.332986	2.06	2.614652
1	-3.053308	3.300488	2.505545
6	-1.940811	1.187259	4.398931
1	-2.910547	1.046218	4.879412
1	-1.249444	0.411129	4.727667
1	-1.533755	2.166815	4.658998

D3' (Mo)

15	-0.944659	-0.931153	0.573159
15	0.426363	-1.001454	2.199587
15	-1.769552	0.721063	3.114002
15	-0.906268	-1.049283	3.898994
7	1.099107	-2.318132	-0.934188
7	-0.015261	-0.901101	-2.103944
6	0.122295	-1.396271	-0.861264
6	0.911531	-1.474114	-2.962781
6	1.619268	-2.36686	-2.223807
6	1.533833	-3.229078	0.122345
1	0.711182	-3.40056	0.809676
1	1.81469	-4.172316	-0.340947
1	2.382832	-2.812065	0.661737
6	2.737531	-3.273779	-2.603567
1	2.435153	-4.322588	-2.547039
1	3.046242	-3.064495	-3.626862
1	3.598797	-3.130889	-1.946863
6	1.001119	-1.088593	-4.397394
1	1.174299	-0.014626	-4.502614
1	1.825687	-1.618434	-4.872009
1	0.08043	-1.33795	-4.930961
6	-1.07093	-0.006522	-2.557191
1	-1.741252	0.193586	-1.72808
1	-0.64563	0.92212	-2.9342
1	-1.631523	-0.496265	-3.353018
6	-3.506453	0.457767	2.62468
1	-3.544836	-0.225789	1.777439
1	-4.026119	0.003762	3.472293
1	-4.00045	1.395654	2.36411
6	-1.943083	1.955719	4.442105
1	-2.559278	1.520514	5.23287
1	-0.963644	2.199444	4.852992
1	-2.424642	2.865638	4.078283
42	-0.121969	1.349544	1.359138
8	2.594197	0.520482	-0.077513
8	1.75913	1.833581	3.814791
6	1.619411	0.799424	0.42946
6	1.067489	1.609304	2.939787
6	-0.269953	2.674923	-0.494018
1	0.167892	2.396583	-1.439387
6	0.400552	3.394131	0.548593
1	1.418765	3.751056	0.523774

6	-1.609268	2.449415	-0.084642
1	-2.386411	1.95575	-0.645099
6	-0.53137	3.591449	1.601142
1	-0.34579	4.106828	2.531412
6	-1.765336	3.011006	1.203639
1	-2.683845	3.039261	1.766009

TS1' (Mo)

15	-0.783433	-0.154261	-0.566137
15	-0.288276	-1.947919	0.474502
15	-3.143553	-0.621094	1.367432
15	-2.087793	-2.486632	1.464564
7	1.097004	-1.119121	-2.453887
7	1.729675	0.781163	-1.678006
6	0.779254	-0.159586	-1.564568
6	2.683395	0.399305	-2.612724
6	2.283485	-0.802884	-3.102266
6	0.314938	-2.314454	-2.758001
1	-0.728906	-2.135233	-2.514667
1	0.402774	-2.519977	-3.822809
1	0.684645	-3.164843	-2.185418
6	2.897963	-1.694449	-4.123719
1	2.27442	-1.758825	-5.019066
1	3.87222	-1.304331	-4.415126
1	3.035343	-2.704075	-3.729875
6	3.8708	1.240966	-2.924593
1	4.443038	1.45995	-2.019721
1	4.52054	0.716122	-3.623329
1	3.575185	2.189957	-3.379324
6	1.7626	2.063776	-0.992588
1	0.793882	2.24069	-0.53963
1	2.545135	2.061534	-0.233755
1	1.962667	2.849007	-1.719978
6	-4.543523	-0.914454	0.218161
1	-4.164626	-0.963834	-0.803253
1	-5.027501	-1.863843	0.469935
1	-5.270189	-0.101091	0.291966
6	-4.038452	-0.583551	2.968508
1	-4.672509	-1.46701	3.074017
1	-3.331017	-0.555727	3.797049
1	-4.668234	0.309039	3.00801
42	-0.412825	0.086112	1.899653
8	2.606293	-0.571022	1.610578
8	-0.156947	-2.000024	4.27617
6	1.481919	-0.370968	1.697813
6	-0.326012	-1.327029	3.377592
6	0.451797	2.168191	2.131199
1	1.388963	2.452318	1.683766
6	0.303566	1.588787	3.423597
1	1.102206	1.338912	4.10523
6	-0.852207	2.39829	1.596701
1	-1.085426	2.844058	0.642034
6	-1.091663	1.431174	3.66323
1	-1.540457	1.02662	4.55796
6	-1.789811	1.963943	2.548046
1	-2.86034	1.996326	2.434728

TS2 (Mo)

15	-0.600795	-0.523723	0.182223
15	0.141045	-1.015606	2.0812
15	-2.319431	0.563617	2.452332
15	-2.009224	-1.460964	2.193874
7	1.464806	-2.178453	-1.069219
7	-0.026675	-1.340435	-2.380752
6	0.373402	-1.392197	-1.094987
6	0.816806	-2.093431	-3.178009
6	1.753975	-2.630524	-2.349651
6	2.256987	-2.537519	0.104085
1	1.747747	-3.308226	0.680263
1	3.220522	-2.906409	-0.236015
1	2.414077	-1.66139	0.724112
6	2.894913	-3.538813	-2.650537
1	2.814285	-4.471191	-2.086932
1	2.897116	-3.78069	-3.712247
1	3.851782	-3.070851	-2.406853
6	0.627128	-2.213604	-4.649757
1	0.629535	-1.230048	-5.125681
1	1.434389	-2.803802	-5.080242
1	-0.320207	-2.704714	-4.885901
6	-1.143415	-0.557007	-2.90082
1	-1.855599	-0.368359	-2.100906
1	-0.777192	0.390083	-3.298608
1	-1.633524	-1.123887	-3.689505
6	-4.094881	0.989025	2.345995
1	-4.487649	0.639048	1.3905
1	-4.654668	0.535412	3.165434
1	-4.212127	2.073832	2.398107
6	-1.865611	1.041405	4.155562
1	-2.632416	0.699762	4.852132
1	-0.90676	0.603987	4.425209
1	-1.787539	2.129534	4.204461
42	0.306852	1.442269	1.28907
8	2.849169	0.76928	-0.296569
8	2.033128	1.152251	3.884728
6	1.890315	0.935459	0.332197
6	1.379533	1.229479	2.947369

6	0.268701	3.244297	-0.151379
1	0.721151	3.162632	-1.128069
6	0.930382	3.62963	1.052713
1	1.968547	3.909917	1.144434
6	-1.090127	3.00185	0.164373
1	-1.852997	2.678717	-0.528737
6	-0.040413	3.639985	2.0949
1	0.142396	3.908463	3.125184
6	-1.287283	3.277122	1.544821
1	-2.230154	3.26479	2.066097

TS^Me^1 (CPCM = CH_2Cl_2)

28	3.443716	0.259876	0.905627
15	3.019411	-0.441129	-1.19474
15	0.908461	0.302841	-1.240343
15	0.015672	-1.025978	-2.707619
15	2.850034	-1.70761	0.509739
8	1.147741	1.857453	-1.83367
7	-2.395965	-1.727564	-1.340126
7	-2.421046	0.334316	-2.014139
6	-3.5958	-1.179264	-0.895476
6	-1.652517	-0.799264	-2.020565
6	4.102327	0.879549	2.810312
6	-3.605321	0.130661	-1.309637
1	4.291108	0.216007	3.650291
6	3.016049	2.303027	1.350931
1	2.255684	2.891303	0.845455
6	4.38694	2.141998	0.911185
6	5.067513	1.307198	1.845739
1	6.112991	1.015305	1.808318
6	2.843306	1.532469	2.523857
1	1.929452	1.428773	3.101462
1	4.824802	2.610738	0.033943
6	1.780252	2.06489	-3.116831
1	1.316332	1.384632	-3.852753
1	2.848335	1.799741	-3.03464
6	1.249401	-2.487556	0.920257
1	0.457533	-1.735225	0.964261
6	1.603032	3.516243	-3.515147
1	2.082853	3.698984	-4.487762
1	2.060955	4.185605	-2.773115
1	0.536649	3.767993	-3.603402
1	0.999711	-3.239682	0.160328
6	4.023551	-3.110164	0.44373
1	3.62824	-3.910345	-0.196923
1	1.342028	-2.970802	1.903065
1	4.983219	-2.754696	0.0502
1	4.172322	-3.497822	1.461564
6	-2.073082	1.584762	-2.674467
1	-1.755009	2.336033	-1.940948
1	-1.243666	1.381883	-3.359974
1	-2.94428	1.956405	-3.226961
6	-4.60601	1.20864	-1.097356
1	-5.022828	1.572835	-2.048577
1	-5.433436	0.834742	-0.483634
1	-4.158141	2.069323	-0.577966
6	-4.592608	-1.965272	-0.12388
1	-4.944576	-2.836444	-0.696866
1	-4.162	-2.331453	0.819243
1	-5.460393	-1.340624	0.116836
6	-1.975902	-3.09291	-1.062955
1	-0.948027	-3.201846	-1.427128
1	-2.010781	-3.275272	0.017849
1	-2.631354	-3.804778	-1.580721
16	-2.302999	-0.338917	2.915325
8	-1.830586	0.917171	2.169641
8	-2.088333	-1.56639	2.165032
8	-3.593386	-0.094131	3.531855
6	-1.07833	-0.431124	4.338821
9	0.175188	-0.594672	3.866005
9	-1.118247	0.693552	5.076424
9	-1.382341	-1.480669	5.126723
6	-0.694528	0.698964	0.873071
1	-1.093254	-0.209221	0.439335
1	-0.902993	1.634961	0.36356
1	0.242927	0.631957	1.41834

D5' (Mo)

15	-2.117062	12.510502	19.662637
15	0.62668	12.618641	20.163261
15	-3.97188	13.582593	19.308696
15	-1.012157	13.540153	21.208205
8	-1.212932	12.69554	22.628658
7	-6.051287	11.730844	18.819177
7	-5.181367	11.435057	20.763742
6	-5.078533	12.18687	19.643736
6	0.696475	10.853948	20.608671
1	0.839489	10.745604	21.685452
1	-0.231707	10.369231	20.308915
6	2.207505	13.24163	20.812364
1	3.032208	12.838612	20.220025
6	-6.762441	10.695768	19.419439
1	1.534288	10.390424	20.082741
6	-6.205206	10.500724	20.63954
6	-1.165777	13.456652	23.844858
1	-0.529831	14.337951	23.703931

1	-0.690762	12.816375	24.592613
1	2.226707	14.329635	20.756398
6	-2.555769	13.868687	24.288977
1	-3.182395	12.98931	24.456821
1	-3.03125	14.494936	23.528613
1	-2.498131	14.437718	25.220977
1	2.323905	12.928096	21.851868
6	-6.319505	12.230082	17.480819
1	-5.457096	12.812987	17.162623
1	-6.472707	11.389501	16.804205
1	-7.207076	12.865097	17.480154
6	-4.408713	11.643025	21.97612
1	-5.049495	11.470091	22.840096
1	-3.546073	10.978399	22.012713
1	-4.055552	12.671355	21.977329
6	-7.898629	10.012501	18.740395
1	-7.571458	9.503732	17.829582
1	-8.337794	9.270647	19.406558
1	-8.677283	10.728677	18.46479
6	-6.52741	9.525991	21.718892
1	-7.30903	8.844682	21.384157
1	-5.647797	8.934629	21.985931
1	-6.880323	10.031285	22.621838
42	-0.295395	13.189294	18.009155
8	1.944729	15.332717	18.216076
6	1.11903	14.527649	18.156908
6	-1.287746	14.865486	18.18935
6	0.88964	12.014935	16.421365
1	1.968984	12.026817	16.398812
6	0.02563	12.938173	15.759396
1	0.335167	13.770305	15.145683
6	0.077925	11.053182	17.082148
1	0.428724	10.220528	17.672972
6	-1.315786	12.541035	16.017406
1	-2.208036	13.030075	15.656456
6	-1.277231	11.377036	16.8255
1	-2.13687	10.845221	17.203179
8	-1.832845	15.881148	18.239668

TS^{Me}1' (CPCM = CH_2Cl_2)

28	2.475555	1.260315	2.413466
15	1.731543	0.395891	0.50829
15	-0.29675	1.313445	0.170282
15	-0.591995	0.282289	-1.732322
15	1.390256	-0.536329	2.388359
8	0.125188	2.920442	-0.102106
7	-3.015529	-1.162573	-1.698815
7	-3.374475	0.947027	-1.31964
6	-4.393347	-1.011705	-1.556254
6	-2.374875	0.039874	-1.551943
6	3.864842	1.817496	3.925669
6	-4.619639	0.318272	-1.302721
1	4.335164	1.132742	4.626163
6	2.341644	3.315431	3.002887
1	1.47395	3.948874	2.845321
6	3.412512	3.102044	2.068715
6	4.379109	2.216996	2.666238
1	5.311557	1.891444	2.214313
6	2.599074	2.495535	4.132799
1	1.968862	2.397401	5.012638
1	3.502048	3.569607	1.09188
6	1.100109	3.262075	-1.108389
1	0.753623	2.877414	-2.083418
1	2.052165	2.755303	-0.866786
6	-0.319321	-0.753747	2.994457
1	-0.87688	0.176963	2.841319
6	1.276068	4.766608	-1.12874
1	2.01748	5.046243	-1.891339
1	1.628762	5.13159	-0.153692
1	0.327637	5.268051	-1.368566
1	-0.809375	-1.575638	2.454813
6	2.157392	-2.187787	2.550395
1	1.553633	-2.942769	2.028866
1	-0.277978	-0.990543	4.067024
1	3.168985	-2.158811	2.12915
1	2.22144	-2.444354	3.617277
6	-3.179903	2.382803	-1.179839
1	-3.150002	2.676387	-0.122666
1	-2.223983	2.64523	-1.647738
1	-3.999875	2.908236	-1.683034
6	-5.885991	1.054399	-1.047719
1	-6.086459	1.804833	-1.827978
1	-6.728464	0.35326	-1.029494
1	-5.857135	1.579883	-0.081222
6	-5.338121	-2.153742	-1.671615
1	-5.257702	-2.643884	-2.653684
1	-5.147293	-2.91839	-0.902923
1	-6.368922	-1.80114	-1.550372
6	-2.347629	-2.428572	-1.964997
1	-1.287462	-2.315171	-1.708419
1	-2.798581	-3.217178	-1.351649
1	-2.43373	-2.697315	-3.02645
16	2.182245	-4.033123	-2.358225
8	0.882114	-3.896339	-1.71766
8	2.994811	-2.741043	-2.370488

8	2.268504	-4.700912	-3.64576
6	3.18028	-5.111923	-1.187789
9	3.245464	-4.545633	0.037376
9	2.596193	-6.321515	-1.069664
9	4.435195	-5.281291	-1.648028
6	2.543118	-1.448316	-1.180027
1	3.012276	-0.645189	-1.7389
1	1.463886	-1.550967	-1.241708
1	3.068727	-1.838504	-0.314917

D8 (Ni)

28	0.643355	2.542974	1.301083
15	0.325166	0.329396	-0.835401
15	1.062846	0.399432	1.228569
15	-1.225071	1.809999	1.978046
15	-0.88852	-0.279436	1.983205
7	1.147807	-2.437531	-1.050221
7	-0.934284	-2.094291	-1.474714
6	0.213981	-1.478622	-1.152759
6	-0.731564	-3.459884	-1.577221
6	0.587797	-3.676393	-1.316305
6	1.106041	3.986398	-0.241349
6	2.291169	3.447605	0.323211
1	3.051858	2.896783	-0.211057
6	2.349412	3.816088	1.708895
6	0.373651	4.575785	0.81801
1	-0.595734	5.046868	0.73697
6	1.164947	4.502953	2.017312
1	3.146744	3.568343	2.39441
1	0.878627	4.891737	2.983974
1	0.78733	3.893283	-1.269398
6	2.563132	-2.235429	-0.754095
1	2.909279	-3.058814	-0.13285
1	3.133948	-2.203194	-1.682331
1	2.682031	-1.305525	-0.205468
6	1.382453	-4.934793	-1.290545
1	1.76885	-5.135344	-0.288184
1	0.753885	-5.772655	-1.588889
1	2.229772	-4.876851	-1.977894
6	-1.834467	-4.405351	-1.902493
1	-2.269081	-4.183761	-2.88033
1	-1.453171	-5.425332	-1.919789
1	-2.629523	-4.347091	-1.154772
6	-2.236556	-1.461732	-1.660349
1	-2.114472	-0.383025	-1.626632
1	-2.640677	-1.758968	-2.627385
1	-2.90746	-1.77526	-0.860971
6	-2.646975	2.285055	0.936508

1	-2.441425	1.986269	-0.092162
1	-3.551429	1.780023	1.285582
1	-2.793792	3.367195	0.975117
6	-1.720658	2.434111	3.618766
1	-2.648431	1.953647	3.939823
1	-0.930895	2.207703	4.335742
1	-1.86643	3.516147	3.572613
6	1.794582	0.591648	-1.85953
7	2.711951	0.859839	-2.504739

D9 (Ni)

28	7.430407	24.156193	-3.067337
15	7.499499	23.969044	-5.186372
15	8.495094	25.894903	-3.589969
15	8.548111	25.633832	-5.718129
1	5.191404	24.908345	-1.386379
6	5.937524	24.138824	-1.519622
6	5.819142	22.985393	-2.322027
1	4.957037	22.697868	-2.906257
6	7.255171	24.142768	-0.970364
6	7.915622	22.939225	-1.359472
1	7.252433	21.30633	-2.722046
6	7.045729	22.242599	-2.224185
1	7.666468	24.906514	-0.325579
1	8.917554	22.646128	-1.082473
6	7.684762	27.46766	-3.18372
1	6.681771	27.466212	-3.6115
1	7.610252	27.559819	-2.097913
1	8.257059	28.305161	-3.588457
6	10.181	26.04646	-2.935524
1	10.13582	26.1305	-1.847478
1	10.743481	25.150936	-3.201746
1	10.670432	26.927799	-3.355554

D10 (Ni)

28	9.629541	8.484499	36.425998
28	9.896295	6.39265	31.591156
15	11.590603	5.773487	32.694046
15	8.784313	6.546927	36.46832
15	11.347507	6.155086	34.823952
15	9.282938	5.410227	34.680384
15	10.525174	8.158956	34.442451
15	8.87635	7.142452	33.388043
6	9.596903	10.600469	36.540774
1	9.610609	11.253051	35.680048
6	8.911233	9.232336	38.230124
6	8.456428	10.115473	37.201469
6	10.767214	10.091894	37.22029
1	7.426888	10.301892	36.93402

6	10.3412	9.2914	38.290824
1	10.969409	8.755841	38.987167
6	9.23851	5.170689	29.94532
6	8.283801	6.158325	30.237708
1	7.26025	5.991864	30.540415
6	8.897067	7.453741	30.048802
6	10.468837	5.845852	29.666779
1	8.277202	8.676826	38.907037
6	10.226915	7.257291	29.646755
1	10.957894	8.023878	29.436299
1	11.789873	10.303129	36.943836
1	9.098042	4.101194	29.998148
1	11.4069	5.36647	29.424146
1	8.403411	8.403748	30.192463
6	12.005802	4.00694	32.556927
1	12.875973	3.766651	33.170792
1	11.150953	3.411925	32.88113
1	12.219973	3.776774	31.511192
6	13.147609	6.611032	32.271014
1	13.964632	6.240706	32.893485
1	13.372352	6.42241	31.219245
1	13.025719	7.684152	32.422342
6	9.273935	5.494592	37.869314
1	8.969195	5.980947	38.798377
1	8.799852	4.513922	37.79612
1	10.358637	5.378662	37.869969
6	6.967103	6.499747	36.517888
1	6.575283	7.030287	35.649433
1	6.610926	5.467565	36.512228
1	6.627161	7.000976	37.426316

$[CN]^-$

6	-0.11837	12.54667	20.779272
7	-0.11837	12.54667	21.947606

IMe_4-PCN

7	3.271456	11.591747	18.072462
6	3.595152	12.697709	18.772562
6	2.619197	10.674138	18.89001
6	2.526828	11.244744	20.116709
7	3.150675	12.487108	20.027843
6	3.357944	13.383899	21.154302
1	2.541701	14.101768	21.234739
1	3.421167	12.792689	22.065267
1	4.293271	13.92017	21.000833
6	1.917615	10.748431	21.381647
1	1.177342	11.456156	21.763127
1	1.41951	9.796352	21.202357
1	2.673417	10.598424	22.157129
6	2.166157	9.347529	18.387718
1	2.998304	8.793412	17.945777
1	1.762766	8.758131	19.210167
1	1.38802	9.452472	17.626967
6	3.560125	11.375465	16.662874
1	4.475043	10.792854	16.545118
1	2.727358	10.845028	16.204268
1	3.684187	12.346282	16.185698
15	4.56107	14.076266	18.10465
6	3.595736	15.356368	18.855079
7	3.005904	16.24724	19.305948

References

[1] omics.pnl.gov/software/molecular-weight-calculator **(2024)**.
[2] E. Mädl, G. Balázs, E. V. Peresypkina, M. Scheer, *Angew. Chem. Int. Ed.* **2016**, *55*, 7702–7707.
[3] O. J. Scherer, H. Sitzmann, G. Wolmershäuser, *J. Organomet. Chem.* **1984**, *268*, C9-C12.
[4] A. J. Arduengo, R. Krafczyk, R. Schmutzler, *Tetrahedron* **1999**, *55*, 14523–14534.
[5] N. Kuhn, T. Kratz, *Synth.* **1993**, 561–562.
[6] M. Gonsior, I. Krossing, N. Mitzel, *Z. anorg. allg. Chem.* **2002**, *628*, 1821.
[7] C. Riesinger, L. Dütsch, G. Balázs, M. Bodensteiner, M. Scheer, *Chem. Eur. J.* **2020**, *26*, 17165–17170.
[8] Agilent, *CrysAlisPro* **2014**, Agilent Technologies Ltd, Yarnton, Oxfordshire, England.
[9] G. M. Sheldrick, *Acta Cryst. A* **2015**, *71*, 3–8.
[10] G. M. Sheldrick, *Acta Cryst. C* **2015**, *71*, 3–8.
[11] O. V. Dolomanov, L. J. Bourhis, R. J. Gildea, J. A. K. Howard, H. Puschmann, *J. Appl. Crystallogr.* **2009**, *42*, 339–341.
[12] M. Piesch, S. Reichl, M. Seidl, G. Balázs, M. Scheer, *Angew. Chem. Int. Ed.* **2021**, *60*, 15101–15108.
[13] a) F. Neese, *WIREs Comput. Mol. Sci.* **2012**, *2*, 73–78; b) F. Neese, *WIREs Comput. Mol. Sci.* **2018**, *8*; c) F. Neese, F. Wennmohs, U. Becker, C. Riplinger, *J. Chem. Phys.* **2020**, *152*, 224108; d) F. Neese, *WIREs Comput. Mol. Sci.* **2022**, *12*; e) F. Neese, *J. Comput. Chem.* **2023**, *44*, 381–396;
[14] "Chemcraft - graphical software for visualization of quantum chemistry computations. https://www.chemcraftprog.com".
[15] a) D. Andrae, U. Huermann, M. Dolg, H. Stoll, H. Preu, *Theoret. Chim. Acta* **1990**, *77*, 123–141; b) V. Barone, M. Cossi, *J. Phys. Chem. A* **1998**, *102*, 1995–2001; c) F. Weigend, R. Ahlrichs, *Phys. Chem. Chem. Phys.* **2005**, *7*, 3297–3305; d) F. Weigend, *Phys. Chem. Chem. Phys.* **2006**, *8*, 1057–1065; e) J.-D. Chai, M. Head-Gordon, *Phys. Chem. Chem. Phys.* **2008**, *10*, 6615–6620; f) S. Grimme, S. Ehrlich, L. Goerigk, *J. Comput. Chem.* **2011**, *32*, 1456–1465; g) Y.-S. Lin, G.-D. Li, S.-P. Mao, J.-D. Chai, *J. Chem. Theory Comput.* **2013**, *9*, 263–272;
[16] E. D. Glendening, C. R. Landis, F. Weinhold, *J. Comput. Chem.* **2019**, *40*, 2234–2241.
[17] A. E. Reed, L. A. Curtiss, F. Weinhold, *Chem. Rev.* **1988**, *88*, 899–926.
[18] V. Ásgeirsson, B. O. Birgisson, R. Bjornsson, U. Becker, F. Neese, C. Riplinger, H. Jónsson, *J. Chem. Theory Comput.* **2021**, *17*, 4929–4945.
